# Chemospecific Cyclizations of α‐Carbonyl Sulfoxonium Ylides on Aryls and Heteroaryls

**DOI:** 10.1002/anie.201910821

**Published:** 2019-09-24

**Authors:** Daniel Clare, Benjamin C. Dobson, Phillip A. Inglesby, Christophe Aïssa

**Affiliations:** ^1^ Department of Chemistry University of Liverpool Crown Street Liverpool L69 7ZD UK; ^2^ Pharmaceutical Technology and Development AstraZeneca Macclesfield Campus Cheshire SK10 2NA UK

**Keywords:** chemospecifity, cyclization, hexafluoroisopropanol, iridium, sulfoxonium

## Abstract

The functionalization of aryl and heteroaryls using α‐carbonyl sulfoxonium ylides without the help of a directing group has remained so far a neglected area, despite the advantageous safety profile of sulfoxonium ylides. Described herein are the cyclizations of α‐carbonyl sulfoxonium ylides onto benzenes, benzofurans and *N*‐*p*‐toluenesulfonyl indoles in the presence of a base in HFIP, whereas pyrroles and *N*‐methyl indoles undergo cyclization in the presence of an iridium catalyst. Significantly, these two sets of conditions are chemospecific for each groups of substrates.

## Graphical abstract

**Despite an excellent safety profile** due to their low thermal potential, α‐carbonyl sulfoxonium ylides remain underexploited for the functionalization of (hetero)aryls without recourse to directing groups. Described is a cyclization of these valuable reagents on aryls and heteroaryls that is chemospecific, depending on the reaction conditions.

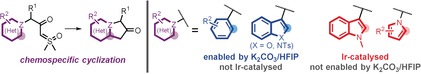

Sulfoxonium ylides have recently raised increased attention as potentially safer surrogates of diazo compounds in metal‐catalyzed reactions.^1^ The superior thermal stability of sulfoxonium ylides is evidenced by differential scanning calorimetry, which shows that α‐diazo ketone **1** is a potential explosive, whereas α‐carbonyl sulfoxonium ylide **2** is not.^2^

These advantageous features have prompted the development of efficient metal‐catalyzed carbon–heteroatom bond formations from sulfoxonium ylides,^3^, ^4^ notably in large‐scale industrial settings.^5^ Metal‐catalyzed reactions of α‐carbonyl sulfoxonium ylides have also been described for the formation of carbon–carbon bonds from carbon–hydrogen bonds.^6^, ^7^ However, most strategies rely on using a directing group.^6^ In contrast, examples of C−H functionalization that do not resort to a directing group are limited to the reactions of α,β‐unsaturated β‐amino‐esters,^8^ and this approach is therefore greatly underexploited. Clearly, sulfoxonium ylides have a great potential in synthesis, but its fulfilment depends on a greater understanding of the reactivity of these valuable reagents.

In an effort to address this issue, we now describe the cyclization of α‐carbonyl sulfoxonium ylides on aryl and heteroaryl fragments that is strikingly chemospecific (Figure 1 b). Thus, the cyclization of benzylic substrates was enabled by a combination of a base and HFIP (1,1,1,3,3,3‐hexafluoro‐2‐propanol), but it did not occur in the presence of a metal catalyst. Benzofurans and *N*‐*p*‐toluenesulfonyl indoles showed similar reactivity. In stark contrast, the cyclization of pyrroles and *N*‐methyl indoles occurred in the presence of [{Ir(cod)Cl}_2_] (cod: cyclooctadiene), but it was not enabled by the combination of HFIP and base. It is noteworthy that the bicyclic ketones obtained by these two methods are direct precursors of drug candidates (e.g. a glycine transporter inhibitor),^9^ as well as natural products ipabildine^10^ and indolizidine 167B,^11^ whose previous syntheses involved the cyclization of potentially more hazardous α‐diazo ketones.^12^, ^13^

**Figure 1:**
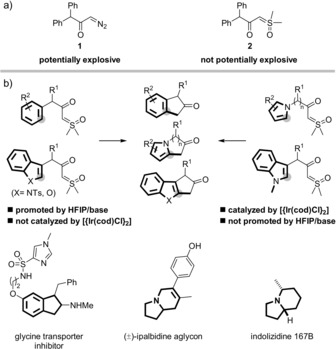
a) Comparison of safety profiles of a typical α‐carbonyl diazo compound and the related sulfoxonium ylide. b) Chemospecific cyclization of α‐carbonyl sulfoxonium ylides (this work) and examples of bioactive compounds and natural products prepared from analogous bicyclic ketones. HFIP: 1,1,1,3,3,3‐hexafluoro‐2‐propanol; cod: cyclooctadiene.

In the course of our studies on rhodium‐catalyzed C−H cross‐coupling of α‐carbonyl sulfoxonium ylides, we found that substrate **2** could undergo cyclization to **3** in the absence of metal catalysts when treated with a base in HFIP at 60 °C [Eq. (1)]. We observed that many bases could promote the reaction to a good extent (see Supporting Information). Among these bases, K_2_CO_3_ was selected for further studies on the grounds of cost and practicality. The reaction could proceed without base (Table 1, entry 1), but its presence led to a higher yield of **3**. Significantly, the presence of HFIP was essential, whereas the conversion remained null in other solvents (entry 2). Using HFIP as additive in another solvent partially restored the reactivity (entry 3). Replacing HFIP with additives of similar acidity left the starting material **2** intact (entries 4 and 5), which suggests that the protic nature of HFIP is not sufficient to explain the cyclization of **2** into indanone **3**.

**Table 1:** Most influential factors on the HFIP‐promoted cyclization of α‐carbonyl sulfoxonium ylide **2** into compound **3**. 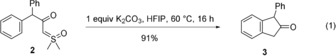

Entry	Variation of conditions^[a]^	Yield^[b]^
1	No base	63 %
2	K_2_CO_3_ (1 equiv), either TFE, i‐PrOH, or 1,2‐DCE	0 %^[c]^
3	No base, HFIP (5 equiv), 1,2‐DCE	20 %
4	No base, 2,6‐Me_2_‐C_6_H_3_OH (5 equiv), 1,2‐DCE	0 %^[c]^
5	No base, TMP⋅HCl (5 equiv), i‐PrOH	0 %^[c]^

[a] From those depicted in Equation (1). [b] Yield of isolated product. [c] The starting material was recovered in at least 90 % yield. 1,2‐DCE: 1,2‐dichloroethane; TFE: trifluoroethanol; TMP: 2,2,6,6‐tetramethylpiperidine.

With optimised conditions in hands, we examined the scope with substrates **4 a**–**o** (Figure 2). Indan‐2‐ones **5 a**–**f** were obtained in good yields, whereas gem‐dimethylated **4 g** failed to afford **5 g**. Nevertheless, we were pleased to observe that halogen‐substituted **4 d** and **4 e** underwent the cyclization to give **5 d** and **5 e** in excellent yield, although more forcing conditions were necessary, as was the case for the cyclization of electron‐poor substrate **4 f** into **5 f**. In the case of substrate **4 c**, the expected cyclization product **5 c** was obtained in 65 % yield alongside solvolysis product **6**. We then studied the regioselectivity of this cyclization with **4 h**–**n**, and observed excellent selectivity in the reactions that gave **5 h**–**m** in very good yields. Thus, no six‐membered ring was formed in the cyclization of **4 k** into **5 k**. However, the two possible regioisomers of **5 o** were obtained in an almost equimolar ratio.

**Figure 2:**
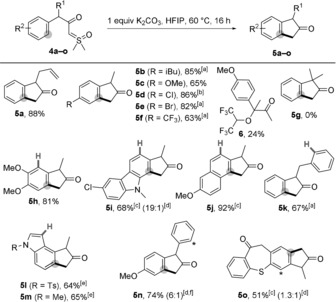
Cyclization of α‐carbonyl sulfoxonium ylides promoted by HFIP. All yields given are for isolated products from reactions conducted with 0.66 mmol of **4** (0.2 m). [a] At 90 °C. [b] Microwave heating at 80 °C for 1 hour. [c] 48 hours. [d] Ratio of regioisomers; the position of the alternative carbon‐carbon bond formation is denoted by an asterisk. [e] 0.1 mmol of **4**. [f] 0.26 mmol of **4**.

We propose the following mechanism to account for these results (Figure 3). In view of infrared spectra of substrates **4 a**–**o** (*ν*_(C=O)_: 1557–1572 cm^−1^), it is reasonable to consider intermediate **I** as starting point, and it would be in equilibrium with **II** and **III** under the conditions. Cyclization of **III** into **IV** by pathway (a) would lead to the observed products after rearomatization. However, several observations point to oxy‐allyl cation **V** as a plausible intermediate from **III** to **IV** and suggest pathway (b) as a possibly more likely alternative. Firstly, HFIP is a very strong H‐bond donor^14^ and it could promote the cleavage of the C−S bond in **III**. Furthermore, the combination of HFIP and a base has been reported to promote the formation of oxy‐allyl cations in the case of other leaving groups.^15^ Secondly, when keeping R^1^ as Me and varying R^2^ (H, *p*‐iBu, *p*‐OMe, *p*‐Cl), a good correlation (R^2^=0.97) of the relative rates with Hammett *σ*_p_ parameters^16^ was obtained and gave a reaction constant *ρ* of −0.6, in good agreement with those found for other examples of antarafacial five‐centres 4π‐electrocyclization of cationic intermediates.^17^ A similar mechanism could therefore be plausible for the rearrangement of **V** into **IV**. Thirdly, in the absence of substituent R^1^, and when R^2^ was *p*‐OMe, the equilibrium between **V** and **VI** led to solvolysis product **7** in 54 % yield as sole product of the reaction. This side reaction was only partly prevented when R^1^ was a methyl group, that is, in the case of **4 c**, and a mixture of **5 c** and **6** was obtained (Figure 2). Moreover, in the absence of substituent R^1^, and when R^2^ was *p*‐CF_3_, a Favorskii rearrangement via cyclopropanone **VII** led to **8** in 70 % yield as the sole product. The Favorskii rearrangement was completely prevented by the R^1^ substituent in the case of **4 f** (Figure 2). The proposed electrocyclization of an oxy‐allyl cation would also explain the absence of product **5 g** and the complete regioselectivity observed for **5 k**–**5 m**, as the other regioisomer cannot be following this mechanism.

**Figure 3:**
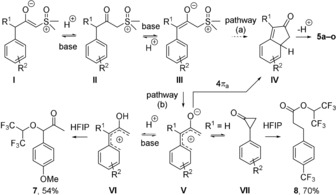
Plausible mechanism of the HFIP‐promoted cyclization of α‐carbonyl sulfoxonium ylides.

Moreover, this method was adapted to the cyclization of indole derivative **9** into **10** and **11**, whereby **10** appeared to be an intermediate, as evidenced by its conversion into **11** under the reaction conditions (Scheme 1). In contrast, benzofuran **12** gave only **13** in excellent yield.

**Scheme 1:**
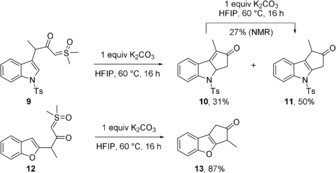
HFIP‐promoted cyclization of heterocyclic compounds. a) K_2_CO_3_ (1 equiv), HFIP, 60 °C, 16 h. All yields given are for isolated products except otherwise noted.

However, placing pyrrole **14** under the reaction conditions led to the solvolysis product **15** (Scheme 2). This result could be understood by considering that although an oxyallyl cation can be formed from **9**, **12**, and **14**, the latter cannot undergo a five‐centres 4π‐electrocyclization.

**Scheme 2:**
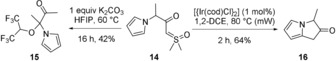
Solvolysis of pyrrole **14** in HFIP versus its Ir‐catalyzed cyclization. mW: microwave. All yields given are for isolated products.

The cyclization of a sulfoxonium ylide on a pyrrole has been described with a single example in a patent,^18^ but the reaction relied on using 10 mol % of [{Ir(cod)Cl}_2_] and, importantly, its scope was not examined. The failed cyclization of **14** spurred us to optimise the conditions of the Ir‐catalyzed reaction and to explore its chemoselectivity in the presence of potentially reactive C−H bonds in the context of the present study. Pleasingly, we could decrease the amount of catalyst to 1 mol % when heating the mixture in a microwave oven. Significantly, besides the cyclizations giving **18 a** and **18 b** (Figure 4), we were delighted to observe that the cyclizations of precursors **17 c**–**e** was very selective for the functionalization of the pyrrolic C−H bond. Thus, products **18 c**–**e** were obtained without any side‐products that would have resulted from aryl C−H insertion or Buchner reaction, or benzylic C−H insertion. Even more strikingly, six‐ and seven‐membered ring compounds **18 f** and **18 g** were obtained in 94 % and 90 % yield, respectively, without cyclization on the electron‐rich phenyl ring, and eight‐membered ring compound **18 h** could be obtained in good yield under iridium catalysis, without any side product besides the recovered starting material.

**Figure 4:**
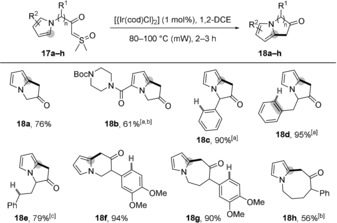
Functionalization of C−H bonds by iridium‐catalyzed cyclization of α‐carbonyl sulfoxonium ylides. All yields given are for isolated products. All reactions were performed on 0.3 mmol of substrate (0.02 m) unless otherwise noted. [a] 0.1 mmol of **17**. [b] 5 mol % [{Ir(cod)Cl}_2_], 3 hours. [c] 2.5 mol % [{Ir(cod)Cl}_2_], 100 °C.

Furthermore, this methodology is also applicable to the cyclization of indoles, as illustrated with the high‐yielding conversion of **19** into **20** (Figure 5), which was used as a precursor of a dual inhibitor of kinase phosphorylation.^19^

**Figure 5:**
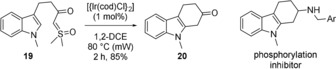
Reaction of indole **19** and structure of a dual inhibitor of kinase phosphorylation. Ar=*p*‐tolyl.

To gain an insight into the mechanism of the iridium‐catalyzed reaction, we turned to deuterium labelling (Figure 6 a). Thus, treatment of [D_1_]**21** with [{Ir(cod)Cl}_2_] gave [D_n_]**22** at full conversion, whereas parallel experiments with **21** and [D_1_]**21** gave no kinetic isotope effect (*k*_H_/*k*_D_=1.0). These result suggest that in the presence of the catalyst, the sulfoxonium ylide would give carbene **VIII**, and this intermediate could undergo a nucleophilic attack to give **IX**, that would in turn undergo rapid 1,2‐deuterium migration to give **X**, before a final elimination to **XI** and its facile re‐aromatization to the observed product, accompanied by intra‐ and intermolecular scrambling of the deuterium label (Figure 6 b). Alternatively, cyclopropanation of **VIII** to **XII** could also explain the observed labelling. Hence, collapse of **XII** into zwitterion **XIII** would deliver an intermediate that could also undergo 1,2‐migration to give **XI**.

**Figure 6:**
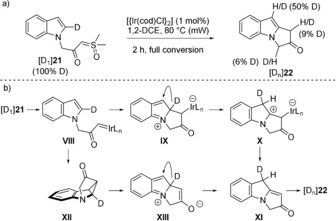
a) Deuterium‐labelling studies. b) Plausible mechanisms of the Ir‐catalyzed cyclization of α‐carbonyl sulfoxonium ylides.

Finally, we would like to emphasize the remarkable chemospecificity of the two methods of cyclization described herein. Significantly, neither **2**, **4 b**, nor **4 h** reacted when treated with [{Ir(cod)Cl}_2_] although the formation of an iridium carbene would have been conceivable. An even more striking difference of reactivity was observed in the case of structurally similar **23** and **25** that in principle can undergo cyclization by the agency of either an oxy‐allyl cation or an iridium carbene (Figure 7). Thus, the cyclization of *N*‐methyl indole **23** into **24** catalyzed by [{Ir(cod)Cl}_2_] was far more efficient than when the HFIP/K_2_CO_3_ conditions were applied, whereas an opposite result was observed in the cyclization of benzofuran **25** into isomers **26** and **27**.

**Figure 7:**
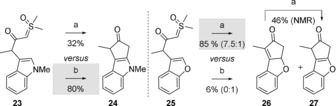
Chemospecific reactions of closely related substrates. a) K_2_CO_3_ (1 equiv), HFIP, 60 °C, 16 h. b) 1 mol % [{Ir(cod)Cl}_2_], 1,2‐DCE, 80 °C (mW), 2 h.

In conclusion, we have uncovered a strikingly chemospecific cyclization of α‐carbonyl sulfoxonium ylides on aryls and heteroaryls. This chemospecificity demonstrates that besides an advantageous safety profile, α‐carbonyl sulfoxonium ylides display a reactivity that is very distinct from that of α‐diazo ketones,^12^, ^13^ or other ylides and their precursors.^20^

## Conflict of interest

The authors declare no conflict of interest.

## Supporting information

As a service to our authors and readers, this journal provides supporting information supplied by the authors. Such materials are peer reviewed and may be re‐organized for online delivery, but are not copy‐edited or typeset. Technical support issues arising from supporting information (other than missing files) should be addressed to the authors.

Supplementary
