# Supplementary material for: Chemospecific Cyclizations of α‐Carbonyl Sulfoxonium Ylides on Aryls and Heteroaryls
Source: Angew Chem Int Ed Engl. 2019 Sep 24;58(45):16198–202. doi: 10.1002/anie.201910821 (PMC6856693; doi:10.1002/anie.201910821)
Supplement: Supplementary file 1 — Supplementary [file ANIE-58-16198-s001.pdf]

Angewandte Chemie
Eine Zeitschrift der Gesellschaft Deutscher Chemiker

Supporting Information

Chemospecific Cyclizations of α-Carbonyl Sulfoxonium Ylides on Aryls and Heteroaryls

*Daniel Clare, Benjamin C. Dobson, Phillip A. Inglesby, and Christophe Aïssa**

anie_201910821_sm_miscellaneous_information.pdf

General. Otherwise noted, all reactions were carried out in flame-dried glassware under dry nitrogen atmosphere. THF was used after passage through Innovative Technology PureSolv MD system. All commercially available compounds were used as received. Flash chromatography: Merck silica gel 60 (230-400 mesh). NMR: Spectra were recorded on a Bruker DRX 500 or DPX 400 in $CDCl_3$; chemical shifts (δ) are given in ppm. The solvent signals were used as references and the chemical shifts converted to the TMS scale ($CDCl_3$: δ_C = 77.0 ppm; residual $CHCl_3$ in $CDCl_3$: δ_H = 7.26 ppm); apparent splitting patterns are designated using the following abbreviations: s (singlet), d (doublet), t (triplet), q (quartet), quint. (quintuplet), m (multiplet), br (broad), and the appropriate combinations. In ^{13}C NMR, an APT sequence was used to separate methylene groups and quaternary carbons (e, even) from methine and methyl groups (o, odd). IR: PerkinElmer Spectrum 100 FT-IR spectrometer, wavenumbers ($\tilde{\nu}$) in cm^{-1}. HRMS determined at the University of Liverpool on micromass LCT mass spectrometer (ES+) and Trio-1000 or Agilent QTOF 7200 mass spectrometers (CI). Melting points: Griffin melting point apparatus (not corrected). Elemental analyses: Elementar Vario Micro Cube instrument at University of Liverpool.

Synthesis of sulfoxonium ylides **2**, **4a–g**, **4k**, and **4n**	S2
Synthesis of sulfoxonium ylides **4h–j**, **4l**, **4m**, **4o**, **9**, **12**, **14**, **17a–h**, **19, [D$_1$]-21**, **23**, **and 25**	S6
Synthesis of compounds **3**, **5a–f**, **5h–o**, **6–8**, **10**, **11**, **13**, **15**, **26**, and **27**	S26
Hammett study of the conversion of compounds **4** into compounds **5**	S32
Synthesis of compounds **16**, **18a–h**, **20**, **22**, and **24**	S33
Deuterium labeling experiments	S37
DSC traces of compounds **1** and **2**	S38
NMR spectra of compounds **2**, **4h–o**, **9**, **12**, **14**, **17a–h**, **19, 21, [D$_1$]-21**, **23**, **and 25**	S39
NMR spectra of compounds **5b–f**, **5h–o**, **6–8**, **10**, **11**, **13**, **15**, **16**, **18a–h**, **20**, **22**, **24**, **26**, **27**	S72

Synthesis of sulfoxonium ylides 2, 4a–g, 4k, and 4n

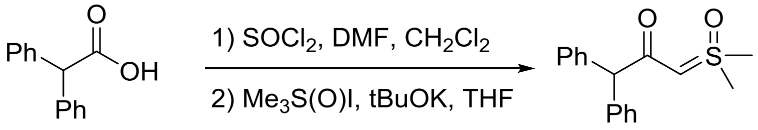

2

Representative procedure A – Under N_2, 2,2-diphenylacetic acid (10 g, 47.11 mmol) in CH_2Cl_2 (50 mL) at 0 °C before adding $SOCl_2$ (5 mL, 70.66 mmol, 1.5 equiv) and one drop of DMF. After 30 minutes stirring at r.t., a small aliquot quenched in MeOH confirmed the complete conversion of the acid, and the volatiles were carefully evaporated under high-vacuum using Schlenck techniques. During that time, trimethylsulfoxonium iodide (31.3 g, 141 mmol, 3 equiv) was suspended under N_2 in dry THF (280 mL) in a flame-dried 500 mL round bottom flask that was protected from light with aluminium foil. Potassium *tert*-butoxide (15.6 g, 141 mmol, 3 equiv) was added, and the mixture was stirred at reflux for 2 hours. After cooling to 0 °C, a solution of 2,2-diphenylacetyl chloride obtained above in THF (170 mL) was added dropwise to the mixture. The mixture was stirred at 0 °C for another hour and then filtered at room temperature through a plug of celite before all volatiles were removed under vacuum. Purification by recrystallisation from EtOAc afforded **2** as off-white solid (7.74 g, 27.02 mmol, 57%).

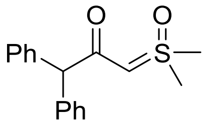

2

Compound 2. m.p.: 123–126 °C; ^{1}H NMR (500 MHz, $CDCl_3$): δ 7.30-7.29 (m, 8H), 7.23-7.20 (m, 2H), 4.85 (s, 1H), 4.38 (s, 1H), 3.38 (s, 6H); ^{13}C NMR (125 MHz, $CDCl_3$): δ 188.9 (e), 141.0 (e, 2C), 129.0 (o, 4C), 128.3 (o, 4C), 126.5 (o, 2C), 71.2 (o), 62.2 (o), 42.0 (e, 2C); IR (neat): $\tilde{\nu}$ = 3098 (w), 3083 (w), 3053 (w) 3005 (w), 2972 (w, 2918 (w, 1596 (w), 1559 (s), 1493 (m), 1458 (w), 1448 (w), 1431 (w), 1374 (s), 1322 (w), 1300 (w), 1257 (w), 1231 (w), 1167 (s), 1119 (s), 1071 (w), 1030 (s), 989 (w), 948 (w), 929 (w), 909 (w), 861 (s), 818 (w), 789 (w), 758 (w), 743 (s), 724 (s), 695 (s) cm^{-1}; HRMS (ESI): *m/z* calcd for $C_{17}H_{18}O_2S+H^+$: 287.1100 $[M+H]^+$; found 287.1103.

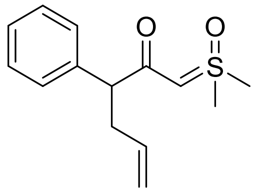

4a

Compound 4a. Synthesised according to representative procedure A from 2-phenylpent-4-enoic acid (2.70 g, 15.33 mmol) except for a purification by flash chromatography (EtOAc/MeOH = 97:3) to afford compound **4a** as an off-white solid (2.24 g, 8.95 mmol, 58%). m.p.: 88–90 °C; ^{1}H NMR (500 MHz, $CDCl_3$): δ 7.31–7.27 (m, 4H), 7.25–7.17 (m, 1H), 5.82–5.64 (m, 1H), 5.04 (dq, *J* = 17.1, 1.4 Hz), 4.94 (d, *J* = 10.3 Hz, 1H), 4.32 (s, 1H), 3.49–3.40 (m, 1H), 3.36 (s, 3H), 3.20 (s, 3H), 2.89–2.78 (m, 1H), 2.54–2.43 (m, 1H); ^{13}C NMR (100 MHz, $CDCl_3$): δ 190.0 (e), 141.2 (e), 136.7 (o), 128.3 (o, 2C), 128.0 (o, 2C), 126.5 (o), 115.8 (e), 69.3 (o), 56.2 (o), 42.2 (o), 42.1 (o), 37.2 (e); IR (neat): $\tilde{\nu}$ = 3069 (m), 3007 (m), 2920 (m), 1642 (w), 1601 (w), 1566 (s), 1497 (w), 1455 (w), 1426 (w), 1375 (s), 1303 (m), 1248 (w), 1167 (s), 1125 (m), 1105 (m), 1025 (s), 1001 (m), 985 (m), 945 (m), 912 (s), 884 (w), 853 (s), 774 (s), 750 (m), 724 (m), 700 (s), 675 (m) cm^{-1}; HRMS (CI(NH_3)): *m/z* calcd for $C_{14}H_{18}O_2S+H^+$: 251.1106 $[M+H]^+$; found: 251.1096; elemental analysis calcd (%) for $C_{14}H_{18}O_2S$: C 67.17, H 7.25, S 12.81; found: C 66.85, H 7.24, S 12.95.

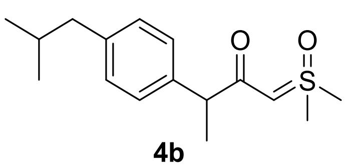

4b

Compound 4b. Synthesised according to representative procedure A from 2-(4-isobutylphenyl)propanoic acid (1 g, 4.84 mmol) except for a purification by flash chromatography (CH_2Cl_2/MeOH = 93:7) and trituration in Et_2O of the solid obtained after concentration of the product-containing fractions to afford compound **4b** as white solid (766 mg, 2.73 mmol, 56%). m.p.: 105–108 °C; ^{1}H NMR (500 MHz, $CDCl_3$): δ 7.18 (d, *J* = 8.1 Hz, 2H), 7.06 (d, *J* = 8.2 Hz, 2H), 4.27 (s, 1H), 3.49

(q, J = 7.2 Hz, 1H), 3.35 (s, 3H), 3.31 (s, 3H), 2.43 (d, J = 7.2 Hz, 2H); 1.84 (sept, J = 7.0 Hz, 1H); 0.89 (d, J = 6.7 Hz, 6H); ^{13}C NMR (125 MHz, $CDCl_3$): δ 192.2 (e), 140.3 (e), 139.6 (e), 129.0 (o, 2C), 127.2 (o, 2C), 68.9 (o), 49.9 (o), 45.0 (o); 42.04 (o), 41.93 (o), 30.1 (o), 22.3 (o, 2C), 18.3; IR (neat): $\tilde{\nu}$ = 3119 (w), 3081 (w), 3013 (w), 2958 (w), 2923 (w), 2867 (w), 2844 (w), 1562 (s), 1508 (w), 1463 (w), 1384 (s), 1332 (w), 1310 (m), 1252 (w), 1178 (s), 1164 (s), 1140 (s), 1086 (w), 1055 (w), 1033 (m), 999 (w), 947 (w), 930 (w), 892 (w), 861 (m), 844 (m), 811 (w), 772 (w), 754 (w), 746 (w), 707 (w), 674 (w), 664 (w) cm^{-1}; HRMS (ESI): *m/z* calcd for $C_{16}H_{24}O_2S+Na^+$: 303.1395 $[M+Na]^+$; found: 303.1391.

Compound 4c. Synthesised according to representative procedure A from 2-(4-methoxyphenyl)propanoic acid (1.49 g, 8.25 mmol) except for a purification by flash chromatography (CH_2Cl_2/MeOH = 98:2) and recrystallisation from EtOAc of the solid obtained after concentration of the product-containing fractions to afford compound **4c** as white powder (1.08 g, 4.25 mmol, 51%). m.p.: 90–92 °C; ^{1}H NMR (500 MHz, $CDCl_3$): δ 7.24 (d, J = 8.6 Hz, 2H), 6.86 (d, J = 8.6 Hz, 2H), 4.28 (s, 1H), 3.81 (s, 3H), 3.50 (q, J = 7.2 Hz, 1H), 3.38 (s, 3H), 3.34 (s, 3H), 1.45 (d, J = 7.2 Hz, 3H); ^{13}C NMR (125 MHz, $CDCl_3$): δ 192.3 (e), 158.2 (e), 135.4 (e), 128.6 (o, 2C), 113.8 (o, 2C), 68.5 (o), 55.2 (o), 49.5 (o), 42.2 (o), 42.1 (o), 18.5 (o); IR (neat): $\tilde{\nu}$ = 3068 (w), 3016 (w), 2968 (w), 2927 (w), 2834 (w), 1611 (w), 1563 (s), 1509 (s), 1462 (m), 1443 (w), 1428 (w), 1414 (w), 1374 (s), 1334 (m), 1303 (m), 1263 (m), 1247 (s), 1169 (s), 1158 (s), 1132 (s), 1113 (m), 1073 (w), 1060 (w), 1027 (s), 994 (m), 951 (w), 891 (w), 854 (s), 838 (m), 824 (w), 812 (m), 761 (s), 758 (w), 729 (m), 694 (w), 671 (w) cm^{-1}; HRMS (ESI): *m/z* calcd for $C_{13}H_{18}O_3S$ $+H^+$: 255.1049 $[M+H]^+$; found: 255.1055; elemental analysis calcd (%) for $C_{13}H_{18}O_3S$: C 61.39, H 7.13, S 12.60; found: C 61.35, H 7.17, S 12.53.

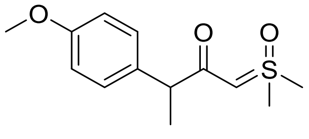

4c

Compound 4d. Synthesised according to representative procedure A from 2-(4-chlorophenyl)propanoic acid (1.13 g, 6.12 mmol) except for a purification by flash chromatography (CH_2Cl_2/MeOH = 98:2) and recrystallisation from EtOAc of the solid obtained after concentration of the product-containing fractions to afford compound **4d** as white powder (1.30 g, 5.04 mmol, 82%). m.p.: 106–110 °C; ^{1}H NMR (400 MHz, $CDCl_3$): δ 7.29–7.22 (m, 4H), 4.27 (s, 1H), 3.50 (q, J = 7.1 Hz, 1H), 3.36 (s, 3H), 3.32 (s, 3H), 1.42 (d, J = 7.2 Hz, 3H); ^{13}C NMR (125 MHz, $CDCl_3$): δ 191.1 (e), 141.7 (e), 132.1 (e), 129.0 (o, 2C), 128.4 (o, 2C), 68.9 (o), 49.6 (o), 42.2 (o), 42.0 (o), 18.3 (o); IR (neat): $\tilde{\nu}$ = 3068 (w); 3016 (w); 2968 (w); 2927 (w); 2834 (w); 1611 (w); 1563 (s); 1509 (s); 1462 (m); 1443 (w); 1428 (w); 1414 (w); 1374 (s); 1334 (m); 1303 (m); 1263 (m); 1247 (s); 1169 (s); 1158 (s); 1132 (s); 1113 (m); 1073 (w); 1060 (w); 1027 (s); 994 (m); 951 (w); 891 (w); 854 (s); 838 (m); 824 (w); 812 (m); 761 (s); 758 (w); 729 (m); 694 (w); 671 (w) cm^{-1}; HRMS (ESI): *m/z* calcd for $C_{12}H_{15}{}^{35}ClO_2S+Na^+$: 281.0379 $[M+Na]^+$; found: 281.0370; elemental analysis calcd (%) for $C_{12}H_{15}ClO_2S$: C 55.70, H 5.84, S 12.39; found: C 55.89, H 5.92, S 12.35.

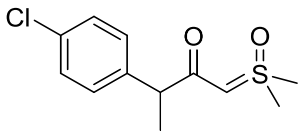

4d

Compound 4e. Synthesised according to representative procedure A from 2-(4-chlorophenyl)propanoic acid (3.49 g, 9.97 mmol) except for a purification by flash chromatography (CH_2Cl_2/MeOH = 98:2) to afford compound **4e** as white powder (1.21 g, 3.39 mmol, 40%). m.p.: 133–135 °C; ^{1}H NMR (500 MHz, $CDCl_3$): δ 7.41 (d, J = 8.2 Hz, 2H), 7.18 (d, J = 8.2 Hz, 2H), 4.27 (s, 1H), 3.49 (q, J = 7.2 Hz, 1H), 3.36 (s, 3H), 3.31 (s, 3H), 1.42 (d, J = 7.2 Hz, 3H); ^{13}C NMR (125 MHz, $CDCl_3$): δ 191.0 (e), 142.2 (e), 131.4 (o, 2C), 129.4 (o, 2C), 120.2 (e), 68.9 (o), 49.7 (o), 42.2 (o), 42.1 (o), 18.3 (o); IR (neat): $\tilde{\nu}$ = 3072 (w), 3018 (w), 3009 (w), 2982 (w), 2963 (w), 2923 (w), 2872 (w), 1564 (s), 1486 (m), 1460 (m), 1449 (w), 1421 (w), 1408 (m), 1376 (s), 1359 (s), 1337 (w), 1327 (w), 1303 (m), 1288 (w), 1267 (w), 1199 (w), 1164 (s), 1121 (s), 1074 (s), 1056 (m), 1023 (s), 1007 (s), 993 (m), 982 (m), 940 (m), 892 (w), 855 (s), 835 (s), 790 (m), 757 (m), 746 (m), 722 (m), 691 (w), 677 (m) cm^{-1}; HRMS (ESI): *m/z* calcd for

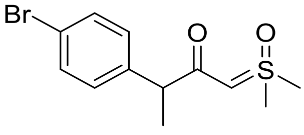

4e

$C_{12}H_{15}{}^{79}BrO_2S+Na^+$: 303.0049 [M+Na]$^+$; found: 303.0054; elemental analysis calcd (%) for $C_{12}H_{15}BrO_2S$: C 47.53, H 4.99, S 10.57; found: C 47.59, H 4.89, S 10.31.

Compound 4f. Synthesised according to representative procedure A from 2-(4-trifluoromethylphenyl)propanoic acid (647 mg, 2.97 mmol) except for a purification by flash chromatography (CH_2Cl_2/MeOH = 98:2) and trituration in Et_2O of the solid obtained after concentration of the product-containing fractions to afford compound **4f** as off-white powder (539 mg, 1.84 mmol, 62%). m.p.: 73–76 °C; ^{1}H NMR (400 MHz, $CDCl_3$): δ 7.55 (d, J = 8.1 Hz, 2H), 7.42 (d, J = 8.2 Hz, 2H), 4.31 (s, 1H), 3.59 (q, J = 7.2 Hz, 1H), 3.37 (s, 3H), 3.32 (s, 3H), 1.46 (d, J = 7.2 Hz, 3H); ^{13}C NMR (100 MHz, $CDCl_3$): δ 190.4 (e), 147.2 (e), 128.6 (e, q, J = 32.4 Hz), 127.9 (o, 2C), 126.6 (e), 125.2 (o, q, J = 3.6 Hz, 2C), 124.9 (e, q, J = 273 Hz), 69.2 (o), 50.1 (o), 42.1 (o), 42.0 (o), 18.3 (o); ^{19}F NMR (376.5 MHz, $CDCl_3$): δ -62.33 (s, 3F); IR (neat): $\tilde{\nu}$ = 3069 (w), 2991 (w), 2928 (w), 1617 (w), 1566 (s), 1463 (w), 1418 (w), 1376 (s), 1362 (m), 1322 (s), 1256 (w), 1161 (s), 1106 (s), 1069 (s), 1031 (s), 1017 (s), 988 (m), 953 (m), 857 (s), 846 (s), 804 (m), 777 (w), 760 (w), 747 (w), 715 (w), 695 (w), 675 (w) cm^{-1}; HRMS (ESI): *m/z* calcd for $C_{13}H_{15}F_3O_2S+H^+$: 293.0823 [M+H]$^+$; found: 293.0822; elemental analysis calcd (%) for $C_{13}H_{15}F_3O_2S$: C 53.42, H 5.17; found: C 53.57, H 5.18.

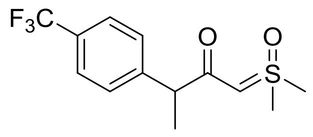

4f

Compound 4g. Synthesised according to representative procedure A from 2-methyl-2-phenylpropanoic acid (1.11 g, 6.08 mmol) except for a purification by flash chromatography (CH_2Cl_2/MeOH = 98:2) to afford compound **4g** as white solid (1.37 g, 5.75 mmol, 94%). m.p.: 92–93 °C; ^{1}H NMR (500 MHz, $CDCl_3$): δ 7.37-7.35 (m, 2H), 7.31-7.28 (m, 2H), 7.22-7.18 (m, 1H), 4.12 (s, 1H), 3.33 (s, 6H), 1.50 (s, 6H); ^{13}C NMR (125 MHz, $CDCl_3$): δ 195.7 (e), 147.2 (e), 128.0 (o, 2C), 126.2 (o, 2C), 125.9 (o), 63.4 (o), 48.8 (e), 41.9 (o, 2C), 26.8 (o, 2C); IR (neat): $\tilde{\nu}$ = 3094 (w), 3059 (w), 3001 (w), 2974 (w), 2963 (w), 2968 (w), 2926 (w), 2909 (w), 1572 (s), 1498 (m), 1471 (m), 1435 (w), 1412 (w), 1380 (m), 1351 (m), 1324 (m), 1306 (m), 1237 (w), 1191 (w), 1149 (s), 1115 (s), 1096 (s), 1071 (m), 1031 (s), 1004 (m), 982 (m), 946 (m), 912 (w), 875 (m), 857 (m), 847 (m), 779 (m), 765 (m), 748 (m), 730 (m), 696 (s), 679 (m) cm^{-1}; HRMS (ESI): *m/z* calcd for $C_{13}H_{18}O_2S+H^+$: 239.1100 [M+H]$^+$; found: 239.1101.

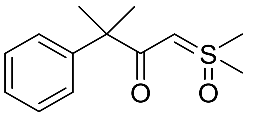

4g

Compound 4k. Synthesised according to representative procedure A from 2,3-diphenylpropanoic acid (2.86 g, 12.63 mmol) except for a purification by flash chromatography (CH_2Cl_2/MeOH = 98:2) to afford compound **4k** as cream-coloured solid (0.96 g, 3.19 mmol, 25%). m.p.: 75–76 °C; ^{1}H NMR (500 MHz, $CDCl_3$): δ 7.35-7.30 (m, 2H), 7.30–7.27 (m, 2H), 7.22-7.18 (m, 3H), 7.15-7.12 (m, 3H), 4.24 (s, 1H), 3.64 (dd, J = 8.9, 7.0 Hz, 1H), 3.44 (dd, J = 13.3, 8.4 Hz, 1H), 2.99-2.94 (m, 1H); ^{13}C NMR (125 MHz, $CDCl_3$): δ 189.5 (e), 141.4 (e), 140.5 (e), 129.1 (o, 2C), 128.3 (o, 2C), 128.0 (o, 2C), 128.0 (o, 2C), 126.5 (o), 125.8 (o), 69.7 (o), 58.2 (o), 42.1 (o, 2C), 39.4 (e); IR (neat): $\tilde{\nu}$ = 3065 (w): 3026 (w), 3007 (w), 2992 (w), 2941 (w), 2915 (w), 2855 (w), 1963 (w), 1732 (w), 1600 (w), 1568 (m), 1539 (s), 1493 (m), 1452 (m), 1377 (s), 1305 (m), 1242 (m), 1185 (s), 1171 (s), 1108 (m), 1071 (m), 1031 (s), 1003 (w), 991 (w), 963 (w), 945 (m), 895 (m), 861 (m), 831 (w), 801 (m), 770 (w), 760 (w), 745 (m), 733 (s) cm^{-1}; HRMS (ESI): *m/z* calcd for $C_{18}H_{20}O_2S+Na^+$: 323.1082 [M+Na]$^+$; found: 323.1079.

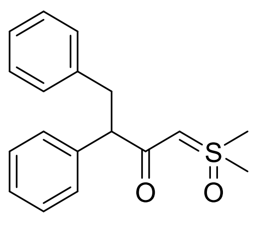

4k

Compound 4n. Synthesised according to representative procedure A from 2-(4-methoxyphenyl)-2-phenylacetic acid (414 mg, 1.14 mmol) except for a purification by flash chromatography (CH_2Cl_2/MeOH = 98:2) and recrystallisation from EtOAc of the solid obtained after concentration of the product-containing fractions to afford compound **4n** as white solid (133 mg, 0.42 mmol, 37%). m.p.: 120–123 °C; ^{1}H NMR (400 MHz, $CDCl_3$): δ 7.32–7.25 (m, 4H), 7.24–7.17 (m, 3H), 6.83 (d, J = 8.6 Hz, 2H), 4.79 (s, 1H), 4.37 (s, 1H), 3.78 (s, 3H), 3.38 (s, 6H); ^{13}C NMR (100 MHz, $CDCl_3$): δ 189.3 (e), 158.2 (e), 141.4 (e), 133.2 (e), 129.9 (o, 2C), 128.9 (o, 2C), 128.3 (o, 2C), 126.4 (o), 113.7 (o, 2C), 71.0 (o), 61.3 (o), 55.2 (o), 42.1 (o, 2C); IR (neat): $\tilde{\nu}$ = 3065 (w), 3011 (w), 2920 (w), 2836 (w), 2051 (w), 1610 (w), 1567 (s), 1509 (s), 1492 (m), 1465 (w), 1455 (w), 1443 (w), 1425 (w), 1373 (s), 1300 (m), 1248 (s), 1167 (s), 1110 (m), 1026 (s), 982 (w), 939 (w), 923 (w), 883 (w), 868 (m), 844 (m), 834 (m), 805 (w), 784 (m), 770 (w), 753 (w), 739 (m), 728 (m), 701 (m), 672 (m) cm^{-1}; HRMS (ESI): *m/z* calcd for $C_{18}H_{20}O_3S+Na^+$: 339.1031 $[M+Na]^+$; found: 339.1025; elemental analysis calcd (%) for $C_{18}H_{20}O_3S$: C 68.33, H 6.37, S 10.13; found: C 68.22, H 6.33, S 10.18.

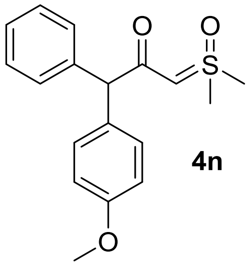

4n

Synthesis of sulfoxonium ylides 4h–j, 4l, 4m, 4o, 9, 12, 14, 17a–h, 19, [D1]-21, 23, and 25

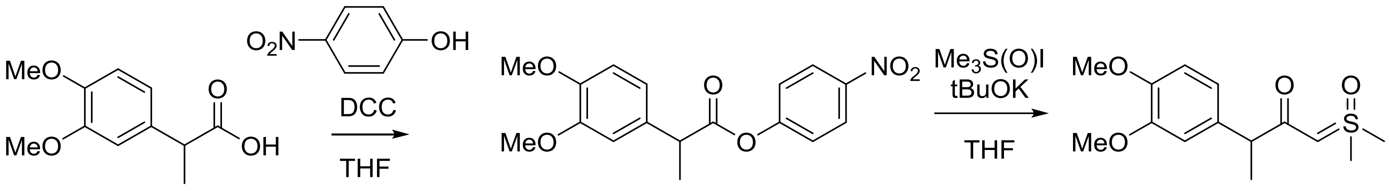

Representative procedure B – 2-(3,4-dimethoxyphenyl)propanoic acid (1.24 g, 5.90 mmol, 1 equiv) and 4-nitrophenol (1.07 g, 7.67 mmol, 1.3 equiv) were dissolved in THF (33 mL) under air. Dicyclohexylcarbodiimde (DCC) (1.58 g, 7.67 mmol, 1.3 equiv) was added in one portion, and the reaction was allowed to stir at r.t. for 18 h. The reaction mixture was then filtered through Celite, and the pad was washed with CH_2Cl_2. The filtrate was concentrated under vacuum, and the crude residue was purified by flash chromatography on SiO_2 (CH_2Cl_2) to afford **S-1** as a yellow amorphous solid (3.49 g, 9.69 mmol, 62%).

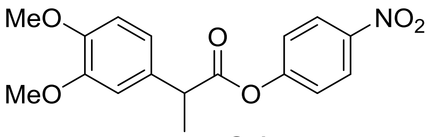

S-1

Compound S-1. ^{1}H NMR (500 MHz, $CDCl_3$): δ 8.23 (d, J = 9.3 Hz, 2H), 7.18 (d, J = 9.3 Hz, 2H), 6.94 (dd, J = 8.1, 2.0 Hz, 1H), 6.90 (d, J = 2.2 Hz, 1H), 6.88 (d, J = 8.3 Hz, 2H), 3.93 (q, J = 7.3 Hz, 1H), 3.90 (s, 3H), 3.89 (s, 3H), 1.62 (d, J = 7.2 Hz, 3H); ^{13}C NMR (125 MHz, $CDCl_3$): δ 172.3 (e), 155.5 (e), 149.2 (e), 148.5 (e), 141.6 (e), 131.7 (e), 125.1 (o, 2C), 122.2 (o, 2C), 119.6 (o), 111.4 (o), 110.5 (o), 55.91 (o), 55.87 (o), 45.1 (o), 18.3 (o); IR (neat): $\tilde{\nu}$ = 3081 (w), 2936 (w), 2836 (w), 2019 (w), 1755 (s), 1614 (w), 1591 (w), 1516 (s), 1489 (s), 1454 (m), 1419 (w), 1345 (s), 1253 (m), 1235 (m), 1205 (s), 1143 (s), 1070 (s), 1026 (s), 918 (w), 891 (w), 863 (m), 810 (w), 782 (w), 765 (w), 751 (w), 718 (w), 682 (w) cm^{-1}; HRMS (ESI): m/z calcd for $C_{17}H_{17}NO_6$+Na^+: 354.0948 [M+Na]$^+$; found: 354.0956.

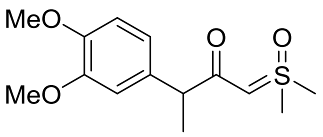

4h

Compound 4h. Synthesised according to the previously described method[1] from 4-nitrophenyl ester **S-1** (1.24 g, 3.74 mmol) and a purification by flash chromatography (CH_2Cl_2/MeOH = 98:2) to afford compound **4h** as pale-green amorphous solid (1.08 g, 3.79 mmol, quant). ^{1}H NMR (500 MHz, $CDCl_3$): δ 6.86-6.77 (m, 3H), 4.28 (s, 1H), 3.87 (s, 3H), 3.85 (s, 3H), 3.47 (q, J = 7.5 Hz, 1H), 3.36 (s, 3H), 3.32 (s, 3H), 1.43 (d, J = 7.2 Hz, 3H); ^{13}C NMR (125 MHz, $CDCl_3$): 192.1 (e), 148.7 (e), 147.5 (e), 135.9 (e), 119.5 (o), 111.1 (o), 110.9 (o), 68.7 (o), 55.80 (o), 55.78 (o), 49.9 (o), 42.1 (o), 42.0 (o), 18.4 (o); IR (neat): $\tilde{\nu}$ = 3431 (w), 3007 (w), 2964 (w), 2926 (w), 2835 (w), 2251 (w), 1567 (s), 1513 (s), 1453 (m), 1416 (m), 1375 (s), 1300 (w), 1260 (s), 1232 (s), 1174 (s), 1141 (s), 1077 (w), 1057 (w), 1025 (w), 944 (w), 916 (w), 856 (m), 812 (w), 778 (w), 762 (w), 730 (w), 676 (w) cm^{-1}; HRMS (ESI): m/z calcd for $C_{14}H_{20}O_4S$+H^+: 285.1155 [M+H]$^+$; found: 285.1154.

---

[1] Barday, M.; Janot, C.; Halcovitch, N. R.; Muir, J.; Aïssa, C. *Angew. Chem. Int. Ed.* **2017**, *56*, 13117–13121.

Compound S-2. Synthesised according to representative procedure B from *N*-Me-carprofen[2] (598 mg, 2.08 mmol) to afford compound **S-2** as yellow solid (645 mg, 1.58 mmol, 76%). The compound was contaminated by some DCC, and was characterized on ly by ^{1}H NMR before being used without further purification in the next step. ^{1}H NMR (500 MHz, $CDCl_3$): δ 8.21 (d, *J* = 9.2 Hz, 2H), 8.04 (d, *J* = 4.5 Hz, 1H), 8.03 (d, *J* = 1.4 Hz, 1H), 7.43 (dd, *J* = 8.6, 2.1 Hz, 1H), 7.40 (d, *J* = 1.0 Hz, 1H), 7.32 (d, *J* = 8.7 Hz, 1H), 7.26 (dd, *J* = 8.3, 1.5 Hz, 1H), 7.18 (d, *J* = 9.2 Hz, 2H), 4.19 (q, *J* = 7.2 Hz, 1H), 3.86 (s, 3H), 1.74 (d, *J* = 7.2 Hz, 3H).

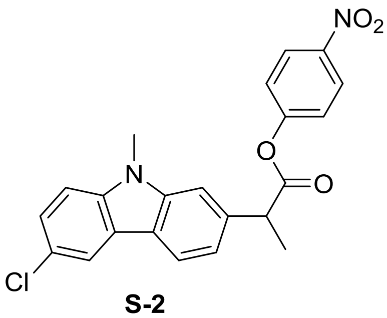

S-2

Compound 4i. Synthesised according to the previously described method[1] from 4-nitrophenyl ester **S-2** (645 mg, 1.58 mmol) and a purification by flash chromatography (CH_2Cl_2/MeOH = 90:10) to afford compound **4i** as white powder (1.21 g, 3.39 mmol, 40%). m.p.: 133–135 °C; ^{1}H NMR (500 MHz, $CDCl_3$): δ 7.99 (d, *J* = 1.9 Hz, 1H), 7.94 (d, *J* = 8.1 Hz, 1H), 7.39 (dd, *J* = 2.0, 8.4 Hz, 1H), 7.34 (s, 1H), 7.28 (d, *J* = 8.7 Hz, 1H), 7.18 (d, *J* = 8.4 Hz, 1H), 4.34 (s, 1H), 3.82 (s, 3H), 3.74 (q, *J* = 7.0 Hz, 1H), 3.36 (s, 3H), 3.31 (s, 3H), 1.57 (d, *J* = 7.2 Hz, 3H); ^{13}C NMR (125 MHz, $CDCl_3$): δ 191.9 (e), 142.2 (e), 141.8 (e), 139.5 (e), 125.3 (o), 124.3 (e), 123.8 (e), 120.4 (e), 120.3 (o), 119.7 (o), 119.4 (o), 109.3 (o), 107.6 (o), 68.9 (o), 51.1 (o), 42.2 (o), 42.1 (o), 29.2 (o), 18.7 (o); IR (neat): $\tilde{\nu}$ = 3022 (w), 2922 (w), 2552 (w), 1630 (w), 1600 (w), 1556 (s), 1493 (w), 1466 (s), 1415 (m), 1369 (s), 1356 (s), 1334 (m), 1307 (w), 1172 (s), 1140 (m), 1072 (w), 1063 (w), 1026 (s), 995 (m), 957 (w), 923 (w), 849 (m), 835 (w), 810 (s), 796 (s), 759 (w), 759 (w), 725 (w), 686 (w) cm^{-1}; HRMS (ESI): *m/z* calcd for $C_{19}H_{20}{}^{35}ClNO_2S+Na^+$: 384.0801 $[M+Na]^+$; found: 384.0790; elemental analysis calcd (%) for $C_{19}H_{20}ClNO_2S$: C 63.06, H 5.57, N 4.03, S 9.22; found: C 62.76, H 5.59, N 3.73, S 9.21.

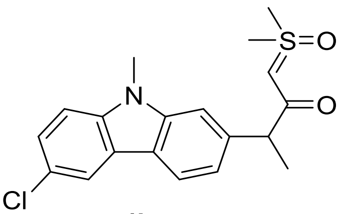

4i

Compound S-3. Synthesised according to representative procedure B from racemic naproxen (1 g, 4.34 mmol) to afford compound **S-3** as white solid (1.21 g, 3.39 mmol, 40%). m.p.: 133–135 °C; ^{1}H NMR (500 MHz, $CDCl_3$): δ 8.21 (d, *J* = 9.2 Hz, 2H), 7.79-7.72 (m, 3H), 7.47 (dd, *J* = 8.5, 2.0 Hz, 1H), 7.20-7.14 (m, 4H), 4.12 (q, *J* = 7.0 Hz, 1H), 3.93 (s, 3H), 1.71 (d, *J* = 7.2 Hz, 3H); ^{13}C NMR (125 MHz, $CDCl_3$): δ 172.2 (e), 157.8 (e), 155.5 (e), 145.2 (e), 134.3 (e), 133.9 (e), 129.2 (o), 128.9 (e), 127.5 (o), 126.1 (o), 125.8 (o), 125.0 (o), 122.2 (o), 119.2 (o), 105.6 (o), 55.2 (o), 45.5 (o), 18.2 (o); IR (neat): $\tilde{\nu}$ = 3116 (w), 3080 (w), 2988 (w), 2937 (w), 2459 (w), 2117 (w), 1924 (w), 1754 (s), 1632 (w), 1616 (w), 1603 (m), 1592 (m), 1533 (m), 1523 (s), 1505 (m), 1489 (m), 1463 (w), 1452 (w), 1417 (w), 1390 (w), 1377 (w), 1345 (s), 1325 (m), 1290 (w), 1264 (m), 1227 (m), 1205 (s), 1161 (s), 1127 (s), 1083 (w), 1067 (s), 1030 (m), 1012 (m), 1012 (w), 994 (w), 958 (w), 927 (w), 899 (m), 853 (s), 814 (m), 789 (m), 754 (w), 740 (m), 700 (m), 675 (w), 657 (w) cm^{-1}; HRMS (CI(CH_4)): *m/z* calcd for $C_{20}H_{17}NO_5+H^+$: 352.1185 $[M+H]^+$; found: 352.1183.

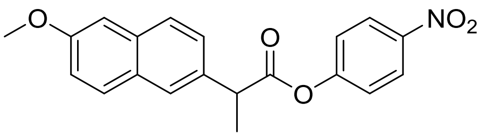

S-3

Compound 4j. Synthesised according to the previously described method[1] from 4-nitrophenyl ester **S-2** (1.20 g, 3.41 mmol) and a purification by flash chromatography (CH_2Cl_2/MeOH = 98:2) to afford compound **4j** as off-white solid (855 mg, 2.81 mmol, 82%). m.p.: 118–119 °C; ^{1}H NMR (500 MHz, $CDCl_3$): δ 7.70-

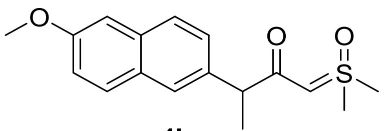

4j

[2] Favia, A. D.; Habrant, D.; Scarpelli, R.; Migliore, M.; Albani, C.; Bertozzi, S. M.; Dionisi, M.; Tarozzo, G.; Piomelli, D.; Cavalli, A.; De Vivo, M. *J. Med. Chem.* **2012**, 55, 8807-8826.

7.65 (m, 3H), 7.41 (dd, J = 8.5, 2.0 Hz, 1H), 7.13-7.10 (m, 2H), 4.30 (s, 1H), 3.91 (s, 3H), 3.66 (q, J = 7.1 Hz, 1H), 3.36 (s, 3H), 3.30 (s, 3H), 1.52 (d, J = 7.1 Hz, 3H); ^{13}C NMR (125 MHz, $CDCl_3$): δ 192.0 (e), 157.3 (e), 138.5 (e), 133.3 (e), 129.2 (o), 129.0 (e), 126.79 (o), 126.76 (o), 125.7 (o), 118.6 (o), 105.5 (o), 69.1 (o), 55.2 (o), 50.3 (o), 42.1 (o), 42.0 (o), 18.2 (o); IR (neat): $\tilde{\nu}$ = 3008 (w), 2977 (w), 2919 (w), 1630 (w), 1604 (m), 1564 (s), 1504 (m), 1481 (w), 1439 (w), 1417 (w), 1371 (s), 1308 (w), 1292 (w), 1264 (m), 1227 (m), 1193 (w), 1164 (s), 1070 (m), 1024 (s), 944 (w), 927 (w), 892 (w), 854 (s), 818 (m), 785 (w), 754 (w), 742 (w), 692 (w), 674 (w) cm^{-1}; HRMS (ESI): m/z calcd for $C_{17}H_{20}O_3S+H^+$: 305.1211 $[M+H]^+$; found: 305.1207; elemental analysis calcd (%) for $C_{17}H_{20}O_3S$: C 67.08, H 6.62, S 10.53; found: C 66.83, H 6.57, S 10.54.

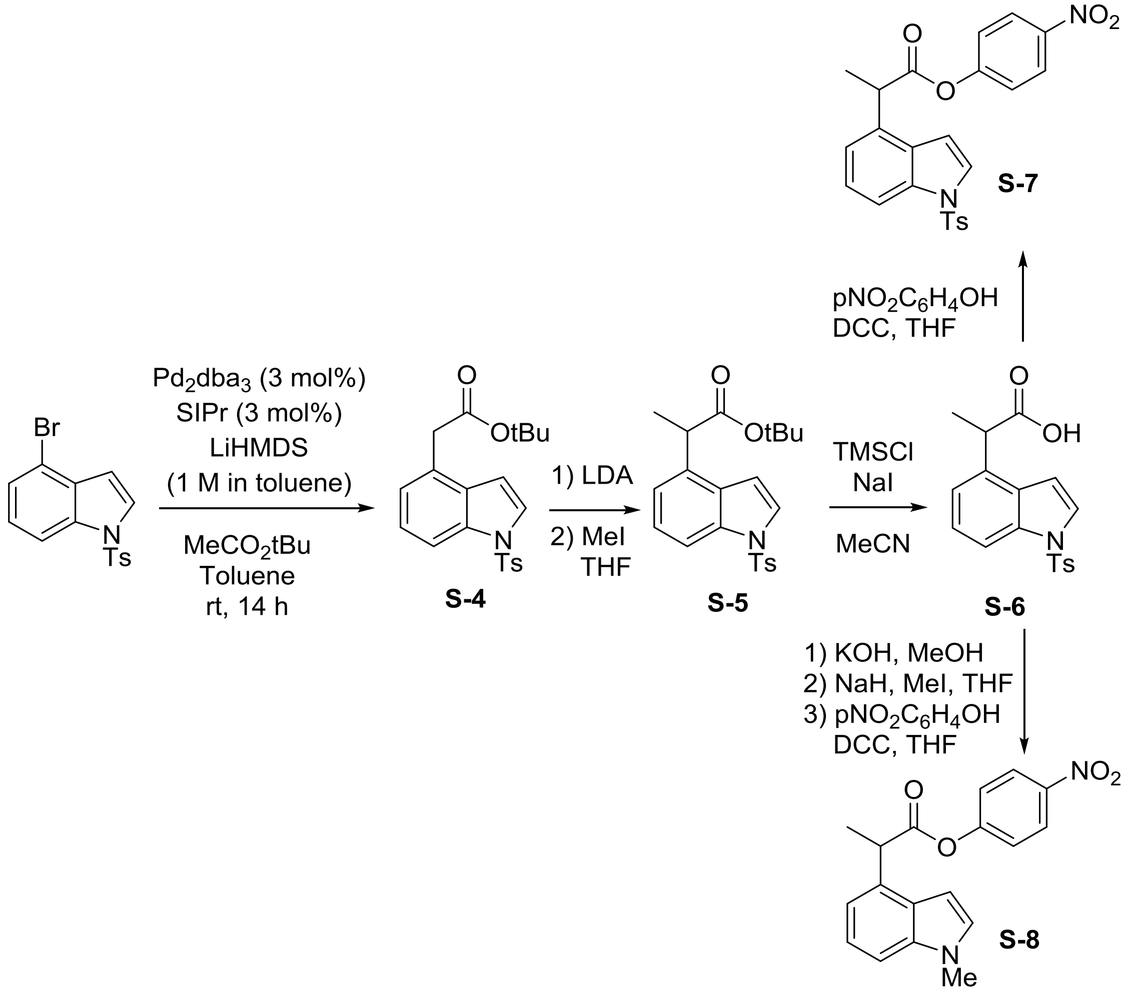

Compound S-4. SIPr (0.04 g, 0.09 mmol, 3 mol%) and $Pd_2(dba)_3$ (0.09 g, 0.09 mmol, 3 mol%) were weighed into a flame dried 2-neck flask, which was then evacuated and backfilled with N_2 three times. LiHMDS (1 M in toluene, 7 mL, 7.22 mmol, 2.3 equiv) was added, followed by compound *N*-Ts-4-bromo-indole (1.10 g, 3.14 mmol, 1.0 equiv), tert-butyl acetate (0.5 mL, 4.08 mmol, 1.3 equiv) and toluene (7 mL, 0.44 M). The reaction was left to stir overnight at room temperature, then partitioned between an aqueous saturated solution of NH_4Cl and Et_2O. The layers were separated and the aqueous layer was washed 3 times with Et_2O. The combined organic layers were washed with brine, dried over $MgSO_4$, filtered, and concentrated under vacuum. The residue was purified by flash chromatography (petroleum ether/ethyl acetate, 93:7) to afford compound **S-4** as a pale brown viscous oil (0.861 g, 2.23 mmol, 71% yield). ^{1}H NMR (500 MHz, $CDCl_3$): δ 7.89 (d, J = 8.4 Hz, 1H), 7.76 (d, J = 8.6 Hz, 2H), 7.57 (d, J = 4.1 Hz, 1H), 7.27-7.24 (m, 1H), 7.21 (d, J = 8.0 Hz, 2H), 7.11 (dd, J = 7.4, 0.8 Hz, 1H), 6.72 (dd, J = 3.7, 0.8 Hz, 1H), 3.70 (s, 2H), 2.34 (s, 3H), 1.37 (s, 9H); ^{13}C NMR (125 MHz, $CDCl_3$): δ 170.3 (e), 144.9 (e), 135.3 (e), 134.8

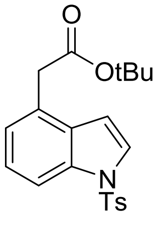

S-4

(e), 130.4 (e), 129.8 (o, 2C), 127.6 (e), 126.8 (o, 2C), 126.1 (o), 124.6 (o), 124.1 (o), 112.4 (o), 107.4 (o), 81.0 (e), 40.2 (e), 27.9 (o, 3C), 21.5 (o); IR (neat): $\tilde{\nu}$ = 3123 (w), 3035 (w), 2976 (w), 1690 (s), 1597 (w), 1520 (w), 1452 (m), 1460 (m), 1424 (s), 1370 (s), 1312 (m), 1279 (s), 1247 (s), 1212 (w), 1177 (s), 1163 (s), 1147 (s), 1124 (s), 1105 (s), 1088 (m), 1079 (m), 1029 (m), 998 (w), 972 (w), 938 (m), 901 (w), 884 (w), 857 (w), 833 (m), 812 (w), 787 (w), 754 (s), 741 (s), 724 (m), 703 (w), 687 (w), 653 (w), 668 (s) cm^{-1}; HRMS (ESI): *m/z* calcd for $C_{21}H_{23}NO_4S+Na^+$: 408.1240 $[M+Na]^+$; found: 408.1242.

Compound S-5. iPr_2NH (1.5 mL, 11.0 mmol, 1.8 equiv) was dissolved in THF (9 mL) and cooled to -10 °C using an ice/brine bath, and nBuLi (2.5 M in hexanes, 4 mL, 1.6 equiv, 9.8 mmol) was added dropwise. The reaction was stirred at -10 °C for 15 minutes, then **S-4** (2.4 g, 6.1 mmol, 1.0 equiv) was added dropwise. The reaction was stirred at -10 °C for 30 minutes, then MeI (0.8 mL, 12.3 mmol, 2.0 equiv) was added. The reaction was stirred at room temperature for 1 h, then quenched with aqueous saturated solution of NH_4Cl. The layers were separated, and the aqueous layer was washed 3 times with Et_2O. The combined organic layers were washed with brine, dried over $MgSO_4$, filtered, and concentrated under vacuum. The residue was purified by flash chromatography (petroleum ether/EtOAc, 94:6) to afford **S-5** as a pale-yellow oil (471 mg, 1.2 mmol, 19%). ^1H NMR (500 MHz, $CDCl_3$): δ 7.88 (dt, *J* = 8.3, 0.8 Hz, 1H); 7.76 (d, *J* = 8.4 Hz, 2H); 7.57 (d, *J* = 3.8 Hz, 1H); 7.28-2.4 (m, 1H); 7.22 (d, *J* = 8.0 Hz, 2H); 7.15 (d, *J* = 7.5 Hz, 1H); 6.78 (dd, *J* = 3.8, 0.7 Hz, 1H); ^{13}C NMR (125 MHz, $CDCl_3$): δ 173.3 (e), 144.8 (e), 135.3 (e), 134.8 (e), 134.0 (e), 129.8 (o, 2C), 129.6 (e), 126.8 (o, 2C), 124.7 (o), 121.2 (o), 112.1 (o), 107.4 (o), 80.7 (e), 43.8 (o), 27.8 (o, 3C), 21.5 (o), 17.4 (o); IR (neat): $\tilde{\nu}$ = 2977 (w), 2934 (w), 1723 (s), 1597 (m), 1528 (w), 1482 (w), 1455 (w), 1423 (w), 1392 (w), 1366 (s), 1326 (m), 1307 (w), 1282 (m), 1254 (m), 1214 (m), 1179 (s), 1164 (s), 1151 (s), 1133 (s), 1089 (s), 1022 (m), 1000 (m), 930 (w), 890 (w), 847 (m), 812 (m), 759 (s), 703 (m), 679 (s) cm^{-1}; HRMS (ESI): *m/z* calcd for $C_{22}H_{25}NO_4S+Na^+$: 422.1397 $[M+Na]^+$; found: 422.1396.

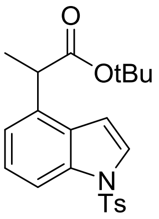

S-5

Compound S-6. Substrate **S-5** (1.39 g, 3.48 mmol, 1.0 equiv) and NaI (1.04 g, 6.97 mmol, 2 equiv) were dissolved in MeCN (3.5 mL) under N_2, and TMSCl (0.9 mL, 6.97 mmol, 2 equiv) was added. The reaction was heated at 45 °C for 2 h, then cooled to rt and quenched with water (9 mL) and extracted 3 times with EtOAc. The combined organic layers were washed with water and then with saturated aqueous sodium thiosulphate solution to remove inorganic salts and iodine, respectively, and the organic layer was dried over $MgSO_4$, filtered, and concentrated under vacuum. The residue was purified by flash chromatography (CH_2Cl_2/MeOH, 98:2) to afford **S-6** (589 mg, 1.71 mmol, 50% yield) as cream-coloured solid, whose identity was confirmed by ^1H NMR and HRMS only. ^1H NMR (500 MHz, $CDCl_3$): δ 7.90 (dt, *J* = 8.4, 0.8 Hz, 1H), 7.77 (d, *J* = 8.4 Hz, 2H), 7.59 (d, *J* = 3.7 Hz, 1H), 7.28 (d, *J* = 7.7 Hz, 1H), 7.23 (d, *J* = 8.1 Hz, 2H), 7.18 (d, *J* = 7.5 Hz, 1H), 6.76 (dd, *J* = 3.8, 0.7 Hz, 1H), 4.04 (q, *J* = 7.5 Hz, 1H), 2.35 (s, 3H), 1.55 (d, *J* = 7.2 Hz, 3H); HRMS (ESI): *m/z* calcd for $C_{18}H_{17}NO_4S+Na^+$: 366.0770 $[M+Na]^+$; found: 366.0771.

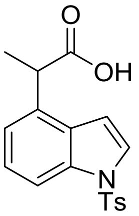

S-6

Compound S-7. Synthesised according to representative procedure B from **S-6** (361 mg, 1.05 mmol) to afford compound **S-7** as pale green amorphous solid (393 mg, 0.85 mmol, 80%). ^1H NMR (500 MHz, $CDCl_3$) δ 8.18 (d, *J* = 9.3 Hz, 2H), 7.97 (d, *J* = 8.5 Hz, 1H), 7.80 (d, *J* = 8.5 Hz, 2H), 7.65 (d, *J* = 3.7 Hz, 1H), 7.34 (t, *J* = 7.7 Hz, 1H), 7.26-7.23 (m, 2H), 7.06 (d, *J* = 9.0 Hz, 2H), 6.83 (d. *J* = 3.7 Hz, 1H), 4.29 (q, *J* = 7.1 Hz, 1H), 2.36 (s, 3H), 1.67 (d, *J* = 7.1 Hz, 3H); ^{13}C NMR (100 MHz, $CDCl_3$): δ 171.8 (e), 155.4 (e), 141.2 (e), 135.3 (e), 135.0 (e), 132.0 (e), 130.0 (o, 2C), 129.3 (e), 127.0 (o, 2C), 126.7 (o), 125.1 (o, 2C), 125.0 (o), 122.3 (o, 2C), 121.6 (o), 112.9 (o), 106.4 (o), 43.2 (o), 21.6 (o), 17.5 (o); IR (neat): $\tilde{\nu}$ = 3117 (w), 3084 (w), 2987 (w), 2856 (w), 2114 (w), 1757 (s), 1615 (w), 1592 (s), 1522 (s), 1489 (w), 1450 (w), 1424 (w), 1401 (w), 1373 (m), 1360 (m), 1344 (s), 1324 (w), 1307 (w), 1284

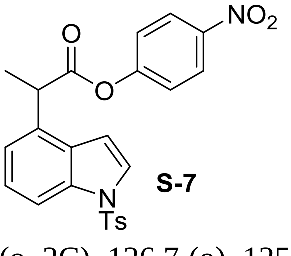

S-7

(w), 1203 (m), 1179 (s), 1164 (s), 1130 (s), 1087 (s), 1074 (s), 1053 (s), 1021 (m), 1013 (m), 996 (m), 952 (w), 923 (w), 892 (m), 864 (m), 838 (w), 812 (w), 759 (s), 712 (w), 702 (w), 674 (s) cm^{-1}; HRMS (CI(NH_3): *m/z* calcd for $C_{24}H_{20}N_2O_6S+H^+$: 465.1120 $[M+H]^+$; found: 465.1119.

Compound 4l. Synthesised according to the previously described method[1] from 4-nitrophenyl ester **S-7** (1.20 g, 3.41 mmol) and a purification by flash chromatography (CH_2Cl_2/MeOH = 98:2) followed by trituration in Et_2O in of the solid obtained after concentration of the product-containing fractions to afford compound **4l** as white powder (855 mg, 2.81 mmol, 82%). m.p.: 113–116 °C; ^{1}H NMR (500 MHz, $CDCl_3$): δ 7.84 (d, *J* = 8.4 Hz, 1H), 7.78 (d, *J* = 8.5 Hz, 2H), 7.55 (d, *J* = 3.8 Hz, 1H), 7.25–7.21 (m, 2H), 7.17 (d, *J* = 7.5 Hz, 1H), 6.79 (dd, *J* = 3.7, 0.7 Hz, 1H), 4.17 (s, 1H), 3.83 (q, *J* = 7.2 Hz, 1H), 3.31 (s, 3H), 3.26 (s, 3H), 2.35 (s, 3H), 1.49 (d, *J* = 7.2 Hz, 3H) (one multiplet (1H) is masked by residual $CHCl_3$); ^{13}C NMR (125 MHz, $CDCl_3$): δ 191.2 (e), 144.9 (e), 136.2 (e), 135.3 (e), 134.7 (e), 129.8 (o, 2C), 129.7 (e), 126.9 (o, 2C), 125.7 (o), 124.7 (o), 121.4 (o), 111.6 (o), 107.3 (o), 70.0 (o), 47.9 (o), 42.1 (o), 42.0 (o), 21.5 (o), 17.6 (o); IR (neat): $\tilde{\nu}$ = 3087 (w), 2996 (w), 2918 (w), 1590 (m), 1568 (s), 1494 (w), 1481 (w), 1454 (w), 1420 (m), 1371 (s), 1357 (s), 1296 (m), 1283 (m), 1268 (m), 1178 (s), 1163 (s), 1128 (s), 1086 (s), 1030 (s), 2703 (m), 680 (s), 667 (s) cm^{-1}; HRMS (ESI): *m/z* calcd for $C_{21}H_{24}NO_4S_2+H^+$: 418.1141 $[M+H]^+$; found: 418.1148; elemental analysis calcd (%) for $C_{17}H_{20}O_3S$: C 60.41, H 5.55, N 3.35, S 15.36; found: C 60.32, H 5.64, N 3.25, S 15.36.

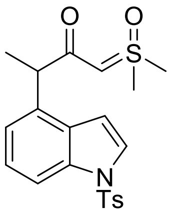

4l

Compound S-8. Carboxylic acid **S-6** (645 mg, 1.88 mmol, 1 equiv) was dissolved in MeOH (59 mL, 0.032 M) and a 5M aq solution of KOH (6 mL, 0.32 M) was added. The reaction was heated at reflux for 20 h, and then cooled to rt, and the MeOH removed *under vacuum*. The residue was diluted with H_2O (5 mL) the acidified to pH 1 with conc. HCl. During the addition, a solid precipitated, which was filtered off, the filter cake washed with 2N HCl, and the solid was dried in a vacuum desiccator overnight to afford an off-white solid (313 mg, 1.65 mmol) that was used without further purification by diluting it in THF (14 mL) at 0 °C under N_2. MeI (1 mL, 16.5 mmol, 10 equiv) was added, followed by NaH (60% in oil, 158 mg, 6.60 mmol, 4 equiv) is small portions. After stirring at r.t. for 3 h, reaction was quenched with H_2O (20 mL) and washed with EtOAc (3 × 15 mL). The aqueous layer was acidified to pH 2-3 with 2N HCl, and then extracted with CH_2Cl_2 (3 × 20 mL). The combined organic layers were dried over $MgSO_4$, filtered and concentrated under vacuum to afford compound a pink solid (322 mg, 1.58 mmol) that was used without further purification according to representative procedure B to afford **S-8** as pale green amorphous solid (365 mg, 1.12 mmol, 71%). ^{1}H NMR (500 MHz, $CDCl_3$): δ 8.18 (d, *J* = 9.2 Hz, 2H), 7.31 (d, *J* = 8.1 Hz, 1H), 7.28-7.25 (m, 1H), 7.16-7.10 (m, 4H), 6.63 (d, *J* = 3.0 Hz, 1H), 4.38 (q, *J* = 7.2 Hz, 1H), 3.83 (s, 3H), 1.73 (d, *J* = 7.2 Hz, 3H); ^{13}C NMR (125 MHz, $CDCl_3$): δ 172.5 (e), 155.8 (e), 137.0 (e), 131.4 (e), 129.1 (o), 126.9 (e), 125.0 (o, 2C), 122.4 (o, 2C), 121.9 (o), 117.8 (o), 108.8 (o), 98.9 (o), 43.7 (o), 33.0 (o), 17.5 (o); IR (neat): $\tilde{\nu}$ = 3081 (w), 2930 (w), 2854 (w), 2114 (w), 1756 (s), 1614 (w), 1592 (m), 1521 (s), 1489 (m), 1445 (w), 1419 (w), 1344 (s), 1304 (m), 1275 (w), 1240 (w), 1206 (s), 1156 (s), 1132 (s), 1073 (s), 1048 (m), 1012 (m), 996 (w), 934 (w), 892 (m), 863 (m), 841 (w), 748 (s), 710 (m), 679 (w) cm^{-1}; HRMS (ESI): *m/z* calcd for $C_{18}H_{16}N_2O_4+H^+$: 325.1183 $[M+H]^+$; found: 325.1182.

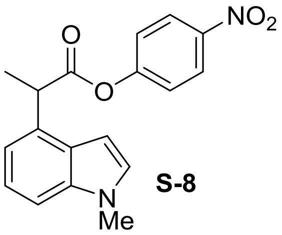

S-8

Compound 4m. Synthesised according to the previously described method[1] from 4-nitrophenyl ester **S-8** (365 mg, 1.12 mmol) and a purification by flash chromatography (CH_2Cl_2/MeOH = 98:2) followed by trituration in Et_2O of the solid obtained after concentration of the product-containing fractions to afford compound **4m** as cream-coloured powder (199 mg, 0.72 mmol, 64%). m.p.: 119–121 °C; ^{1}H NMR (500 MHz, $CDCl_3$): δ 7.22-7.16 (m, 2H), 7.06 (dd, *J* = 6.0, 2.0 Hz, 1H), 7.03 (d, *J* = 3.1 Hz, 1H), 6.58 (d, *J* = 3.2 Hz, 1H), 4.24 (s, 1H), 3.96 (q, *J* = 7.3 Hz, 1H), 3.78 (s, 3H), 3.33 (s, 3H), 3.27 (s, 3H), 1.56 (d, *J* = 7.2 Hz, 3H); ^{13}C NMR (125 MHz, $CDCl_3$): δ 136.7 (e), 135.6 (e), 128.3 (o), 127.5 (e), 121.7 (o), 117.4 (o), 107.5 (o), 99.5 (o), 68.5 (o), 48.1 (o), 42.2 (o), 42.0 (o), 32.9 (o), 17.4 (o); IR (neat): $\tilde{\nu}$ = 3090 (w), 3072 (w), 3006 (w), 2994 (w), 2693 (w), 2914 (w), 2861 (w), 1713 (w), 1679 (w), 1562 (s), 1515 (w), 1492 (s), 1444 (m), 1424 (m), 1416 (m), 1376 (s), 1360 (m), 1339 (m), 1309 (m), 1291 (m), 1278 (m), 1258 (w), 1240 (w), 1180 (s), 1168 (s), 1128 (m), 1082 (s), 1062 (w), 1032 (s), 996 (m), 948 (m), 906 (w), 882 (w), 856 (s), 804 (w), 781 (m), 759 (s), 709 (s), 671 (m) cm^{-1}; HRMS (ESI): *m/z* calcd for $C_{15}H_{19}NO_2S+H^+$: 278.1209 $[M+H]^+$; found: 278.1217; elemental analysis calcd (%) for $C_{15}H_{19}NO_2S$: C 64.95, H 6.90, N 5.05, S 11.56; found: C 64.99, H 6.92, N 4.94, S 11.40.

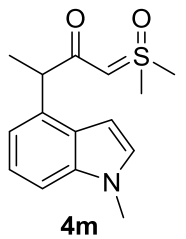
4m

Compound S-9. Synthesised according to representative procedure B from racemic zaltoprofen (2 g, 6.70 mmol) to afford compound **S-9** as a beige amorphous solid (2.44 g, 5.82 mmol, 87%). ^{1}H NMR (500 MHz, $CDCl_3$): δ 8.26-8.18 (m, 3H), 7.67 (d, *J* = 7.9 Hz, 1H), 7.61 (dd, *J* = 8.0, 1.0 Hz, 1H), 7.47 (d, *J* = 2.0 Hz, 1H), 7.44 (td, *J* = 7.4, 1.8 Hz, 1H), 7.36-7.30 (m, 1H), 7.24 (d, *J* = 7.9, 1.9 Hz, 1H), 7.18 (d, *J* = 9.3 Hz, 2H), 4.40 (s, 2H), 3.99 (q, *J* = 7.1 Hz, 1H), 1.62 (d, *J* = 7.2 Hz, 3H); ^{13}C NMR (125 MHz, $CDCl_3$): δ 191.6 (e), 171.5 (e), 155.3 (e), 141.4 (e), 140.0 (e), 138.4 (e), 136.1 (e), 134.0 (e), 132.6 (o), 131.8 (o), 131.5 (o), 130.8 (o), 128.6 (o), 126.9 (o) 126.2 (o), 125.2 (o, 2C), 122.2 (o, 2C), 51.1 (e), 45.3 (o), 18.3 (o); IR (neat): $\tilde{\nu}$ = 3115 (w), 3080 (w), 2980 (w), 2936 (w), 2115 (w), 1758 (s), 1670 (s), 1615 (w), 1589 (m), 1519 (s), 1488 (m), 1458 (m), 1428 (w), 1377 (w), 1344 (s), 1284 (s), 1238 (w), 1202 (s), 1158 (s), 1114 (s), 1068 (s), 1041 (m), 1022 (m), 1011 (m), 993 (w), 924 (w), 892 (w), 863 (s), 845 (m), 832 (w), 811 (w), 755 (s), 729 (m), 695 (w), 677 (w), 661 (w) cm^{-1}; HRMS (ESI): *m/z* calcd for $C_{23}H_{17}NO_5S$ $+Na^+$: 442.0725 $[M+Na]^+$; found: 442.0732

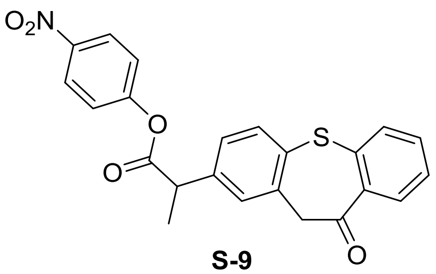
S-9

Compound 4o. Synthesised according to the previously described method[1] from 4-nitrophenyl ester **S-9** (1.11 g, 2.65 mmol) and a purification by flash chromatography (EtOAc/MeOH = 90:10) to afford compound **4n** as amorphous pink gum (444 mg, 1.19 mmol, 45%). ^{1}H NMR (400 MHz, $CDCl_3$): δ 8.19 (dd, *J* = 8.0, 1.4 Hz, 1H), 7.59 (dd, *J* = 8.0, 1.0 Hz, 1H), 7.56 (d, *J* = 8.0 Hz, 1H), 7.42 (ddd, *J* = 7.9, 7.2, 1.6 Hz, 1H), 7.37 (d, *J* = 2.0 Hz, 1H), 7.30 (ddd, *J* = 8.1, 7.2, 1.3 Hz, 1H), 7.16 (dd, *J* = 8.0, 2.0 Hz, 1H), 4.35 (s, 2H), 4.28 (s, 1H), 3.52 (q, *J* = 7.3 Hz, 1H), 3.36 (s, 3H), 3.30 (s, 3H), 1.42 (d, *J* = 7.2 Hz, 3H); ^{13}C NMR (100 MHz, $CDCl_3$): δ 191.6 (e), 190.7 (e), 145.6 (e), 140.5 (e), 137.5 (e), 136.2 (e), 132.4 (o), 132.2 (e), 131.4 (o), 131.2 (o), 130.8 (o), 128.7 (o), 126.7 (o), 126.5 (o), 69.1 (o), 51.1 (e), 49.9 (o), 42.2 (o), 42.1 (o), 18.4 (o); IR (neat): $\tilde{\nu}$ = 3011 (w), 2924 (w), 2552 (w), 1737 (m), 1667 (s), 1563 (s), 1459 (s), 1428 (m), 1411 (m), 1374 (s), 1282 (s), 1238 (m), 1175 (s), 1127 (m), 1074 (w), 1024 (s), 942 (w), 886 (w), 853 (m), 806 (w), 797 (w), 753 (s), 729 (m), 690 (w), 661 (w) cm^{-1}; HRMS (ESI): *m/z* calcd for $C_{20}H_{20}O_3S_2+Na^+$: 395.0752 $[M+Na]^+$; found: 395.0750.

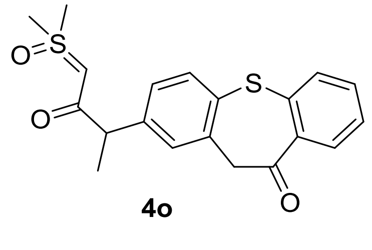
4o

Compound S-10. Synthesised according to method B from 4-trifluoromethylphenylacetic acid (2 g, 9.80 mmol) to afford compound **S-10** as a pale green amorphous solid (1.45 g, 4.46 mmol, 46%). ^{1}H NMR (500 MHz, $CDCl_3$): δ 8.26 (d, *J* = 9.3 Hz, 2H), 7.65 (d, *J* = 8.2 Hz, 2H), 7.50 (d, *J* = 8.2 Hz, 2H), 7.26 (d, *J* = 9.4 Hz, 2H), 3.97 (s, 2H); ^{13}C NMR (100 MHz, $CDCl_3$): δ 168.3 (e), 155.1 (e), 145.5 (e), 136.5 (e), 130.1 (e, q, *J* = 32.5 Hz), 129.8 (o, 2C), 125.8 (o, q, *J* = 4.1 Hz, 2C), 125.2 (o, 2C), 124.0 (e, q, *J* = 272.0 Hz), 122.3 (o, 2C), 40.9 (e); IR (neat): $\tilde{\nu}$ = 3120 (w), 3088 (w), 1759 (w), 1666 (w), 1593 (m), 1519 (s), 1490 (m), 1453 (w), 1421 (w), 1412 (w), 1349 (m), 1337 (s), 1322 (s), 1219 (s), 1207 (m), 1161 (m), 1111 (s), 1063 (s), 1020 (s), 968 (w), 928 (w), 876 (s), 865 (m), 854 (s), 825 (s), 770 (m), 753 (w), 738 (s), 704 (s), 679 (m), 665 (w) cm^{-1}; HRMS (ESI): *m/z* calcd for $C_{15}H_{10}F_3NO_4+Na^+$ $[M+Na]^+$: 348.0460; found 348.0459.

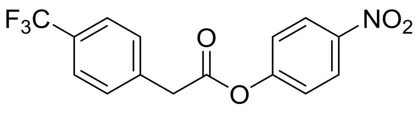

S-10

Compound S-11. Synthesised according to the previously described method[1] from 4-nitrophenyl ester **S-10** (1.32 g, 4.06 mmol) to afford compound **S-11** as white powder, 727 mg, 2.61 mmol, 64%, m.p. = 80-82 °C; ^{1}H NMR (400 MHz, $CDCl_3$): δ 7.55 (d, *J* = 8.0 Hz, 2H), 7.37 (d, *J* = 8.0 Hz, 2H), 4.28 (s, 1H), 3.52 (s, 2H), 3.36 (s, 6H); ^{13}C NMR (125 MHz, $CDCl_3$): δ 186.9 (e), 141.1 (e), 129.6 (o, 2C), 128.6 (q, *J* = 32.6 Hz), 125.2 (o, q, *J* = 3.6 Hz, 2C), 124.3 (e, q, *J* = 272.1 Hz), 70.0 (o), 47.5 (e), 42.1 (o, 2C); IR (neat): $\tilde{\nu}$ = 3088 (w), 3014 (w), 2926 (w), 1614 (w), 1562 (s), 1482 (w), 1417 (m), 1403 (m), 1375 (s), 1315 (s), 1288 (m), 1252 (w), 1205 (w), 1187 (m), 1157 (s), 1113 (s), 1092 (s), 1061 (s), 1026 (s), 1017 (s), 988 (m), 959 (m), 951 (m), 935 (m), 912 (w), 870 (s), 845 (m), 821 (s), 779 (m), 758 (m), 741 (w), 721 (w), 710 (m), 692 (s) cm^{-1}; HRMS (ESI): *m/z* calcd for $C_{12}H_{13}F_3O_2S+H^+$: 279.0661 $[M+H]^+$; found: 279.0657; elemental analysis calcd (%) for $C_{12}H_{13}F_3O_2S$: C 51.79, H 4.71; found: C 51.63, H 4.69.

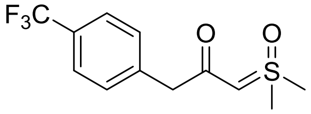

S-11

Compound S-12. Synthesised according to representative procedure B from 4-methoxyphenylacetic acid acid (2 g, 12.03 g) to afford compound **S-12** as a yellow amorphous solid (2.50 g, 8.71 mmol, 73%). ^{1}H NMR (500 MHz, DMSO-d6): δ 8.30 (d, *J* = 9.1 Hz, 2H), 7.43 (d, *J* = 9.1 Hz, 2H), 7.31 (d, *J* = 8.9 Hz, 2H), 6.93 (d, *J* = 8.9 Hz, 2H), 3.95 (s, 2H), 3.75 (s, 3H); ^{13}C NMR (100 MHz, DMSO-d6): δ 172.9 (e), 158.4 (e), 155.4 (e), 135.8 (e), 130.6 (o, 2C), 130.3 (o, 2C), 127.0 (e), 126.2 (o, 2C), 114.0 (o, 2C), 55.0 (o), 40.0 (e); IR (neat): $\tilde{\nu}$ = 3115 (w), 3071 (w), 2990 (w), 2928 (w), 2854 (w), 2115 (m), 1924 (w), 1760 (m), 1708 (w), 1616 (w), 1590 (m), 1519 (s), 1489 (m), 1466 (w), 1454 (m), 1423 (w), 1380 (w), 1343 (s), 1321 (s), 1288 (w), 1261 (w), 1244 (w), 1204 (s), 1164 (w), 1115 (s), 1091 (s), 1063 (s), 1048 (s), 1010 (m), 982 (w), 956 (w), 931 (w), 900 (s), 865 (s), 857 (s), 813 (w), 770 (w), 746 (s), 719 (w), 708 (m), 675 (w) cm^{-1}; HRMS (ESI): *m/z* calcd (%) for $C_{15}H_{13}NO_5+Na^+$: 310.0691 $[M+Na]^+$; found 310.0690.

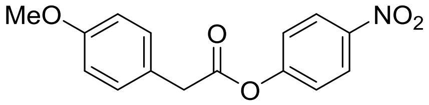

S-12

Compound S-13. Synthesised according to the previously described method[1] from 4-nitrophenyl ester **S-12** (2.4 g, 8.35 mmol) to afford compound **S-13** as yellow powder (1.62 g, 6.76 mmol, 81%). m.p.: 103–106 °C; ^{1}H NMR (400 MHz, $CDCl_3$): δ 7.15 (d, *J* = 8.7 Hz, 2H); 6.83 (d, *J* = 8.7 Hz, 2H); 4.23 (s, 1H); 3.78 (s, 3H); 3.40 (s, 2H); 3.34 (s, 6H); ^{13}C NMR (100 MHz, $CDCl_3$): δ 189.0 (e), 158.2 (e), 130.3 (o, 2C), 129.2 (e), 113.8 (o, 2C), 69.5 (o), 55.2 (o), 47.1 (e), 42.1 (o, 2C); IR (neat): $\tilde{\nu}$ = 3069 (w), 3016 (w), 2964 (w), 2927 (w), 2840 (w), 1610 (w), 1583 (w), 1562 (s), 1509 (s), 1469 (w), 1459 (w), 1443 (w), 1431 (w), 1375 (s), 1329 (w), 1274 (w), 1246 (s), 1186 (w), 1168 (s), 1155 (s), 1117 (m), 1087 (m), 1022 (s), 995 (m), 957 (w), 935 (m), 857 (w), 846 (m), 832 (m), 813 (m), 789 (s), 758 (w), 719 (m), 698 (w), 687 (m) cm^{-1}; HRMS (ESI): *m/z* calcd for $C_{12}H_{16}O_3S+Na^+$: 263.0718 $[M+Na]^+$; found: 263.0727; elemental analysis calcd (%) for $C_{12}H_{16}O_3S$: C 59.98, H 6.71, S 13.34; found: C 59.99, H 6.73, S 13.41.

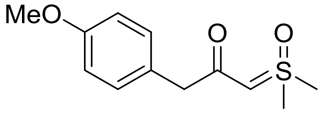

S-13

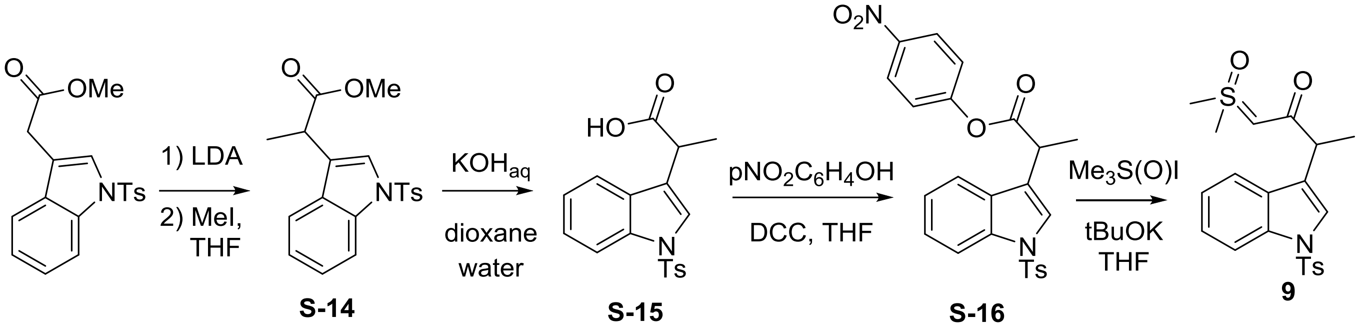

S-14 S-15 S-16 9

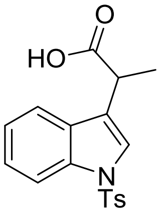

S-15

Compound S-15. Intermediate **S-14** (pale yellow liquid, 474 mg, 1.33 mmol, 30%) was first synthesised from methyl 2-(1-tosyl-1H-indol-3-yl)acetate[3] (1.5 g, 4.37 mmol) and its identity was verified by ^{1}H NMR (500 MHz, $CDCl_3$): δ 7.96 (dt, *J* = 0.9, 8.3 Hz, 1H), 7.76 (d, *J* = 8.4 Hz, 2H), 7.55 (dt, *J* = 8.0, 1.0 Hz, 1H), 7.51 (s, 1H), 7.33-7.28 (m, 1H), 7.24-7.20 (m, 3H), 3.92 (q, *J* = 7.3 Hz, 1H), 3.66 (s, 3H), 2.34 (s, 3H), 1.59 (d, *J* = 7.3 Hz, 3H). Some of this material (345 mg, 0.96 mmol, 1 equiv) was dissolved in 1,4-dioxane/H_2O (2:1, 45 mL, 0.02 M), and a 5% aqueous solution of KOH (3 mL) was added. The reaction was stirred at rt for 4 h, then acidified to pH 2-3 with 2N HCl, and extracted with 3 times with CH_2Cl_2. The combined organic layers were dried over $MgSO_4$, filtered and concentrated under vacuum. The crude residue was purified by flash chromatography on SiO_2 (CH_2Cl_2/MeOH, 98:2) to afford compound **S-15** as an off white solid (330 mg, 0.96 mmol, quant.). m.p. = 108-110 °C; ^{1}H NMR (500 MHz, $CDCl_3$): δ 10.75 (br s, 1H), 7.95 (d, *J* = 8.2 Hz, 1H), 7.75 (d, *J* = 8.1 Hz, 2H), 7.56 (d, *J* = 8.0 Hz, 1H), 7.53 (s, 1H), 7.31 (t, *J* = 7.7 Hz, 1H), 7.24 (d, *J* = 7.7 Hz, 1H), 7.21 (d, *J* = 8.3 Hz, 2H), 3.93 (q, *J* = 7.2 Hz, 1H), 2.33 (s, 3H), 1.61 (d, *J* = 7.1 Hz, 3H); ^{13}C NMR (100 MHz, $CDCl_3$): δ 179.7 (e), 144.9 (e), 135.2 (e), 135.1 (e), 129.9 (o, 2C), 129.6 (e), 126.8 (o, 2C), 124.8 (o), 123.6 (o), 123.2 (o), 120.9 (e), 119.8 (o), 113.7 (o), 36.7 (o), 21.5 (o), 17.0 (o); IR (neat): $\tilde{\nu}$ = 2979 (w), 2941 (w), 2639 (w), 1920 (w), 1744 (w), 1700 (s), 1594 (w), 1562 (w), 1492 (w), 1446 (m), 1420 (w), 1397 (w), 1373 (s), 1304 (m), 1282 (m), 1247 (w), 1233 (w), 1210 (w), 1187 (m), 1172 (s), 1134 (s), 1122 (s), 1108 (m), 1086 (s), 1050 (w), 1016 (m), 997 (m), 959 (m), 865 (w), 811 (m), 794 (w), 760 (m), 749 (s), 729 (m), 701 (m), 665 (s) cm^{-1}; HRMS (ESI neg): *m/z* calcd for $C_{18}H_{17}NO_4S$-H^-: 342.0806 $[M-H]^-$; found: 342.0800.

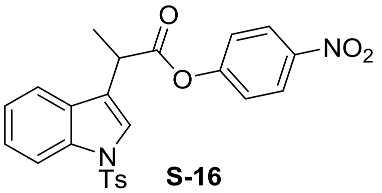

S-16

Compound S-16. Synthesised according to representative procedure B from **S-15** (300 mg, 0.87 mmol) to afford compound **S-16** as a pale green amorphous solid (317 mg, 0.68 mmol, 78%). ^{1}H NMR (500 MHz, $CDCl_3$): δ 8.21 (d, *J* = 9.1 Hz, 2H), 8.01 (dt, *J* = 8.3, 0.3 Hz, 1H), 7.78 (d, *J* = 8.5 Hz, 2H), 7.62 (dt, *J* = 7.9, 0.9 Hz, 1H), 7.61 (s, 1H), 7.39-7.33 (m, 1H), 7.30-7.27 (m, 1H), 7.22 (d, *J* = 8.4 Hz, 2H), 7.11 (d, *J* = 9.2 Hz, 2H), 4.19 (q, *J* = 7.2 Hz, 1H), 2.34 (s, 3H), 1.74 (d, *J* = 7.2 Hz, 3H); ^{13}C NMR (100 MHz, $CDCl_3$): δ 171.3 (e), 155.3 (e), 145.3 (e), 145.1 (e), 135.2 (e), 135.1 (e), 129.9 (o, 2C), 129.3 (e), 126.8 (o, 2C), 125.1 (o, 2C), 123.8 (o), 123.4 (o), 122.2 (o, 2C), 120.5 (e), 119.5 (o), 113.9 (o), 37.1 (o), 21.5 (o), 17.0 (o); IR (neat): $\tilde{\nu}$ = 3115 (w), 2984 (w), 2938 (w), 2116 (w), 1916 (w), 1760 (s), 1615 (w), 1592 (m), 1522 (s), 1489 (m), 1447 (m), 1369 (m), 1346 (s), 1325 (w), 1307 (w), 1283 (w), 1204 (s), 1188 (s), 1173 (s), 1120 (s), 1087 (s), 1074 (s), 1012 (w), 1000 (w), 957 (w), 897 (w), 864 (w), 846 (w), 812 (w), 763 (w), 745 (m), 732 (s), 702 (w) cm^{-1}; HRMS (ESI): *m/z* calcd for $C_{24}H_{20}N_2O_6S+Na^+$: 487.0934 $[M+Na]^+$; found: 487.0936.

[3] Hang, J.; Hongming, L;, Deng, L. *Org. Lett.* **2002**, 4, 3321-3324

Compound 9. Synthesised according to the previously described method[1] from 4-nitrophenyl ester **S-16** (317 mg, 0.68 mmol) and a purification by flash chromatography (CH_2Cl_2/MeOH = 98:2) followed by trituration in Et_2O of the solid obtained after concentration of the product-containing fractions to afford compound **9** as pale green powder (177 mg, 0.42 mmol, 62%). m.p.: 49–51 °C; ^{1}H NMR (500 MHz, $CDCl_3$): δ 7.95 (dt, J = 8.3, 0.8 Hz, 1H), 7.75 (d, J = 8.5 Hz, 2H), 7.55 (dt, J = 8.0, 1.0 Hz, 1H), 7.45 (d, J = 0.8 Hz, 1H), 7.30-7.26 (m, 1H), 7.23-7.16 (m, 3H), 4.23 (s, 1H), 3.66 (q, J = 7.2 Hz, 1H), 3.30 (s, 3H), 3.29 (s, 3H), 2.32 (s, 3H), 1.52 (d, J = 7.2 Hz, 3H); ^{13}C NMR (125 MHz, $CDCl_3$): δ 190.8 (e), 144.8 (e), 135.34 (e), 135.28 (e), 130.4 (e), 129.8 (o, 2C), 126.7 (o, 2C), 124.8 (e), 124.6 (o), 123 (o), 122.9 (o), 120.1 (o), 113.7 (o), 68.4 (o), 42.11 (o), 42.04 (o), 42.01 (o), 21.5 (o), 17.6 (o); IR (neat): $\tilde{\nu}$ = 3015 (w), 2925 (w), 1570 (s), 1494 (w), 1446 (s), 1358 (s), 1304 (m), 1277 (m), 1168 (s), 1120 (s), 1101 (s), 1087 (s), 1020 (s), 998 (m), 961 (m), 899 (w), 854 (m), 812 (m), 764 (w) cm^{-1}; HRMS (ESI): *m/z* calcd for $C_{21}H_{23}NO_4S_2$+H^+: 418.1141 [M+H]$^+$; found: 418.1145.

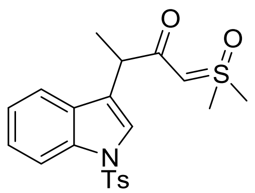

9

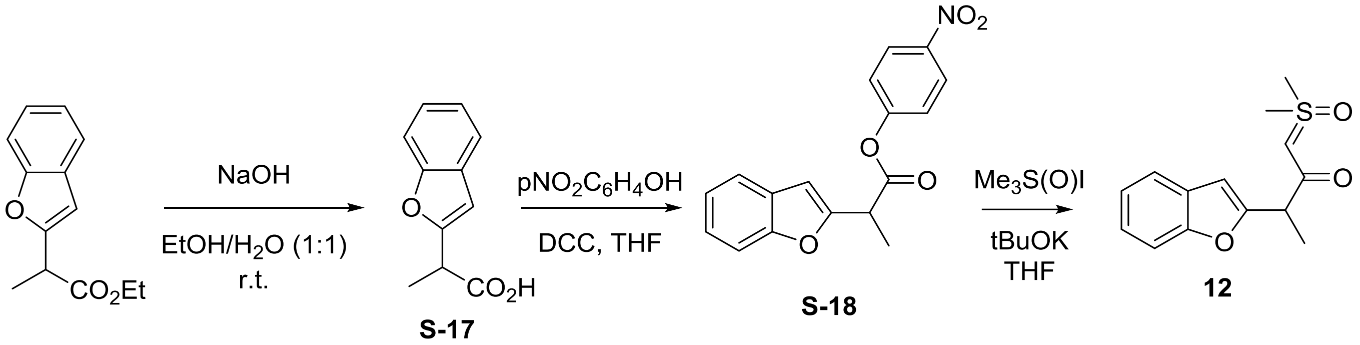

Compound S-17. Ethyl 2-(benzofuran-2-yl)propanoate[4] (1.53 g, 7.01 mmol, 1 equiv) was dissolved in EtOH/H_2O (1:1, 44 mL, 0.16 M) and NaOH (0.56 g, 14.02 mmol, 2 equiv) was added. The reaction was stirred at r.t. for 2.5 h, then carefully acidified to pH 3 using 2N HCl. The reaction was then quickly extracted 3 times with CH_2Cl_2. The combined organic layers were dried over $MgSO_4$, filtered and concentrated under vacuum to afford compound **S-17** as a light brown solid (1.32 g, 6.94 mmol, quant.). m.p. = 58-60 °C; ^{1}H NMR (500 MHz, $CDCl_3$): δ 7.52 (d, J = 7.5 Hz, 1H), 7.45 (d, J = 8.2 Hz, 1H), 7.28-7.23 (m, 1H), 7.20 (t, J = 7.6 Hz, 1H), 6.62 (s, 1H), 4.00 (q, J = 7.2 Hz, 1H), 1.66 (d, J = 7.6 Hz, 3H); ^{13}C NMR (100 MHz, $CDCl_3$): δ 178.0 (e), 155.4 (e), 154.8 (e), 128.0 (e), 124.1 (o), 122.7 (o), 120.8 (o), 111.1 (o), 103.5 (o), 39.7 (o), 15.5 (o); IR (neat): $\tilde{\nu}$ = 2989 (w); 2946 (w); 2889 (w); 2616 (w); 2533 (w); 2050 (w); 1941 (w); 1903 (w); 1790 (w); 1693 (s); 1606 (m); 1588 (w); 1497 (w); 1453 (s); 1412 (m); 1375 (m); 1348 (w); 1327 (w); 1303 (w); 1286 (m); 1256 (s); 1230 (m); 1215 (s); 1171 (s); 1154 (m); 1110 (w); 1070 (m); 1006 (w); 932 (s); 882 (m); 859 (w); 816 (m); 741 (s); 731 (s); 670 (s) cm^{-1}; HRMS (ESI neg): *m/z* calcd for $C_{11}H_{10}O_3$-H^-: 189.0557 [M-H]$^-$; found: 189.0556 [M-H]$^-$.

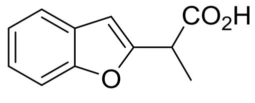

S-17

[4] Heunissen, C.; Wang, J.; Evano, G. *Chem. Sci.* **2017**, 8, 3465-3470

Compound S-18. Synthesised according to representative procedure B from **S-17** (1.52 g, 7.99 mmol) to afford compound **S-18** as viscous yellow oil (1.42 g, 4.56 mmol, 57%). ^{1}H NMR (500 MHz, $CDCl_3$): δ 8.26 (d, *J* = 8.8 Hz, 2H), 7.57 (d, *J* = 8.0 Hz, 1H), 7.49 (d, *J* = 8.0 Hz, 1H), 7.32-7.22 (m, 4H), 6.70 (s, 1H), 4.24 (q, *J* = 7.3 Hz, 1H), 1.78 (d, *J* = 7.3 Hz, 3H); ^{13}C NMR (100 MHz, $CDCl_3$): δ 170.0 (e), 155.3 (e), 154.86 (e), 154.81 (e), 145.4 (e), 128.1 (e), 125.2 (o, 2C), 124.3 (o), 122.9 (o), 122.3 (o, 2C), 120.9 (o), 111.1 (o), 103.8 (o), 40.1 (o), 15.6 (o); IR (neat): $\tilde{\nu}$ = 3116 (w), 3096 (w), 2990 (w), 2936 (w), 2856 (w), 2116 (w), 1810 (w), 1763 (w), 1615 (m), 1592 (m), 1521 (s), 1489 (m), 1454 (s), 1379 (w), 1345 (s), 1254 (w), 1201 (s), 1154 (s), 1120 (s), 1063 (s), 1011 (m), 993 (w), 939 (w), 897 (m), 888 (w), 864 (s), 847 (w), 807 (w), 779 (w), 748 (s), 710 (w), 679 (w) cm^{-1}; HRMS (ESI): calc for $C_{17}H_{13}NO_5+H^+$: 312.0827 $[M+H]^+$; found: 312.0830.

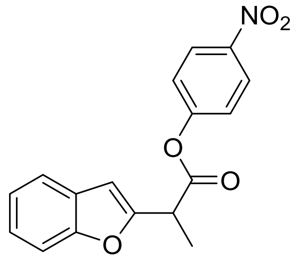

S-18

Compound 12. Synthesised according to the previously described method[1] from 4-nitrophenyl ester **S-18** (1.42 g, 4.56 mmol) and a purification by flash chromatography (CH_2Cl_2/MeOH = 98:2) followed by trituration in Et_2O of the solid obtained after concentration of the product-containing fractions to afford compound **12** as white solid (638 mg, 2.41 mmol, 53%). m.p.: 70–74 °C; ^{1}H NMR (500 MHz, $CDCl_3$): δ 7.50 (d, *J* = 7.8 Hz, 1H), 7.43 (d, *J* = 7.8 Hz, 1H), 7.24-7.16 (m, 2H), 6.54 (s, 1H), 4.40 (s, 1H), 3.72 (q, *J* = 7.2 Hz, 1H), 3.38 (s, 3H), 3.36 (s, 3H), 1.56 (d, *J* = 7.2 Hz, 3H); ^{13}C NMR (125 MHz, $CDCl_3$): δ 188.5 (e), 159.4 (e), 154.6 (e), 128.7 (e), 123.4 (o), 122.4 (o), 120.5 (o), 111 (o), 102.5 (o), 68.7 (o), 44.8 (o), 42.11 (o), 42.08 (o), 16.1 (o); IR (neat): $\tilde{\nu}$ = 3093 (w), 3015 (w), 3000 (w), 2980 (w), 2914 (w), 1561 (s), 1472 (w), 1455 (s), 1384 (s), 1366 (s), 1327 (w), 1310 (w), 1299 (w), 1255 (m), 1179 (s), 1141 (m), 1106 (w), 1053 (w), 1027 (s), 994 (s), 957 (w), 937 (m), 883 (w), 852 (s), 816 (w), 803 (s), 740 (s), 708 (w), 693 (w), 657 (m) cm^{-1}; HRMS (ESI): *m/z* calcd for $C_{14}H_{16}O_3S+Na^+$ $[M+Na]^+$: 287.0718; found: 287.0720.

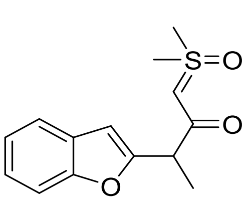

12

Compound S-19. Synthesised according to representative procedure B from 2-(1H-pyrrol-1-yl)propanoic acid[5] (1.52 g, 7.99 mmol) to afford compound **S-18** as viscous yellow oil (1.42 g, 4.56 mmol, 57%). ^{1}H NMR (500 MHz, $CDCl_3$): δ 8.26 (d, *J* = 9.1 Hz, 2H), 7.24 (d, *J* = 9.1 Hz, 2H), 6.84 (t, *J* = 2.1 Hz, 2H), 6.25 (t, *J* = 2.1 Hz, 2H), 5.04 (q, J = 7.3 Hz, 1H), 1.89 (d, *J* = 7.3 Hz, 3H); ^{13}C NMR (100 MHz, $CDCl_3$): δ 168.9 (e), 155.0 (e), 153.5 (e), 125.3 (o, 2C), 122.2 (o, 2C), 119.7 (o, 2C), 109.3 (o, 2C), 57.0 (o), 18.0 (o); IR (neat): 3116 (w), 2989 (w), 2922 (w), 2865 (w), 1764 (s), 1702 (m), 1662 (s), 1615 (w), 1591 (m), 1521 (s), 1489 (s), 1453 (w), 1410 (w), 1346 (s), 1302 (m), 1282 (w), 1248 (w), 1228 (w), 1200 (s), 1159 (s), 1107 (w), 1091 (s), 1064 (m), 1050 (m), 1011 (m), 961 (w), 946 (w), 911 (w), 895 (w), 864 (m), 849 (w), 835 (w), 812 (w), 789 (w), 748 (w), 720 (s), 702 (s), 677 (w); HRMS (ESI): *m/z* calcd for $C_{13}H_{12}N_2O_4+Na^+$: 283.0695 $[M+Na]^+$; found: 283.0692.

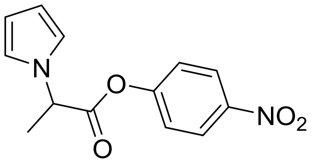

S-19

Compound 14. Synthesised according to the previously described method[1] from 4-nitrophenyl ester **S-19** (2.49 g, 9.56 mmol) and a purification by flash chromatography (CH_2Cl_2/MeOH = 98:2) followed by trituration in Et_2O of the solid obtained after concentration of the product-containing fractions to afford compound **14** as off-white solid (1.66 g, 7.79 mmol, 81%). m.p.: 86–87 °C; ^{1}H NMR (500 MHz, $CDCl_3$): δ 6.74 (t, *J* = 2.1 Hz, 2H), 6.17 (t, *J* = 2.1 Hz, 2H), 4.52 (q, *J* = 7.4 Hz, 1H), 4.02 (s, 1H), 3.36 (s, 3H), 3.35 (s, 3H), 1.68 (d, *J* = 7.3

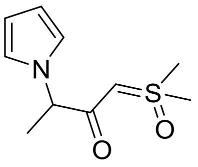

14

[5] Kumar, N. N. B.; Mukhina, O. A.; Kutateladze, A. G. *J. Am. Chem. Soc.* **2013**, 135, 9608-9611

Hz, 3H); ^{13}C NMR (125 MHz, $CDCl_3$): δ 189.2 (e), 119.8 (o, 2C), 108.1 (o, 2C), 68.2 (o), 61.7 (o), 41.9 (o), 41.8 (o), 18.0 (o); IR (neat): $\tilde{\nu}$ = 3099 (w), 2996 (w), 2916 (w), 1537 (s), 1493 (w), 1458 (w), 1435 (w), 1403 (w), 1382 (s), 1363 (m), 1327 (w), 1300 (s), 1282 (m), 1270 (w), 1173 (s), 1090 (m), 1064 (w), 1027 (s), 994 (w), 958 (w), 945 (w), 881 (w), 855 (s), 751 (w), 727 (s), 703 (m), 679 (w) cm^{-1}; HRMS (CI(CH_4)): *m/z* calcd for $C_{10}H_{15}NO_2S$+H^+: 214.0902 [M+H]$^+$; found: 214.0890; elemental analysis calcd (%) for $C_{10}H_{14}NO_2S$: C 56.31, H 7.09, N 6.57, S 15.03; found: C 56.18, H 7.11, N 6.40, S 14.53.

Compound S-20. Synthesised according to representative procedure B from 2-(1H-pyrrol-1-yl)acetic acid (1.89 g, 15.10 mmol) to afford compound **S-20** as pale-brown amorphous solid (1.89 g, 7.68 mmol, 51%). The compound was contaminated by some DCC, and was characterized only by ^{1}H NMR before being used without further purification in the next step. ^{1}H NMR (500 MHz, $CDCl_3$): δ 7.28 (d, *J* = 9.2 Hz, 2H), 7.32 (d, *J* = 9.2 Hz, 2H), 6.75 (t, *J* = 2.0 Hz, 2H), 6.26 (t, *J* = 2.1 Hz, 2H), 4.94 (s, 2H).

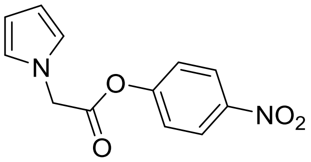
S-20

Compound 17a. Synthesised according to the previously described method[1] from 4-nitrophenyl ester **S-20** (1.00 g, 4.06 mmol) and a purification by flash chromatography (CH_2Cl_2/MeOH = 95:5) to afford compound **17a** as white solid (555 mg, 2.78 mmol, 68%). m.p.: 95–97 °C; ^{1}H NMR (500 MHz, $CDCl_3$): δ 6.65 (t, *J* = 2.0 Hz, 2H), 6.17 (t, *J* = 2.0 Hz, 2H), 4.44 (s, 2H), 4.07 (s, 1H), 3.37 (s, 6H); ^{13}C NMR (125 MHz, $CDCl_3$): δ 186.2 (e), 121.8 (o, 2C), 108.7 (o, 2C), 68.8 (o), 56.9 (e), 42.1 (o, 2C); IR (neat): $\tilde{\nu}$ = 3106 (w), 3068 (w), 3023 (w), 3006 (m), 2921 (w), 2240 (w), 1723 (w), 1548 (s), 1493 (w), 1424 (w), 1403 (w), 1375 (s), 1324 (m), 1308 (m), 1288 (m), 1174 (s), 1164 (s), 1089 (m), 1064 (m), 1028 (s), 991 (m), 974 (m), 964 (m), 916 (m), 859 (m), 816 (w), 762 (m), 726 (s), 701 (s) cm^{-1}; HRMS (ESI): m/z calcd for $C_9H_{13}NO_2S$+H^+: 200.0740 [M+H]$^+$; found: 200.0742.

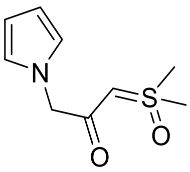
17a

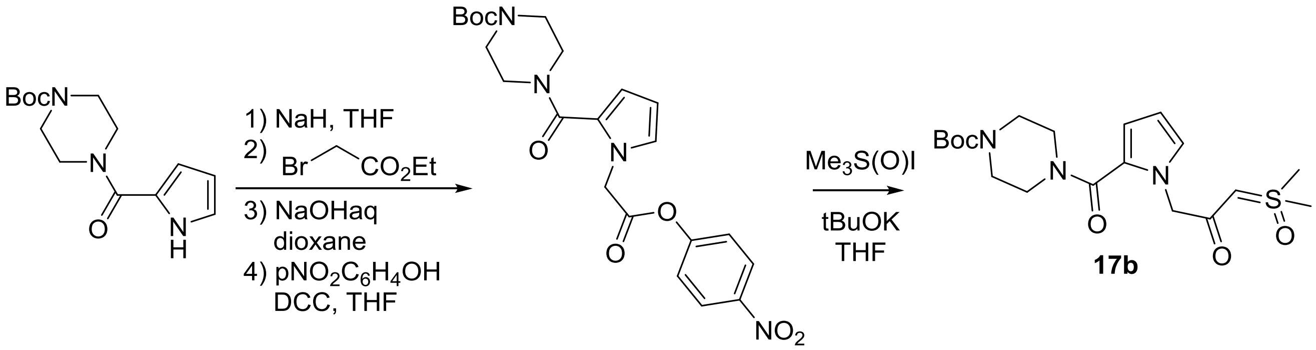

S-21

Compound S-21. NaH (60% in oil, 430 mg, 10.74 mmol, 1.2 equiv) was added in small portion to a solution of *tert*-butyl 4-(1H-pyrrole-2-carbonyl)piperazine-1-carboxylate[#] (2.5 g, 8.95 mmol) in dry DMF (36 mL) at 0 °C under N_2. After stirring at room temperature for 1 h, ethyl 2-bromoacetate (1.1 mL, 9.84 mmol, 1.1 equiv) was added. After stirring at room temperature for 16 h, the reaction was quenched and extracted 3 times with EtOAc. The combined organic layers were washed first with water, then brine, and then dried over $MgSO_4$, filtered and concentrated under vacuo to afford a pale-brown oil (2.62 g, 7.17 mmol, 80%) that was dissolved in 1,4-dioxane (118 mL) before a 5% aqueous solution of NaOH (118 mL) was added. After at room temperature for 1 h, the reaction was then acidified to pH 2-3 with 2N HCl, then extracted with CH_2Cl_2 (4 × 100 mL). The combined organic layers were passed through a phase separator cartridge, and the filtrate was concentrated *under vacuum* to afford a brown gum (2.39 g, 7.09 mmol, quant.) that was then used without further purification according to

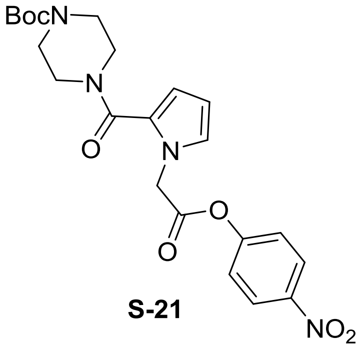
S-21

representative procedure B to afford **S-21** as pale-brown amorphous solid (1.54 g, 3.36 mmol, 47%). ^{1}H NMR (500 MHz, CDCl$_3$): δ 8.29-8.24 (m, 2H), 7.93-7.32 (m, 2H), 6.84 (dd, *J* = 2.5, 1.6 Hz, 1H), 6.47 (dd, *J* = 3.8, 1.5 Hz, 1H), 6.25-6.19 (m, 1H), 5.16 (s, 2H), 3.80–3.60 (m, 4H), 3.49-3.43 (m, 4H), 1.47 (s, 9H); ^{13}C NMR (125 MHz, CDCl$_3$): δ 166.6 (e), 162.7 (e), 154.9 (e), 154.3 (e), 145.2 (e), 126.6 (o), 125.0 (o, 2C), 124.0 (e), 122.0 (o, 2C), 114.0 (o), 107.8 (o), 80.0 (e), 50.1 (e), 28.1 (o, 3C) (Two resonances of methylene carbon nuclei of piperazine are not visible due to slow rotation of Boc group); HRMS (ESI): *m/z* calcd for $C_{22}H_{26}N_4O_7$+Na$^+$: 481.1694 [M+Na]$^+$; found: 481.1710.

Compound 17b. Synthesised according to the previously described method[1] from 4-nitrophenyl ester **S-21** (770 mg, 1.68 mmol) and a purification on a BIOTAGE flash chromatography system (gradient 0–30% MeOH in EtOAc) to afford compound **17b** as white solid (538 mg, 1.31 mmol, 78%). m.p.: 125–127 °C; ^{1}H NMR (500 MHz, CDCl$_3$): δ 6.75 (dd, *J* = 2.5, 1.7 Hz, 1H), 6.33 (dd, *J* = 3.8, 1.6 Hz, 1H), 6.14 (dd, *J* = 3.7, 2.7 Hz, 1H), 4.73 (s, 2H), 4.13 (s, 1H), 3.74–3.67 (m, 4H), 3.42–3.50 (m, 4H), 3.36 (s, 6H), 1.47 (s, 9H); ^{13}C NMR (125 MHz, CDCl$_3$): δ 185.0 (e), 163.2 (e), 154.6 (e), 126.5 (o), 125.0 (e), 112.9 (o), 107.4 (o), 80.2 (e), 67.9 (o), 55.0 (e), 42.2 (o. 2C), 28.3 (o, 3C) (Two signals of methylene carbon nuclei of piperazine are not visible due to slow rotation of Boc group); IR (neat): $\tilde{\nu}$ = 3082 (w), 2975 (w), 2917 (w), 2867 (w), 1674 (s), 1624 (s), 1561 (s), 1470 (m), 1455 (m), 1418 (s), 1378 (s), 1325 (m), 1305 (m), 1280 (m), 1242 (s), 1183 (s), 1164 (s), 1138 (m), 1123 (s), 1072 (m), 1055 (w), 1022 (m), 997 (m), 970 (w), 954 (w), 917 (w), 856 (m), 842 (w), 826 (w), 806 (w), 784 (w), 770 (w), 736 (m), 706 (w), 691 (w), 672 (w), 657 (w) cm^{-1}; HRMS (ESI): *m/z* calcd for $C_{19}H_{29}N_3O_5S$+Na$^+$: 434.1720 [M+Na]$^+$; found: 434.1731.

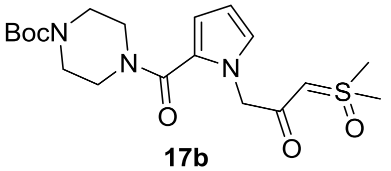

17b

Compound S-22. Synthesised according to representative procedure B from 2-phenyl-2-(1H-pyrrol-1-yl)acetic acid[5] (2.6 g, 12.92 mmol) to afford compound **S-22** as pale-green amorphous solid (2.76 g, 8.56 mmol, 66%). ^{1}H NMR (500 MHz, CDCl$_3$): δ 8.30–8.23 (m, 2H), 7.50–7.39 (m, 5H), 7.31–7.26 (m, 2H), 6.82 (t, *J* = 2.2 Hz, 2H), 6.25 (t, *J* = 2.2 Hz, 2H), 6.12 (s, 1H); ^{13}C NMR (125 MHz, CDCl$_3$): δ 167.3 (e), 154.8 (e), 145.7 (e), 129.4 (o), 129.3 (o, 2C), 127.9 (o, 2C), 125.3 (o, 2C), 122.2 (o, 2C), 120.9 (o, 2C), 109.4 (o, 2C), 65.4 (o); IR (neat): $\tilde{\nu}$ = 2934 (w), 2836 (w), 2162 (w), 1757 (m), 1614 (w), 1591 (m), 1450 (s), 1489 (s), 1463 (m), 1419 (w), 1345 (s), 1205 (s), 1157 (s), 1142 (s), 1115 (s), 1089 (s), 1026 (s), 959 (w), 907 (w), 862 (m), 807 (w), 763 (w), 728 (s), 682 (w) cm^{-1}; HRMS (ESI): *m/z* calcd for $C_{18}H_{14}N_2O_4$+Na$^+$: 345.0851 [M+Na]$^+$; found: 345.0850.

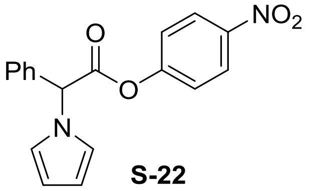

S-22

Compound 17c. Synthesised according to the previously described method[1] from 4-nitrophenyl ester **S-22** (2.76 g, 8.56 mmol) and a purification on a BIOTAGE flash chromatography system (gradient 0–10% MeOH in CH_2Cl_2) to afford compound **17c** as white solid (1.02 g, 3.70 mmol, 43 %). m.p.: 125–126 °C; ^{1}H NMR (500 MHz, CDCl$_3$): δ 7.39-7.28 (m, 5H), 6.69 (t, *J* = 2.1 Hz, 2H), 6.16 (t, *J* = 2.1 Hz, 2H), 5.62 (s, 1H), 4.31 (s, 1H), 3.43 (s, 3H), 3.40 (s, 3H); ^{13}C NMR (125 MHz, CDCl$_3$): δ 186.0 (e), 137.5 (e), 128.6 (o. 2C), 128.5 (o. 2C), 128.1 (o), 121.0 (o. 2C), 108.3 (o. 2C), 70.7 (o), 69.9 (o), 42.2 (o), 42.0 (o); IR (neat): $\tilde{\nu}$ = 3279 (w), 3114 (w), 3075 (w), 3016 (m), 2929 (m), 1698 (w), 1646 (s), 1532 (s), 1525 (m), 1466 (m), 1373 (s), 1325 (s), 1307 (m), 1253 (m), 1243 (m), 1175 (s), 1146 (m), 1086 (s), 1029 (s), 995 (m), 967 (m), 941 (m), 917 (m), 876 (w), 856 (s), 776 (m), 742 (s), 703 (m), 672 (w) cm^{-1}; HRMS (ESI): *m/z* calcd for $C_{15}H_{17}NO_2S$+Na$^+$: 298.0878 [M+Na]$^+$; found: 298.0868.

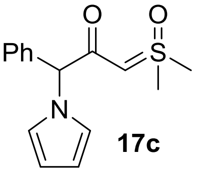

17c

Compound S-23. Synthesised according to representative procedure B from 3-phenyl-2-(1H-pyrrol-1-yl)propanoic acid[5] (2.62 g, 12.17 mmol) to afford compound **S-23** as pale-green amorphous solid (1.55 g, 4.61 mmol, 38%); ^{1}H NMR (500 MHz, $CDCl_3$): δ 8.23 (d, *J* = 2.6 Hz, 2H), 7.34-7.27 (m, 3H), 7.14 (dd, *J* = 7.6, 1.6 Hz, 2H), 7.10-7.05 (m, 2H), 6.14 (t, *J* = 2.1 Hz, 2H), 6.23 (t, *J* = 2.1 Hz, 2H), 5.05 (t, *J* = 7.9 Hz, 1H), 3.55 (dd, *J* = 13.7, 7.7 Hz, 1H, 3.42 (dd, *J* = 13.7, 8.0 Hz, 1H); ^{13}C NMR (125 MHz, $CDCl_3$): δ 167.6 (e), 154.4 (e), 145.4 (e), 135.2 (e), 128.8 (o, 2C), 128.6 (o, 2C), 127.2 (o), 125.0 (o, 2C), 121.9, (o, 2C), 119.8 (o, 2C), 109.1 (o, 2C), 63.2 (o), 39.2 (e); IR (neat): $\tilde{\nu}$ = 3277 (w), 3115, (w), 3085 (w), 3030 (w), 2934 (w), 2857 (w), 1760 (s), 1702 (w), 1667 (m), 1617 (m), 1592 (m), 1520 (s), 1486 (s), 1455 (m), 1446 (m), 1420 (w), 1389 (w), 1344 (s), 1312 (m), 1277 (s), 1243 (m), 1202 (s), 1174 (m), 1157 (m), 1125 (s), 1107 (m), 1093 (s), 1071 (s), 1032 (m), 1024 (m), 1012 (m), 990 (w), 969 (m), 916 (w), 895 (m), 865 (m), 843 (m), 807 (w), 789 (w), 755 (m), 746 (m), 746 (s), 719 (s), 698 (s), 680 (s) cm^{-1}; HRMS (ESI): *m/z* calcd for $C_{19}H_{16}N_2O_4$+H^+: 337.1183 [M+H]$^+$; found: 337.1186.

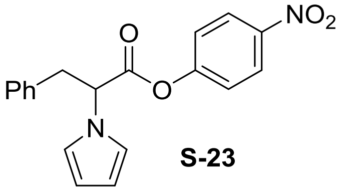

S-23

Compound 17d. Synthesised according to the previously described method[1] from 4-nitrophenyl ester **S-23** (1.45 g, 4.31 mmol) a purification on a BIOTAGE flash chromatography system (gradient 0–10% MeOH in EtOAc) to afford compound **17d** as brown powder (0.78 g, 2.71 mmol, 63%). m.p.: 90–92 °C; ^{1}H NMR (500 MHz, $CDCl_3$): δ 7.23-7.13 (m, 3H), 7.07-7.02 (m, 2H), 6.70 (t, *J* = 2.1 Hz, 2H), 6.12 (t, *J* = 2.1 Hz, 2H), 4.53 (dd, *J* = 9.7, 5.4 Hz, 1H), 4.17 (s, 1H), 3.54 (dd, *J* = 14.1, 5.4 Hz, 1H), 3.35 (s, 3H), 3.31 (s, 3H), 3.16 (dd, *J* = 14.1, 9.7 Hz, 1H); ^{13}C NMR (125 MHz, $CDCl_3$): δ 186.7 (e), 138.2 (e), 128.9 (o. 2C), 128.3 (o. 2C), 126.5 (o), 120.3 (o. 2C), 108.2 (o. 2C), 69.6 (o), 68.1 (o), 42.1 (o), 41.8 (o), 38.5 (e); IR (neat): $\tilde{\nu}$ = 3113 (w), 3075 (w), 3015 (w), 2929 (w), 1698 (w), 1646 (s), 1561 (s), 1525 (m), 1494 (w), 1466 (m), 1374 (s), 1325 (s), 1307 (m), 1253 (m), 1243 (m), 1175 (s), 1147 (m), 1085 (s), 1029 (s), 994 (m), 967 (m), 941 (m), 918 (m), 876 (w), 856 (s), 776 (w), 740 (s), 702 (m), 679 (w) cm^{-1}; HRMS (ESI): *m/z* calcd for $C_{16}H_{19}NO_2S$+Na^+: 312.1029 [M+Na]$^+$; found: 312.1027; elemental analysis calcd (%) for $C_{16}H_{19}NO_2S$: C 66.41, H 6.62, N 4.84, S 11.08; found: C 66.35, H 6.56, N 4.72 S 11.09.

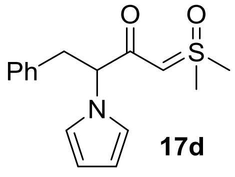

17d

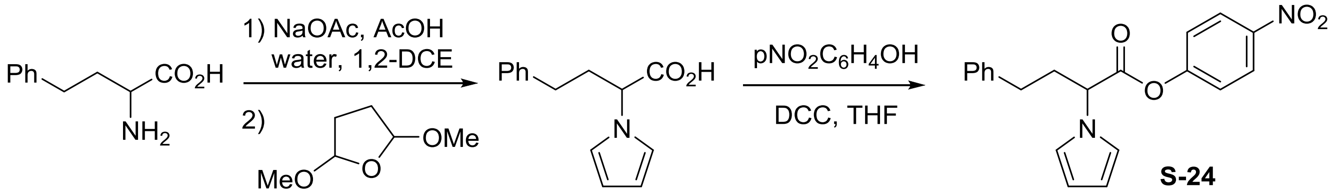

Compound S-24. Homophenylalanine (2 g, 11.16 mmol) and NaOAc (915 mg, 11.16 mmol, 1.0 equiv) were dissolved in AcOH (1.7 mL), water (5 mL), and 1,2-DCE (7 mL). After heating at 90 °C for 5 minutes, 2,5-dimethoxytetrahydrofuran (1.5 mL, 11.16 mmol, 1 equiv) was added, and the reaction was heated at 90 °C for 16 h before being cooled to room temperatured and diluted with ethyl acetate. After washing with brine, and then water water, the combined organic layers were dried over $MgSO_4$, filtered and concentrated under vacuum to afford 4-phenyl-2-(1H-pyrrol-1-yl)butanoic acid (2.25 g, 9.81 mmol, 88%) that was used withour further purification according to representative procedure B to afford compound **S-24** as pale-green amorphous solid (1.94 g, 5.54 mmol, 56%); ^{1}H NMR (500 MHz, $CDCl_3$): δ 8.25 – 8.20 (m, 2H), 7.36 – 7.22 (m, 4H), 7.19 (m, 4H), 6.82 (t, *J* = 2.1, 2H), 6.28 (t, *J* = 2.1, 2H), 4.81 – 4.73 (m, 1H), 2.78 – 2.66 (m, 1H), 2.66 – 2.45 (m, 3H), 2.45 – 2.27 (m, 1H); ^{13}C NMR (125 MHz, $CDCl_3$): δ 168.1 (e), 154.6 (e), 139.3 (e), 128.4 (o, 2C), 128.3 (o, 2C), 128.2 (e), 126.3 (o), 124.9 (o, 2C), 121.9 (o, 2C), 119.8 (o, 2C), 109.1 (o, 2C), 60.4 (o),

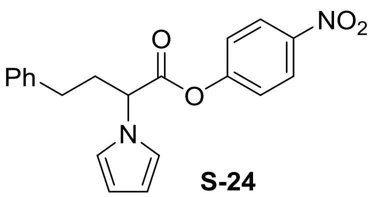

S-24

33.5 (e), 31.3 (e); IR (neat): $\tilde{\nu}$ = 3079 (w), 2934 (w), 2836 (w), 2115 (w), 1754 (s), 1614 (m), 1591 (m), 1516 (s), 1489 (s), 1463 (m), 1419 (w), 1345 (s), 1262 (s), 1237 (s), 1205 (s), 1157 (s), 1142 (s), 1115 (s), 1089 (s), 1026 (m), 959 (w), 907 (w), 862 (m), 808 (w), 763 (w), 728 (s), 682 (w) cm^{-1}; HRMS (CI(CH_4)): *m/z* calcd for $C_{20}H_{18}N_2O_4+H^+$: 351.1339 $[M+H]^+$; found: 351.1337.

Compound 17e. Synthesised according to the previously described method[1] from 4-nitrophenyl ester **S-24**

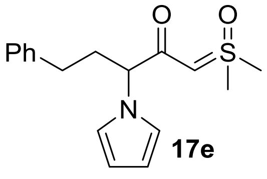
17e

(1.45 g, 4.31 mmol) a purification on a BIOTAGE flash chromatography system (gradient 0–10% MeOH in CH_2Cl_2) to afford compound **17e** as white solid (0.95 g, 3.13 mmol, 56%.). m.p.: 65–68 °C; ^1H NMR (500 MHz, $CDCl_3$): δ 7.31–7.26 (m, 2H), 7.21–7.11 (m, 3H), 6.73 (s, 2H), 6.21 (s, 2H), 4.28 (dd, *J* = 10.9, 4.2 Hz, 1H), 4.09 (s, 1H), 3.33 (s, 6H), 2.67-2.44 (m, 3H), 2.29-2.20 (m, 1H); ^{13}C NMR (125 MHz, $CDCl_3$): δ 188.2 (e), 140.9 (e), 128.6 (o, 2C), 128.4 (o, 2C), 126.0 (o), 120.1 (o, 2C), 108.3 (o, 2C), 68.7 (o), 65.6 (o), 42.1 (o), 41.9 (o), 33.5 (e), 32.2 (e); IR (neat): $\tilde{\nu}$ = 3076 (w), 3001 (w), 2953 (w), 2919 (w), 2861 (w), 1571 (s), 1510 (w), 1487 (w), 1454 (w), 1435 (w), 1415 (s), 1390 (s), 1350 (w), 1313 (w), 1301 (w), 1277 (w), 1263 (w), 1235 (w), 1183 (s), 1169 (s), 1125 (s), 1089 (s), 1071 (w), 1062 (w), 1050 (w), 1028 (s), 1000 (w), 948 (w), 927 (w), 927 (w), 909 (w), 857 (m), 798 (w), 760 (m), 748 (m), 760 (s), 698 (s), 678 (m) cm^{-1}; HRMS (ESI): *m/z* calcd for $C_{17}H_{21}NO_2S+H^+$: 304.1366 $[M+H]^+$; found: 304.1362.

Compound S-25. Synthesised according to representative procedure B from 2-(3,4-dimethoxyphenyl)-3-

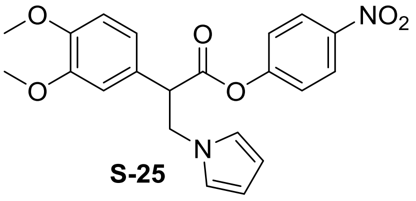
S-25

(1H-pyrrol-1-yl)propanoic acid[5] (2.29 g, 8.32 mmol) to afford compound **S-25** as pale brown solid (1.06 g, 2.67 mmol, 32%). m.p.: 68–70 °C; ^1H NMR (500 MHz, $CDCl_3$): δ 8.22 (d, *J* = 9.2 Hz, 2H), 7.07 (d, *J* = 9.2 Hz, 2H), 6.94 (dd, *J* = 8.3, 2.0 Hz, 1H), 6.89 (d, *J* = 8.2 Hz, 1H), 6.81 (d, *J* = 2.1 Hz, 1H), 6.65 (t, *J* = 2.0 Hz, 2H), 6.16 (t, *J* = 2.1 Hz, 2H), 4.62 (dd, *J* = 13.7, 9.0 Hz, 1H), 4.24 (dd, *J* = 13.8, 5.8 Hz, 1H), 4.19 (dd, *J* = 9.0, 5.8 Hz, 1H), 3.90 (s, 3H), 3.88 (s, 3H); ^{13}C NMR (125 MHz, $CDCl_3$): δ 170.2 (e), 155.1 (e), 149.5 (e), 149.3 (e), 145.5 (e), 127.0 (e), 125.2 (o, 2C); 122.3 (o, 2C); 121.0 (o, 2C), 120.1 (o), 111.6 (o), 110.9 (o), 108.8 (2C, o), 56.0 (o), 55.9 (o), 53.6 (o), 52.4 (e)); IR (neat): $\tilde{\nu}$ = 3120 (w), 3079 (w), 2989 (w), 2953 (w), 2836 (w), 1751 (s), 1619 (m), 1592 (m), 1525 (s), 1513 (s), 1492 (m), 1466 (m), 1453 (m), 1440 (w), 1422 (w), 1347 (s), 1285 (m), 1272 (w), 1249 (m), 1233 (m), 1203 (m), 1182 (w), 1162 (w), 1125 (s), 1088 (s), 1072 (m), 1060 (w), 1037 (w), 1022 (s), 968 (w), 943 (w), 896 (m), 864 (m), 849 (w), 841 (w), 819 (w), 769 (w), 754 (w), 738 (w), 724 (s), 701 (m) cm^{-1}; HRMS (ESI): *m/z* calcd for $C_{21}H_{20}N_2O_6+H^+$: 397.1394 $[M+H]^+$; found: 397.1391; elemental analysis calcd (%) for $C_{21}H_{20}N_2O_6$: C 63.63, H 5.09, N 7.07; found: C 63.51, H 5.26, N 7.24.

Compound 17f. Synthesised according to the previously described method[1] from 4-nitrophenyl ester **S-25**

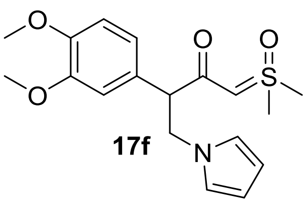
17f

(3.99 g, 10.06 mmol) and a purification by flash chromatography (CH_2Cl_2/MeOH = 98:2) to afford compound **17f** as cream-coloured solid (1.70 g, 4.85 mmol, 48%). m.p.: 78–80 °C; ^1H NMR (500 MHz, $CDCl_3$): δ 6.86–6.77 (m, 3H), 6.57 (t, *J* = 2.0 Hz, 2H), 6.04 (t, *J* = 2.0 Hz, 2H), 4.58 (dd, *J* = 13.6, 8.6 Hz, 1H), 4.28 (s, 1H), 4.08 (dd, *J* = 13.6, 6.0 Hz, 1H), 3.86 (s, 3H), 3.85 (s, 3H), 3.75–3.63 (m, 1H), 3.33 (s, 3H), 3.28 (s, 3H); ^{13}C NMR (100 MHz, $CDCl_3$): δ 187.8 (e), 148.9 (e), 148.2 (e), 131.4 (e), 121.1 (o. 2C), 120.1 (o), 111.1 (o), 111.0 (o), 107.7 (o. 2C), 70.8 (o), 57.6 (o), 55.85 (o), 55.80 (o), 52.3 (e), 41.94 (o), 41.89 (o); IR (neat): $\tilde{\nu}$ = 3113 (w), 3075 (w), 3015 (w), 2929 (w), 1698 (w), 1646 (s), 1561 (s), 1525 (m), 1494 (w), 1466 (m), 1374 (s), 1325 (s), 1307 (m), 1253 (m), 1243 (m), 1175 (s), 1147 (m), 1085 (s), 1029 (s), 994 (m), 967 (m), 941 (m), 918 (m), 876 (w), 856 (s), 776 (w), 740 (s), 702 (m), 679 (w) cm^{-1}; HRMS (ESI): *m/z* calcd for $C_{18}H_{23}NO_4S+Na^+$: 372.1245 $[M+Na]^+$; found: 372.1250.

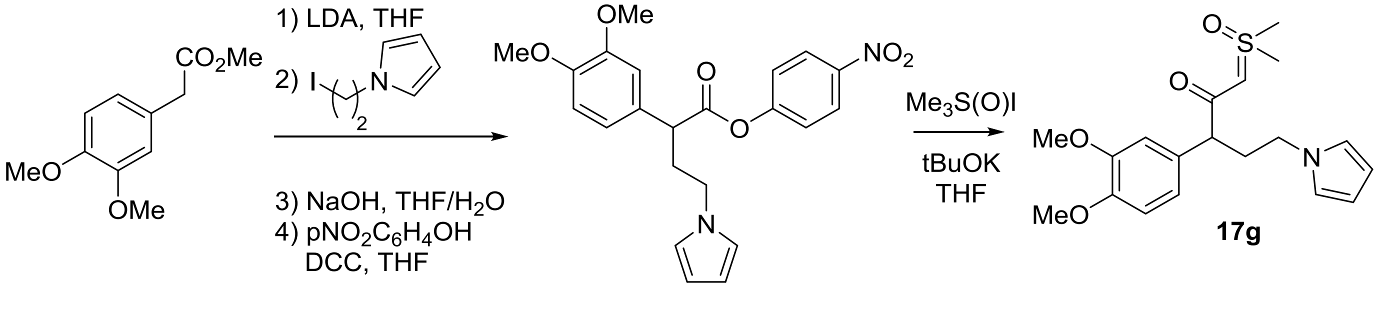

Compound S-26. A flame-dried 2-neck 250 mL flask under N_2 containing $^{i}Pr_2NH$ (6 mL, 42.81 mmol, 1.8 equiv) in THF (25 mL) was cooled to -10 °C using an ice/brine bath before adding $^{n}BuLi$ (2.5 M in hexanes, 16 mL, 39.65 mmol, 1.6 equiv) dropwise. The reaction was then left to stir at -10 °C for 30 minutes before a solution of methyl 2-(3,4-dimethoxyphenyl)acetate (5 g, 24.78 mmol, 1 equiv) in THF (49 mL) was added dropwise. The reaction was left to stir at -10 °C for 30 mins, then 1-(2-iodoethyl)-1H-pyrrole[6] (10.95 g, 49.56 mmol, 2 equiv) was added. The reaction was then left to stir at room temperature for 1 h before being quenched with H_2O, and extracted 3 times with EtOAc. The combined organic layers were washed once with brine, dried over $MgSO_4$, filtered and concentrated under vacuum to afford a pale-yellow oil (2.69 g, 8.87 mmol, 37%) that was dissolved in THF/H_2O (1:1, 35 mL, 0.25 M) before NaOH (1.77 g, 44.35 mmol, 5 equiv) was added. The reaction was stirred at reflux for 15 h, then carefully acidified to pH 2 using 2N HCl. The reaction was then quickly extracted 3 times with CH_2Cl_2. The combined organic layers were dried over $MgSO_4$, filtered and concentrated under vacuum to afford a brown solid (2.55 g, 8.1 mmol, quant.) that was used according to representative procedure B to afford **S-26** as pale-brown amorphous solid (1.81 g, 4.41 mmol, 48%). ^{1}H NMR (500 MHz, $CDCl_3$): δ 8.25 (d, J = 9.3 Hz, 2H), 7.15 (d, J = 9.4 Hz, 2H), 6.97-6.88 (m, 3H), 6.67 (t, J = 2.1 Hz, 2H), 6.22 (t, J = 2.1 Hz, 2H), 4.05-3.98 (m, 1H), 3.93 (s, 3H), 3.92 (s, 3H), 3.91-3.83 (m, 1H), 3.63 (t, J = 7.8 Hz, 1H), 2.73-2.62 (m, 1H), 2.41-2.30 (m, 1H); ^{13}C NMR (125 MHz, $CDCl_3$): δ 171.2 (e), 155.3 (e), 149.4 (e), 148.9 (e), 145.4 (e), 129.0 (e), 125.1 (o, 2C), 122.2 (o, 2C), 120.5 (o, 2C), 120.4 (o), 111.6 (o), 111.0 (o), 108.5 (o, 2C), 56.02 (o), 55.92 (o), 47.9 (o), 46.7 (e), 34.2 (e); IR (neat): $\tilde{\nu}$ = 3080 (w), 2933 (w), 2855 (w), 2115 (w), 1755 (m), 1703 (w), 1615 (w), 1591 (m), 1515 (s), 1500 (m),1489 (m), 1463 (m), 1452 (m), 1420 (w), 1344 (s), 1283 (m), 1261 (s), 1236 (s), 1205 (s), 1157 (m), 1141 (s), 1111 (s), 1089 (m), 1025 (m), 940 (w), 862 (m), 809 (w), 764 (w), 726 (s), 683 (w) cm^{-1}; HRMS (ESI): *m/z* calcd for $C_{22}H_{22}N_2O_6+H^+$: 411.1551 $[M+H]^+$; found: 411.1551.

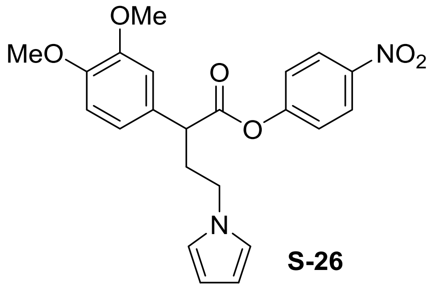

S-26

Compound 17g. Synthesised according to the previously described method[1] from 4-nitrophenyl ester **S-26** (1.81 g, 4.41 mmol) and a purification by flash chromatography (CH_2Cl_2/MeOH = 98:2) to afford compound **17g** as cream-coloured solid (716 mg, 1.97 mmol, 45%). m.p.: 92–93 °C; ^{1}H NMR (500 MHz, $CDCl_3$): δ 6.82–6.78 (m, 3H), 6.62 (t, J = 2.1 Hz, 2H), 6.13 (t, J = 2.1 Hz, 2H), 4.30 (s, 1H), 3.89–3.84 (m, 7H), 3.81–3.74 (m, 1H), 3.36 (s, 3H), 3.29 (s, 3H), 3.19 (t, J = 8.0 Hz, 1H), 2.60–2.50 (m, 1H), 2.18–2.09 (m, 1H); ^{13}C NMR (125 MHz, $CDCl_3$): δ 189.9 (e), 148.9 (e), 147.8 (e), 133.3 (e), 120.6 (o. 2C), 120.1 (o), 111.1 (o), 111.0 (o), 107.8 (o. 2C), 69.8 (o), 55.84 (o), 55.79 (o), 52.5 (o), 47.4 (e), 42.1 (o), 41.9 (o), 34.4

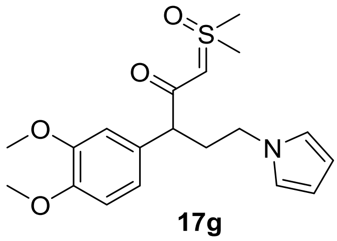

17g

[6] Galeazzi, E.; Guzman, A.; Pinedo, A.; Saldana, A.; Torre, D.; Muchowski, J. M. *Can. J. Chem.* **1983**, *61*, 454–456

(e); IR (neat): $\tilde{\nu}$ = 3016 (w), 2927 (w), 2843 (w), 1606 (w), 1590 (w), 1559 (s), 1515 (s), 1451 (m), 1417 (m), 1360 (s), 1322 (w), 1308 (w), 1289 (w), 1257 (s), 1225 (s), 1172 (s), 1140 (s), 1087 (m), 1065 (w), 1029 (s), 1019 (s), 998 (m), 982 (w), 971 (w), 946 (w), 857 (m), 844 (s), 813 (m), 767 (m), 749 (w), 721 (s), 684 (w) cm^{-1}; HRMS (ESI): *m/z* calcd for $C_{19}H_{25}NO_4S+H^+$: 364.1583 [M+H]$^+$; found: 364.1579; elemental analysis calcd (%) for $C_{19}H_{25}NO_4S$: C 62.79, H 6.93, N 3.85, S 8.82; found: C 62.61, H 6.96, N 3.83, S 8.64.

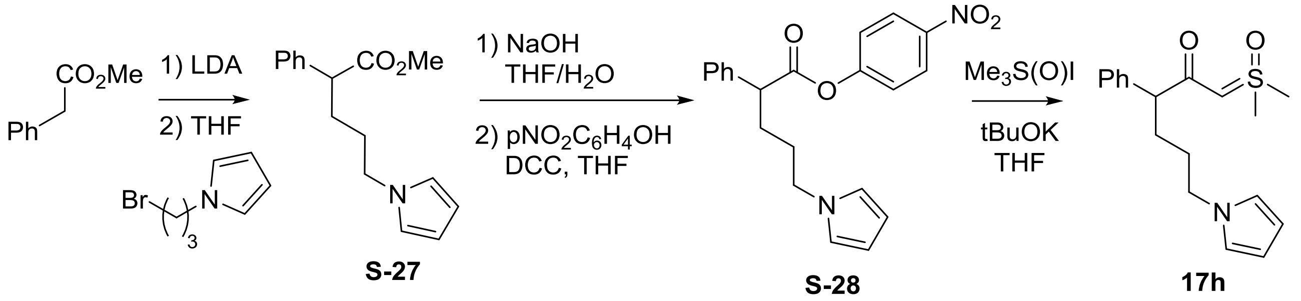

Compound S-27. ^{1}H NMR (500 MHz, $CDCl_3$): δ 7.39-7.20 (m, 5H); 6.60 (t, *J* = 2.2 Hz, 2H); 6.12 (t, *J* = 2.0 Hz, 2H); 3.91-3.80 (m, 2H); 3.65 (s, 3H); 3.50 (t, *J* = 6.6 Hz, 1H); 2.13-2.00 (m, 1H); 1.84-1.62 (m, 3H); ^{13}C NMR (125 MHz, $CDCl_3$): δ 175.8 (e), 141.7 (e), 128.5 (o, 2C), 127.0 (o), 126.3 (o, 2C), 120.4 (o, 2C), 108.1 (o, 2C), 53.3 (e), 52.2 (o), 49.6 (o), 31.8 (e), 26.3 (e); IR (neat): $\tilde{\nu}$ = 3029 (w), 2951 (w), 2872 (w), 1729 (s), 1601 (w), 1584 (w), 1547 (w), 1498 (m), 1453 (m), 1434 (m), 1353 (m), 1280 (m), 1208 (m), 1151 (s), 1088 (s), 1072 (m), 1030 (w), 1005 (w), 969 (w), 919 (w), 821 (w), 721 (s), 697 (s) cm^{-1}; HRMS (ESI): *m/z* calcd for $C_{16}H_{19}NO_2+H^+$: 258.1489 [M+H]$^+$; found: 258.1487.

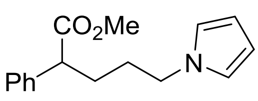

S-27

Compound S-28. ^{1}H NMR (500 MHz, $CDCl_3$): δ 8.22 (d, *J* = 9.1 Hz, 2H), 7.43-7.28 (m, 5H), 7.14 (d, *J* = 9.1 Hz, 2H), 6.63 (t, *J* = 2.1 Hz, 2H), 6.15 (t, *J* = 2.1 Hz, 2H), 3.91 (t, *J* = 6.4 Hz, 2H), 3.72 (t, *J* = 7.2 Hz, 1H), 1.96-1.69 (m, 4H); ^{13}C NMR (100 MHz, $CDCl_3$): δ 171.3 (e), 155.4 (e), 137.4 (e), 131.4 (e), 129.1 (o, 2C), 128.0 (o), 127.9 (o, 2C), 125.2 (o, 2C), 122.3 (o, 2C), 120.5 (o, 2C), 108.3 (o, 2C), 51.3 (o), 49.2 (e), 30.3 (e), 29.4 (e); IR (neat): $\tilde{\nu}$ = 3115 (w), 3071 (w), 2990 (w), 2928 (w), 2854 (w), 2115 (m), 1924 (w), 1760 (m), 1708 (w), 1616 (w), 1590 (m), 1519 (s), 1489 (m), 1466 (w), 1454 (m), 1423 (w), 1380 (w), 1343 (s), 1321 (s), 1288 (w), 1261 (w), 1244 (w), 1204 (s), 1164 (w), 1115 (s), 1091 (s), 1063 (s), 1048 (s), 1010 (m), 982 (w), 956 (w), 931 (w), 900 (s), 865 (s), 857 (s), 813 (w), 770 (w), 746 (s), 719 (w), 708 (m), 675 (w) cm^{-1}; HRMS (ESI): *m/z* calcd for $C_{21}H_{20}N_2O_4+H^+$: 365.1496 [M+H]$^+$; found: 365.1494.

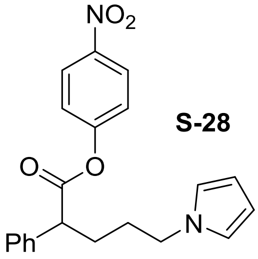

S-28

Compound 17h. Synthesised according to the previously described method[1] from 4-nitrophenyl ester **S-28** (418 mg, 0.98 mmol) and a purification by flash chromatography (CH_2Cl_2/MeOH = 98:2) followed by trituration in Et_2O of the solid obtained after concentration of the product-containing fractions to afford compound **17h** as pale yellow gum (243 mg, 0.64 mmol, 65%). ^{1}H NMR (400 MHz, $CDCl_3$): δ 7.31-7.24 (m, 4H), 7.23-7.18 (m, 1H), 6.61 (t, *J* = 1.9 Hz, 2H), 6.11 (t, *J* = 2.0 Hz, 2H), 4.30 (s, 1H), 3.92-3.77 (m, 2H), 3.35 (s, 3H), 3.32-3.26 (m, 4H), 2.14-2.03 (m, 1H), 1.82-1.62 (m, 3H); ^{13}C NMR (100 MHz, $CDCl_3$): δ 190.2 (e), 141.4 (e), 128.4 (o, 2C), 127.4 (o, 2C), 126.6, 120.4 (o, 2C), 107.8 (o, 2C), 69.7 (o), 56.1 (o), 49.5 (e), 42.1 (o), 41.9 (o), 30.1 (e), 29.9 (e); IR (neat): $\tilde{\nu}$ = 3023 (w), 2926 (w), 2238 (w), 1724 (w), 1562 (s), 1499 (w), 1451 (w), 1375 (s), 1302 (w), 1281 (m), 1176 (s), 1119 (w), 1088 (m), 1069 (w), 1024 (m), 988 (w), 968 (w), 908 (s), 856 (w), 721 (vs), 698 (vs) cm^{-1}; HRMS (ESI): *m/z* calcd for $C_{18}H_{23}NO_2S+H^+$: 318.1522 [M+H]$^+$; found: 318.1524.

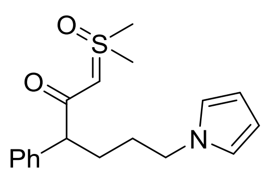

17h

Compound 19. Synthesised according to the previously described method[1] from the corresponding 4-nitrophenyl ester[7] (1.56 g, 4.82 mmol) and a purification by flash chromatography (CH_2Cl_2/MeOH = 98:2) followed by trituration in Et_2O of the solid obtained after concentration of the product-containing fractions to afford compound **19** as white powder (577 mg, 2.07 mmol, 43%). m.p.: 68–70 °C; ^{1}H NMR (500 MHz, $CDCl_3$): δ 7.61 (d, *J* = 7.8 Hz, 1H), 7.28–7.25 (m, 1H), 7.22-7.18 (m, 1H), 7.10-7.06 (m, 1H), 6.86 (s, 1H), 4.37 (s, 1H), 3.73 (s, 3H), 3.35 (s, 6H), 3.08–3.02 (m, 2H), 2.58-2.54 (m, 2H); ^{13}C NMR (125 MHz, $CDCl_3$): δ 190.5 (e), 136.9 (e), 127.8 (e), 126.1 (o), 121.4 (o), 119.0 (o), 118.5 (o), 114.6 (e), 109.0 (o), 69.1 (o), 42.2 (o, 2C), 41.7 (e), 32.5 (o), 21.5 (e); IR (neat): $\tilde{\nu}$ = 3057 (w), 3025 (w), 3002 (w), 2911 (w), 2852 (w), 1614 (w), 1555 (s), 1485 (m), 1472 (m), 1448 (m), 1417 (m), 1373 (s), 1321 (m), 1308 (m), 1280 (w), 1245 (m), 1201 (w), 1165 (s), 1127 (m), 1104 (w), 1060 (w), 1032 (s), 1011 (m), 990 (m), 954 (w), 926 (w), 849 (s), 802 (w), 790 (w), 778 (w), 758 (w), 734 (s), 724 (s), 684 (w) cm^{-1}; HRMS (ESI): *m/z* calcd for $C_{15}H_{19}NO_2S+H^+$: 278.1209 $[M+H]^+$; found: 278.1216; elemental analysis calcd (%) for $C_{15}H_{19}NO_2S$: C 64.95, H 6.90, N 5.05, S 11.56; found: C 64.92, H 6.82, N 5.00, S 11.57.

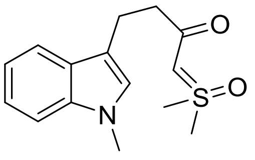

19

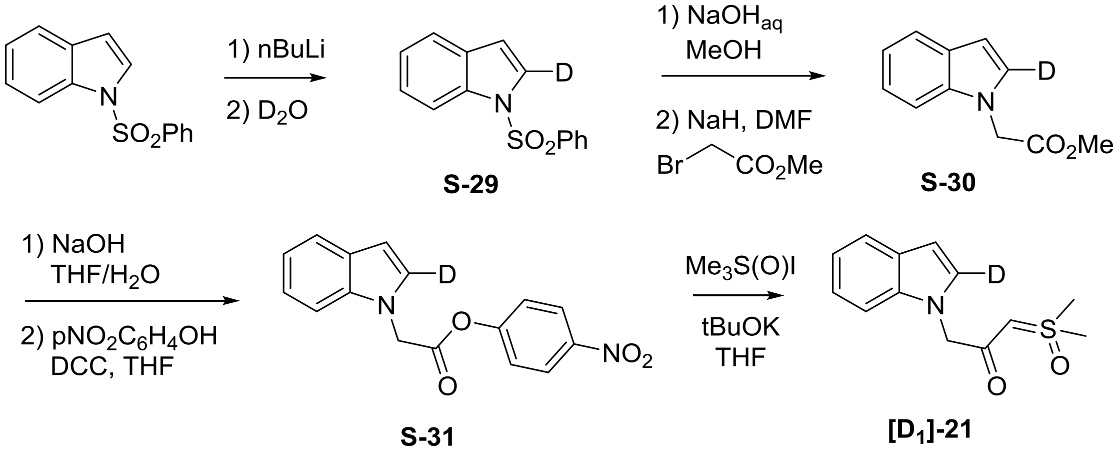

Compound S-30. Compound **S-29**[#8] (2.1 g, 8.17 mmol, 1 equiv) was dissolved in MeOH (11 mL, 0.77 M), and a 2 M aqueous NaOH solution (14 mL) was added. The reaction was heated at reflux for 16 h, then the MeOH was removed under vacuum. The residue was extracted with CH_2Cl_2 (3 × 20 mL), and the combined organic layers were dried over $MgSO_4$, filtered and concentrated under vacuum. The resulting white solid (804 mg, 6.80 mmol, 1 equiv) was used in the next step with no further purification and dissolved in dry DMF (28 mL) under N_2, and cooled to 0 °C. NaH (60% in oil, 326 mg, 8.16 mmol, 1.2 equiv) was added in small portions, and the reaction stirred at room tempearure for 1 h. Methyl bromoacetate (0.7 mL, 7.48 mmol, 1.1 equiv) was added and the reaction was allowed to stir at room tempearure for 16 h. The reaction was quenched with water, and extracted with EtOAc (3 × 100 mL). The combined organic layers were washed with five times water, then once with brine, then dried over $MgSO_4$, filtered, and concentrated under vacuum. The residue was purified by flash column chromatography on SiO_2 (9:1, petroleum ether/Et_2O) to afford compound **S-30** as a colourless oil (641 mg, 3.37 mmol, 50%). ^{1}H NMR (500 MHz, $CDCl_3$): δ 7.64 (dt, *J* = 7.9, 1.0 Hz, 1H), 7.26-7.21 (m, 2H), 7.16-7.12 (m, 1H), 6.57 (s, 1H), 4.87 (s, 2H), 3.74 (s, 3H); ^{13}C NMR (125 MHz, $CDCl_3$): δ 169.1 (e), 136.5 (e), 128.6* (e), 122.1 (o), 121.2 (o), 119.9 (o), 108.9 (o), 102.4 (o), 52.5 (o), 47.7 (e) (the triplet for the ^{13}C nucleus in the deuterated position overlaps with the peak marked

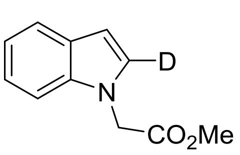

S-30

[7] Hao, L.; Chen, S.; Xu, J.; Tiwari, B.; Zhengqian, L.; Tong, L.; Jieyan, C.; Yonggui, R. *Org. Lett.* **2013**, 15, 4956-4959.

[8] Tiwari, V. K.; Kamal, N.; Kapur, M. *Org. Lett.* **2015**, *17*, 1766–1769.

by an asterisk and is not visible); IR (neat): $\tilde{\nu}$ = 3051 (w), 2952 (w), 1739 (s), 1613 (w), 1496 (w), 1461 (s), 1437 (m), 1421 (m), 1392 (w), 1356 (m), 1329 (m), 1306 (w), 1275 (m), 1211 (s), 1173 (s), 1113 (w), 1063 (w), 1011 (w), 954 (w), 928 (w), 901 (w), 844 (w), 798 (w), 740 (s), 697 (w), 659 (m) cm^{-1}; HRMS (CI(CH_4)): *m/z* calcd for $C_{11}H_{10}DNO_2+H^+$: 191.0925 $[M+H]^+$; found: 191.0931.

Compound S-31. Compound **S-30** (641 mg, 3.37 mmol) was dissolved in THF/H_2O (1:1, 12 mL) before NaOH (404 mg, 16.9 mmol, 5 equiv) was added. The reaction was stirred at reflux for 15 h, then carefully acidified to pH 2 using 2N HCl. The reaction was then quickly extracted 3 times with CH_2Cl_2. The combined organic layers were dried over $MgSO_4$, filtered and concentrated under vacuum to afford a pale-brown solid (559 mg, 3.17 mmol, 94%) that was used without further purification according to representative procedure B to afford **S-31** as yellow amorphous solid (552 mg, 1.86 mmol, 61%). ^1H NMR (500 MHz, $CDCl_3$): δ 8.25 (d, *J* = 9.3 Hz, 2H), 7.68 (dt, *J* = 7.9, 1.0 Hz, 1H), 7.34 (dd, *J* = 8.3, 0.8 Hz, 1H), 7.31-7.26 (m, 3H), 7.21-7.16 (m, 1H), 6.62 (d, *J* = 0.8 Hz, 1H), 5.15 (s, 2H); ^{13}C NMR (125 MHz, $CDCl_3$): δ 166.3 (e), 154.7 (e), 145.6 (e), 136.4 (e), 128.8* (e), 126.2 (o), 125.3 (o, 2C), 122.5 (o), 122.1 (o, 2C), 121.4 (o), 120.3 (o), 115.8 (o), 108.7 (o), 103.1 (o), 47.9 (e) (the triplet for the ^{13}C nucleus in the deuterated position overlaps with the peak marked by an asterisk and is not visible); IR (neat): $\tilde{\nu}$ = 3122 (w), 3076 (w), 3030 (w), 3011 (w), 2988 (w), 2909 (w), 1723 (w), 1608 (w), 1557 (s), 1513 (w), 1483 (w), 1463 (m), 1428 (m), 1385 (s), 1363 (m), 1335 (s), 1319 (s), 1257 (m), 1177 (s), 1152 (s), 1119 (w), 1088 (w), 1041 (s), 1012 (w), 997 (w), 974 (w), 956 (w), 946 (w), 926 (m), 861 (s), 767 (s), 749 (s), 725 (s), 692 (m) cm^{-1}; HRMS (ESI): *m/z* calcd for $C_{16}H_{11}DN_2O_4+H^+$: 298.0933 $[M+H]^+$; found: 298.0929.

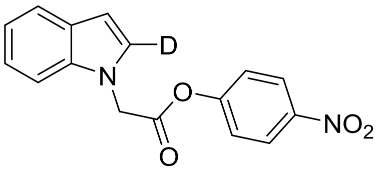

S-31

Compound [D1]-21. Synthesised according to the previously described method[1] from 4-nitrophenyl ester **S-31** (552 mg, 1.86 mmol) and a purification by flash chromatography (CH_2Cl_2/MeOH = 98:2) followed by trituration in Et_2O of the solid obtained after concentration of the product-containing fractions to afford compound **[D1]-21** as cream-coloured solid (288 mg, 1.15 mmol, 62%). m.p.: 96–100 °C; ^1H NMR (500 MHz, $CDCl_3$): δ 7.63 (dt, *J* = 7.9, 0.9 Hz, 1H), 7.31 (dd, *J* = 8.3, 0.9 Hz, 1H), 7.23-7.18 (m, 1H), 7.14-7.09 (m, 1H), 6.54 (d, J = 0.8 Hz, 1H), 4.68 (s, 2H), 3.99 (s, 1H), 3.33 (s, 6H); ^{13}C NMR (125 MHz, $CDCl_3$): δ 185.3 (e), 136.3 (e), 128.5* (e), 121.8 (o), 120.9 (o), 119.6 (o), 109.4 (o), 101.8 (o), 69.2 (o), 53.7 (e), 41.9 (o, 2C) (the triplet for the ^{13}C nucleus in the deuterated position overlaps with the peak marked by an asterisk and is not visible); IR (neat): $\tilde{\nu}$ = 3417 (w), 3115 (w), 3076 (w), 3029 (w), 3011 (w), 2988 (w), 2909 (w), 2324 (w), 1937 (w), 1902 (w), 1769 (w), 1731 (w), 1607 (w), 1557 (s), 1460 (s), 1427 (m), 1384 (s), 1356 (m), 1330 (m), 1315 (m), 1250 (w), 1171 (s), 1149 (s), 1041 (s), 1011 (m), 997 (m), 974 (m), 956 (w), 946 (w), 926 (m), 896 (m), 863 (m), 843 (s), 808 (s), 750 (s), 742 (s), 692 (w), 661 (m) cm^{-1}; HRMS (ESI): *m/z* calcd for $C_{13}H_{14}DNO_2+H^+$: 251.0959 $[M+H]^+$; found: 251.0963.

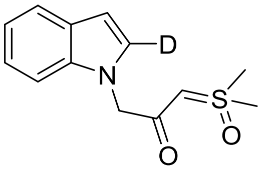

[D1]-21

Compound 21. This compound was prepared analogously. ^1H NMR (500 MHz, $CDCl_3$): δ 7.64 (d, *J* = 7.9 Hz, 1H), 7.31 (dd, *J* = 8.4, 0.8 Hz, 1H), 7.23-7.18 (m, 1H), 7.14–7.09 (m, 2H), 6.55 (dd, *J* = 3.2, 0.9 Hz, 1H), 4.68 (s, 2H), 3.99 (s, 1H), 3.34 (s, 6H); ^{13}C NMR (125 MHz, $CDCl_3$): δ 185.3 (e), 136.4 (e), 128.6 (o), 128.5 (e), 121.8 (o), 120.9 (o), 119.6 (o), 109.4 (o), 102.0 (o), 69.1 (o), 53.7 (e), 41.9 (o, 2C); HRMS (ESI): *m/z* calcd for $C_{13}H_{15}NO_2+H^+$: 250.0896 $[M+H]^+$; found: 250.0899.

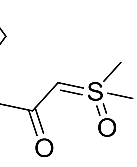

21

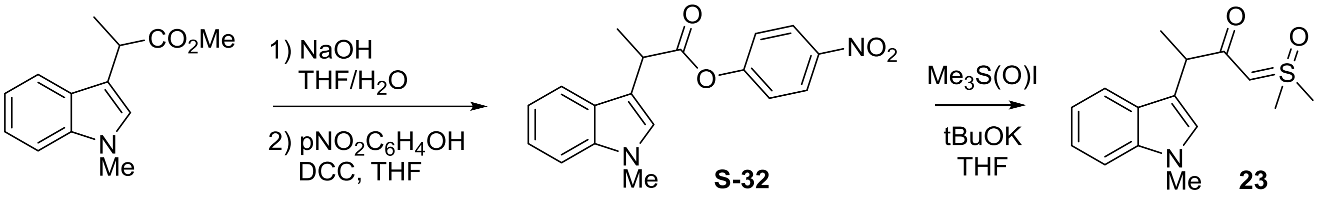

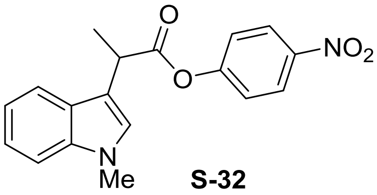

S-32

Compound S-32. Methyl 2-(1-methyl-1H-indol-3-yl)propanoate[9] (3.08 g, 14.18 mmol) was dissolved in THF/H_2O (1:1, 55 mL) before NaOH (2.84 g, 70.9 mmol, 5 equiv) was added. The reaction was stirred at reflux for 15 h, then carefully acidified to pH 2 using 2N HCl. The reaction was then quickly extracted 3 times with CH_2Cl_2. The combined organic layers were dried over $MgSO_4$, filtered and concentrated under vacuum to afford a pale-brown solid (2.87 g, 14.12 mmol, quant.) that was used without further purification according to representative procedure B to afford **S-32** as pale-green amorphous solid (3.96 g, 12.21 mmol, 86%). ^1H NMR (500 MHz, $CDCl_3$): δ 8.20 (d, *J* = 9.3 Hz, 2H), 7.74 (dt, *J* = 8.0, 0.9 Hz, 1H), 7.34 (dt, *J* = 8.3, 1.0 Hz, 1H), 7.31-7.27 (m, 2H), 7.17 (d, *J* = 9.3 Hz, 2H), 7.09 (s, 1H), 4.29 (q, *J* = 7.4 Hz, 1H), 3.80 (s, 3H), 1.74 (d, *J* = 7.2 Hz, 3H); ^{13}C NMR (125 MHz, $CDCl_3$): δ 171.5 (e), 155.9 (e), 145.2 (e), 137.0 (e), 131.5 (e), 129.1 (o), 125.1 (o, 2C), 122.3 (o, 2C), 121.8 (o), 119.0 (o), 112.1 (e), 108.8 (o), 101.2 (o), 45.2 (o), 34.2 (o), 17.5 (o); IR (neat): $\tilde{\nu}$ = 3081 (w), 2934 (w), 2837 (w), 2115 (w), 1760 (m), 1653 (w), 1615 (w), 1591 (m), 1513 (s), 1488 (s), 1463 (m), 1418 (w), 1345 (s), 1259 (s), 1234 (s), 1203 (s), 1158 (s), 1140 (s), 1097 (s), 1024 (s), 962 (w), 930 (w), 913 (w), 913 (w), 884 (w), 862 (s), 806 (w), 782 (w), 758 (m), 722 (s), 682 (w) cm^{-1}; HRMS (ESI): *m/z* calcd for $C_{18}H_{16}N_2O_4+H^+$: 325.1183 $[M+H]^+$; found: 325.1181.

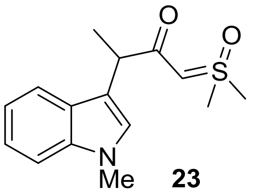

23

Compound 23. Synthesised according to the previously described method[1] from 4-nitrophenyl ester **S-32** (2.35 g, 7.24 mmol) and a purification by flash chromatography (Et2O, then a gradient of 1–2% of MeOH in CH_2Cl_2) followed by trituration in Et_2O of the solid obtained after concentration of the product-containing fractions to afford compound **23** as cream-coloured powder (677 mg, 2.44 mmol, 34%); m.p.: 71-73 °C; ^1H NMR (500 MHz, $CDCl_3$): δ 7.64 (dt, *J* = 7.8, 0.8 Hz, 1H), 7.28 (dt, *J* = 8.4, 1.1 Hz, 1H), 7.23-7.17 (m, 1H), 7.10-7.05 (m, 1H), 6.97 (s, 1H), 4.39 (s, 1H), 3.80 (q, *J* = 7.2 Hz, 1H), 3.76 (s, 3H), 1.54 (d, *J* = 7.2 Hz, 3H); ^{13}C NMR (125 MHz, $CDCl_3$): δ 192.8 (e), 137.0 (e), 127.3 (e), 126.1 (o), 121.4 (o), 119.4 (o), 118.6 (o), 116.6 (e), 109.1 (o), 68.1 (o), 42.11 (o), 42.05 (o), 41.95 (o), 32.6 (o), 18.3 (o); IR (neat): $\tilde{\nu}$ = 3123 (w); 3086 (w); 3047 (w); 3026 (w); 3006 (w); 2984 (w); 2970 (w); 2933 (w); 2909 (w); 2824 (w); 1559 (s); 1544 (m); 1480 (w); 1468 (m); 1446 (w); 1426 (w); 1380 (s); 1367 (m); 1360 (m); 1322 (m); 1303 (m); 1270 (w); 1230 (w); 1209 (w); 1169 (s); 1137 (m); 1114 (m); 1082 (w); 1043 (s); 1008 (m); 994 (w); 977 (w); 944 (w); 924 (w); 887 (w); 854 (m); 832 (w); 801 (w); 758 (w); 741 (s); 702 (m); 671 (m); 641 (w); 627 (w); 572 (w); 558 (w); 539 (w); 519 (w) cm^{-1}; HRMS (ESI): *m/z* calcd for $C_{15}H_{19}NO_2S+H^+$: 278.1209 $[M+H]^+$; found: 278.1216.

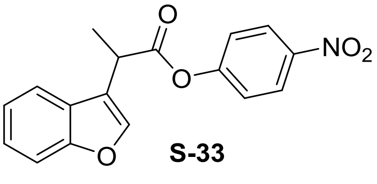

S-33

Compound S-33. Synthesised from ethyl(-1-benzofuran-3-yl)propanoate[10] (1.50 g, 6.97 mmol, 1 equiv) according to the procedure described for the preparation of **S-32** to afford **S-33** as pale-brown amorphous solid (1.38 g, 4.43 mmol, 78%). ^1H NMR (500 MHz, $CDCl_3$): δ 8.22 (d, *J* = 9.0 Hz, 2H), 7.69 (d, *J* = 7.7 Hz, 1H), 7.67 (s, 1H), 7.53 (d, *J* = 8.0 Hz, 1H), 7.38-7.32

[9] Nieman, J. A., Coleman, J. E., Wallace, D. J., Piers, E., Lim, L. Y., Roberge, M., Andersen, R. J. *J. Nat. Prod.* **2003**, 66, 183-199

[10] Yip, S. S. S.; Aïssa, C. *Angew. Chem. Int. Ed.* **2015**, *57*, 6870-6873.

(m, 1H), 7.32-7.27 (m, 1H), 7.19 (d, J = 9.1 Hz, 2H), 4.21 (q, J = 7.4 Hz, 1H), 1.77 (d, J = 7.1 Hz, 3H); ^{13}C NMR (125 MHz, $CDCl_3$): δ 171.5 (e), 155.5 (e), 150.4 (e), 149.8 (e), 136.5 (e), 126.5 (e), 125.2 (o, 2C), 124.5 (o), 122.9 (o), 122.3 (o, 2C), 119.8, 111.9 (o), 36.3 (o), 17.0 (o); IR (neat): $\tilde{\nu}$ = 3115 (w), 3071 (w), 2990 (w), 2928 (w), 2854 (w), 2115 (m), 1924 (w), 1760 (m), 1708 (w), 1616 (w), 1590 (m), 1519 (s), 1489 (m), 1466 (w), 1454 (m), 1423 (w), 1380 (w), 1343 (s), 1321 (s), 1288 (w), 1261 (w), 1244 (w), 1204 (s), 1164 (w), 1115 (s), 1091 (s), 1063 (s), 1048 (s), 1010 (m), 982 (w), 956 (w), 931 (w), 900 (s), 865 (s), 857 (s), 813 (w), 770 (w), 746 (s), 719 (w), 708 (m), 675 (w) cm^{-1}; HRMS (ESI): *m/z* calcd for $C_{17}H_{13}NO_5S$+Na^+: 334.0691 $[M+Na]^+$; found: 334.0695.

Compound 25. Synthesised according to the previously described method[1] from 4-nitrophenyl ester **S-33**

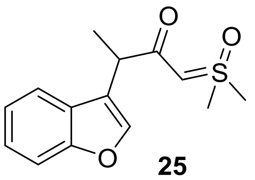

25

(1.38 g, 4.43 mmol) and a purification by flash chromatography (CH_2Cl_2/MeOH = 98:2) followed by trituration in Et_2O of the solid obtained after concentration of the product-containing fractions to afford compound **25** as off-white powder (400 mg, 1.51 mmol, 34%); m.p.: 52-55 °C; ^{1}H NMR (500 MHz, $CDCl_3$): δ 7.62 (d, J = 7.8 Hz, 1H), 7.52 (s, 1H), 7.46 (dt, J = 8.2, 0.8 Hz, 1H), 7.29-7.25 (m, 1H), 7.23-7.18 (m, 1H), 4.39 (s, 1H), 3.69 (q, J = 7.2 Hz, 1H), 3.36 (s, 3H), 3.33 (s, 3H), 1.55 (d, J = 7.2 Hz, 3H); ^{13}C NMR (125 MHz, $CDCl_3$): δ 190.8 (e), 155.4 (e), 141.4 (o), 127.4 (e), 124.1 (o), 122.3 (o), 122.2 (e), 120.2 (o), 111.4 (o), 68.5 (o), 42.11 (o), 42.05 (o), 41.0 (o), 17.5 (o); IR (neat): $\tilde{\nu}$ = 3109 (w); 2994 (w); 2975 (w); 2915 (w); 1615 (w); 1577 (s); 1555 (s); 1471 (w); 1453 (s); 1415 (m); 1404 (m); 1382 (s); 1362 (s); 1329 (m); 1307 (w); 1280 (w); 1250 (w); 1172 (s); 1145 (m); 1120 (w); 1094 (m); 1083 (m); 1061 (m); 1039 (s); 1023 (s); 992 (m); 980 (m); 945 (w); 890 (w); 757 (s); 716 (w); 698 (w); 684 (w) cm^{-1}; HRMS (ESI): *m/z* calcd for $C_{14}H_{16}O_3S$+Na^+: 287.0712 $[M+Na]^+$; found: 287.0718.

Synthesis of compounds 3, 5a–f, 5h–o, 6–8, 10, 11, 13, 15, 26, and 27

Table S1 - Effect of base

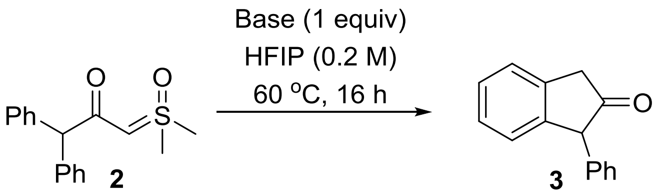

Entry	Base	%Yield[a]
1	NaOAc	77
2	KOPiv	78
3	NaOAc[b]	82
4	KOPiv[b]	79
5	K_2CO_3	91[c]
6	K_3PO_4	77
7	$NaHCO_3$	60
8	NaH	39
9	Et_3N	74
10	DIPEA	79
11	DBU	56
12	DABCO	80
13	Pyridine	38
14	DMAP	42
15	Li_2CO_3	81
16	Na_2CO_3	74
17	Cs_2CO_3	83
18	None	61

Reaction conditions: **1** (0.17 mmol), base (0.17 mmol). [a] Isolated yield. [b] 2 equiv. [c] 200 mg scale

Representative procedure C – Sulfoxonium ylide **2** (189 mg, 0.66 mmol, 1 equiv) and HFIP (3.3 mL) were added to a flame-dried J-Young Schlenk tube under N_2. K_2CO_3 (91 mg, 0.66 mmol, 1 equiv) was then added, the Schlenk was sealed and heated at 60 °C for 16 hours. The mixture is then concentrated under vacuum and the residue was purified by flash column chromatography on SiO_2 (petroleum ether/EtOAc, 98:2) to afford **3** (125 mg, 0.60 mmol, 91%) as purple amorphous solid.

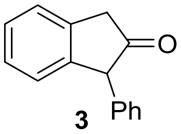

3

Compound 3. ^{1}H NMR (500 MHz, $CDCl_3$): δ 7.40 (d, J = 7.7 Hz, 1H); 7.37-7.26 (m, 5H); 7.20 (d, J = 7.7 Hz, 1H); 7.12 (d, J = 7.0 Hz, 2H); 4.68 (s, 1H); 3.68 (s, 2H). The ^{1}H NMR data is in agreement with that described previously.[11]

Compound 5a. Synthesised according to representative procedure C from compound **4a** (165 mg, 0.66 mmol) to afford compound **5a** as yellow oil (102 mg, 0.59 mmol, 88%). ^{1}H NMR (500 MHz, $CDCl_3$): δ 7.35-7.33 (m, 1H), 7.32-7.27 (m, 3H), 5.76-5.68 (m, 1H), 5.07 (dq, J = 16.9, 1.5 Hz, 1H), 5.02 (d, J = 10.1 Hz, 1H), 3.59-3.53 (m, 2H), 3.46 (d, J = 22.8 Hz, 1H), 2.74-2.69 (m, 1H), 2.63-2.57 (m, 1H). The ^{1}H NMR data is in agreement with that described previously.[12]

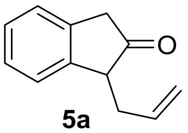

5a

[11] C.-D. Wang, Y.-F. Hsieh, R.-S. Liu, *Adv. Synth. Catal.* **2014**, *356*, 144–152.

Compound 5b. Synthesised according to representative procedure C from **4b** (185 mg, 0.66 mmol), except that the temperature was set at 90 °C, to afford compound **5b** as pale yellow gum (113 mg, 0.56 mmol, 89%). ^{1}H NMR (500 MHz, $CDCl_3$): δ 7.18 (d, *J* = 7.5 Hz, 1H), 7.11-7.05 (m, 2H), 3.58 (d, *J* = 22.5 Hz, 1H), 3.53-3.41 (m, 2H), 2.47 (d, *J* = 7.2 Hz, 2H), 1.87 (sept, *J* = 6.8 Hz, 1H), 1.40 (d, *J* = 7.5 Hz, 3H), 0.91 (d, *J* = 6.6 Hz, 6H); ^{13}C NMR (100 MHz, $CDCl_3$): δ 218.3 (e), 141.0 (e), 140.6 (e), 136.1 (e), 128.4 (o), 125.3 (o), 123.7 (o), 47.5 (o), 45.4 (e), 43.0 (e), 30.3 (o), 22.3 (o, 2C), 15.5 (o); IR (neat): $\tilde{\nu}$ = 3006 (w), 2970 (w), 2958 (w), 2932 (w), 2909 (w), 2867 (w), 2849 (w), 2741 (w), 2633 (w), 1701 (vs), 1671 (vs), 1611 (m): 1567 (m), 1497 (w), 1466 (w), 1428 (m), 1397 (m), 1384 (w), 1350 (s), 1303 (w), 1285 (w), 1257 (s), 1244 (s), 1221 (s), 1192 (w), 1164 (w), 1149 (w), 1116 (w), 1091 (w), 1059 (w), 1018 (w), 982 (w), 957 (w), 930 (m), 889 (m), 843 (m), 788 (m), 731 (w), 711 (w), 685 (m) cm^{-1}; HRMS (CI(CH_4)): *m/z* calcd for $C_{14}H_{18}O+H^+$: 203.1436 $[M+H]^+$; found: 203.1437.

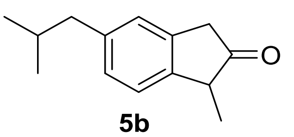
5b

Compound 5c. Synthesised according to representative procedure C from **4c** (168 mg, 0.66 mmol) to afford compound **5c** as pale-yellow oil (75 mg, 0.62 mmol, 65%), besides **6** as colourless liquid (57 mg, 0.16 mmol, 25%). ^{1}H NMR (500 MHz, $CDCl_3$): δ 7.20-7.18 (m, 1H), 6.86-6.83 (m, 2H), 3.82 (s, 3H), 3.58 (d, *J* = 22.3 Hz, 1H), 3.49 (d, *J* = 23.0 Hz, 1H), 3.43 (q, *J* = 7.5 Hz, 1H), 1.38 (d, *J* = 7.4 Hz, 3H); ^{13}C NMR (125 MHz, $CDCl_3$): δ 218.0 (e), 159.1 (e), 137.5 (e), 135.4 (e), 125.0 (o), 113.7 (o), 110.0 (o), 55.4 (o), 47.1 (o), 43.2 (e), 15.8 (o); IR (neat): $\tilde{\nu}$ = 2982 (w), 2968 (w), 2930 (w), 2893 (w), 2871 (w), 2837 (w), 2039 (w), 1899 (w), 1736 (s), 1698 (w), 1609 (m), 1583 (w), 1491 (s), 1467 (m), 1446 (m), 1430 (w), 1389 (w), 1373 (w), 1300 (m), 1271 (s), 1226 (s), 1184 (m), 1167 (w), 1146 (s), 1087 (s), 1046 (s), 1021 (s), 989 (w), 948 (w), 927 (w), 895 (w), 831 (s), 788 (w), 762 (m), 738 (w), 697 (w), 666 (w) cm^{-1}; HRMS (CI(CH_4)): *m/z* calcd for $C_{11}H_{12}O_2+H^+$: 177.0916 $[M+H]^+$; found: 177.0912.

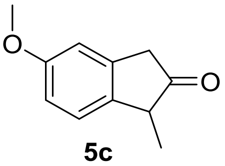
5c

Compound 6. ^{1}H NMR (500 MHz, $CDCl_3$): δ 7.28 (d, *J* = 8.9 Hz, 2H), 6.92 (d, *J* = 8.9 Hz, 2H), 4.34 (sept, *J* = 5.8 Hz, 1H), 3.82 (s, 3H), 2.18 (s, 3H), 1.72 (s, 3H); ^{13}C NMR (125 MHz, $CDCl_3$): δ 206.8 (e), 159.6 (e), 130.2 (e), 127.5 (o, 2C), 127.5 (o, 2C), 114.2 (o, 2C), 88.8 (e), 70.5 (o, hept, *J* = 33.3 Hz), 55.3 (o), 25.2 (o), 21.0 (o) (the resonance for the CF_3 nuclei were not visible); IR (neat): $\tilde{\nu}$ = 2941 (w), 1722 (s), 1610 (m), 1583 (w), 1513 (s), 1465 (w), 1357 (s), 1286 (s), 1256 (s), 1219 (s), 1193 (s), 1101 (s), 1033 (s), 901 (s), 832 (m), 784 (w), 742 (w), 687 (w) cm^{-1}; HRMS (ESI): *m/z* calcd for $C_{14}H_{14}F_6O_3+Na^+$: 367.0739 $[M+Na]^+$; found: 367.0741.

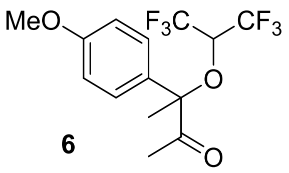

6

Compound 5d. Synthesised according to representative procedure C from **4d** (185 mg, 0.66 mmol), except that the temperature was set at 80 °C and heating was provided by a microwave oven for 1 h, to afford compound **5d** as pale-pink amorphous solid (102 mg, 0.56 mmol, 86%). ^{1}H NMR (500 MHz, $CDCl_3$): δ 7.30 (br s, 1H), 7.29-7.27 (m, 1H), 7.22-7.20 (m, 1H), 3.60 (d, *J* = 22.7 Hz, 1H), 3.50 (d, *J* = 22.8 Hz, 1H), 3.44 (q, *J* = 7.4 Hz, 1H), 1.40 (d, *J* = 7.5 Hz, 3H); ^{13}C NMR (125 MHz, $CDCl_3$): δ 216.5 (e), 141.7 (e), 138.0 (e), 133.0 (e), 127.8 (o), 125.4 (o), 125.0 (o), 47.4 (o), 42.8 (e), 15.4 (o); IR (neat): $\tilde{\nu}$ = 2980 (w), 2934 (w), 2908 (w), 2645 (w), 1742 (s), 1703 (m), 1669 (m), 1600 (w), 1561 (w), 1475 (s), 1450 (m), 1420 (m), 1397 (w), 1384 (w), 1366 (w), 1280 (w), 1245 (w), 1186 (s), 1155 (w), 1111 (w), 1074 (w), 1060 (w), 1038 (w): 983 (w), 960 (w), 936 (w), 903 (m),

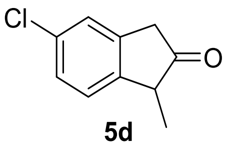

5d

[12] Padwa, A.; Austin, D. J.; Price, A. T.; Semones, M. A.; Doyle, M. P.; Protopopova, M. N.; Winchester, W. R.; Tran, A. *J. Am. Chem. Soc.* **1993**, *115*, 8669.

882 (m), 850 (w), 833 (s), 786 (w), 759 (w), 718 (w), 661 (w), 656 (w) cm^{-1}; HRMS ($CI(CH_4)$): *m/z* calcd for $C_{10}H_9ClO+H^+$: 181.0432 $[M+H]^+$; found 181.0433.

Compound 5e. Synthesised according to representative procedure C from **4e** (200 mg, 0.66 mmol), except that the temperature was set at 90 °C, to afford compound **5e** as yellow powder (121 mg, 0.54 mmol, 82%). m.p. 100–103 °C; ^1H NMR (500 MHz, $CDCl_3$): δ 7.46-7.42 (m, 2H), 7.15 (d, *J* = 8.0 Hz, 1H), 3.60 (d, *J* = 22.6 Hz, 1H), 3.50 (d, *J* = 22.9 Hz, 1H), 3.42 (q, *J* = 7.4 Hz, 1H), 1.40 (d, *J* = 7.5 Hz, 3H); ^{13}C NMR (125 MHz, $CDCl_3$): δ 216.4 (e), 142.3 (e), 138.4 (e), 130.7 (o), 128.0 (o), 125.8 (o), 121.0 (e), 47.4 (o), 42.7 (e), 15.4 (o); IR (neat): $\tilde{\nu}$ = 3011 (w), 2921 (w), 2744 (w), 2639 (w), 2558 (w), 1747 (w), 1701 (s), 1668 (s), 1588 (m), 1555 (m), 1473 (w), 1423 (m), 1412 (m), 1393 (w), 1358 (w), 1341 (m), 1287 (m), 1241 (s), 1099 (m), 1059 (w), 1014 (w), 959 (m), 931 (m), 889 (m), 860 (m), 824 (s), 759 (m), 713 (w), 682 (w) cm^{-1}; HRMS ($CI(CH_4)$): *m/z* calcd for $C_{10}H_9{}^{79}BrO+H^+$: 226.0930 $[M+H]^+$; found 226.0933.

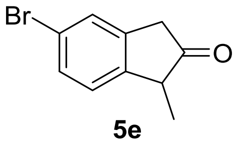

5e

Compound 5f. Synthesised according to representative procedure C from **4f** (193 mg, 0.66 mmol), except that the temperature was set at 90 °C, to afford compound **5f** as colourless liquid (89 mg, 0.42 mmol, 63%). ^1H NMR (500 MHz, $CDCl_3$): δ 7.61–7.55 (m, 2H), 7.40 (d, *J* = 7.9 Hz, 1H), 3.66 (d, *J* = 22.6 Hz, 1H), 3.57 (d, *J* = 22.5 Hz, 1H), 3.53 (q, *J* = 7.3 Hz, 1H), 1.44 (d, *J* = 7.5 Hz, 3H); ^{13}C NMR (125 MHz, $CDCl_3$): δ 216.0 (e), 147.3 (e), 137.1 (e), 129.9 (e, *J* = 32.0 Hz), 124.60 (o, *J* = 3.7 Hz), 124.58 (o), 124.1 (e, *J* = 272 Hz), 121.8 (o, *J* = 3.9 Hz), 47.8 (o), 42.7 (e), 15.2 (o); ^{19}F NMR (376 MHz, $CDCl_3$): δ -62.4 (s, 3F); IR (neat): $\tilde{\nu}$ = 2976 (w), 1753 (s), 1627 (w), 1437 (m), 1372 (w), 1334 (s), 1284 (s), 1243 (m), 1184 (s), 1153 (s), 1121 (s), 1068 (s), 913 (w), 894 (m), 834 (m), 750 (w), 718 (w), 690 (w) cm^{-1}; HRMS ($CI(CH_4)$): *m/z* calcd for $C_{12}H_{14}O_3+H^+$: 207.1016 $[M+H]^+$; found 207.1012

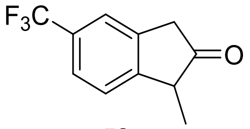

5f

Compound 5h. Synthesised according to representative procedure C from **4h** (188 mg, 0.66 mmol), to afford compound **5h** as yellow powder (110 mg, 0.53 mmol, 81%). m.p.: 100–103 °C; ^1H NMR (500 MHz, $CDCl_3$): δ 6.83 (s, 1H), 6.79 (s, 1H), 3.90 (s, 3H), 3.89 (s, 3H), 3.54 (d, *J* = 22.4 Hz, 1H), 3.50–3.40 (m, 2H), 1.39 (d, *J* = 7.5 Hz, 3H); ^{13}C NMR (125 MHz, $CDCl_3$): δ 218.1 (e), 149.0 (e), 148.8 (e), 135.2 (e), 127.9 (e), 107.8 (o), 107.2 (o), 56.06 (o), 56.04 (o), 47.8 (o), 42.8 (e), 15.9 (o); IR (neat): $\tilde{\nu}$ = 3139 (w), 2977 (w), 2941 (w), 2844 (w), 1723 (s), 1634 (w), 1607 (w), 1594 (w), 1516 (s), 1446 (m), 1420 (w), 1397 (w), 1347 (w), 1332 (w), 1312 (w), 1301 (w), 1277 (m), 1248 (m), 1236 (m), 1224 (s), 1189 (m), 1164 (m), 1143 (s), 1067 (w), 1022 (s), 927 (w), 884 (w), 848 (m), 815 (w), 795 (w), 764 (w), 719 (s) cm^{-1}; HRMS ($CI(CH_4)$): *m/z* calcd for $C_{12}H_{14}O_3+H^+$: 207.1016 $[M+H]^+$; found 207.1012.

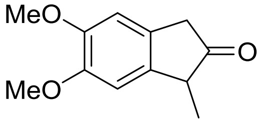

5h

Compound 5i. Synthesised according to representative procedure C from **4i** (230 mg, 0.66 mmol), but heating was maintained for 48 h, to afford compound **5i** as cream-coloured powder (121 mg, 0.45 mmol, 69%). m.p.: 165–167 °C; ^1H NMR (500 MHz, $CDCl_3$): δ 8.02 (d, *J* = 1.8 Hz, 1H), 7.98 (d, *J* = 8.0 Hz, 1H), 7.42 (dd, *J* = 8.7, 2.1 Hz, 1H), 7.29 (d, *J* = 8.7 Hz, 1H), 7.16 (d, *J* = 8.0 Hz, 1H), 4.11-4.01 (m, 2H), 3.98 (s, 3H), 3.63 (q, *J* = 8.1 Hz, 1H), 1.50 (d, *J* = 7.5 Hz, 3H); ^{13}C NMR (125 MHz, $CDCl_3$): δ 217.3 (e), 142.4 (e), 139.3 (e), 137.5 (e), 125.5 (o), 124.6 (e), 123.8 (e), 121.3 (e), 119.9 (o), 119.4 (o), 117.2 (e), 115.3 (o), 109.4 (o), 47.2 (o), 41.5 (e), 30.6 (o), 16.1 (o); IR (neat): $\tilde{\nu}$ = 2932 (w), 1741 (s), 1622 (w), 1600 (w) 1580 (w), 1490 (w), 1459 (s), 1433 (w), 1410 (w), 1372 (w), 1359 (w), 1342 (w), 1305 (m), 1275 (m), 1229 (m), 1189 (m), 1157 (w), 1128 (m), 1102 (w), 1078 (m), 1055 (w), 1038 (w) 1002 (w), 940 (w), 917 (m), 886 (m), 806 (s), 795 (s), 770 (m), 738 (m), 730 (w), 710 (w), 685 (w), 660 (m) cm^{-1}; HRMS ($CI(CH_4)$): *m/z* calcd for $C_{17}H_{14}ClNO+H^+$: 283.0764 $[M+H]^+$; found 284.0833.

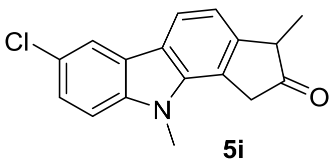

5i

Compound 5j. Synthesised according to representative procedure C from **4j** (200 mg, 0.66 mmol), but heating was maintained for 48 h, to afford compound **5j** as off-white solid (137 mg, 0.61 mmol, 92%). m.p.: 82–85 °C; ^{1}H NMR (500 MHz, $CDCl_3$): δ 7.70 (d, J = 8.2 Hz, 1H), 7.55 (d, J = 8.8 Hz, 1H), 7.36 (d, J = 8.3 Hz, 1H), 7.24-7.14 (m, 2H), 3.93 (s, 3H), 3.72 (s, 2H), 3.64 (q, J = 7.5 Hz, 1H), 1.47 (d, J = 7.5 Hz, 3H); ^{13}C NMR (125 MHz, $CDCl_3$): δ 217.8 (e), 157.5 (e), 138.2 (e), 134.0 (e), 132.5 (e), 126.9 (o), 125.9 (o), 125.2 (e), 122.5 (o), 119.3 (o), 106.9 (o), 55.3 (o), 48.5 (o), 41.4 (e), 15.9 (o); IR (neat): $\tilde{\nu}$ = 3040 (w), 3005 (w), 2977 (w), 2959 (w), 2935 (w), 2898 (w), 2860 (w), 2840 (w), 1733 (s), 1625 (m), 1598 (m), 1516 (w), 1479 (m), 1448 (w), 1428 (m), 1396 (w), 1362 (m), 1317 (w), 1290 (w), 1274 (w), 1262 (m), 1243 (s), 1226 (m), 1194 (m), 1166 (m), 1150 (m), 1135 (m), 1047 (s), 1028 (s), 998 (m), 961 (w), 936 (w), 905 (w), 896 (w), 873 (s), 813 (s), 768 (w), 748 (w), 735 (w), 710 (w), 698 (w), 698 (w), 659 (w) cm^{-1}; HRMS (CI(CH_4)): m/z calcd for $C_{15}H_{14}O_2$+H^+: 227.1072 $[M+H]^+$; found 227.1075.

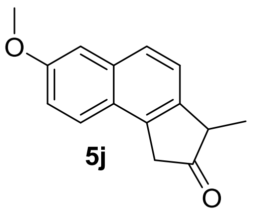

5j

Compound 5k. Synthesised according to representative procedure C from **4k** (198 mg, 0.66 mmol), except that the temperature was set at 90 °C, to afford compound **5k** as yellow oil (94 mg, 0.42 mmol, 67%). ^{1}H NMR (500 MHz, $CDCl_3$): δ 7.25-7.17 (m, 6H); 7.08-7.06 (m, 2H); 6.95 (d, J = 7.2 Hz, 1H); 3.79-3.76 (m, 1H); 3.48 (d, J = 22.5 Hz, 1H); 3.34 (dd, J = 13.7, 4.7 Hz, 1H); 3.26 (d, J = 22.8 Hz, 1H); 2.99 (dd, J = 13.7, 8.1 Hz, 1H); ^{13}C NMR (125 MHz, $CDCl_3$): δ 217.1 (e), 141.3 (e), 138.0 (e), 136.9 (e), 129.4 (o, 2C), 128.2 (o, 2C), 127.5 (o), 127.1 (o), 126.4 (o), 125.1 (o), 124.7 (o), 54.4 (o), 43.2 (e), 37.9 (e); IR (neat): $\tilde{\nu}$ = 3070 (w), 3028 (w), 2922 (w), 1744 (s), 1602 (w), 1533 (w), 1495 (m), 1479 (m), 1454 (m), 1391 (m), 1343 (w), 1308 (w), 1280 (w), 1246 (w), 1234 (w), 1188 (w), 1139 (m), 1074 (m), 1042 (w), 1026 (w), 968 (w), 950 (w), 901 (w), 867 (w), 837 (w), 821 (w), 802 (w), 776 (w), 740 (s), 712 (m), 696 (s) cm^{-1}; HRMS (CI(CH_4)): m/z calcd for $C_{16}H_{14}O$+H^+: 223.1117 $[M+H]^+$; found 223.1120.

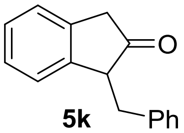

5k

Compound 5l. Synthesised according to representative procedure C from **4l** (40 mg, 0.10 mmol) to afford compound **5l** as off-white solid (21 mg, 0.06 mmol, 64%). m.p.: 143–145 °C; ^{1}H NMR (500 MHz, $CDCl_3$): δ 7.92 (dt, J = 8.6, 0.8 Hz, 1H), 7.78 (d, J = 8.3 Hz, 2H), 7.63 (d, J = 3.7 Hz, 1H), 7.26-7.22 (m, 3H), 6.65 (dd, J = 3.8, 0.9 Hz, 1H), 3.66 (q, J = 7.4 Hz, 1H), 3.62-3.59 (m, 2H), 2.35 (s, 3H), 1.49 (d, J = 7.5 Hz, 3H); ^{13}C NMR (125 MHz, $CDCl_3$): δ 217.7 (e), 145.0 (e), 135.5 (e), 135.2 (e), 134.5 (e), 130.9 (e), 129.9 (o, 2C), 127.1 (o), 126.8 (o, 2C), 120.9 (o), 112.8 (o), 106.4 (o), 47.5 (o), 42.8 (e), 21.6 (o), 15.7 (o); IR (neat): $\tilde{\nu}$ = 3147 (w), 3113 (w), 3061 (w), 3027 (w), 2981 (w), 2940 (w), 2876 (w), 1745 (s), 1654 (w), 1594 (m), 1529 (w), 1489 (w): 1463 (w): 1427 (m), 1363 (s), 1339 (m), 1295 (m), 1275 (m), 1241 (w), 1218 (w), 1178 (s), 1155 (s), 1137 (s), 1118 (s), 1085 (m), 1063 (m), 1023 (m), 1016 (m), 987 (m), 907 (w), 895 (m), 874 (w), 841 (w), 815 (m), 799 (m), 776 (m), 735 (m), 703 (m), 679 (s) cm^{-1}; HRMS (ESI): m/z calcd for $C_{19}H_{17}NO_3S$+Na^+: 362.0821 $[M+Na]^+$; found 362.0821.

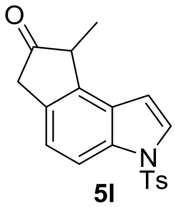

5l

Compound 5m. Synthesised according to representative procedure C from **4m** (30 mg, 0.11 mmol) to afford compound **5m** as off-white solid (14 mg, 0.07 mmol, 65%). m.p.: 105–107 °C; ^{1}H NMR (500 MHz, $CDCl_3$): δ 7.28-7.24 (m, 1H), 7.17 (d, J = 8.4 Hz, 1H), 7.11 (d, J = 3.1 Hz, 1H), 6.46 (dd, J = 3.1, 0.8 Hz, 1H), 3.82 (s, 3H), 3.76 (q, J = 7.6 Hz, 1H), 3.68-3.64 (m, 2H), 1.56 (d, J = 7.5 Hz, 3H); ^{13}C NMR (125 MHz, $CDCl_3$): δ 219.6 (e), 136.5 (e), 134.6 (e), 129.4 (o), 126.5 (e), 124.7 (e), 118.0 (o), 108.8 (o), 98.7 (o), 47.8 (o), 43.1 (e), 33.0 (o), 15.5 (o); IR (neat): $\tilde{\nu}$ = 3117 (w), 3096 (w), 2976 (w), 2934 (w), 2901 (w), 2869 (w), 2824 (w), 1872 (w), 1735 (s), 1603 (w), 1508 (w), 1482 (m), 1455 (w), 1442 (w), 1421 (s), 1396 (w), 1386 (w), 1365 (w), 1337 (m), 1300 (m), 1287 (m), 1256 (m), 1239 (s), 1218 (m), 1176 (m), 1165 (m), 1146 (m), 1090

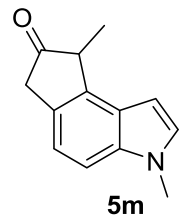

5m

(m), 1065 (w), 1020 (w), 999 (w), 978 (w), 940 (w), 923 (w), 897 (w), 872 (w), 799 (s), 778 (s), 760 (w), 734 (s), 690 (w) cm^{-1}; HRMS (CI(CH_4)): *m/z* calcd for $C_{13}H_{13}NO+H^+$: 200.1070 $[M+H]^+$; found 200.1067.

Compound 5n. Synthesised according to representative procedure C from **4m** (83 mg, 0.26 mmol) to afford compound **5n** as pale-yellow oil (46 mg, 0.19 mmol, 74%) as mixture of regioisomers (6:1). ^1H NMR (500 MHz, $CDCl_3$): δ 7.39 (d, *J* = 7.4 Hz, 0.17H) (*minor*), 7.36–7.29 (m, 2H) (*major* + *minor*), 7.28–7.24 (m, integration not possible due to overlap with residual $CHCl_3$), 7.19 (d, J = 7.1 Hz, 0.15H) (*minor*), 7.14-7.08 (m, 2.4H) (*major* + *minor*), 7.03 (d, *J* = 8.8 Hz, 2H) (*major* + *minor*), 6.93 (br s, 0.8H) (*major*), 6.90–6.81 (m, 1H) (*major* + *minor*), 4.62 (br s) (*major* + *minor*), 3.85 (s, 2.5H) (*major*), 3.78 (s, 0.4H) (*minor*), {3.71–3.59 (m) (*minor*) + 3.64 (s) (*major*), 2H}. The ^1H NMR data of the mixture is in agreement with that described for each separate regioisomer in the literature.[11,13]

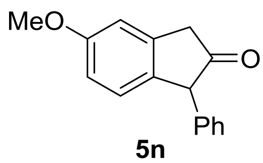

5n
major regioisomer

Compound 5o. Synthesised according to representative procedure C from **4o** (246 mg, 0.66 mmol) to afford compound **5o** as pink solid (120 mg, 0.34 mmol, 51%, 1.3:1 regioisomer ratio). ^1H NMR (500 MHz, $CDCl_3$): δ 8.21 (dd, *J* = 7.9, 1.4 Hz, 1H), 8.18 (dd, *J* = 7.9, 1.4 Hz, 1H), 7.62-7.61 (m, 3H), 7.45-7.42 (m, 2H), 7.38 (br s, 1H), 7.35-7.31 (m, 2H), 7.12 (d, *J* = 7.9 Hz, 1H), 4.41 (s, 2H), 4.37 (s, 2H), 3.77 (d, *J* = 22.5 Hz, 1H), 3.72 (d, *J* = 22.8 Hz, 1H), 3.60-3.43 (m, 4H), 1.41 (d, *J* = 3.7 Hz, 3H), 1.39 (d, *J* = 3.6 Hz, 3H); ^{13}C NMR (100 MHz, $CDCl_3$): δ 216.3 (e), 215.9 (e), 191.3 (e), 190.5 (e), 145.6 (e), 145.3 (e), 140.2 (e), 140.1 (e), 137.1 (e), 136.7 (e), 136.1 (e), 136.0 (e), 135.5 (e), 133.8 (e), 133.7 (e), 133.6 (e), 132.5 (o, 2C), 131.5 (o), 131.4 (o), 130.83 (o), 130.81 (o), 130.6 (o), 127.6 (o), 126.8 (o), 125.1 (o), 122.7 (o), 51.0 (e), 48.3 (o), 48.0 (e), 47.7 (o), 42.4 (e), 42.0 (e), 15.4 (o), 15.3 (o); IR (neat): $\tilde{\nu}$ = 3055 (w), 2971 (w), 2923 (w), 2901 (w), 1746 (s), 1664 (s), 1586 (m), 1473 (w), 1455 (m), 1430 (m), 1395 (m), 1334 (w): 1282 (s), 1236 (m), 1214 (m), 1189 (m), 1155 (m), 1135 (m), 1110 (m), 1072 (m), 1042 (w), 1025 (w), 993 (w), 966 (w), 945 (w), 906 (w), 871 (w), 860 (w), 818 (m), 752 (s), 720 (m), 698 (w), 686 (w), 662 (w) cm^{-1}; HRMS (ESI): *m/z* calcd for $C_{18}H_{14}O_2S+H^+$: 295.0793 $[M+H]^+$; found 295.0750.

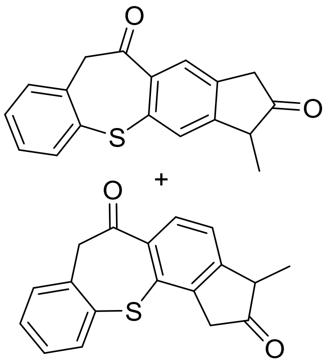

5o

Compound 7. Synthesised according to representative procedure C from **S-13** (159 mg, 0.66 mmol) to afford compound **7** as colourless oil (118 mg, 0.36 mmol, 54%). ^1H NMR (400 MHz, $CDCl_3$): δ 7.28 (d, *J* = 8.7 Hz, 2H), 6.95 (d, *J* = 8.8 Hz, 2H), 5.09 (s, 1H), 4.14 (sept, *J* = 5.8 Hz, 1H), 3.83 (s, 3H), 2.22 (s, 3H); ^{13}C NMR (125 MHz, $CDCl_3$): δ 203.1 (e), 160.9 (e), 129.6 (o, 2C), 124.5 (e), 121.7 (e, q, *J* = 284.5 Hz), 121.2 (e, q, *J* = 281.6 Hz), 114.7 (o, 2C), 88.4 (e), 72.9 (o, sept, *J* = 32.7 Hz), 55.3 (o), 25.8 (o); IR (neat): $\tilde{\nu}$ = 2938 (w), 1730 (s), 1610 (s), 1585 (w), 1513 (s), 1361 (s), 1466 (w), 1361 (s), 1286 (s), 1260 (s), 1219 (s), 1192 (s), 1125 (s), 1102 (s), 1031 (m), 905 (w), 879 (w), 833 (w), 741 (w), 687 (m) cm^{-1}; HRMS (ESI): *m/z* calcd for $C_{13}H_{12}F_6O_3+Na^+$: 353.0583 $[M+Na]^+$; found 353.0584.

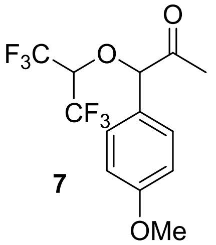

7

Compound 8. Synthesised according to representative procedure C from **S-11** (184 mg, 0.66 mmol) to afford compound **8** as colourless oil (169 mg, 0.46 mmol, 70%). ^1H NMR (500 MHz, $CDCl_3$): δ 7.57 (d, *J* = 8.0 Hz, 2H), 7.32 (d, *J* = 8.0 Hz, 2H), 5.75 (hept, *J* = 6.1 Hz, 1H), 3.08 (t, *J* = 7.6 Hz, 2H), 2.88 (t, *J* = 7.7 Hz, 2H); ^{13}C NMR (125 MHz, $CDCl_3$): δ 169.2 (e), 143.1 (e), 129.2 (e, q, *J* = 32.5 Hz), 128.6 (o, 2C), 126.6 (o, q, *J* = 3.7 Hz, 2C), 124.2 (e, q, *J* = 271.5 Hz), 120.4 (e, q, *J* = 283.6 Hz, 2C), 66.6

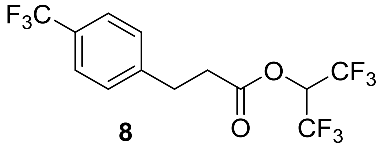

8

[13] A. A. Tudjarian, T. G. Minehan, *J. Org. Chem.* **2011**, *76*, 3576–3581.

(o, hept, J = 33.8 Hz), 34.4 (e), 30.1 (e); ^{19}F NMR (376 MHz, $CDCl_3$): δ -62.56 (s, 3F), -73.13 (s, 6F); IR (neat): $\tilde{\nu}$ = 2971 (w), 1781 (s), 1621 (w), 1420 (w), 1386 (m), 1357 (m), 1325 (s), 1287 (m), 1268 (m), 1228 (s), 1200 (s), 1165 (s), 1106 (vs), 1067 (vs), 1019 (m), 941 (w), 906 (m), 877 (w), 839 (m) cm^{-1}; HRMS (ESI): *m/z* calcd for $C_{13}H_9F_9O_2+Na^+$: 391.0357 $[M+Na]^+$; found 391.0356.

Compound 10. Synthesised according to representative procedure C from **9** (40 mg, 0.1 mmol) to afford

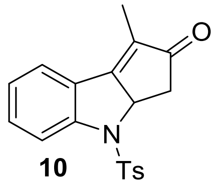

10

compound **10** (10 mg, 0.03 mmol, 31%) as pale-pink powder and **11** (16.1 mg, 0.05 mmol, 50%) as off-white solid. m.p.: 88–90 °C; ^1H NMR (500 MHz, $CDCl_3$): δ 7.86 (d, J = 8.4 Hz, 1H), 7.78 (d, J = 8.2 Hz, 2H), 7.53 (d, J = 7.8 Hz, 1H), 7.49-7.43 (m, 1H), 7.29 (d, J = 8.2 Hz, 2H), 7.15 (td, J = 7.6, 0.9 Hz, 1H), 4.65 (ddq, J = 5.8, 4.2, 2.7 Hz, 1H), 3.15 (dd, J = 17.2, 5.6 Hz, 1H), 2.96 (dd, J = 17.0, 4.2 Hz, 1H), 2.39 (s, 3H), 1.94 (d, J = 2.7 Hz, 3H); ^{13}C NMR (100 MHz, $CDCl_3$): δ 205.0 (e), 163.9 (e), 146.9 (e), 145.1 (e), 132.8 (o), 132.5 (e), 130.6 (e), 130.0 (o, 2C), 127.8 (o, 2C), 124.8 (o), 124.4 (o), 115.6 (o), 66.0 (o), 45.1 (e), 21.6 (o), 8.5 (o); IR (neat): $\tilde{\nu}$ = 3675 (w), 2987 (m), 2901 (m), 1752 (w), 1704 (s), 1655 (s), 1597 (m), 1492 (w), 1452 (s), 1412 (m), 1394 (w), 1375 (w), 1354 (s), 1335 (s), 1302 (m), 1286 (m), 1273 (m), 1233 (s), 1203 (m), 1167 (s), 1155 (s), 1117 (s), 1104 (s), 1081 (s), 1058 (s), 1038 (s), 1014 (s), 976 (w), 955 (m), 905 (m), 867 (w), 830 (w), 914 (m), 814 (s), 763 (m), 742 (s), 705 (m), 656 (w), 661 (s) cm^{-1}; HRMS (ESI): *m/z* calcd for $C_{19}H_{17}NO_3S+Na^+$: 362.0821 $[M+Na]^+$; found 362.0826.

Compound 11. m.p.: 140–143 °C; ^1H NMR (500 MHz, $CDCl_3$): δ 8.11 (dt, J = 8.4, 0.9 Hz, 1H); 7.74 (d, J = 8.4 Hz, 2H); 7.44 (d, J = 7.8 Hz, 1H); 7.37-7.30 (m, 1H); 7.27 (dd, J = 7.7, 1.1 Hz, 1H); 7.24 (d, J = 7.8

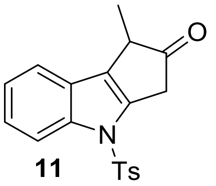

11

Hz, 2H); 3.78 (s, 2H); 3.52 (q, J = 7.4 Hz, 1H); 2.36 (s, 3H); 1.44 (d, J = 7.3 Hz, 3H); ^{13}C NMR (100 MHz, $CDCl_3$): δ 215.0 (e), 145.2 (e), 137.8 (e), 135.6 (e), 135.3 (e), 130.1 (o, 2C), 126.7 (e), 126.6 (o, 2C), 125.5 (e), 124.5 (e), 123.7 (o), 114.4 (o), 44.4 (o), 40.6 (e), 21.6 (o), 15.4 (o); IR (neat): $\tilde{\nu}$ = 3054 (w), 2971 (w), 2911 (w), 1749 (s), 1594 (m), 1491 (w), 1478 (w), 1446 (m), 1417 (w), 1397 (w), 1364 (s), 1345 (m), 1328 (m), 1303 (w), 1294 (w), 1266 (w), 1239 (m), 1218 (w), 1197 (W), 1185 (s), 1164 (s), 1141 (m), 1120 (m), 1103 (w), 1086 (s), 1047 (m), 1008 (m), 955 (w), 931 (w), 913 (m), 808 (m), 796 (m), 759 (m), 745 (s), 700 (w), 666 (s) cm^{-1}; HRMS (ESI): *m/z* calcd for $C_{19}H_{17}NO_3S+Na^+$: 362.0821 $[M+Na]^+$; found 362.0823.

Compound 13. Synthesised according to representative procedure C from **12** (100 mg, 0.38 mmol) to afford

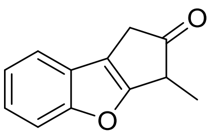

13

compound **13** as off-white solid (61 mg, 0.33 mmol, 87%). m.p.: 58–61 °C; ^1H NMR (500 MHz, $CDCl_3$): δ 7.53 (d, J = 8.0 Hz, 1H), 7.49 (d, J = 8.0, 1H), 7.33-7.26 (m, 2H), 3.56-3.48 (m, 2H), 3.44 (d, J = 21.1 Hz, 1H), 1.46 (d, J = 7.4 Hz, 3H); ^{13}C NMR (125 MHz, $CDCl_3$): δ 214.0 (e), 160.5 (e), 157.5 (e), 125.2 (e), 124.0 (o), 123.2 (o), 119.6 (e), 114.6 (e), 112.0 (o), 43.6 (e), 38.2 (e), 14.3 (o); IR (neat): $\tilde{\nu}$ = 3040 (w), 2960 (w), 2941 (w), 2917 (w), 1750 (s), 1728 (m), 1677 (w), 1616 (w), 1500 (w), 1479 (w), 1445 (s), 1430 (m), 1415 (m), 1359 (w), 1312 (w), 1280 (w), 1227 (s), 1199 (w), 1174 (m), 1145 (m), 1113 (m), 1078 (m), 1043 (s), 1013 (s), 996 (m), 959 (w), 934 (w), 913 (w), 895 (w), 865 (w), 823 (w), 774 (w), 748 (vs), 726 (m), 711 (m) cm^{-1}; HRMS (CI(CH_4)): *m/z* calcd for $C_{12}H_{10}O_2+H^+$: 187.0754 $[M+H]^+$; found 187.0750.

Compound 15. Obtained from **14** (140 mg, 0.66 mmol) according to representative procedure C; colourless

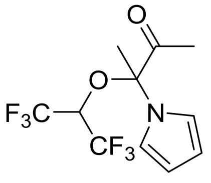

15

oil (84 mg, 0.28 mmol, 42%). ^1H NMR (500 MHz, $CDCl_3$): δ 6.75 (t, J = 2.2 Hz, 2H), 6.30 (t, J = 2.2 Hz, 2H), 3.59 (sept, J = 5.9 Hz, 1H), 2.40 (s, 3H), 1.93 (s, 3H); ^{13}C NMR (125 MHz, $CDCl_3$): δ 202.4 (e), 121.1 (e, q, J = 282.3 Hz), 120.9 (e, q, J = 282.3 Hz), 119.6 (o, 2C), 111.0 (o, 2C), 93.5 (e), 69.8 (o, sept, J = 33.4 Hz), 25.3 (o), 22.1 (o); ^{19}F NMR (376 MHz, $CDCl_3$): δ -72.6 (q, J = 9.1 Hz, 3F), -73.0 (q, J = 9.4 Hz, 3F); IR (neat): $\tilde{\nu}$ = 2944 (w), 1737 (s), 1477 (w), 1421 (w), 1359 (s), 1283 (s), 1258 (m), 1227 (s), 1194

(s), 1101 (s), 1080 (s), 1044 (w), 1000 (w), 970 (w), 949 (w), 897 (s), 805 (w), 730 (s), 687 (s), 687 (s), 663 (w) cm^{-1}; HRMS (ESI): *m/z* calcd for $C_{11}H_{11}F_6NO_2$+H^+: 304.0767 $[M+H]^+$; found 304.0766.

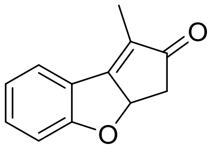

26

Compound 26. Synthesised according to representative procedure C from **25** (50 mg, 0.19 mmol) to afford compound **26** as cream-coloured solid (26 mg, 0.14 mmol, 75%) alongside compound **27** as amorphous solid (3.5 mg, 0.02 mmol, 10%). m.p.: 75–77 °C; ^{1}H NMR (500 MHz, $CDCl_3$): δ 7.59 (dd, *J* = 7.7, 1.1 Hz, 1H), 7.44-7.38 (m, 1H), 7.06 (td, *J* = 7.6, 1.0 Hz, 1H), 7.01-6.97 (m, 1H), 5.53 (ddq, *J* = 5.9, 4.0, 2.6 Hz, 1H), 3.06 (dd, *J* = 16.2, 5.8 Hz, 1H), 2.80 (dd, *J* = 16.4, 4.1 Hz, 1H), 1.99 (d, *J* = 2.8 Hz, 3H); ^{13}C NMR (125 MHz, $CDCl_3$): δ 203.9 (e), 169.6 (e), 165.1 (e), 133.5 (o), 130.3 (e), 124.5 (o), 122.3 (e), 122.0 (o), 112.1 (o), 85.6 (o), 44.1 (e), 8.6 (o); IR (neat): $\tilde{\nu}$ = 3087 (w), 3056 (w), 3015 (w), 2990 (w), 2933 (w), 2908 (w), 1705 (s), 1657 (s), 1603 (s), 1476 (w), 1453 (s), 1445 (s), 1411 (w), 1376 (m), 1346 (m), 1335 (s), 1285 (s), 1270 (m), 1233 (m), 1208 (s), 1181 (w), 1155 (m), 1114 (m), 1100 (m), 1057 (s), 1014 (w), 985 (m), 972 (s), 946 (m), 891 (m), 870 (w), 849 (m), 801 (m), 767 (s), 756 (s), 731 (m), 691 (w), 655 (w) cm^{-1}; HRMS (CI(CH_4)): *m/z* calcd for $C_{12}H_{10}O_2$+H^+: 187.0754 $[M+H]^+$; found 187.0752.

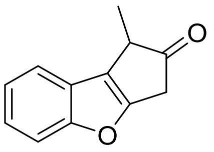

27

Compound 27. ^{1}H NMR (500 MHz, $CDCl_3$): δ 7.56–7.46 (m, 2H), 7.34–7.21 (m, 2H), 3.66–3.54 (m, 2H), 3.52 (d, *J* = 22.6 Hz, 1H), 1.15 (d, *J* = 7.3 Hz, 3H); ^{13}C NMR (125 MHz, $CDCl_3$): δ 214.0, 157.6 (e), 154.6 (e), 125.1 (e), 124.0 (o), 123.2 (o), 121.9 (e), 119.1 (o), 112.0 (o), 45.1 (e), 38.3 (o), 15.5 (o); IR (neat): $\tilde{\nu}$ = 3052 (w), 2992 (w), 2932 (w), 2904 (w), 2873 (w), 1955 (w), 1914 (w), 1751 (w), 1622 (m), 1478 (w), 1445 (m), 1431 (w), 1417 (w), 1369 (w), 1349 (m), 1308 (m), 1288 (w), 1263 (w), 1225 (m), 1188 (m), 1167 (m), 1144 (m), 1132 (m), 1110 (m), 1056 (w), 1015 (w), 979 (m), 935 (w), 910 (w), 859 (w), 828 (m), 761 (s), 752 (s), 723 (w), 703 (m), 688 (w), 656 (w) cm^{-1}; HRMS (CI(CH_4)): *m/z* calcd for $C_{12}H_{10}O_2$+H^+: 187.0754 $[M+H]^+$; found 187.0751,

Hammett study of the cyclisation of sulfoxonium ylides in the presence of K_2CO_3 in HFIP.

The reactions were conducted according the representative procedure C and the conversion was determined by ^{1}H NMR after 5 and 10 minutes for each substrate; kobs was determined from the slope of the graphs of ln([**4**]) = f(t).

Figure S1 – Hammett plot of the conversion of **4** into **5** according to representative procedure C.

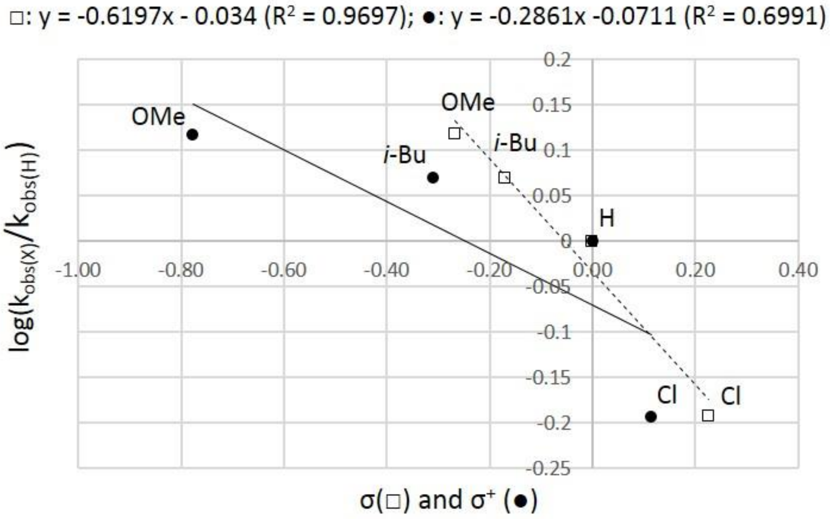

Synthesis of compounds 16, 18a–h, 19, 20, 22, and 24

Optimisation of the reaction conditions

Table S2. Optimization of the cyclization of **14** into **16**

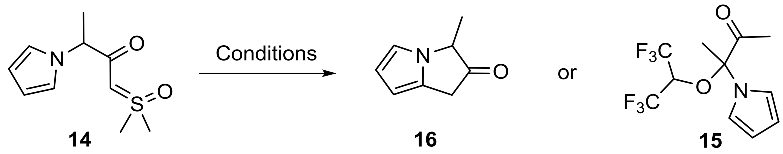

Entry	Conditions	Product, yield[a]
1	K_2CO_3 (1 equiv), HFIP, 60 °C, 16 h	**15**, 15%
2	{[Ir(cod)Cl]$_2$} (10 mol%), 1,2-DCE, 80 °C, 10 minutes	**16**, 60%
3	{[Ir(cod)Cl]$_2$} (1 mol%), 1,2-DCE, 80 °C (mW), 2 h	**16**, 64%

[a] Yield of isolated product. cod: cyclooctadiene. mW: microwave.

Representative procedure D – To a flame dried microwave vial under N_2 were added 14 (64 mg, 0.3 mmol) and [Ir(cod)Cl]$_2$ (2.1 mg, 0.003 mmol), and the vial was evacuated and back-filled with N_2 three times. 1,2-DCE was added (15 mL), and the reaction mixture was degassed with an argon balloon for 30 mins before the vial was sealed and heated to 80 °C in a microwave oven (Anton Paar Microwave Synthesis Reactor Monowave 300) for 2 h. The reaction was let to cool to room temperature and concentrated under vacuum. The residue was purified by flash chromatography (petroleum ether/EtOAc, 9:1) on SiO_2 to give **16** as dark-green solid (26 mg, 0.19 mmol, 64%).

Compound 16. m.p.: 46–48 °C; ^{1}H NMR (500 MHz, $CDCl_3$): δ 6.80-6.71 (m, 1H), 6.32-6.21 (m, 1H), 6.05-5.95 (m, 1H), 4.39 (q, *J* = 6.9 Hz, 1H), 3.62-3.45 (m, 2H), 1.54 (d, *J* = 7.1 Hz, 3H); ^{13}C NMR (100 MHz, $CDCl_3$): δ 212.5 (e), 128.2 (e), 114.0 (o), 111.1 (o), 101.4 (o), 59.8 (o), 36.5 (e), 17.3 (o); IR (neat): $\tilde{\nu}$ = 3500 (w), 3042 (w), 2998 (w), 2978 (w), 2934 (w), 2901 (w), 2873 (w), 1755 (s), 1717 (w), 1625 (w), 1568 (w), 1551 (w), 1468 (w), 1455 (w), 1447 (w), 1399 (w), 1366 (m), 1312 (m), 1294 (m), 1255 (s), 1229 (w), 1193 (w), 1181 (w), 1116 (w), 1103 (w), 1073 (m), 1064 (m), 1032 (m), 1025 (m), 1003 (w), 977 (w), 937 (w), 904 (m), 898 (m), 874 (w), 857 (w), 815 (w), 770 (w), 724 (m), 704 (s) cm^{-1}; HRMS (CI(CH_4)): *m/z* calcd for $C_8H_9NO+H^+$: 136.0757 $[M+H]^+$; found 136.0762.

16
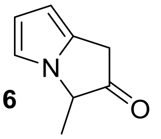

Compound 18a. Synthesised according to representative procedure D from **17a** (60 mg, 0.3 mmol) to afford compound **18a** as dark-green solid (27 mg, 0.22 mmol, 74%). ^{1}H NMR (500 MHz, $CDCl_3$): δ 6.77 (dd, *J* = 2.8, 1.2 Hz, 1H); 6.27 (t, *J* = 3.1 Hz, 1H); 6.02 (dq, *J* = 3.5, 1.2 Hz, 1H); 4.40 (s, 2H); 3.54 (d, *J* = 1.0 Hz, 2H); ^{13}C NMR (100 MHz, $CDCl_3$): δ 209.6 (e), 130.2 (e), 115.4 (o), 111.2 (o), 101.8 (o), 54.4 (e), 37.4 (e). The NMR data obtained is in agreement with that described previously.[14]

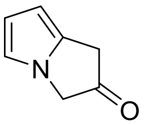
18a

[14] Jefford, C. W.; Johncock, W. *Helv. Chim. Acta.* **1983**, *66*, 2666–2671.

Compound 18b. Synthesised according to representative procedure D from **17b** (41 mg, 0.1 mmol) using 5 mol% of $[Ir(cod)Cl]_2$ for 3 hours to afford compound **18b** as a purple powder (20.5 mg, 0.06 mmol, 61%). m.p.: 115–116 °C; ^{1}H NMR (500 MHz, $CDCl_3$): δ 6.49 (d, *J* = 3.9 Hz, 1H), 6.07 (d, *J* = 3.8 Hz, 1H), 4.67 (s, 2H), 3.89–3.71 (m, 4H), 3.52-3.49 (m, 6H), 1.48 (s, 9H); ^{13}C NMR (100 MHz, $CDCl_3$): δ 208.7 (e), 162.2 (e), 154.6 (e), 134.4 (e), 121.6 (e), 115.4 (o), 102.7 (o), 80.3 (e), 55.9 (e), 44.9 (e)*, 43.8 (e)*, 37.2 (e), 28.4 (o, 3C) (peaks marked by an asterisk are very broad due to slow rotation of Boc group); IR (neat): $\tilde{\nu}$ = 2978 (w), 2903 (w), 1766 (s), 1690 (s), 1617 (s), 1552 (w), 1475 (s), 1409 (s), 1368 (m), 1283 (m), 1242 (s), 1164 (s), 1132 (s), 1069 (m), 1041 (m), 995 (m), 977 (m), 862 (m), 839 (w), 822 (w), 809 (w), 767 (m), 735 (s), 669 (w) cm^{-1}; HRMS (ESI): *m/z* calcd for $C_{17}H_{23}N_3O_4+Na^+$: 356.1581 $[M+Na]^+$; found 356.1585.

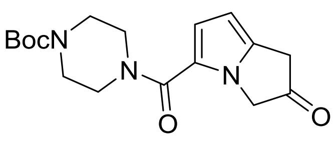

18b

Compound 18c. Synthesised according to representative procedure D from **17c** (28 mg, 0.10 mmol) to afford compound **18c** as yellow gum (17 mg, 0.08 mmol, 90%). ^{1}H NMR (500 MHz, $CDCl_3$): δ 7.39-7.33 (m, 3H), 7.10-7.05 (m, 2H), 6.71 (dd, *J* = 2.7, 1.1 Hz, 1H), 6.35 (t, *J* = 3.1 Hz, 1H), 6.12 (dq, *J* = 3.5, 1.1 Hz, 1H), 5.38 (s, 1H), 3.63 (s, 2H); ^{13}C NMR (125 MHz, $CDCl_3$): δ 208.5 (e), 135.9 (e), 129.2 (e), 129.0 (o,, 128.6 (o), 126.2 (o,, 115.3 (o), 111.7 (o), 101.6 (o), 68.1 (o), 36.2 (e); IR (neat): $\tilde{\nu}$ = 3085 (w), 3068 (w), 3028 (w), 2901 (w), 1956 (w), 1759 (s), 1601 (w), 1582 (w), 1555 (w), 1523 (w), 1495 (m), 1470 (m), 1454 (m), 1438 (w), 1401 (m), 1350 (m), 1296 (m), 1279 (m), 1249 (m), 1222 (m), 1193 (w), 1185 (m), 1129 (w), 1094 (m), 1078 (m), 1057 (m), 1030 (w), 1013 (w), 986 (w), 967 (w), 934 (w), 901 (m), 845 (w), 826 (w), 781 (w), 770 (m), 759 (m), 740 (m), 724 (s), 715 (m), 700 (s) cm^{-1}; HRMS (CI(CH_4)): *m/z* calcd for $C_{13}H_{11}NO+H^+$: 198.0913 $[M+H]^+$; found 198.0922.

18c

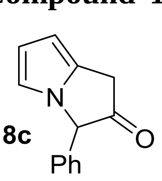

Compound 18d. Synthesised according to representative procedure D from **17d** (29 mg, 0.10 mmol) to afford compound **18d** as purple gum (20 mg, 0.09 mmol, 95%). ^{1}H NMR (500 MHz, $CDCl_3$): δ 7.26-7.20 (m, 3H), 6.99 (dd, *J* = 6.4, 2.8 Hz, 2H), 6.50 (d, *J* = 1.9 Hz, 1H), 6.20 (t, *J* = 3.1 Hz, 1H), 5.87-5.94 (m, 1H), 4.69-4.61 (m, 1H), 3.44-3.33 (m, 2H), 3.00-3.10 (m, 2H); ^{13}C NMR (125 MHz, $CDCl_3$): δ 211.7 (e), 135.1 (e), 129.2 (o, 2C), 128.7 (e), 128.3 (o, 2C), 126.9 (o), 114.6 (o) , 110.8 (o), 101.1 (o), 65.0 (o) , 38.5 (o), 36.7 (o); IR (neat): $\tilde{\nu}$ = 3138 (w), 3113 (w), 3062 (w), 2901 (w), 1970 (w), 1758 (s), 1600 (w), 1585 (w), 1552 (w), 1496 (w), 1468 (m), 1455 (m), 1403 (m), 1380 (w), 1343 (w), 1295 (m), 1267 (w), 1250 (m), 1214 (w), 1187 (m), 1166 (m), 1128 (w), 1085 (m), 1052 (m), 1028 (w), 1010 (w), 998 (w), 974 (w), 946 (w), 921 (w), 871 (w), 853 (m), 836 (w), 779 (m), 765 (m), 747 (m): 715 (s): 700 (s) cm^{-1}; HRMS (CI(CH_4)): *m/z* calcd for $C_{14}H_{13}NO+H^+$: 212.1070 $[M+H]^+$; found 212.1080.

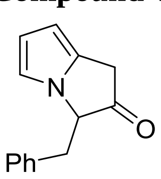

18d

Compound 18e. Synthesised according to representative procedure D from **17e** (91 mg, 0.3 mmol) using 2.5 mol% of $[Ir(cod)Cl]_2$ at 100 °C to afford compound **18e** as yellow oil (54 mg, 0.24 mmol, 79%).^{1}H NMR (500 MHz, $CDCl_3$): δ 7.29-7.26 (m, 2H), 7.22-7.18 (m, 1H), 7.14-7.12 (m, 2H), 6.80 (dd, *J* = 2.7, 1.2 Hz, 1H), 6.32-6.30 (m, 1H), 6.04-6.02 (m, 1H), 4.39 (td, *J* = 5.4, 1.0 Hz, 1H), 3.52 (d, *J* = 22.9 Hz, 1H), 3.39 (dt, *J* = 22.8, 1.1 Hz, 1H), 2.67-2.56 (m, 2H), 2.35-2.24 (m, 2H); ^{13}C NMR (125 MHz, $CDCl_3$): δ 212.4 (e), 140.2 (e), 129.0 (e), 128.48 (o, 2C), 128.50 (o, 2C), 126.3 (o), 114.4 (o), 111.4 (o), 101.6 (o), 63.5 (o), 37.1 (e), 33.9 (e), 30.6 (e); IR (neat): $\tilde{\nu}$ = 3026 (w), 2923 (w), 1759 (s), 1602 (w), 1558 (w): 1496 (w), 1470 (m), 1454 (m), 1403 (m), 1403 (m), 1257 (m), 1193 (w), 1180 (w), 1099 (m), 1080 (w), 1058 (m), 1029 (w), 903 (m), 846 (w), 776 (m), 748 (m), 699 (s) cm^{-1}; HRMS (CI(CH_4)): *m/z* calcd for $C_{15}H_{15}NO+H^+$: 226.1226 $[M+H]^+$; found 226.1232.

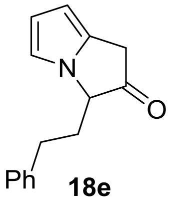

18e

Compound 18f. Synthesised according to general procedure D from compound **17f** (105 mg, 0.3 mmol) to afford compound **18f** as pale-brown amorphous solid (76 mg, 0.28 mmol, 94%).^{1}H NMR (500 MHz, $CDCl_3$): δ 6.81 (d, *J* = 8.3 Hz, 1H), 6.69 (br s, 1H), 6.61 (dd, *J* = 8.3, 2.2 Hz, 1H), 6.41 (d, *J* = 2.1 Hz, 1H), 6.19 (t, *J* = 3.2 Hz, 1H), 6.00 (d, *J* = 2.8 Hz, 1H), 4.49 (dd, *J* = 13.1, 5.4 Hz, 1H), 4.39 (dd, *J* = 13.1, 7.2 Hz, 1H), 3.87-3.74 (m, 3H), 3.85 (s, 3H), 3.78 (s, 3H); ^{13}C NMR (125 MHz, $CDCl_3$): δ 205.6 (e), 149.2 (e), 148.6 (e), 128.4 (e), 124.8 (e), 120.4 (o), 119.6 (o), 111.4 (o), 111.0 (o), 108.8 (o), 106.0 (o), 55.84 (o), 55.81 (o), 55.1 (o), 48.8 (e), 37.8 (e); IR (neat): $\tilde{\nu}$ = 3135 (w), 3007 (w), 2967 (w), 2938 (w), 2876 (w), 2841 (w), 1724 (s), 1633 (w), 1605 (w), 1592 (w), 1550 (w), 1515 (s), 1487 (m), 1464 (m), 1445 (m), 1418 (w), 1400 (w), 1373 (w), 1345 (w), 1328 (w), 1299 (w), 1275 (s), 1245 (s), 1235 (s), 1223 (s), 1187 (m), 1162 (m), 1143 (s), 1128 (m), 1067 (w), 1021 (s), 982 (w), 945 (w), 925 (w), 886 (w), 849 (m), 831 (w), 814 (m): 793 (w), 780 (w), 763 (w), 719 (s) cm^{-1}; HRMS (ESI): *m/z* calcd for $C_{16}H_{17}NO_3+Na^+$: 294.1101 $[M+Na]^+$; found 294.1102.

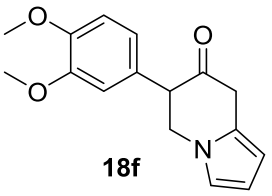
18f

Compound 18g. Synthesised according to representative procedure D from compound **17g** (109 mg, 0.3 mmol) to afford compound **18g** as pale-brown amorphous solid (77 mg, 0.27 mmol, 90%).^{1}H NMR (500 MHz, $CDCl_3$): δ 6.82 (d, *J* = 8.2 Hz, 1H), 6.72 (dd, *J* = 8.3, 2.0 Hz, 1H), 6.69 (d, *J* = 2.0 Hz, 1H), 6.66 (dd, *J* = 2.6, 1.9 Hz, 1H), 6.10 (t, *J* = 3.3 Hz, 1H), 6.06-6.05 (m, 1H), 4.28–4.22 (m, 2H), 3.92-3.86 (m, 2H), 3.86 (s, 3H), 3.85 (s, 3H), 3.76 (d, *J* = 15.8 Hz, 1H), 2.46-2.39 (m, 1H), 2.33-2.26 (m, 1H); ^{13}C NMR (125 MHz, $CDCl_3$): δ 206.4 (e), 148.7 (e), 148.2 (e), 130.3 (e), 124.5 (e), 121.3 (o), 120.5 (o), 111.9 (o), 111.0 (o), 108.4 (o), 107.3 (o), 56.7 (o), 55.81 (o), 55.78 (o), 46.4 (e), 42.4 (e), 35.7 (e); IR (neat): $\tilde{\nu}$ = 3101 (w), 2998 (w), 2916 (w), 2841 (w), 1706 (m), 1594 (w), 1537 (m), 1518 (w), 1488 (w), 1449 (w), 1384 (m), 1353 (w), 1328 (m), 1301 (m), 1283 (w), 1249 (m), 1236 (m), 1173 (s), 1136 (m), 1090 (m), 1065 (w), 1024 (s), 958 (w), 883 (w), 857 (m), 822 (w), 800 (w), 762 (w), 722 (s), 680 (w) cm^{-1}; HRMS (ESI): *m/z* calcd for $C_{17}H_{19}NO_3+Na^+$: 308.1257 $[M+Na]^+$; found 308.1262.

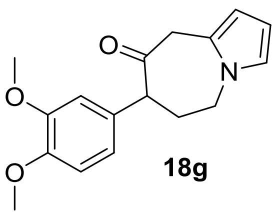
18g

Compound 18h. Synthesised according to representative procedure D from compound **17h** (95 mg, 0.3 mmol) using 5 mol% of $[Ir(cod)Cl]_2$ to afford compound **18h** as pale-brown amorphous solid (40 mg, 0.17 mmol, 56%). ^{1}H NMR (500 MHz, $CDCl_3$): δ 7.32-7.27 (m, 2H), 7.25-7.20 (m, 3H), 6.69–6.65 (m, 1H), 6.22 (t, *J* = 3.0 Hz, 1H), 6.08–6.04 (m, 1H), 4.08 (ddd, *J* = 15.3, 8.2, 2.7 Hz, 1H), 3.83 (ddd, *J* = 15.4, 7.9, 3.1 Hz, 1H), 3.80 (s, 2H), 3.75 (dd, *J* = 11.1, 2.7 Hz, 1H), 2.14-2.05 (m, 1H), 2.04-1.87 (m, 2H), 1.82-1.72 (m, 1H); ^{13}C NMR (100 MHz, $CDCl_3$): δ 211.2 (e), 140.1 (e), 128.4 (o, 2C), 128.0 (o, 2C), 127.0 (o), 126.3 (e), 121.2 (o), 108.9 (o), 108.6 (o), 55.6 (o), 45.9 (e), 41.6 (e), 32.2 (e), 30.6 (e); IR (neat): $\tilde{\nu}$ = 2927 (w), 1707 (s), 1600 (w), 1484 (m), 1452 (m), 1398 (w), 1352 (w), 1304 (m), 1277 (w), 1208 (w), 1102 (w), 1075 (m), 1030 (w), 971 (w), 939 (w), 910 (w), 878 (w), 826 (w), 795 (m); 758 (m), 698 (s) cm^{-1}; HRMS (ESI): *m/z* calcd for $C_{16}H_{17}NO+Na^+$: 262.1208 $[M+Na]^+$; found 262.1209.

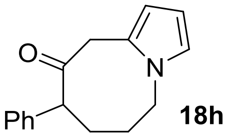
18h

Compound 20. Synthesised according to representative procedure D from compound **19** (28 mg, 0.1 mmol), to afford compound **20** as a pale-pink powder (17 mg, 0.08 mmol, 85%). ^{1}H NMR (500 MHz, $CDCl_3$): δ 7.51 (d, *J* = 7.6 Hz, 1H), 7.30 (d, *J* = 8.3 Hz, 1H), 7.25-7.20 (m, 1H), 7.16-7.11 (m, 1H), 3.64–3.61 (m, 5H), 3.14-3.09 (m, 2H), 2.78 (t, *J* = 6.7 Hz, 2H); ^{13}C NMR (125 MHz, $CDCl_3$): δ 208.0 (e), 137.6 (e), 131.8 (e), 126.0 (e), 121.4 (o), 119.3 (o), 118.0 (o), 108.4 (e), 39.5 (e), 37.5 (e), 29.2 (o), 19.8 (e); IR (neat): $\tilde{\nu}$ = 3051 (w), 2978 (w), 2933 (w), 2856 (w), 1906 (w), 1871 (w), 1705 (s), 1615 (w), 1586 (w), 1567 (w), 1470 (s), 1418 (w), 1375 (m), 1345 (w), 1314 (m), 1291 (w), 1273 (w), 1239 (s), 1203 (w), 1178 (m), 1144 (w), 1129 (w), 1034 (m), 1010 (w), 983 (w), 964 (w), 935 (w), 920 (w), 877 (w), 786 (w), 757 (s), 736 (s), 664 (w); elemental analysis calcd (%) for $C_{13}H_{13}NO$: C 78.36, H 6.58, N 7.03; found: C 78.30, H 6.60, N 6.98.

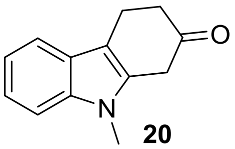
20

Compound 22. Synthesised according to representative procedure D from compound **21** (75 mg, 0.30 mmol), to afford compound **22** as a pale-brown powder (42 mg, 0.25 mmol, 82%). ^{1}H NMR (500 MHz, $CDCl_3$): δ 7.63 (d, *J* = 7.9 Hz, 1H), 7.26-7.19 (m, 2H), 7.18-7.13 (m, 1H), 6.38 (q, *J* = 1.1 Hz, 1H), 4.46 (s, 2H), 3.71 (s, 2H); ^{13}C NMR (100 MHz, $CDCl_3$): δ 208.6 (e), 136.5 (e), 133.5 (e), 130.5 (e), 121.5 (o), 120.8 (o), 120.3 (o), 109.6 (o), 95.7 (o), 52.2 (e), 37.5 (e), HRMS (CI(CH_4)): *m/z* calcd for: $C_{11}H_9NO+H^+$ $[M+H]^+$ 172.0757; found 172.0765. The NMR data is in agreement with that previously reported.[15]

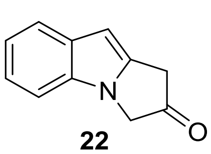
22

Compound 24. Synthesised according to representative procedure D from **23** (83 mg, 0.3 mmol) using 2.5 mol% of $[Ir(cod)Cl]_2$ at 100 °C to afford compound **18e** as cream-coloured solid (49 mg, 0.25 mmol, 82%). m.p.: 91–93 °C; ^{1}H NMR (500 MHz, $CDCl_3$): δ 7.53 (d, *J* = 7.9 Hz, 1H), 7.35 (dt, *J* = 8.2, 0.9 Hz, 1H), 7.25-7.21 (m, 1H), 7.17-7.12 (m, 1H), 3.74 (s, 3H), 3.67 (q, *J* = 7.4 Hz, 1H), 3.56 (d, *J* = 22.0 Hz, 1H), 3.50 (d, *J* = 21.9 Hz, 1H), 1.49 (d, *J* = 7.3 Hz, 3H); ^{13}C NMR (125 MHz, $CDCl_3$): δ 216.5 (e), 139.2 (e), 137.6 (e), 124.2 (e), 121.3 (o), 119.6 (o), 118.6 (o), 117.4 (e), 109.6 (o), 45.3 (o), 37.4 (e), 31.1 (o), 16.4 (o); IR (neat): $\tilde{\nu}$ = 3048 (w), 2973 (w), 2931 (w), 2896 (w), 2858 (w), 2827 (w), 1739 (s), 1612 (w), 1558 (w), 1484 (m),1469 (m), 1448 (w), 1420 (w), 1402 (w), 1382 (m), 1264 (w), 1226 (m), 1182 (w), 1143 (w), 1132 (w), 1113 (w), 1070 (w), 1060 (w), 1011 (m), 958 (w), 930 (w), 904 (w), 856 (w), 805 (w), 746 (s), 669 (w) cm^{-1}; HRMS (CI(CH_4)): *m/z* calcd for $C_{13}H_{13}NO+H^+$: 200.1070 $[M+H]^+$; found 200.1077.

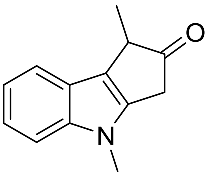
24

[15] Salim, M.; Capretta, A. *Tetrahedron* **2000**, *56*, 8063-8069.

Deuterium-labelling experiments

Compound [D]-22 was observed when representative procedure D was applied to **D-21** (75 mg, 0.30 mmol). The amount and location of deuterium in the product was determined by comparison of ^{1}H and ^{2}H NMR of the crude material. However, purification of this crude material by flash column chromatography on SiO_2 afforded compound **22** without any deuterium content (39 mg, 0.23 mmol, 78%).

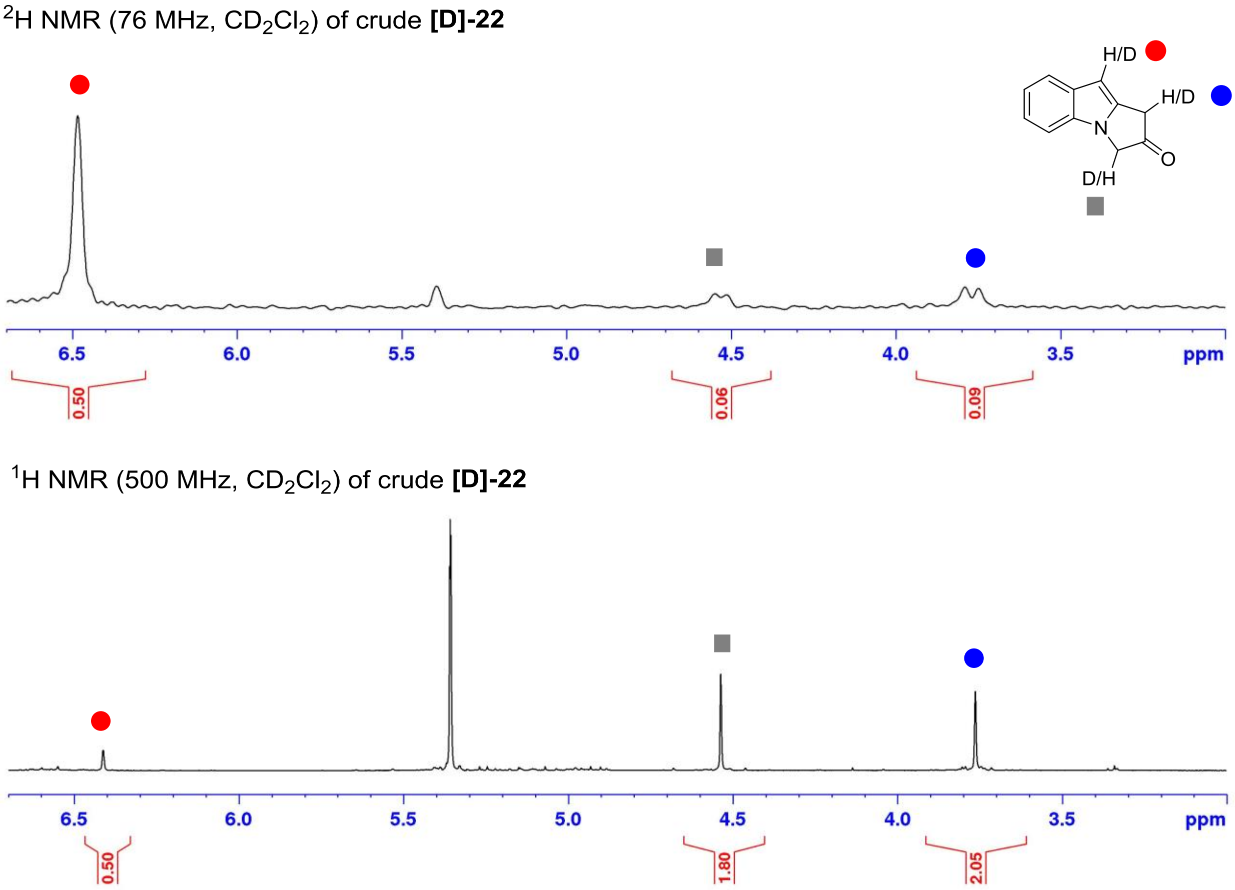

A possible kinetic isotope effect was evaluated by measuring the initial rate in the first 10 minutes of the reactions of **21** and [**D**]-**21** under the conditions described in the representative procedure D. We did not observe any effect (Figure S2).

Figure S2 – Evaluation of kinetic isotope effect

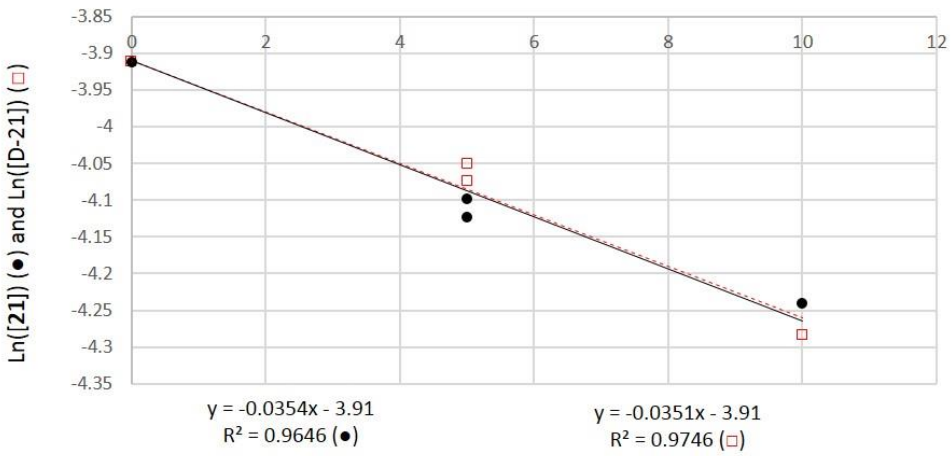

Figure S3 – DSC trace for compound **1**

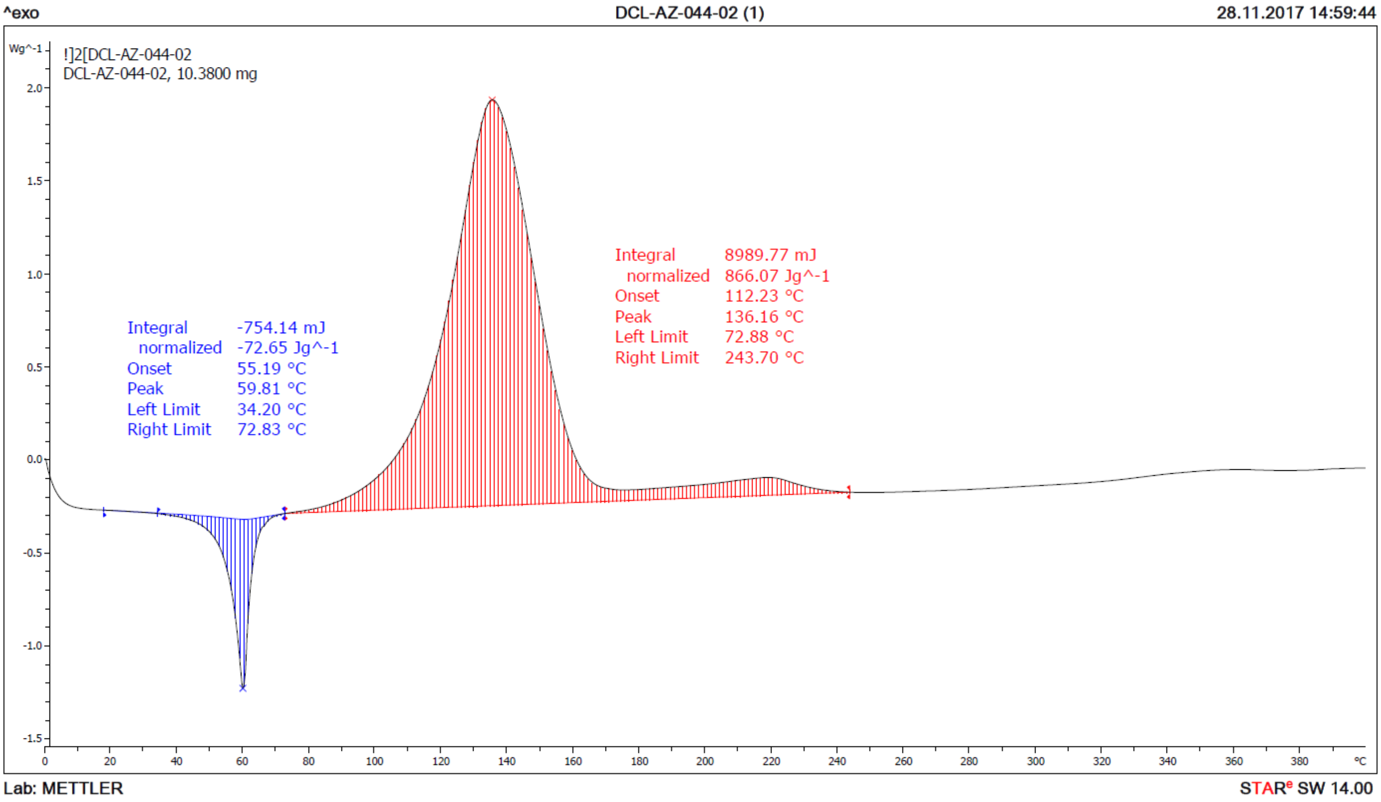

Figure S4 – DSC trace for compound **2**

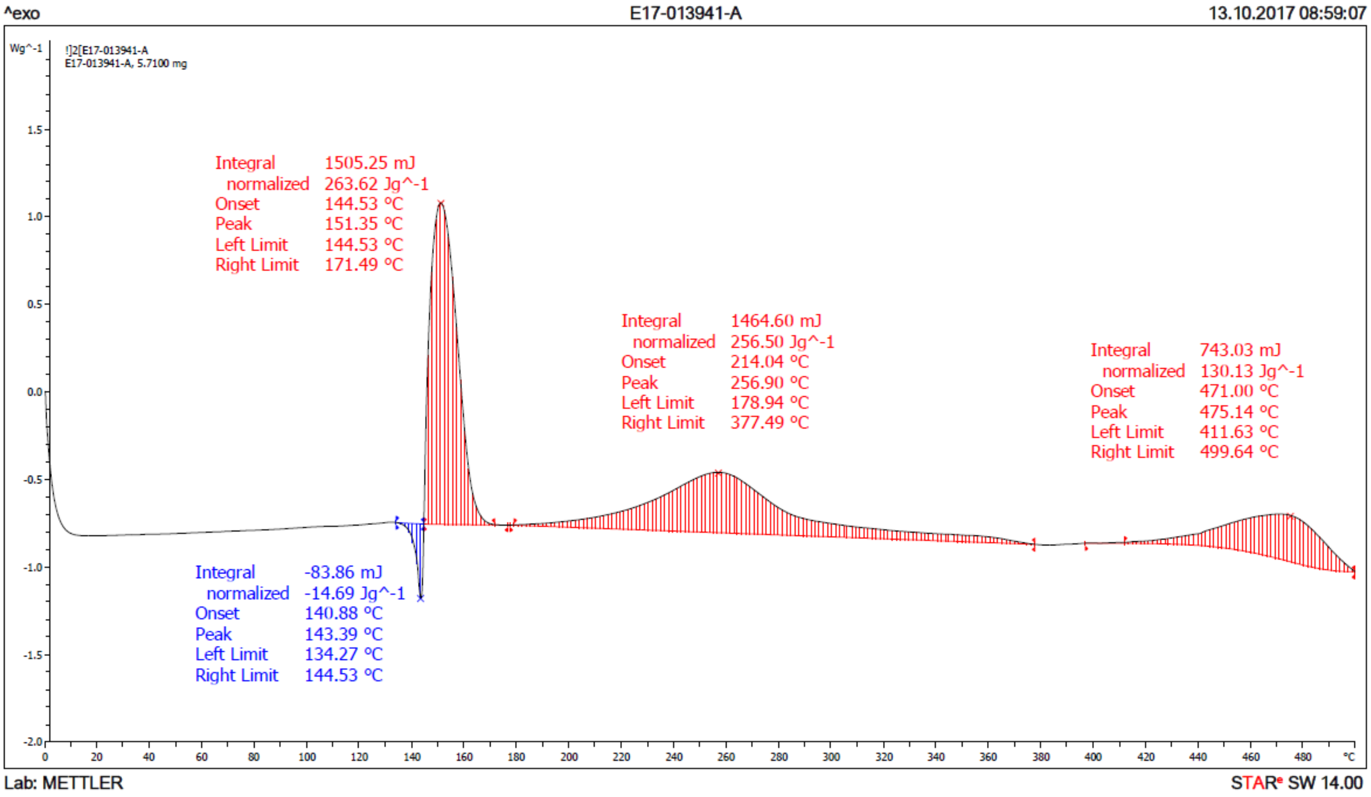

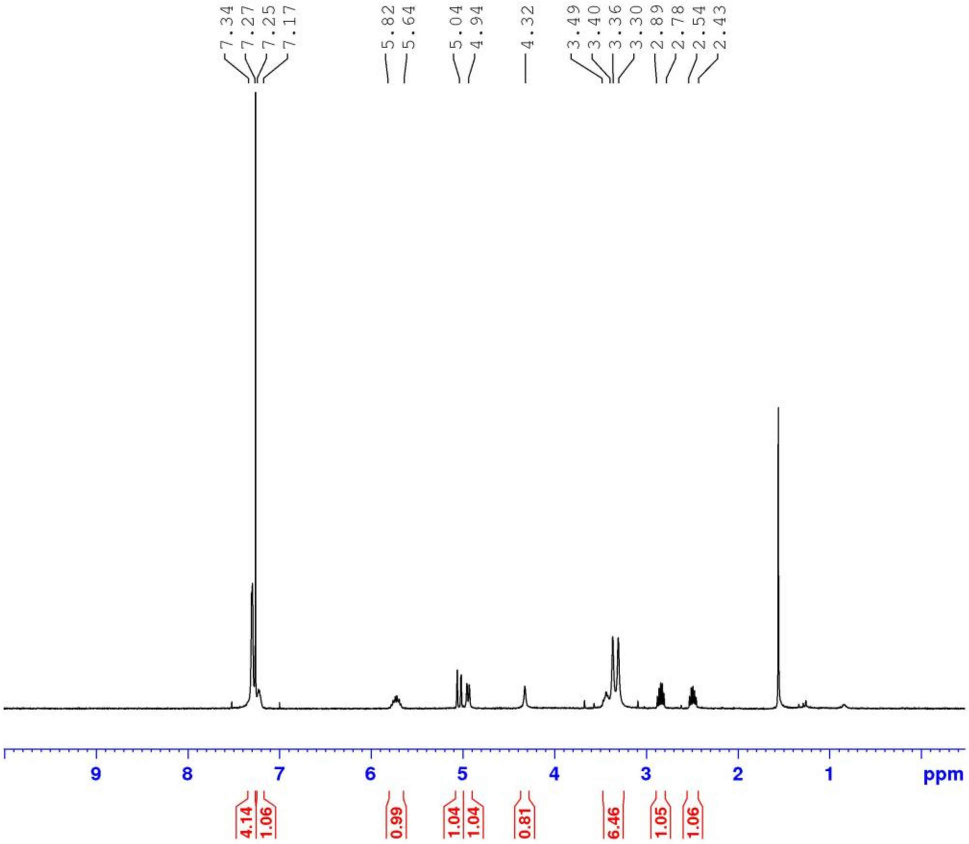

Current Data Parameters
NAME compound 4a
EXPNO 1
PROCNO 1

F2 - Acquisition Parameters
Date_ 20170517
Time 16.39
INSTRUM spect
PROBHD 5 mm QNP 1H/13
PULPROG zg30
TD 65536
SOLVENT CDCl3
NS 16
DS 2
SWH 8278.146 Hz
FIDRES 0.126314 Hz
AQ 3.9583745 sec
RG 512
DW 60.400 usec
DE 6.50 usec
TE 298.1 K
D1 1.00000000 sec
TD0 1

======== CHANNEL f1 ========
NUC1 1H
P1 10.00 usec
PL1 1.00 dB
SFO1 400.1324710 MHz

F2 - Processing parameters
SI 32768
SF 400.1300095 MHz
WDW EM
SSB 0
LB 0.30 Hz
GB 0
PC 1.00

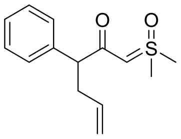

4a

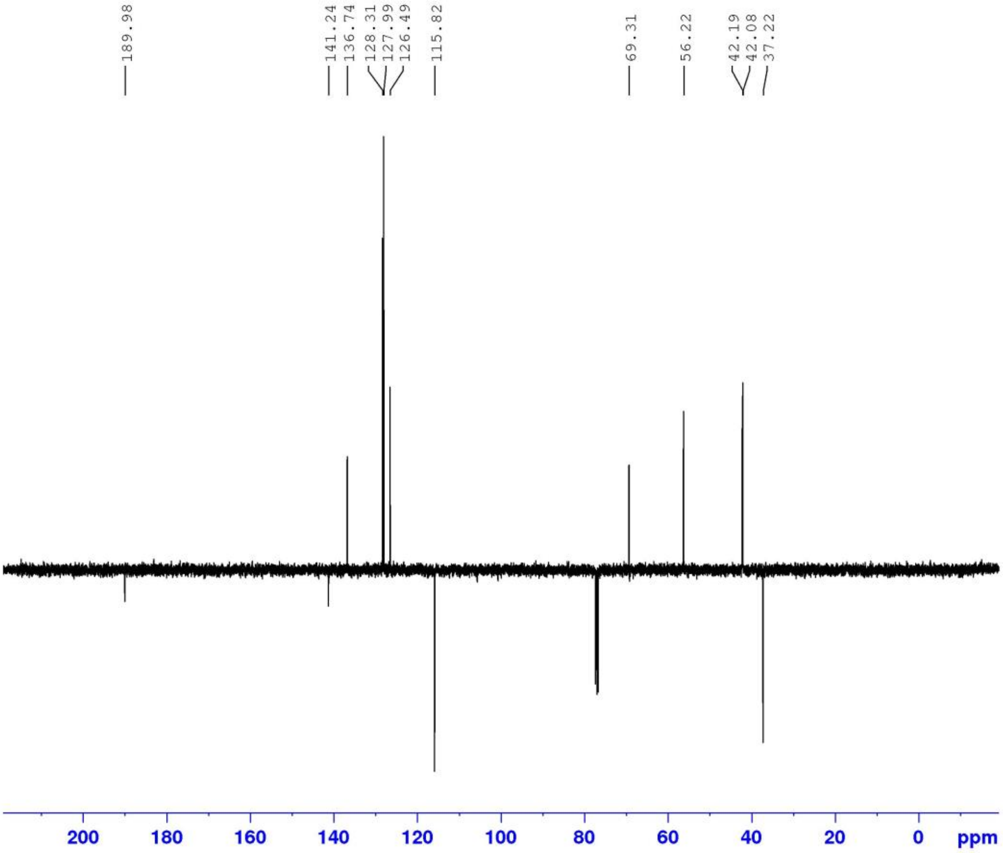

Current Data Parameters
NAME compound 4a
EXPNO 2
PROCNO 1

F2 - Acquisition Parameters
Date_ 20170517
Time 17.18
INSTRUM spect
PROBHD 5 mm QNP 1H/13
PULPROG jmod
TD 65536
SOLVENT CDCl3
NS 256
DS 4
SWH 23980.814 Hz
FIDRES 0.365918 Hz
AQ 1.3664256 sec
RG 16384
DW 20.850 usec
DE 6.50 usec
TE 298.0 K
CNST2 145.0000000
CNST11 1.0000000
D1 2.00000000 sec
D20 0.00689655 sec
TD0 1

======== CHANNEL f1 ========
NUC1 13C
P1 8.00 usec
P2 16.00 usec
PL1 0 dB
SFO1 100.6228298 MHz

======== CHANNEL f2 ========
CPDPRG[2 waltz16
NUC2 1H
PCPD2 80.00 usec
PL2 1.00 dB
PL12 19.06 dB
SFO2 400.1316005 MHz

F2 - Processing parameters
SI 32768
SF 100.6127750 MHz
WDW EM
SSB 0
LB 1.00 Hz
GB 0
PC 1.40

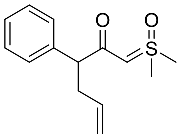

4a

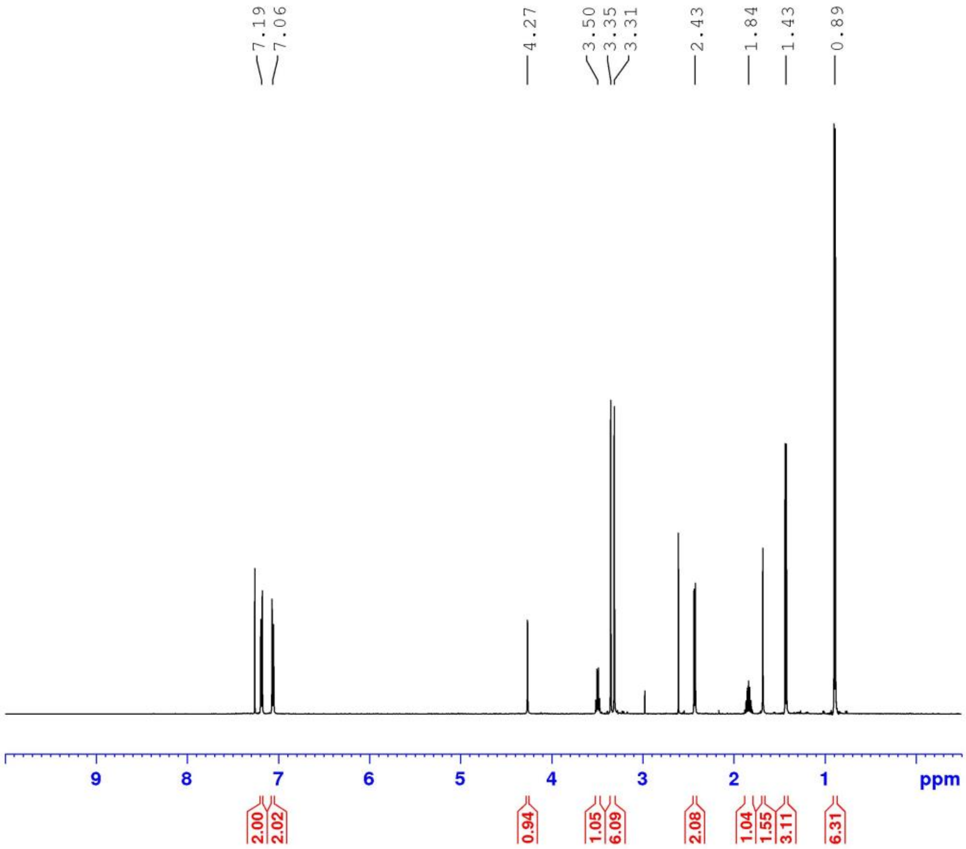

Current Data Parameters
NAME compound 4 b
EXPNO 1
PROCNO 1

F2 - Acquisition Parameters
Date_ 20161011
Time 15.07
INSTRUM spect
PROBHD 5 mm PABBO BB-
PULPROG zg30
TD 65536
SOLVENT CDCl3
NS 16
DS 2
SWH 10000.000 Hz
FIDRES 0.152588 Hz
AQ 3.2767999 sec
RG 198.17
DW 50.000 usec
DE 6.50 usec
TE 298.0 K
D1 1.00000000 sec
TD0 1

======== CHANNEL f1 ========
SFO1 500.1330885 MHz
NUC1 1H
P1 10.00 usec
PLW1 26.00000000 W

F2 - Processing parameters
SI 65536
SF 500.1300123 MHz
WDW EM
SSB 0
LB 0.30 Hz
GB 0
PC 1.00

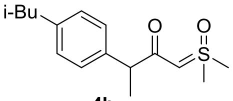

4b

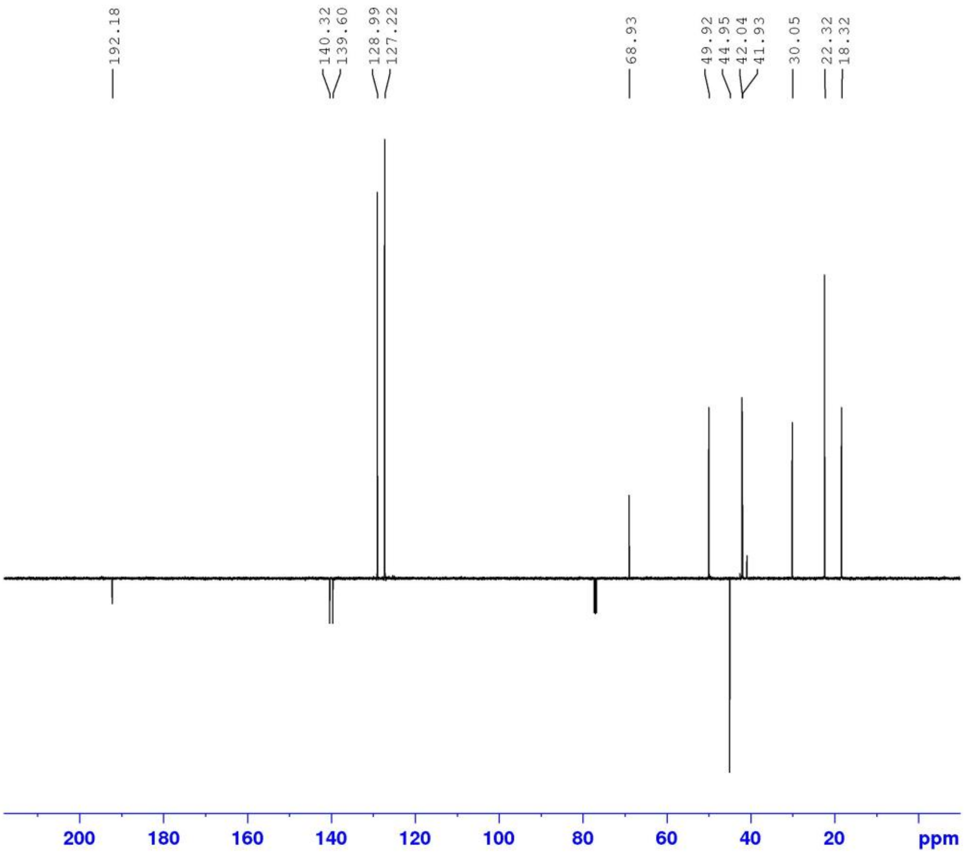

Current Data Parameters
NAME compound 4 b
EXPNO 2
PROCNO 1

F2 - Acquisition Parameters
Date_ 20161011
Time 17.26
INSTRUM spect
PROBHD 5 mm PABBO BB-
PULPROG jmod
TD 65536
SOLVENT CDCl3
NS 256
DS 4
SWH 29761.904 Hz
FIDRES 0.454131 Hz
AQ 1.1010048 sec
RG 198.17
DW 16.800 usec
DE 6.50 usec
TE 298.0 K
CNST2 145.0000000
CNST11 1.0000000
D1 2.00000000 sec
D20 0.00689655 sec
TD0 1

======== CHANNEL f1 ========
SFO1 125.7703643 MHz
NUC1 13C
P1 8.00 usec
P2 16.00 usec
PLW1 53.00000000 W

======== CHANNEL f2 ========
SFO2 500.1320005 MHz
NUC2 1H
CPDPRG[2 waltz16
PCPD2 80.00 usec
PLW2 28.00000000 W
PLW12 0.42017001 W

F2 - Processing parameters
SI 32768
SF 125.7578040 MHz
WDW EM
SSB 0
LB 1.00 Hz
GB 0
PC 1.40

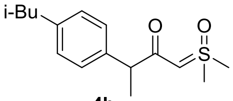

4b

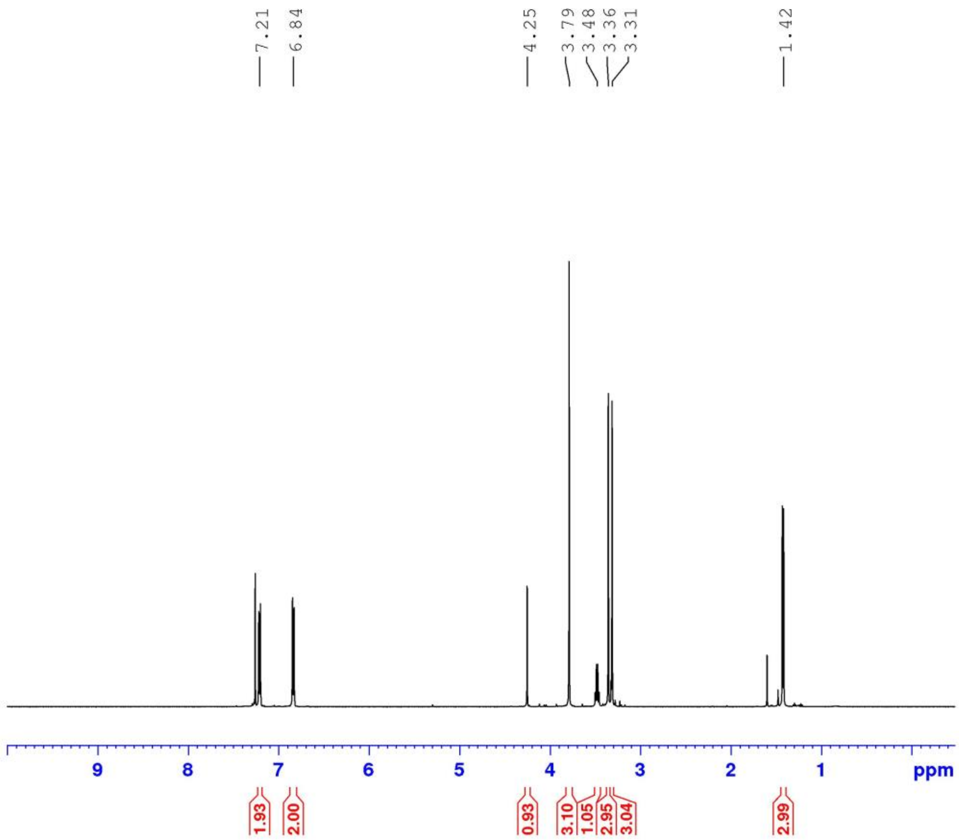


```
Current Data Parameters
NAME          compound 4c
EXPNO                   2
PROCNO                  1

F2 - Acquisition Parameters
Date_            20180913
Time                17.34 h
INSTRUM             spect
PROBHD   Z800701_0108 (
PULPROG              zg30
TD                  65536
SOLVENT             CDCl3
NS                     16
DS                      2
SWH             10000.000 Hz
FIDRES           0.305176 Hz
AQ              3.2767999 sec
RG                 198.17
DW                 50.000 usec
DE                   6.50 usec
TE                  298.0 K
D1             1.00000000 sec
TD0                     1
SFO1          500.1330883 MHz
NUC1                   1H
P1                  10.00 usec
PLW1          26.00000000 W

F2 - Processing parameters
SI                  65536
SF            500.1300125 MHz
WDW                    EM
SSB      0
LB                   0.30 Hz
GB       0
PC                   1.00
```

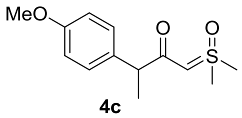

4c

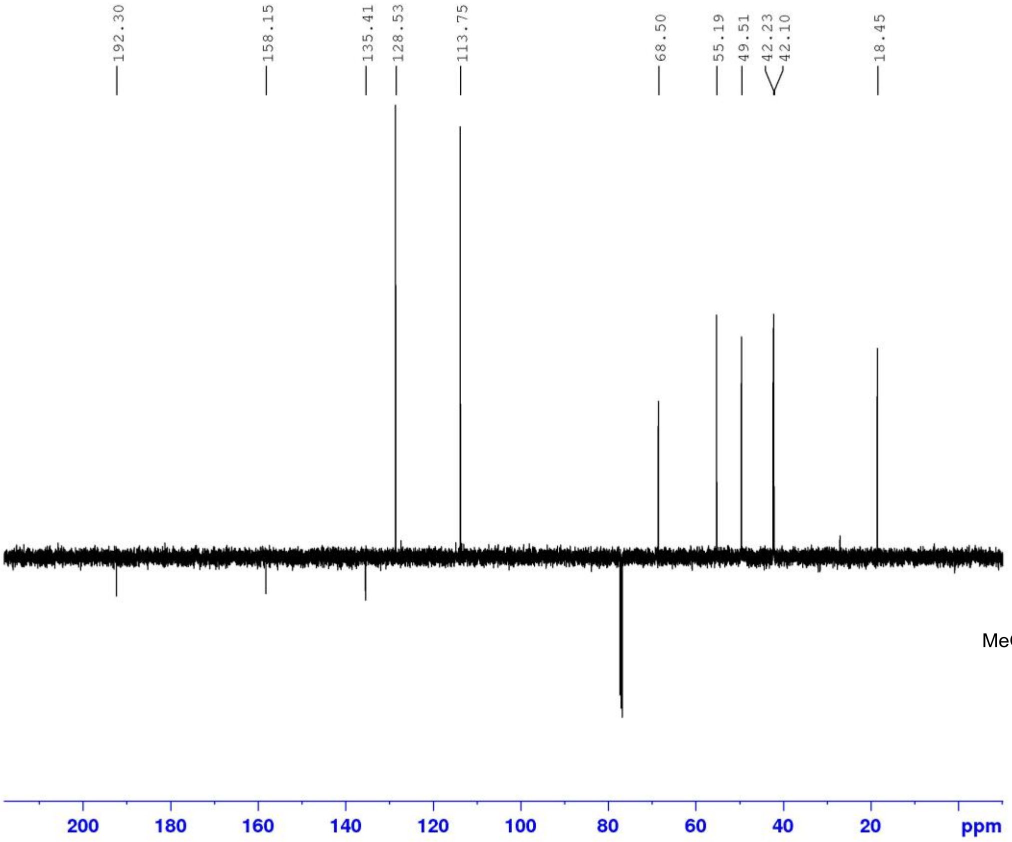


```
Current Data Parameters
NAME          compound 4c
EXPNO                   3
PROCNO                  1

F2 - Acquisition Parameters
Date_            20180913
Time                17.47 h
INSTRUM             spect
PROBHD   Z800701_0108 (
PULPROG              jmod
TD                  65536
SOLVENT             CDCl3
NS                     74
DS                      4
SWH             29761.904 Hz
FIDRES           0.908261 Hz
AQ              1.1010048 sec
RG                 198.17
DW                 16.800 usec
DE                   6.50 usec
TE                  298.0 K
CNST2         145.0000000
CNST11          1.0000000
D1             2.00000000 sec
D20            0.00689655 sec
TD0                     1
SFO1          125.7703643 MHz
NUC1                  13C
P1                   8.00 usec
P2                  16.00 usec
PLW1          53.00000000 W
SFO2          500.1320005 MHz
NUC2                   1H
CPDPRG[2          waltz16
PCPD2               80.00 usec
PLW2          28.00000000 W
PLW12          0.42017001 W

F2 - Processing parameters
SI                  32768
SF            125.7577954 MHz
WDW                    EM
SSB      0
LB                   1.00 Hz
GB       0
PC                   1.40
```

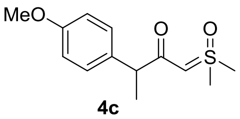

4c

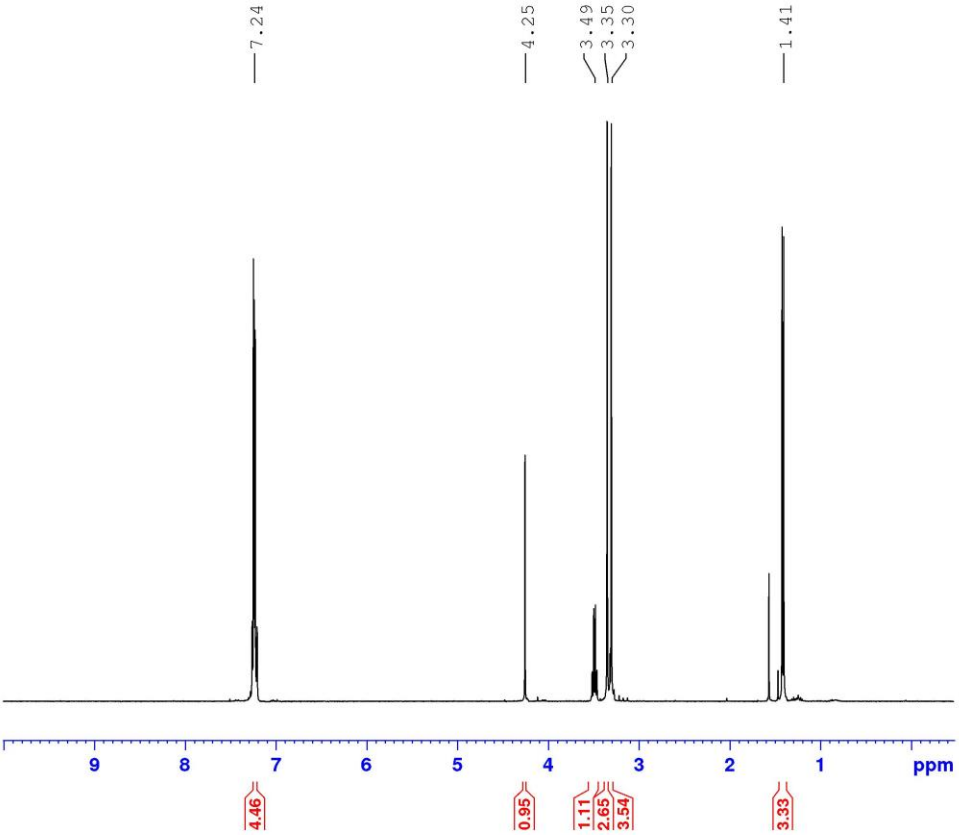
7.24
4.25
3.49
3.35
3.30
1.41
9
8
7
6
5
4
3
2
1
ppm
4.46
0.95
1.11
2.65
3.54
3.33
Current Data Parameters
NAME compound 4d
EXPNO 1
PROCNO 1
F2 - Acquisition Parameters
Date_ 20180904
Time 10.51
INSTRUM spect
PROBHD 5 mm QNP 1H/13
PULPROG zg30
TD 65536
SOLVENT CDCl3
NS 16
DS 2
SWH 8278.146 Hz
FIDRES 0.126314 Hz
AQ 3.9583745 sec
RG 362
DW 60.400 usec
DE 6.50 usec
TE 298.0 K
D1 1.00000000 sec
TD0 1
======== CHANNEL f1 ========
NUC1 1H
P1 10.00 usec
PL1 1.00 dB
SFO1 400.1324710 MHz
F2 - Processing parameters
SI 32768
SF 400.1300150 MHz
WDW EM
SSB 0
LB 0.30 Hz
GB 0
PC 1.00

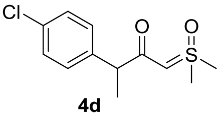
Cl
O
O
S

4d

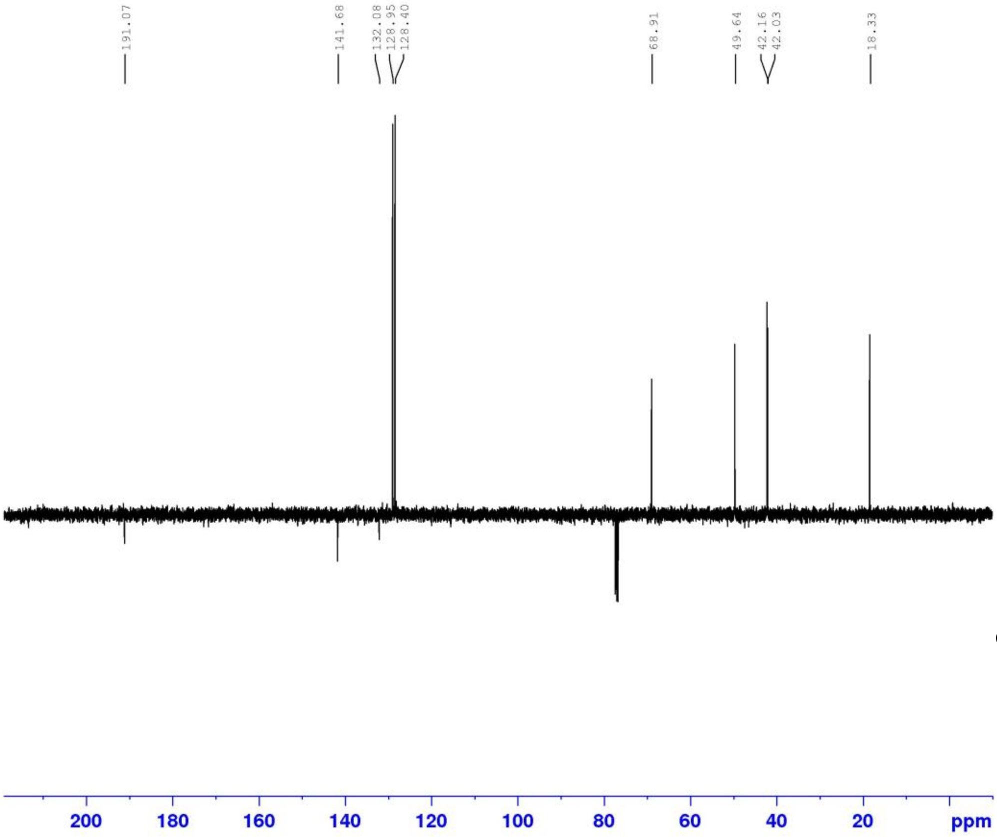
191.07
141.68
132.08
128.95
128.40
68.91
49.64
42.16
42.03
18.33
200
180
160
140
120
100
80
60
40
20
ppm
Current Data Parameters
NAME compound 4d
EXPNO 2
PROCNO 1
F2 - Acquisition Parameters
Date_ 20180808
Time 17.31
INSTRUM spect
PROBHD 5 mm QNP 1H/13
PULPROG jmod
TD 65536
SOLVENT CDCl3
NS 71
DS 4
SWH 23980.814 Hz
FIDRES 0.365918 Hz
AQ 1.3664256 sec
RG 16384
DW 20.850 usec
DE 6.50 usec
TE 298.0 K
CNST2 145.0000000
CNST11 1.0000000
D1 2.00000000 sec
D20 0.00689655 sec
TD0 1
======== CHANNEL f1 ========
NUC1 13C
P1 8.00 usec
P2 16.00 usec
PL1 0 dB
SFO1 100.6228298 MHz
======== CHANNEL f2 ========
CPDPRG[2 waltz16
NUC2 1H
PCPD2 80.00 usec
PL2 1.00 dB
PL12 19.06 dB
SFO2 400.1316005 MHz
F2 - Processing parameters
SI 32768
SF 100.6127757 MHz
WDW EM
SSB 0
LB 1.00 Hz
GB 0
PC 1.40

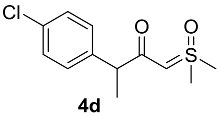
Cl
O
O
S

4d

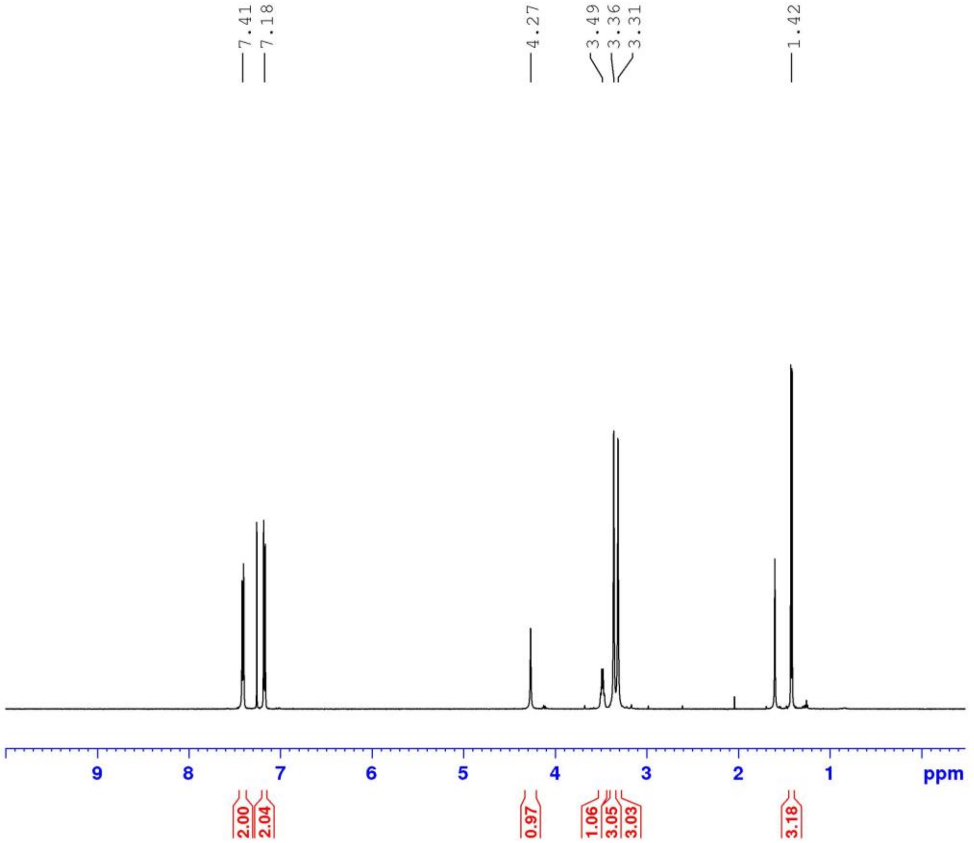


```
Current Data Parameters
NAME          compound 4e
EXPNO                   3
PROCNO                  1

F2 - Acquisition Parameters
Date_            20180316
Time                15.42 h
INSTRUM             spect
PROBHD    Z800701_0108 (
PULPROG              zg30
TD                  65536
SOLVENT             CDCl3
NS                     16
DS                      2
SWH             10000.000 Hz
FIDRES           0.305176 Hz
AQ              3.2767999 sec
RG                 198.17
DW                 50.000 usec
DE                   6.50 usec
TE                  298.0 K
D1             1.00000000 sec
TD0                     1
SFO1          500.1330883 MHz
NUC1                   1H
P1                  10.00 usec
PLW1          26.00000000 W

F2 - Processing parameters
SI                  65536
SF            500.1300127 MHz
WDW                    EM
SSB      0
LB                   0.30 Hz
GB       0
PC                   1.00
```

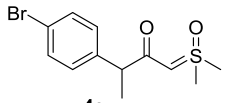

4e

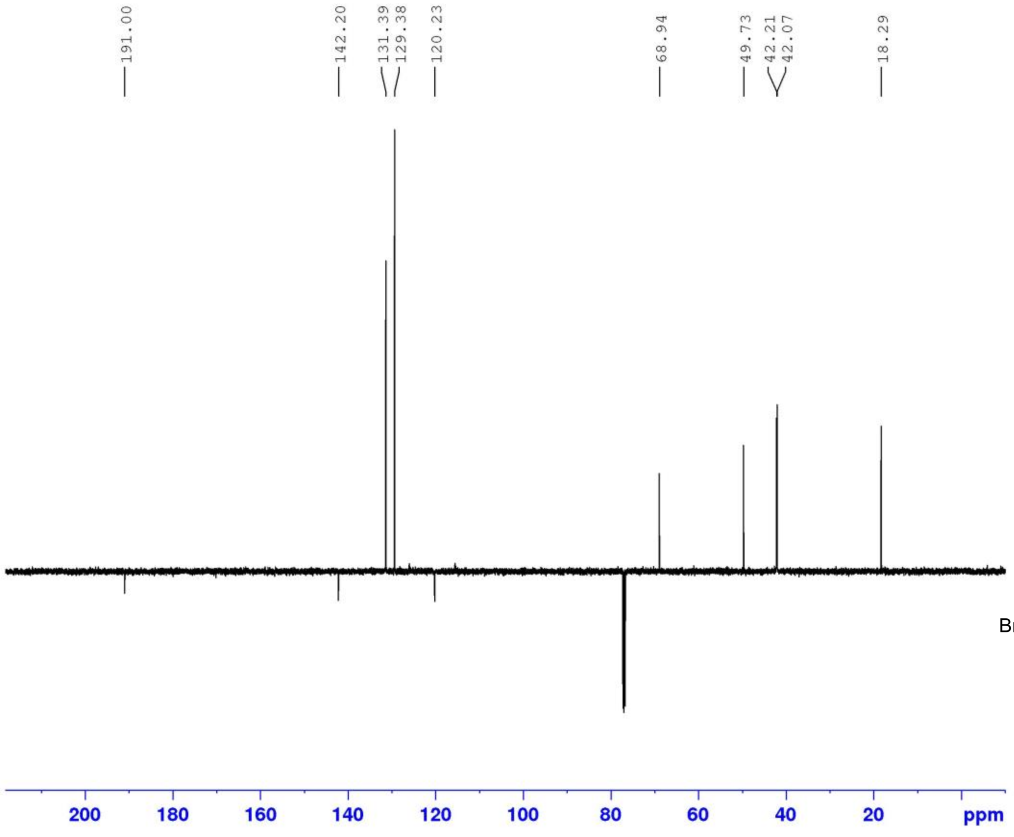


```
Current Data Parameters
NAME          compound 4e
EXPNO                   2
PROCNO                  1

F2 - Acquisition Parameters
Date_            20180315
Time                17.37 h
INSTRUM             spect
PROBHD    Z800701_0108 (
PULPROG              jmod
TD                  65536
SOLVENT             CDCl3
NS                    512
DS                      4
SWH             29761.904 Hz
FIDRES           0.908261 Hz
AQ              1.1010048 sec
RG                 198.17
DW                 16.800 usec
DE                   6.50 usec
TE                  298.0 K
CNST2         145.0000000
CNST11          1.0000000
D1             2.00000000 sec
D20            0.00689655 sec
TD0                     1
SFO1          125.7703643 MHz
NUC1                  13C
P1                   8.00 usec
P2                  16.00 usec
PLW1          53.00000000 W
SFO2          500.1320005 MHz
NUC2                   1H
CPDPRG[2          waltz16
PCPD2               80.00 usec
PLW2          28.00000000 W
PLW12          0.42017001 W

F2 - Processing parameters
SI                  32768
SF            125.7577951 MHz
WDW                    EM
SSB      0
LB                   1.00 Hz
GB       0
PC                   1.40
```

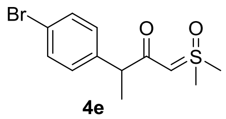

4e

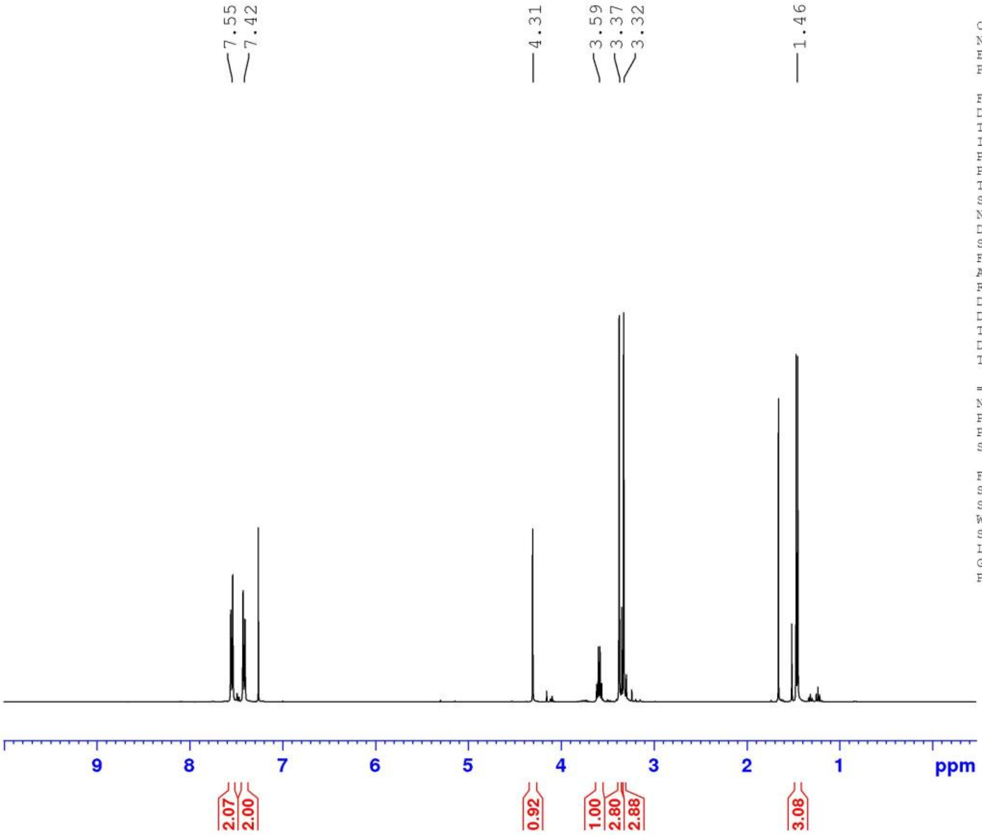


```
Current Data Parameters
NAME          compound 4f
EXPNO                   1
PROCNO                  1

F2 - Acquisition Parameters
Date_            20181204
Time                18.40
INSTRUM             spect
PROBHD   5 mm QNP 1H/13
PULPROG              zg30
TD                  65536
SOLVENT             CDCl3
NS                     16
DS                      2
SWH              8278.146 Hz
FIDRES           0.126314 Hz
AQ              3.9583745 sec
RG                    256
DW                 60.400 usec
DE                   6.50 usec
TE                  291.8 K
D1             1.00000000 sec
TD0                     1

======== CHANNEL f1 ========
NUC1                   1H
P1                  10.00 usec
PL1                  1.00 dB
SFO1          400.1324710 MHz

F2 - Processing parameters
SI                  32768
SF            400.1300095 MHz
WDW                    EM
SSB       0
LB                   0.30 Hz
GB        0
PC                   1.00
```

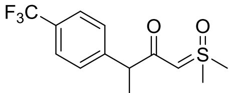

4f

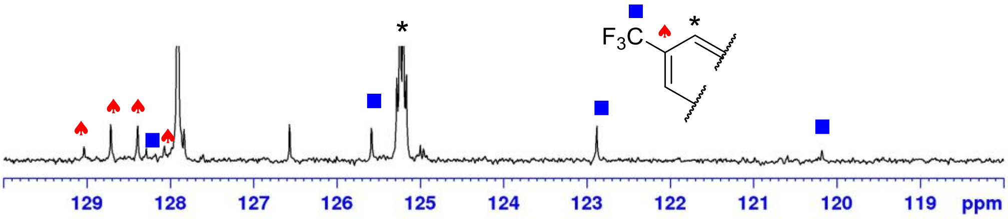


```
Current Data Parameters
NAME          compound 4f
EXPNO                   4
PROCNO                  1

F2 - Acquisition Parameters
Date_            20181204
Time                20.03
INSTRUM             spect
PROBHD   5 mm QNP 1H/13
PULPROG            zgpg30
TD                  65536
SOLVENT             CDCl3
NS                   1024
DS                      4
SWH             23980.814 Hz
FIDRES           0.365918 Hz
AQ              1.3664256 sec
RG                 1625.5
DW                 20.850 usec
DE                   6.50 usec
TE                  292.2 K
D1             2.00000000 sec
D11            0.03000000 sec
TD0                     1

======== CHANNEL f1 ========
NUC1                  13C
P1                   8.00 usec
PL1       0 dB
SFO1          100.6228298 MHz

======== CHANNEL f2 ========
CPDPRG[2          waltz16
NUC2                   1H
PCPD2               80.00 usec
PL2                  1.00 dB
PL12                19.06 dB
PL13                20.00 dB
SFO2          400.1316005 MHz

F2 - Processing parameters
SI                  32768
SF            100.6127770 MHz
WDW                    EM
SSB       0
LB                   1.00 Hz
GB        0
PC                   1.40
```

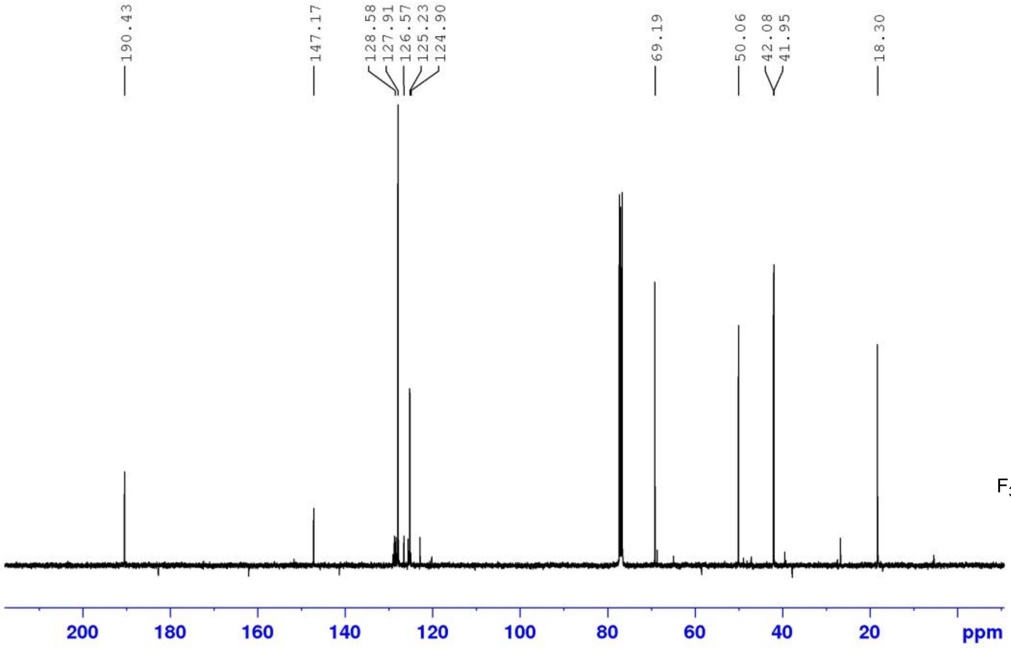

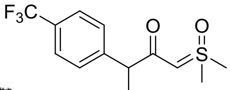

4f

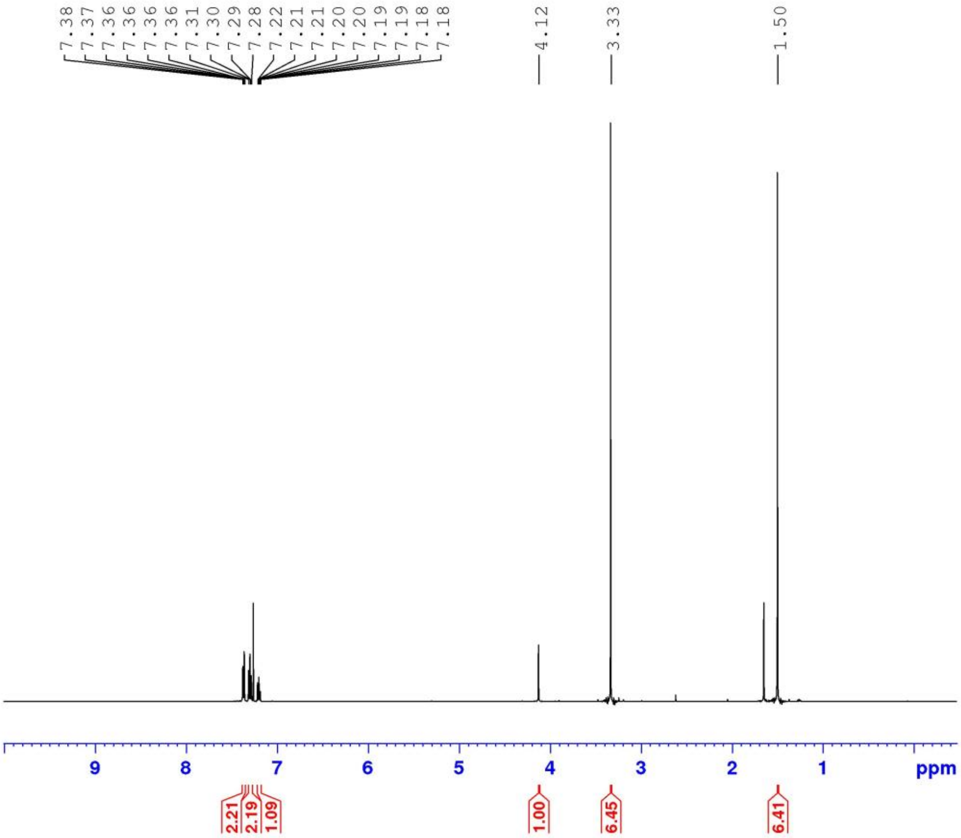


```
Current Data Parameters
NAME          compound 4g
EXPNO                   1
PROCNO                  1

F2 - Acquisition Parameters
Date_            20161025
Time                12.46
INSTRUM             spect
PROBHD   5 mm PABBO BB-
PULPROG              zg30
TD                  65536
SOLVENT             CDCl3
NS                     16
DS                      2
SWH             10000.000 Hz
FIDRES           0.152588 Hz
AQ              3.2767999 sec
RG                 198.17
DW                 50.000 usec
DE                   6.50 usec
TE                  290.5 K
D1             1.00000000 sec
TD0                     1

======== CHANNEL f1 ========
SFO1          500.1330885 MHz
NUC1                   1H
P1                  10.00 usec
PLW1          26.00000000 W

F2 - Processing parameters
SI                  65536
SF            500.1300122 MHz
WDW                    EM
SSB      0
LB                   0.30 Hz
GB       0
PC                   1.00
```

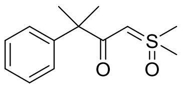

4g

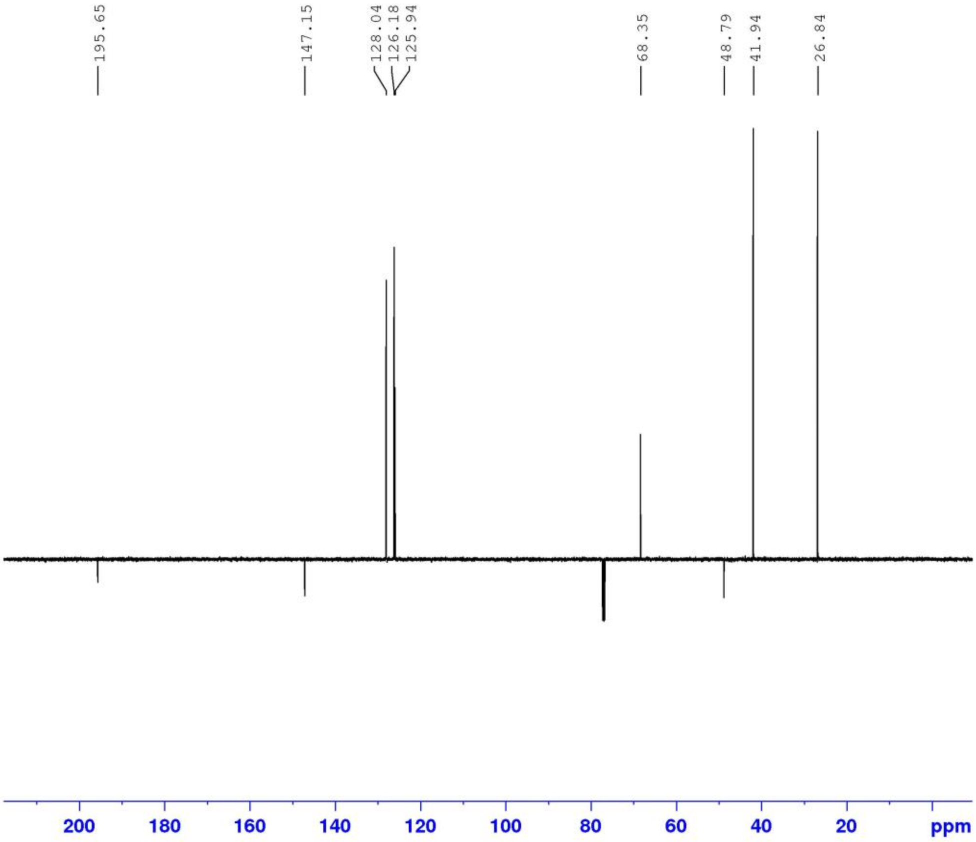


```
Current Data Parameters
NAME          compound 4g
EXPNO                   2
PROCNO                  1

F2 - Acquisition Parameters
Date_            20161025
Time                13.48
INSTRUM             spect
PROBHD   5 mm PABBO BB-
PULPROG              jmod
TD                  65536
SOLVENT             CDCl3
NS                    256
DS                      4
SWH             29761.904 Hz
FIDRES           0.454131 Hz
AQ              1.1010048 sec
RG                 198.17
DW                 16.800 usec
DE                   6.50 usec
TE                  292.0 K
CNST2         145.0000000
CNST11          1.0000000
D1             2.00000000 sec
D20            0.00689655 sec
TD0                     1

======== CHANNEL f1 ========
SFO1          125.7703643 MHz
NUC1                  13C
P1                   8.00 usec
P2                  16.00 usec
PLW1          53.00000000 W

======== CHANNEL f2 ========
SFO2          500.1320005 MHz
NUC2                   1H
CPDPRG[2          waltz16
PCPD2               80.00 usec
PLW2          28.00000000 W
PLW12          0.42017001 W

F2 - Processing parameters
SI                  32768
SF            125.7578022 MHz
WDW                    EM
SSB      0
LB                   1.00 Hz
GB       0
PC                   1.40
```

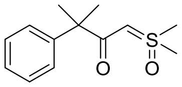

4g

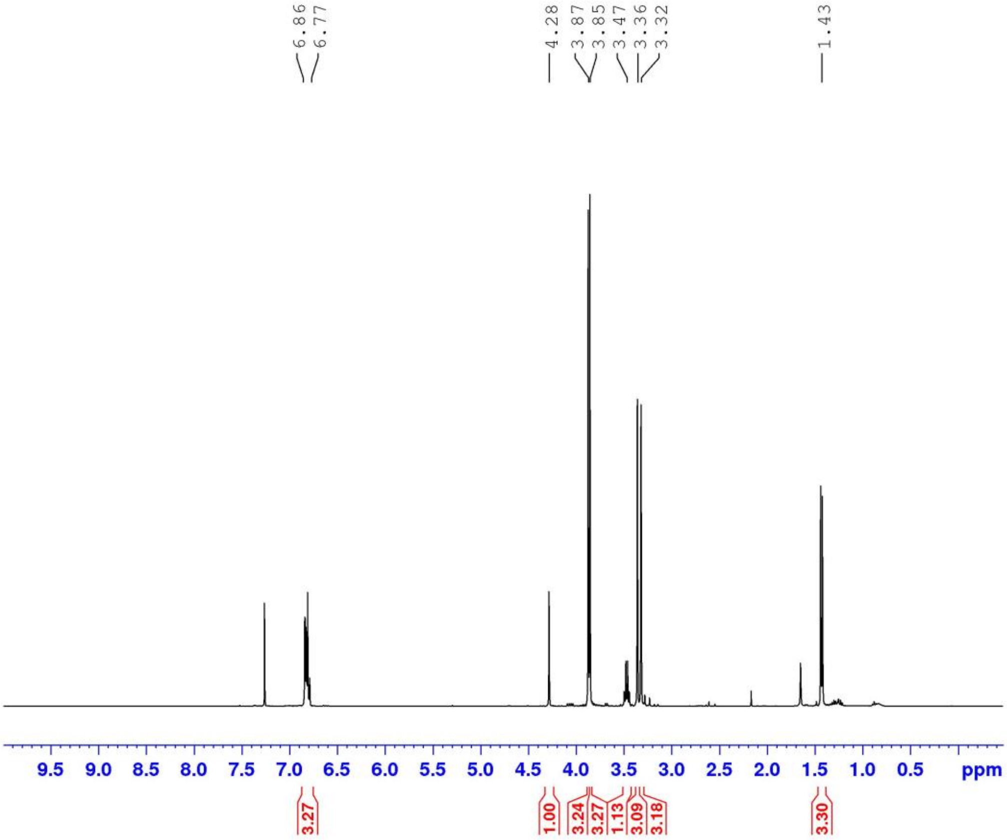

Current Data Parameters
NAME compound 4h
EXPNO 1
PROCNO 1

F2 - Acquisition Parameters
Date_ 20190618
Time 15.05
INSTRUM spect
PROBHD 5 mm QNP 1H/1
PULPROG zg30
TD 65536
SOLVENT CDCl3
NS 16
DS 2
SWH 8278.146 Hz
FIDRES 0.126314 Hz
AQ 3.9583745 sec
RG 256
DW 60.400 usec
DE 6.50 usec
TE 298.0 K
D1 1.00000000 sec
TD0 1

======== CHANNEL f1 ========
NUC1 1H
P1 10.00 usec
PL1 0.50 dB
SFO1 400.1324710 MHz

F2 - Processing parameters
SI 32768
SF 400.1300103 MHz
WDW EM
SSB 0
LB 0.30 Hz
GB 0
PC 1.00

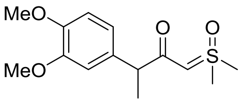

4h

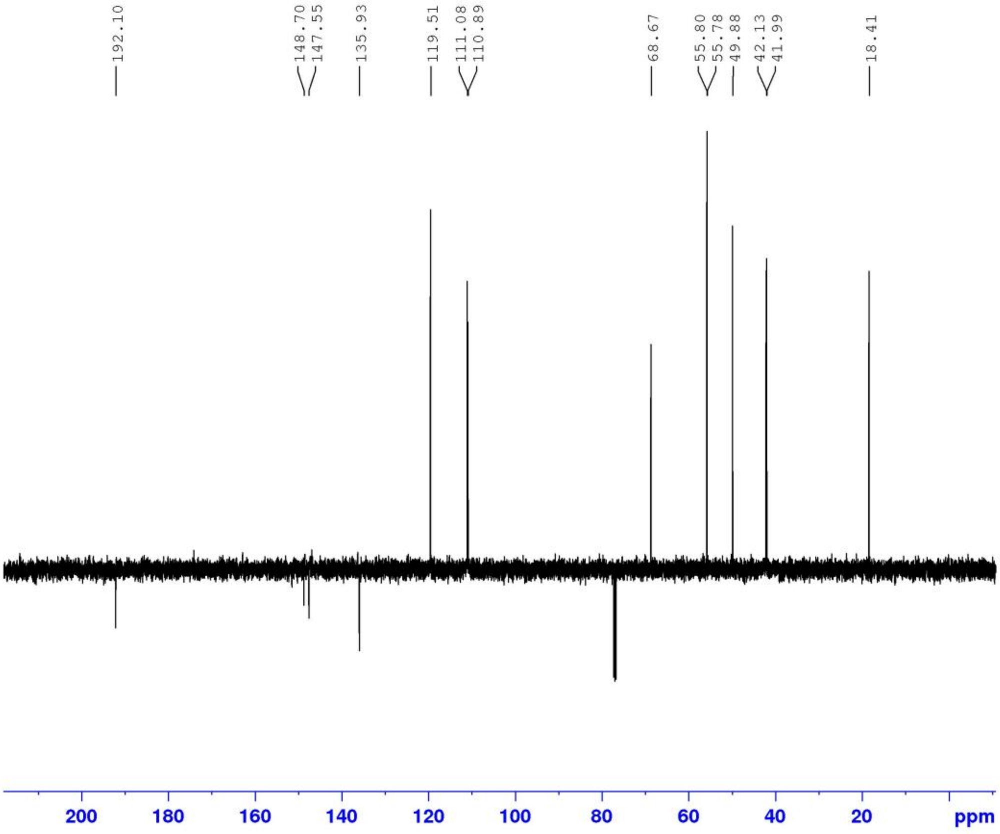

Current Data Parameters
NAME compound 4 h
EXPNO 1
PROCNO 1

F2 - Acquisition Parameters
Date_ 20190727
Time 16.30 h
INSTRUM spect
PROBHD Z800701_0108 (
PULPROG jmod
TD 65536
SOLVENT CDCl3
NS 32
DS 4
SWH 29761.904 Hz
FIDRES 0.908261 Hz
AQ 1.1010048 sec
RG 198.17
DW 16.800 usec
DE 6.50 usec
TE 298.4 K
CNST2 145.0000000
CNST11 1.0000000
D1 2.00000000 sec
D20 0.00689655 sec
TD0 1
SFO1 125.7703643 MHz
NUC1 13C
P1 8.00 usec
P2 16.00 usec
PLW1 53.00000000 W
SFO2 500.1320005 MHz
NUC2 1H
CPDPRG[2 waltz65
PCPD2 80.00 usec
PLW2 28.00000000 W
PLW12 0.42017001 W

F2 - Processing parameters
SI 32768
SF 125.7578000 MHz
WDW EM
SSB 0
LB 1.00 Hz
GB 0
PC 1.40

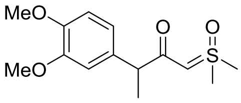

4h

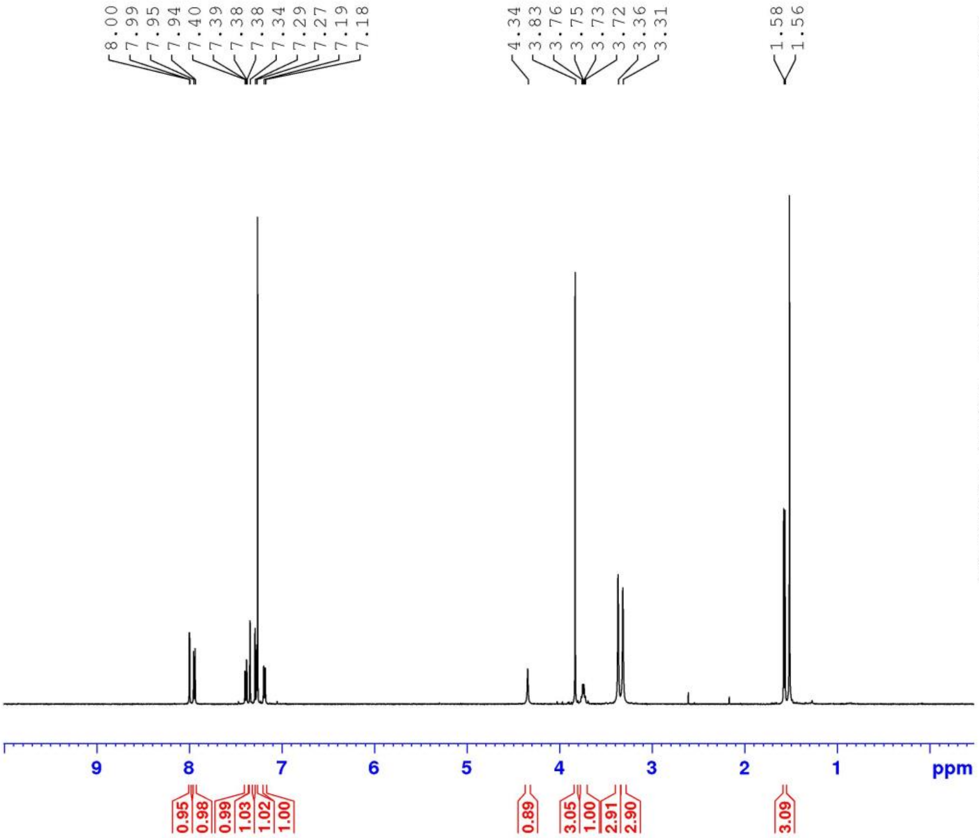


```
Current Data Parameters
NAME          compound 4i
EXPNO                   1
PROCNO                  1

F2 - Acquisition Parameters
Date_            20170425
Time                 9.07
INSTRUM             spect
PROBHD   5 mm PABBO BB-
PULPROG              zg30
TD                  65536
SOLVENT             CDCl3
NS                     16
DS                      2
SWH             10000.000 Hz
FIDRES           0.152588 Hz
AQ              3.2767999 sec
RG                 198.17
DW                 50.000 usec
DE                   6.50 usec
TE                  313.2 K
D1             1.00000000 sec
TD0                     1

======== CHANNEL f1 ========
SFO1          500.1330885 MHz
NUC1                   1H
P1                  10.00 usec
PLW1          26.00000000 W

F2 - Processing parameters
SI                  65536
SF            500.1300119 MHz
WDW                    EM
SSB      0
LB                   0.30 Hz
GB       0
PC                   1.00
```

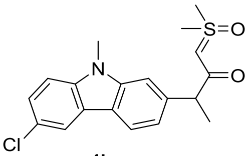

4i

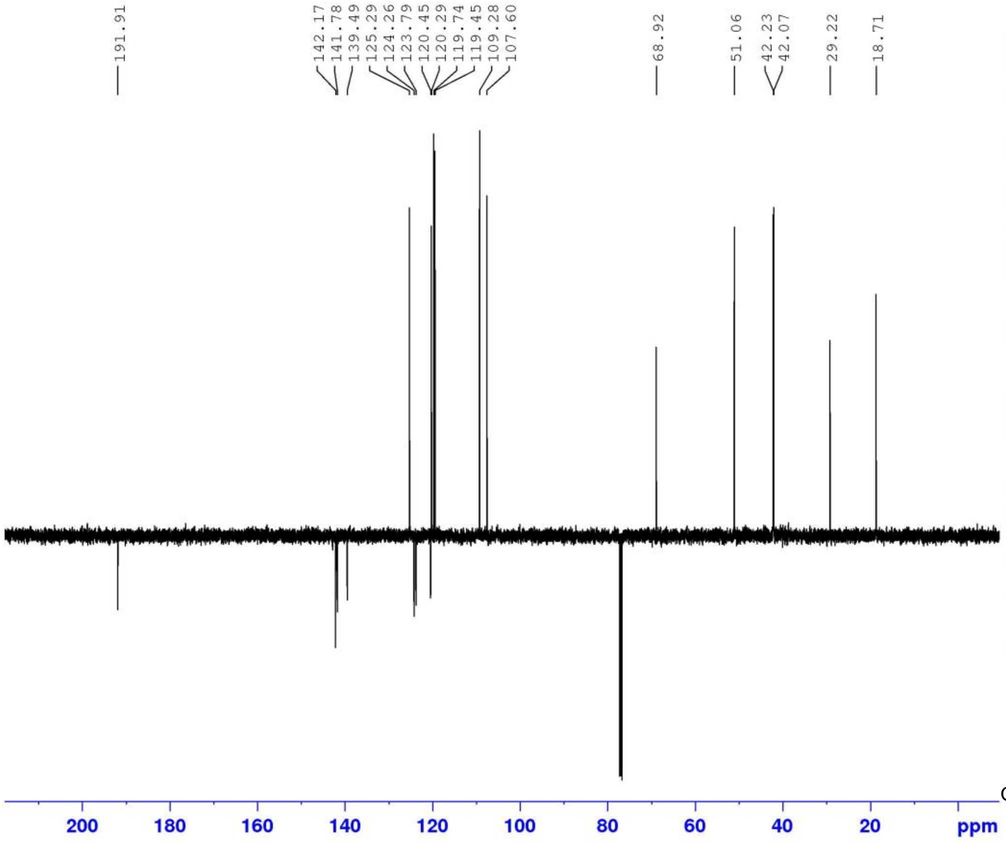


```
Current Data Parameters
NAME          compound 4i
EXPNO                   2
PROCNO                  1

F2 - Acquisition Parameters
Date_            20170501
Time                12.21
INSTRUM             spect
PROBHD   5 mm PABBO BB-
PULPROG              jmod
TD                  65536
SOLVENT             CDCl3
NS                    512
DS                      4
SWH             29761.904 Hz
FIDRES           0.454131 Hz
AQ              1.1010048 sec
RG                 198.17
DW                 16.800 usec
DE                   6.50 usec
TE                  298.0 K
CNST2         145.0000000
CNST11          1.0000000
D1             2.00000000 sec
D20            0.00689655 sec
TD0                     1

======== CHANNEL f1 ========
SFO1          125.7703643 MHz
NUC1                  13C
P1                   8.00 usec
P2                  16.00 usec
PLW1          53.00000000 W

======== CHANNEL f2 ========
SFO2          500.1320005 MHz
NUC2                   1H
CPDPRG[2          waltz16
PCPD2               80.00 usec
PLW2          28.00000000 W
PLW12          0.42017001 W

F2 - Processing parameters
SI                  32768
SF            125.7577931 MHz
WDW                    EM
SSB      0
LB                   1.00 Hz
GB       0
PC                   1.40
```

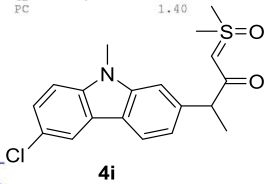

4i

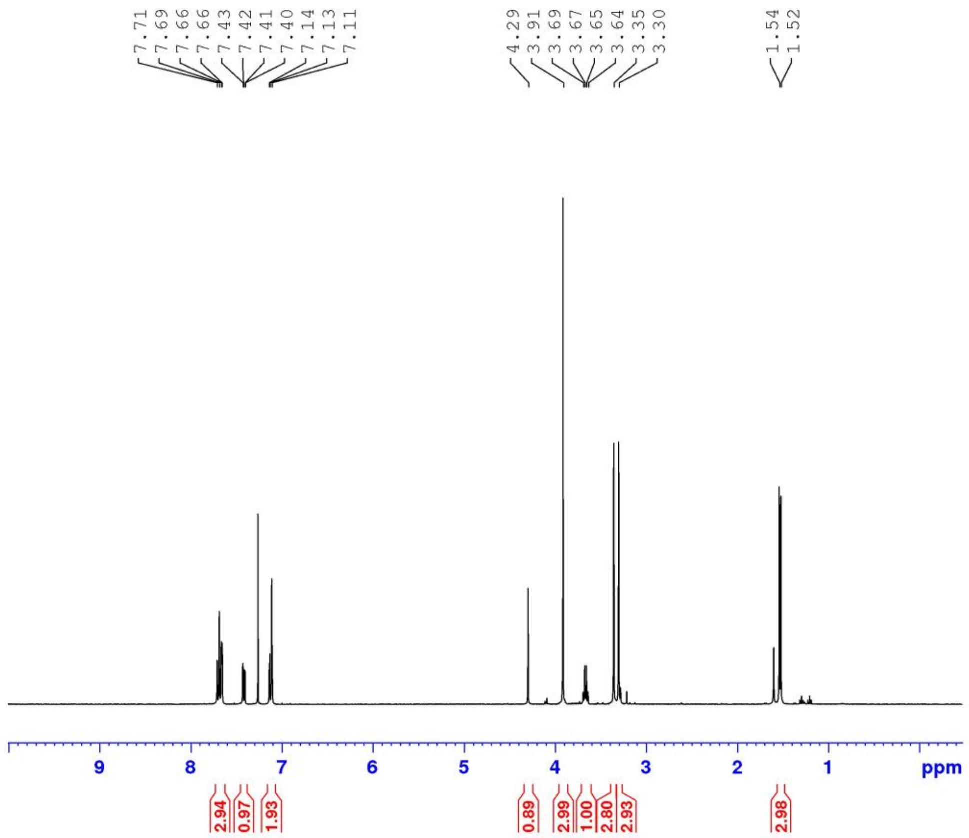


```
Current Data Parameters
NAME          compound 4 j
EXPNO                    1
PROCNO                   1

F2 - Acquisition Parameters
Date_             20180828
Time                 15.28
INSTRUM              spect
PROBHD   5 mm QNP 1H/13
PULPROG               zg30
TD                   65536
SOLVENT              CDCl3
NS                      16
DS                       2
SWH             8278.146 Hz
FIDRES          0.126314 Hz
AQ             3.9583745 sec
RG                   322.5
DW                  60.400 usec
DE                    6.50 usec
TE                   298.0 K
D1            1.00000000 sec
TD0                      1

======== CHANNEL f1 ========
NUC1                    1H
P1                   10.00 usec
PL1                   1.00 dB
SFO1           400.1324710 MHz

F2 - Processing parameters
SI                   32768
SF             400.1300093 MHz
WDW                     EM
SSB      0
LB                    0.30 Hz
GB       0
PC                    1.00
```

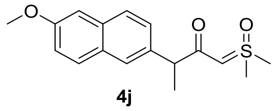

4j

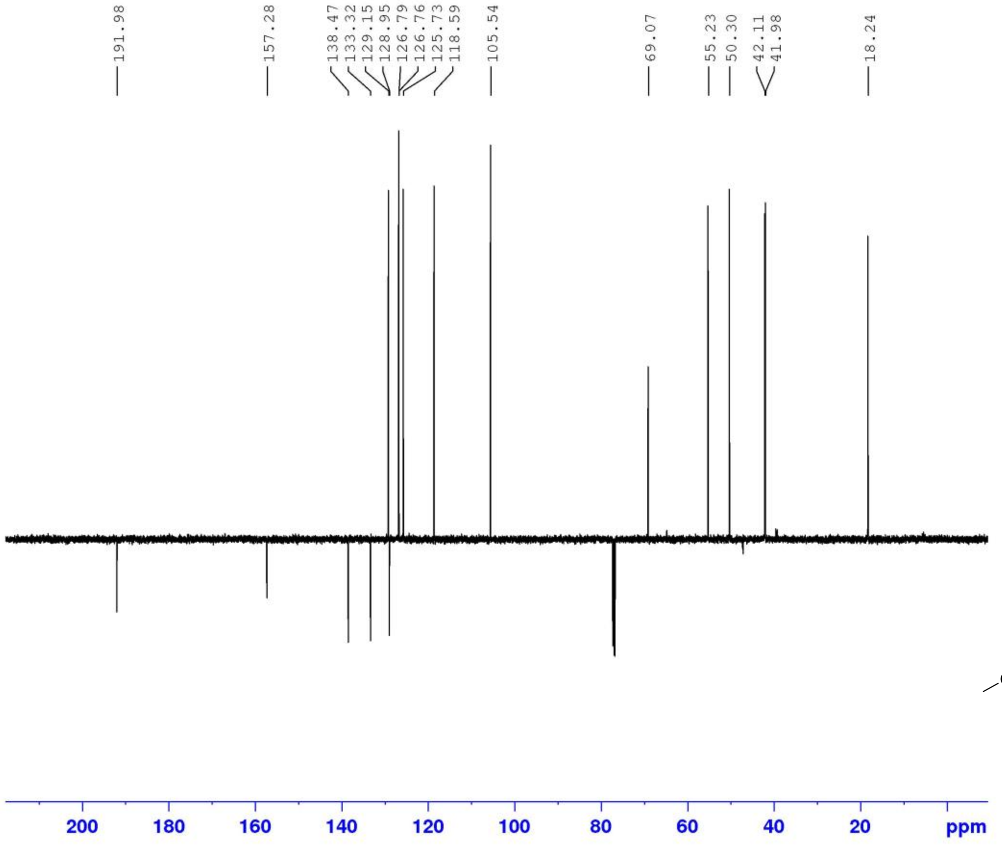


```
Current Data Parameters
NAME          compound 4j
EXPNO                   2
PROCNO                  1

F2 - Acquisition Parameters
Date_            20170707
Time                10.55
INSTRUM             spect
PROBHD   5 mm PABBO BB-
PULPROG              jmod
TD                  65536
SOLVENT             CDCl3
NS                    256
DS                      4
SWH             29761.904 Hz
FIDRES           0.454131 Hz
AQ              1.1010048 sec
RG                 198.17
DW                 16.800 usec
DE                   6.50 usec
TE                  298.0 K
CNST2         145.0000000
CNST11          1.0000000
D1             2.00000000 sec
D20            0.00689655 sec
TD0                     1

======== CHANNEL f1 ========
SFO1           125.7703643 MHz
NUC1                   13C
P1                    8.00 usec
P2                   16.00 usec
PLW1          53.00000000 W

======== CHANNEL f2 ========
SFO2           500.1320005 MHz
NUC2                    1H
CPDPRG[2            waltz16
PCPD2                80.00 usec
PLW2          28.00000000 W
PLW12          0.42017001 W

F2 - Processing parameters
SI                   32768
SF             125.7577991 MHz
WDW                     EM
SSB      0
LB                    1.00 Hz
GB       0
PC                    1.40
```

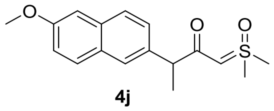

4j

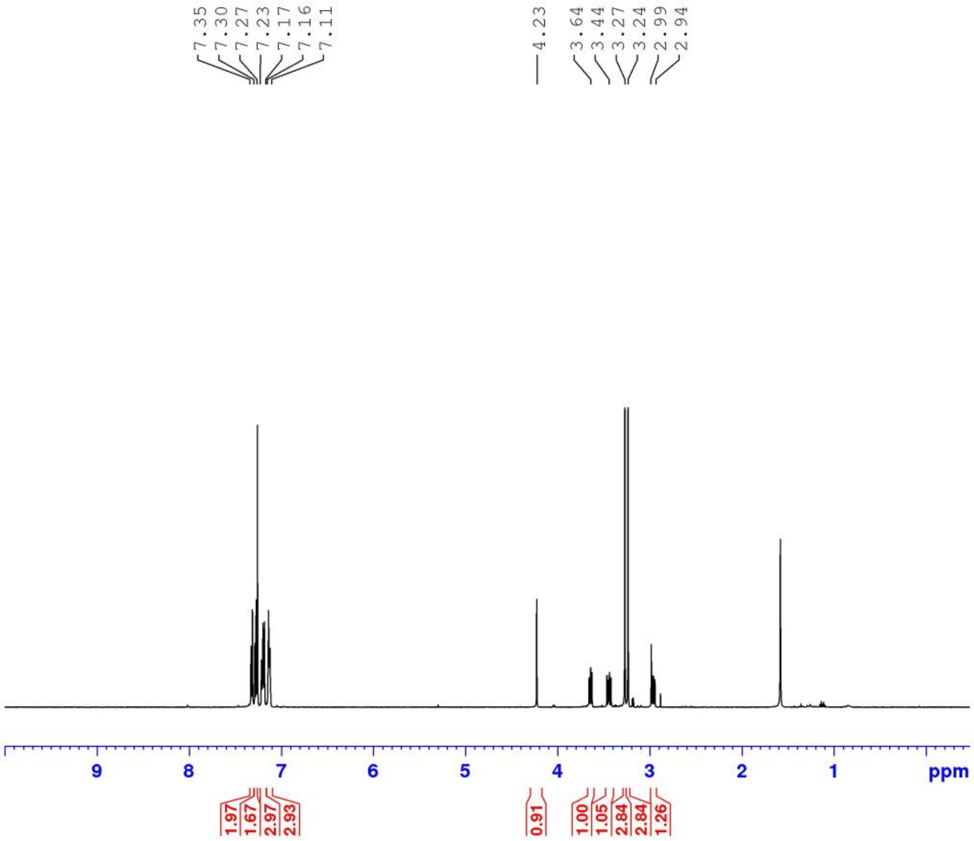


```
Current Data Parameters
NAME          compound 4k
EXPNO                   1
PROCNO                  1

F2 - Acquisition Parameters
Date_            20181004
Time                12.28 h
INSTRUM             spect
PROBHD   Z800701_0108 (
PULPROG              zg30
TD                  65536
SOLVENT             CDCl3
NS                     16
DS                      2
SWH             10000.000 Hz
FIDRES           0.305176 Hz
AQ              3.2767999 sec
RG                 198.17
DW                 50.000 usec
DE                   6.50 usec
TE                  298.8 K
D1             1.00000000 sec
TD0                     1
SFO1          500.1330883 MHz
NUC1                   1H
P1                  10.00 usec
PLW1          26.00000000 W

F2 - Processing parameters
SI                  65536
SF            500.1300126 MHz
WDW                    EM
SSB      0
LB                   0.30 Hz
GB       0
PC                   1.00
```

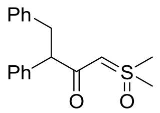

4k

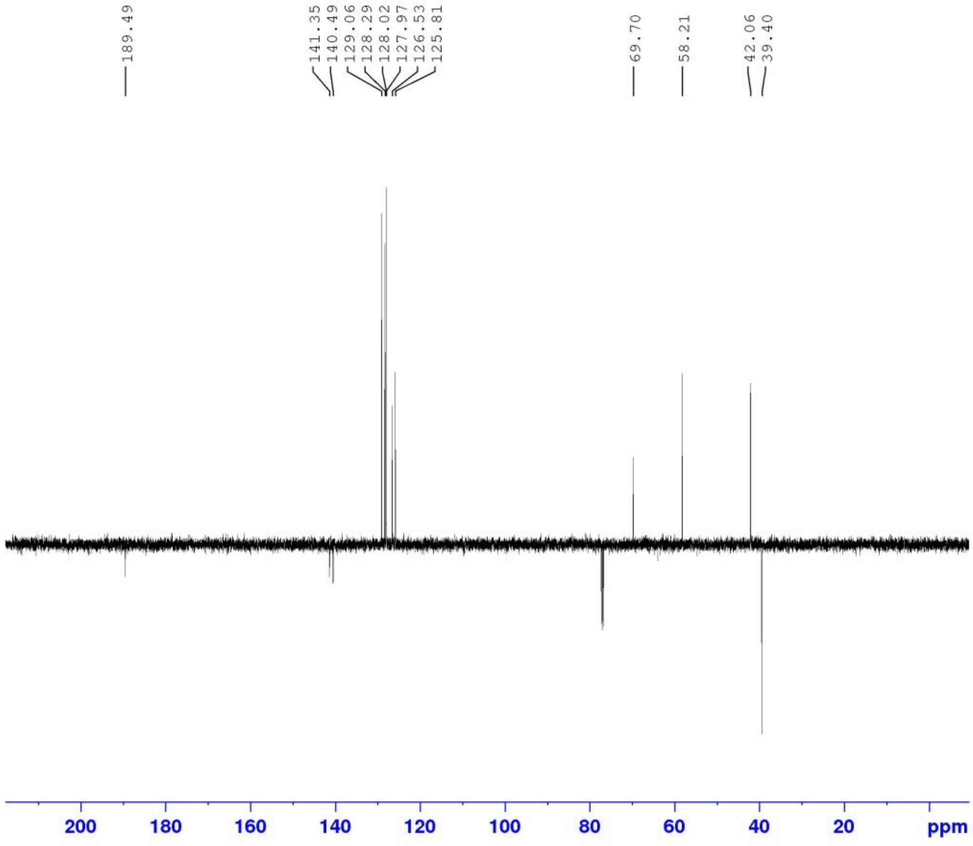


```
Current Data Parameters
NAME          compound 4k
EXPNO                   2
PROCNO                  1

F2 - Acquisition Parameters
Date_            20181004
Time                12.42 h
INSTRUM             spect
PROBHD   Z800701_0108 (
PULPROG              jmod
TD                  65536
SOLVENT             CDCl3
NS                     50
DS                      4
SWH             29761.904 Hz
FIDRES           0.908261 Hz
AQ              1.1010048 sec
RG                 198.17
DW                 16.800 usec
DE                   6.50 usec
TE                  299.9 K
CNST2         145.0000000
CNST11          1.0000000
D1             2.00000000 sec
D20            0.00689655 sec
TD0                     1
SFO1          125.7703643 MHz
NUC1                  13C
P1                   8.00 usec
P2                  16.00 usec
PLW1          53.00000000 W
SFO2          500.1320005 MHz
NUC2                   1H
CPDPRG[2          waltz16
PCPD2               80.00 usec
PLW2          28.00000000 W
PLW12          0.42017001 W

F2 - Processing parameters
SI                  32768
SF            125.7577953 MHz
WDW                    EM
SSB      0
LB                   1.00 Hz
GB       0
PC                   1.40
```

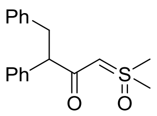

4k

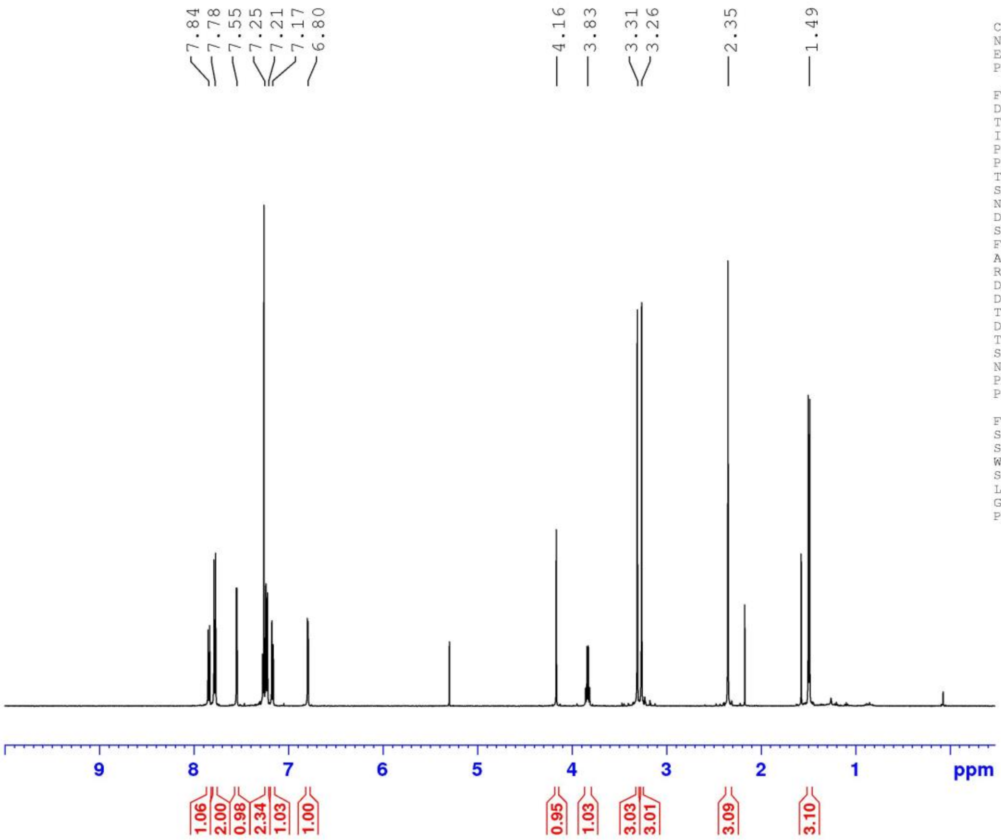


```
Current Data Parameters
NAME          compound 41
EXPNO                   1
PROCNO                  1

F2 - Acquisition Parameters
Date_            20190307
Time                 9.22 h
INSTRUM             spect
PROBHD   Z800701_0108 (
PULPROG              zg30
TD                  65536
SOLVENT             CDCl3
NS                     16
DS                      2
SWH             10000.000 Hz
FIDRES           0.305176 Hz
AQ              3.2767999 sec
RG                 198.17
DW                 50.000 usec
DE                   6.50 usec
TE                  298.0 K
D1             1.00000000 sec
TD0                     1
SFO1          500.1330883 MHz
NUC1                   1H
P1                  10.00 usec
PLW1          26.00000000 W

F2 - Processing parameters
SI                  65536
SF            500.1300120 MHz
WDW                    EM
SSB      0
LB                   0.30 Hz
GB       0
PC                   1.00
```

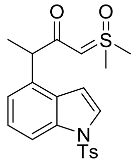

4l

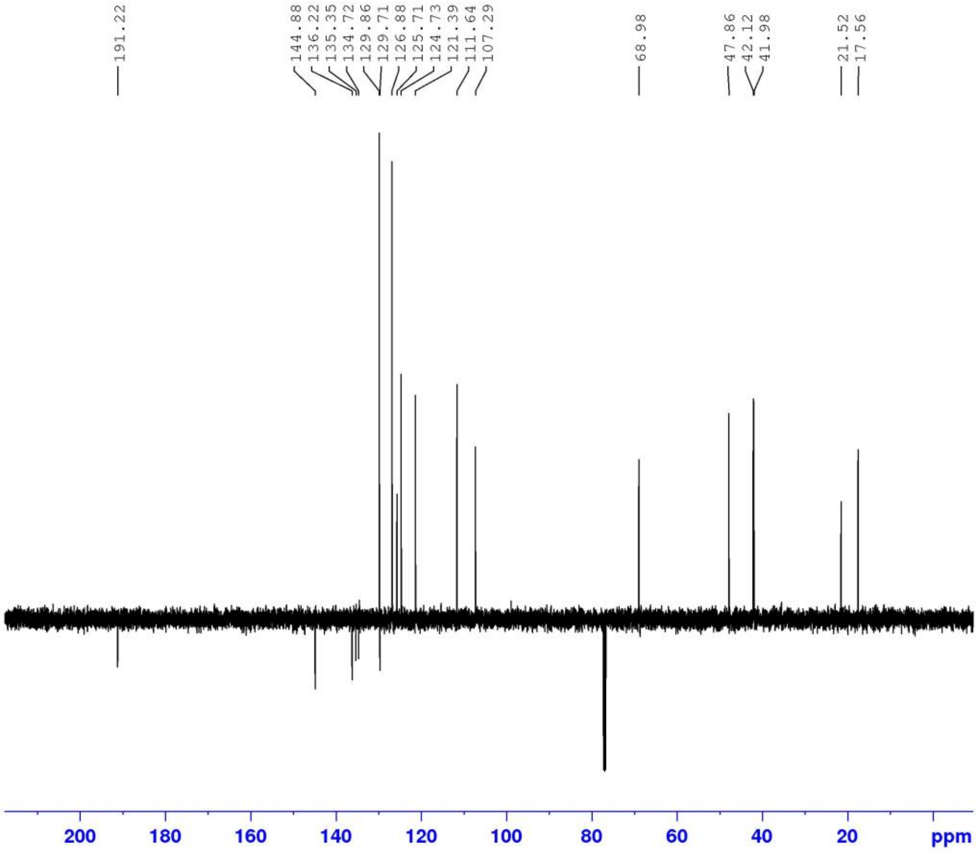


```
Current Data Parameters
NAME          compound 41
EXPNO                   2
PROCNO                  1

F2 - Acquisition Parameters
Date_            20190307
Time                10.42 h
INSTRUM             spect
PROBHD   Z800701_0108 (
PULPROG              jmod
TD                  65536
SOLVENT             CDCl3
NS                     60
DS                      4
SWH             29761.904 Hz
FIDRES           0.908261 Hz
AQ              1.1010048 sec
RG                 198.17
DW                 16.800 usec
DE                   6.50 usec
TE                  298.0 K
CNST2         145.0000000
CNST11          1.0000000
D1             2.00000000 sec
D20            0.00689655 sec
TD0                     1
SFO1          125.7703643 MHz
NUC1                  13C
P1                   8.00 usec
P2                  16.00 usec
PLW1          53.00000000 W
SFO2          500.1320005 MHz
NUC2                   1H
CPDPRG[2          waltz16
PCPD2               80.00 usec
PLW2          28.00000000 W
PLW12          0.42017001 W

F2 - Processing parameters
SI                  32768
SF            125.7577951 MHz
WDW                    EM
SSB      0
LB                   1.00 Hz
GB       0
PC                   1.40
```

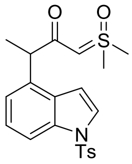

4l

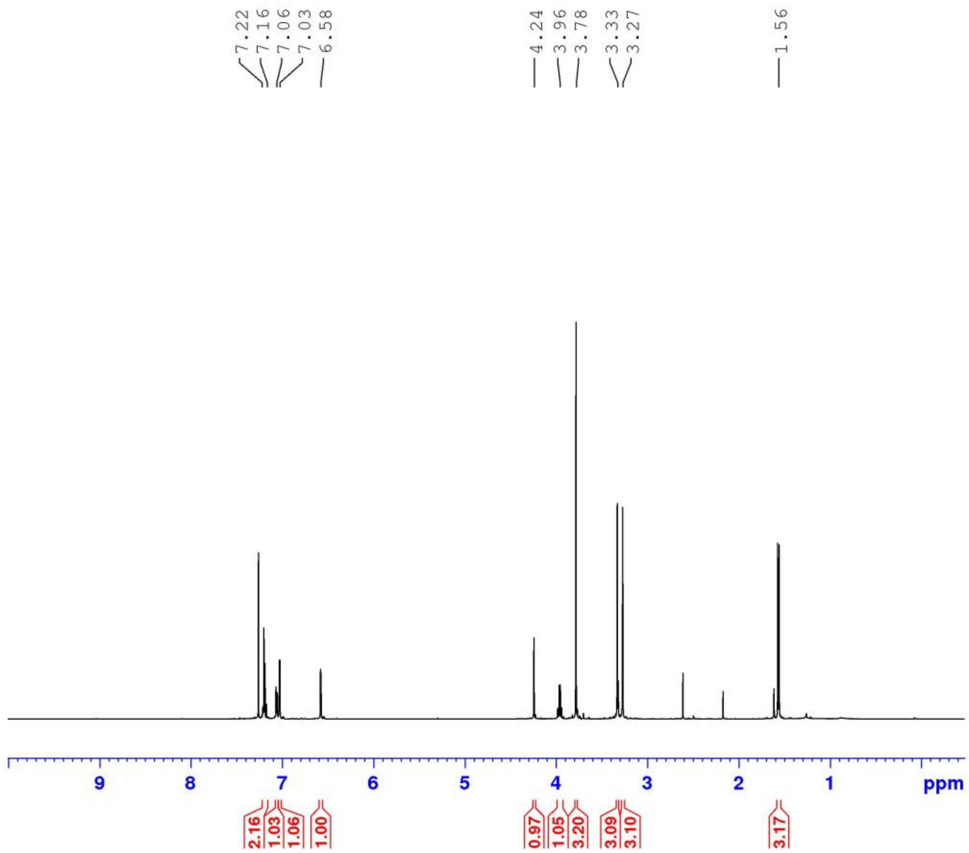

Current Data Parameters
NAME compound 4m
EXPNO 1
PROCNO 1

F2 - Acquisition Parameters
Date_ 20190404
Time 9.23 h
INSTRUM spect
PROBHD Z800701_0108 (
PULPROG zg30
TD 65536
SOLVENT CDCl3
NS 16
DS 2
SWH 10000.000 Hz
FIDRES 0.305176 Hz
AQ 3.2767999 sec
RG 198.17
DW 50.000 usec
DE 6.50 usec
TE 298.0 K
D1 1.00000000 sec
TD0 1
SFO1 500.1330883 MHz
NUC1 1H
P1 10.00 usec
PLW1 26.00000000 W

F2 - Processing parameters
SI 65536
SF 500.1300121 MHz
WDW EM
SSB 0
LB 0.30 Hz
GB 0
PC 1.00

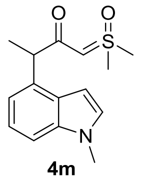

4m

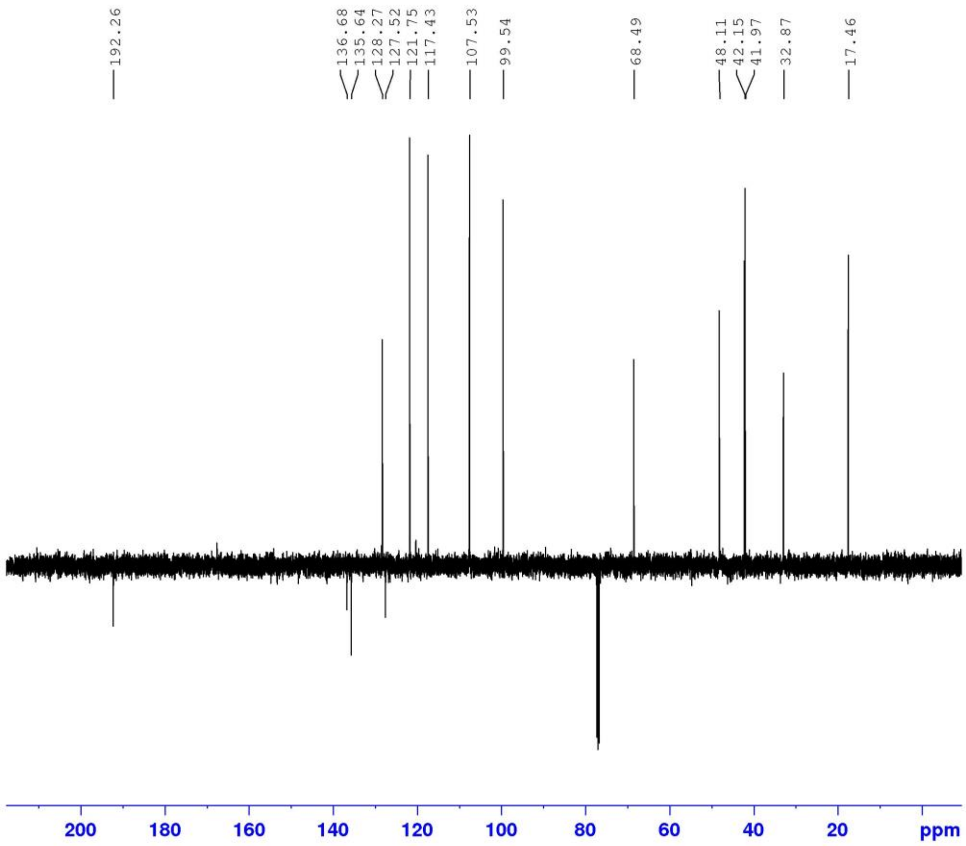

Current Data Parameters
NAME compound 4m
EXPNO 2
PROCNO 1

F2 - Acquisition Parameters
Date_ 20190404
Time 9.36 h
INSTRUM spect
PROBHD Z800701_0108 (
PULPROG jmod
TD 65536
SOLVENT CDCl3
NS 74
DS 4
SWH 29761.904 Hz
FIDRES 0.908261 Hz
AQ 1.1010048 sec
RG 198.17
DW 16.800 usec
DE 6.50 usec
TE 298.0 K
CNST2 145.0000000
CNST11 1.0000000
D1 2.00000000 sec
D20 0.00689655 sec
TD0 1
SFO1 125.7703643 MHz
NUC1 13C
P1 8.00 usec
P2 16.00 usec
PLW1 53.00000000 W
SFO2 500.1320005 MHz
NUC2 1H
CPDPRG[2 waltz16
PCPD2 80.00 usec
PLW2 28.00000000 W
PLW12 0.42017001 W

F2 - Processing parameters
SI 32768
SF 125.7577972 MHz
WDW EM
SSB 0
LB 1.00 Hz
GB 0
PC 1.40

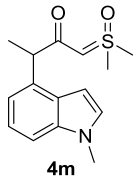

4m

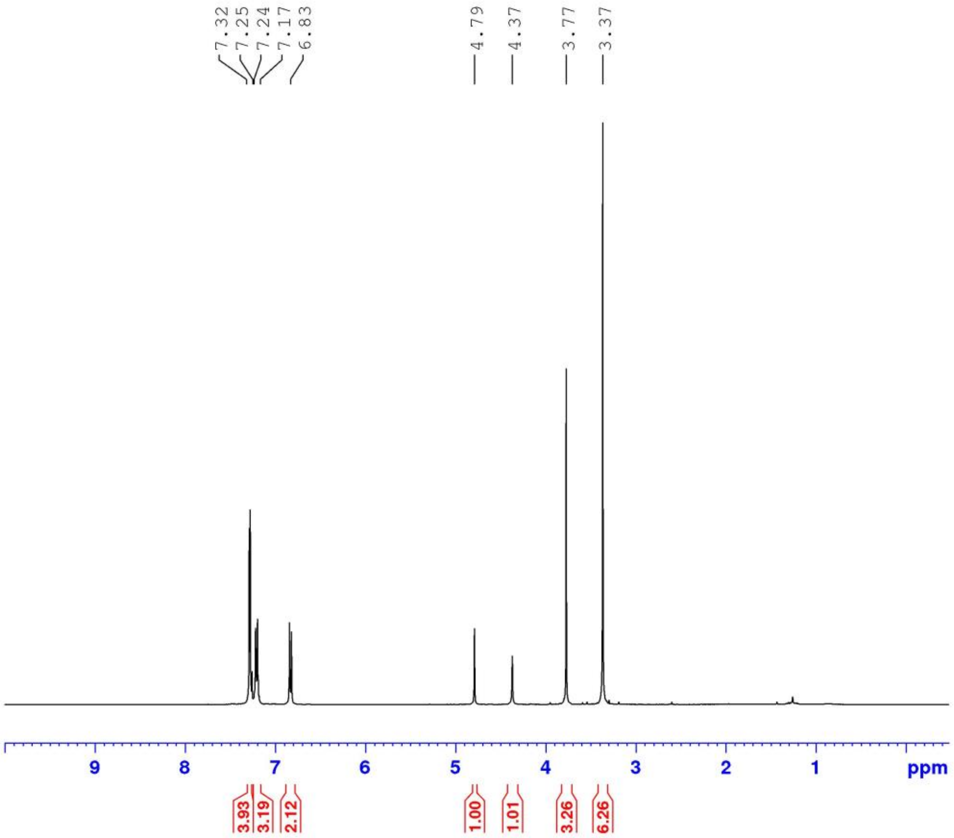


```
Current Data Parameters
NAME          compound 4n
EXPNO                   1
PROCNO                  1

F2 - Acquisition Parameters
Date_            20180815
Time                17.22
INSTRUM             spect
PROBHD   5 mm QNP 1H/13
PULPROG              zg30
TD                  65536
SOLVENT             CDCl3
NS                     16
DS                      2
SWH              8278.146 Hz
FIDRES           0.126314 Hz
AQ              3.9583745 sec
RG                    114
DW                 60.400 usec
DE                   6.50 usec
TE                  298.0 K
D1             1.00000000 sec
TD0                     1

======== CHANNEL f1 ========
NUC1                   1H
P1                  10.00 usec
PL1                  1.00 dB
SFO1          400.1324710 MHz

F2 - Processing parameters
SI                  32768
SF            400.1300100 MHz
WDW                    EM
SSB      0
LB                   0.30 Hz
GB       0
PC                   1.00
```

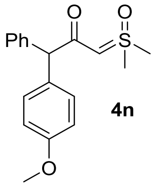

4n

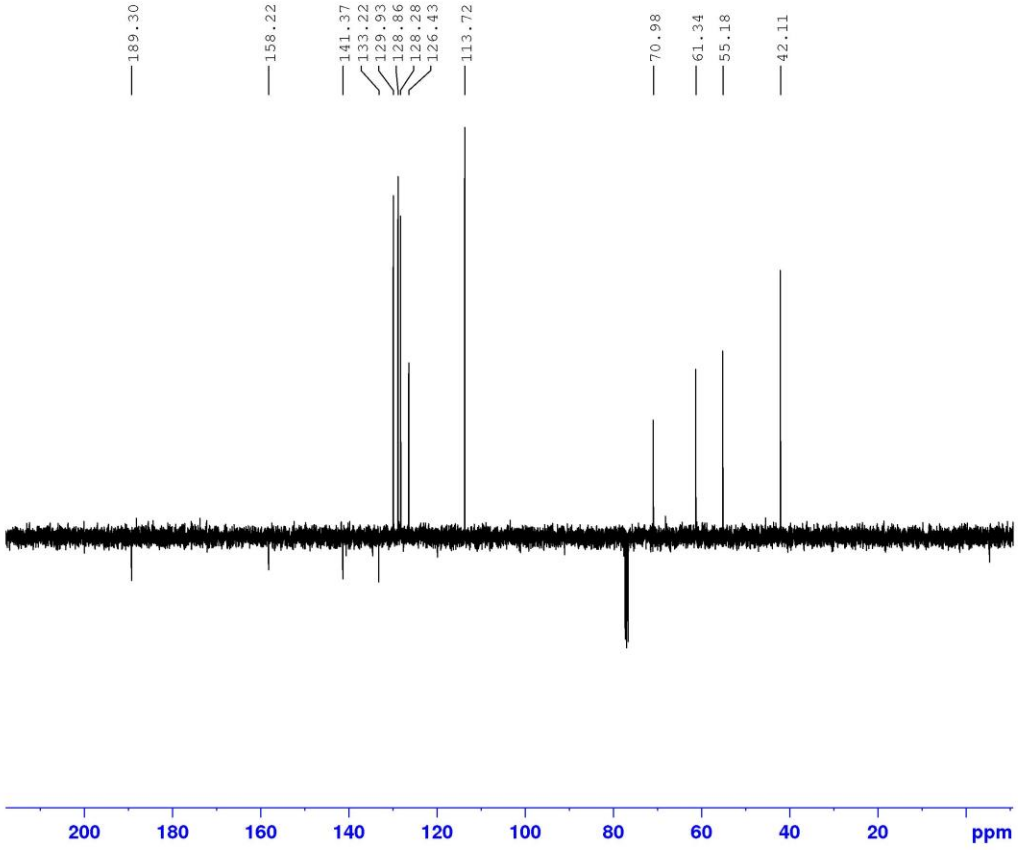


```
Current Data Parameters
NAME          compound 4n
EXPNO                   2
PROCNO                  1

F2 - Acquisition Parameters
Date_            20180815
Time                17.27
INSTRUM             spect
PROBHD   5 mm QNP 1H/13
PULPROG              jmod
TD                  65536
SOLVENT             CDCl3
NS                     68
DS                      4
SWH             23980.814 Hz
FIDRES           0.365918 Hz
AQ              1.3664256 sec
RG                  16384
DW                 20.850 usec
DE                   6.50 usec
TE                  297.9 K
CNST2         145.0000000
CNST11          1.0000000
D1             2.00000000 sec
D20            0.00689655 sec
TD0                     1

======== CHANNEL f1 ========
NUC1                  13C
P1                   8.00 usec
P2                  16.00 usec
PL1      0 dB
SFO1          100.6228298 MHz

======== CHANNEL f2 ========
CPDPRG[2          waltz16
NUC2                   1H
PCPD2               80.00 usec
PL2                  1.00 dB
PL12                19.06 dB
SFO2          400.1316005 MHz

F2 - Processing parameters
SI                  32768
SF            100.6127751 MHz
WDW                    EM
SSB      0
LB                   1.00 Hz
GB       0
PC                   1.40
```

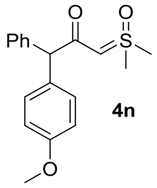

4n

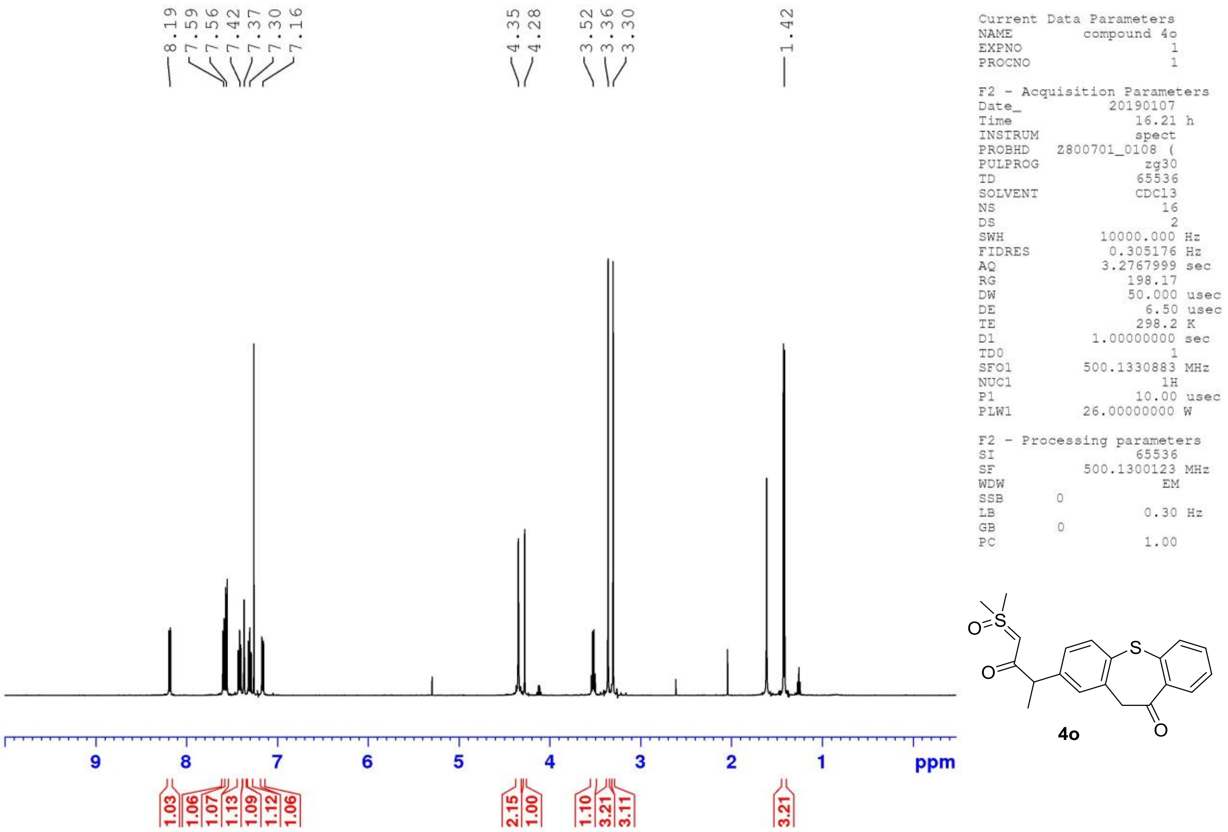
8.19
7.59
7.56
7.42
7.37
7.30
7.16
4.35
4.28
3.52
3.36
3.30
1.42
Current Data Parameters
NAME compound 4o
EXPNO 1
PROCNO 1
F2 - Acquisition Parameters
Date_ 20190107
Time 16.21 h
INSTRUM spect
PROBHD Z800701_0108 (
PULPROG zg30
TD 65536
SOLVENT CDCl3
NS 16
DS 2
SWH 10000.000 Hz
FIDRES 0.305176 Hz
AQ 3.2767999 sec
RG 198.17
DW 50.000 usec
DE 6.50 usec
TE 298.2 K
D1 1.00000000 sec
TD0 1
SFO1 500.1330883 MHz
NUC1 1H
P1 10.00 usec
PLW1 26.00000000 W
F2 - Processing parameters
SI 65536
SF 500.1300123 MHz
WDW EM
SSB 0
LB 0.30 Hz
GB 0
PC 1.00
4o
9
8
7
6
5
4
3
2
1
ppm
1.03
1.06
1.07
1.13
1.09
1.12
1.06
2.15
1.00
1.10
3.21
3.11
3.21

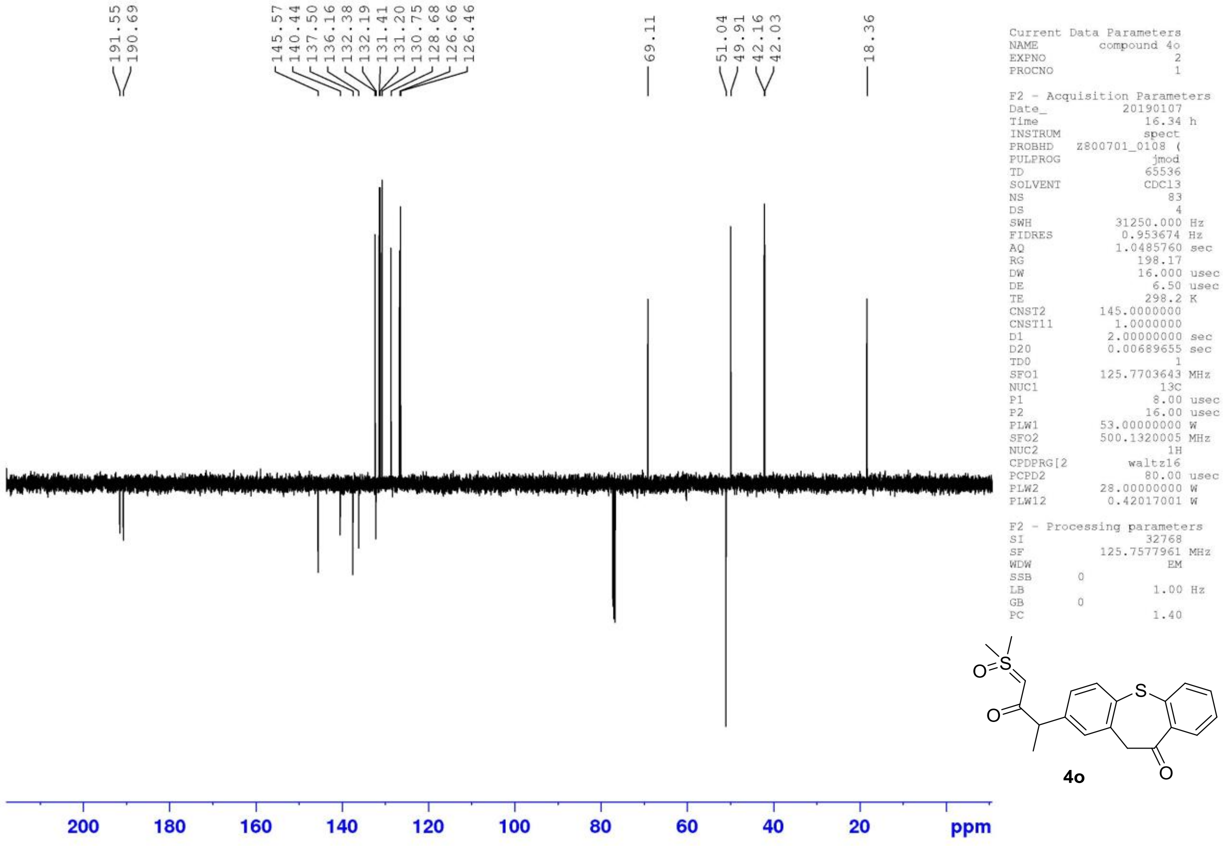
191.55
190.69
145.57
140.44
137.50
136.16
132.38
132.19
131.41
131.20
130.75
128.68
126.66
126.46
69.11
51.04
49.91
42.16
42.03
18.36
Current Data Parameters
NAME compound 4o
EXPNO 2
PROCNO 1
F2 - Acquisition Parameters
Date_ 20190107
Time 16.34 h
INSTRUM spect
PROBHD Z800701_0108 (
PULPROG jmod
TD 65536
SOLVENT CDCl3
NS 83
DS 4
SWH 31250.000 Hz
FIDRES 0.953674 Hz
AQ 1.0485760 sec
RG 198.17
DW 16.000 usec
DE 6.50 usec
TE 298.2 K
CNST2 145.0000000
CNST11 1.0000000
D1 2.00000000 sec
D20 0.00689655 sec
TD0 1
SFO1 125.7703643 MHz
NUC1 13C
P1 8.00 usec
P2 16.00 usec
PLW1 53.00000000 W
SFO2 500.1320005 MHz
NUC2 1H
CPDPRG[2 waltz16
PCPD2 80.00 usec
PLW2 28.00000000 W
PLW12 0.42017001 W
F2 - Processing parameters
SI 32768
SF 125.7577961 MHz
WDW EM
SSB 0
LB 1.00 Hz
GB 0
PC 1.40
4o
200
180
160
140
120
100
80
60
40
20
ppm

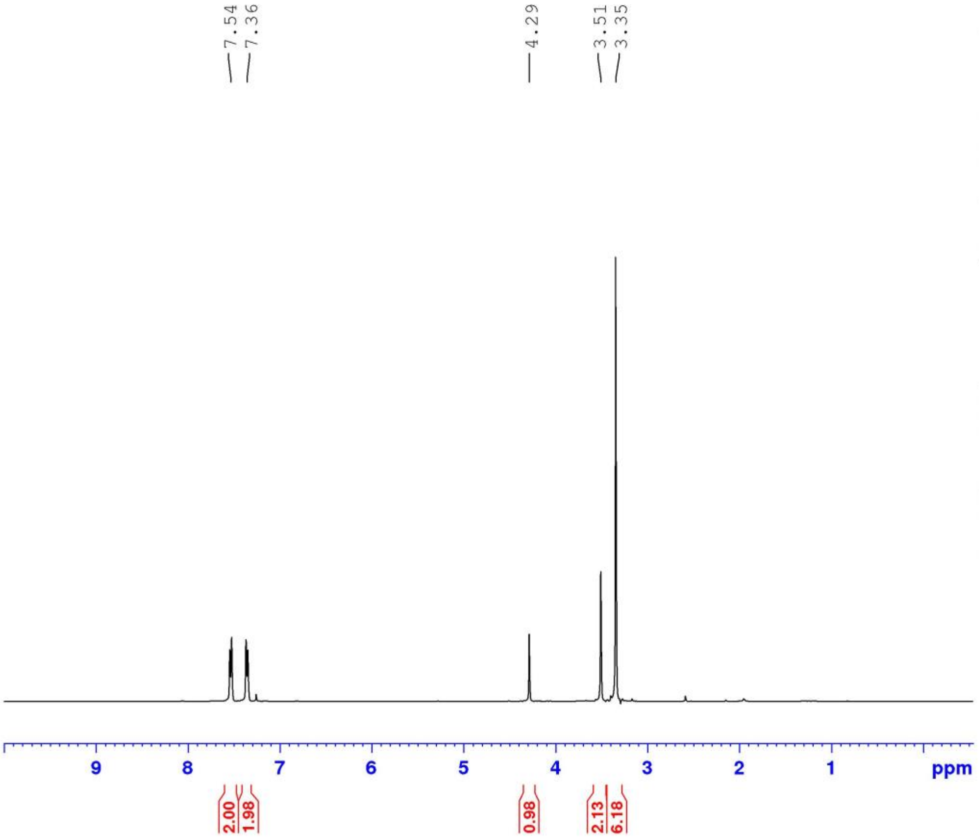

Current Data Parameters
NAME compound S-11
EXPNO 1
PROCNO 1

F2 - Acquisition Parameters
Date_ 20190806
Time 16.24
INSTRUM spect
PROBHD 5 mm QNP 1H/1
PULPROG zg30
TD 65536
SOLVENT CDCl3
NS 16
DS 2
SWH 8278.146 Hz
FIDRES 0.126314 Hz
AQ 3.9583745 sec
RG 90.5
DW 60.400 usec
DE 6.50 usec
TE 298.0 K
D1 1.00000000 sec
TD0 1

======== CHANNEL f1 ========
NUC1 1H
P1 10.00 usec
PL1 0.50 dB
SFO1 400.1324710 MHz

F2 - Processing parameters
SI 32768
SF 400.1300113 MHz
WDW EM
SSB 0
LB 0.30 Hz
GB 0
PC 1.00

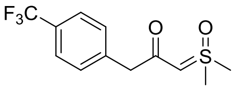

S-11

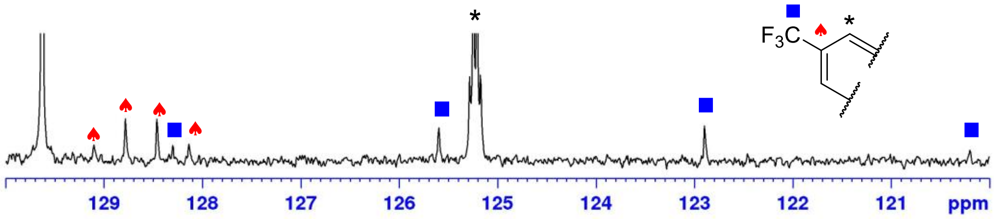

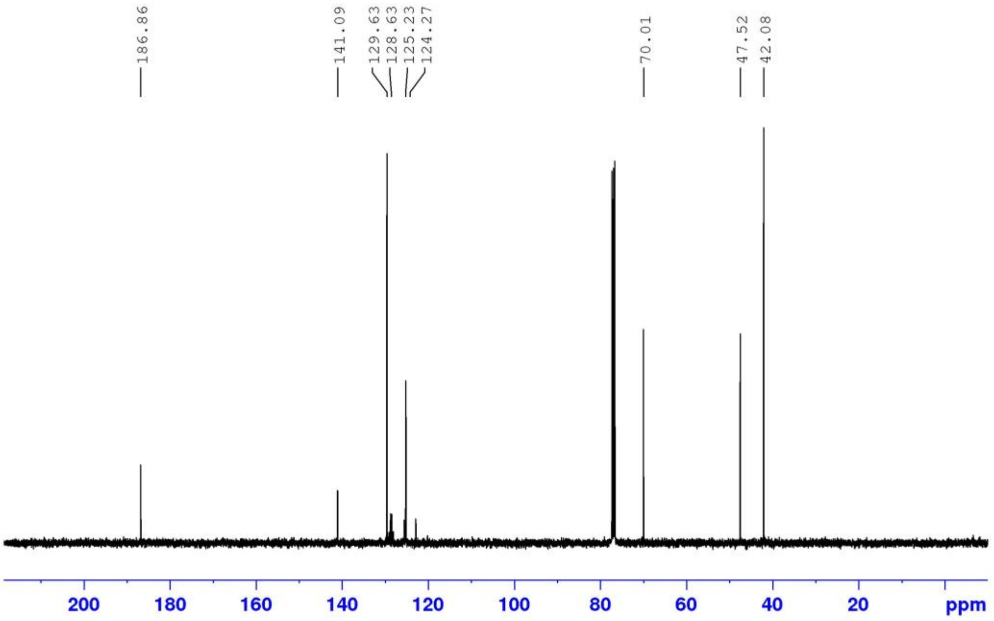

Current Data Parameters
NAME compound S-11
EXPNO 2
PROCNO 1

F2 - Acquisition Parameters
Date_ 20190806
Time 16.30
INSTRUM spect
PROBHD 5 mm QNP 1H/1
PULPROG zgpg30
TD 65536
SOLVENT CDCl3
NS 356
DS 4
SWH 23980.814 Hz
FIDRES 0.365918 Hz
AQ 1.3664256 sec
RG 161.3
DW 20.850 usec
DE 6.50 usec
TE 298.0 K
D1 2.00000000 sec
D11 0.03000000 sec
TD0 1

======== CHANNEL f1 ========
NUC1 13C
P1 6.50 usec
PL1 0 dB
SFO1 100.6228298 MHz

======== CHANNEL f2 ========
CPDPRG[2 waltz16
NUC2 1H
PCPD2 80.00 usec
PL2 1.70 dB
PL12 19.76 dB
PL13 21.00 dB
SFO2 400.1316005 MHz

F2 - Processing parameters
SI 32768
SF 100.6127761 MHz
WDW EM
SSB 0
LB 1.00 Hz
GB 0
PC 1.40

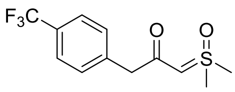

S-11

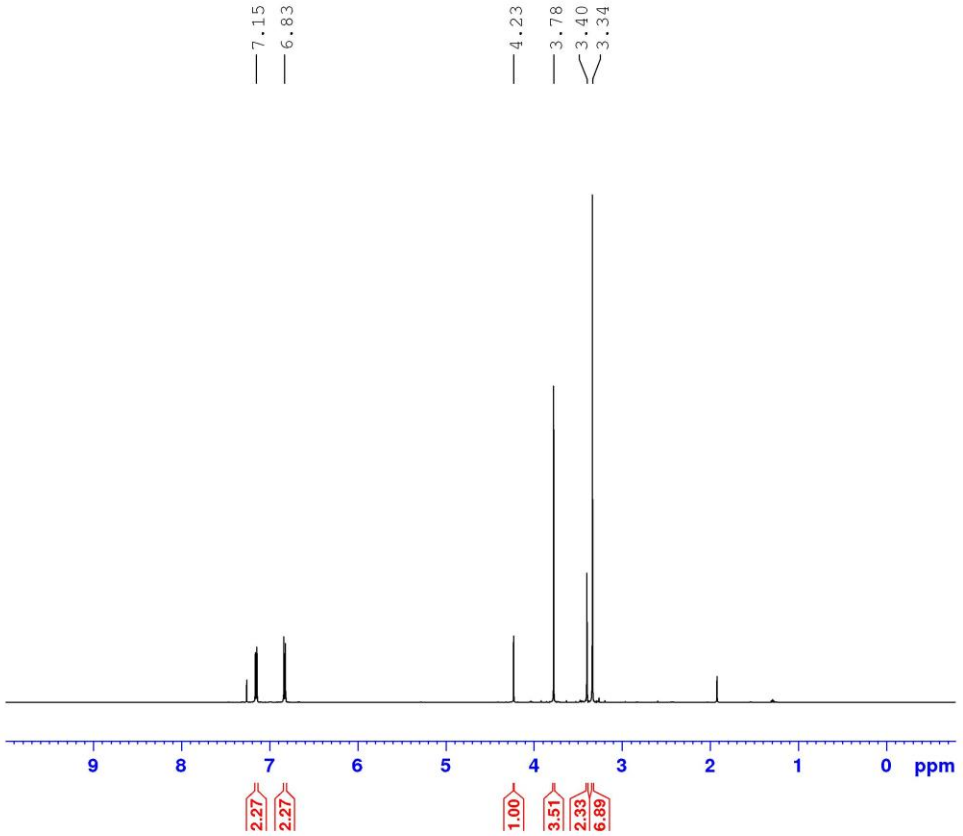

Current Data Parameters
NAME Compound S-13
EXPNO 1
PROCNO 1

F2 - Acquisition Parameters
Date_ 20161020
Time 15.05
INSTRUM spect
PROBHD 5 mm PABBO BB-
PULPROG zg30
TD 65536
SOLVENT CDCl3
NS 16
DS 2
SWH 10000.000 Hz
FIDRES 0.152588 Hz
AQ 3.2767999 sec
RG 134.89
DW 50.000 usec
DE 6.50 usec
TE 298.0 K
D1 1.00000000 sec
TD0 1

======== CHANNEL f1 ========
SFO1 500.1330885 MHz
NUC1 1H
P1 10.00 usec
PLW1 26.00000000 W

F2 - Processing parameters
SI 65536
SF 500.1300121 MHz
WDW EM
SSB 0
LB 0.30 Hz
GB 0
PC 1.00

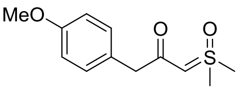

S-13

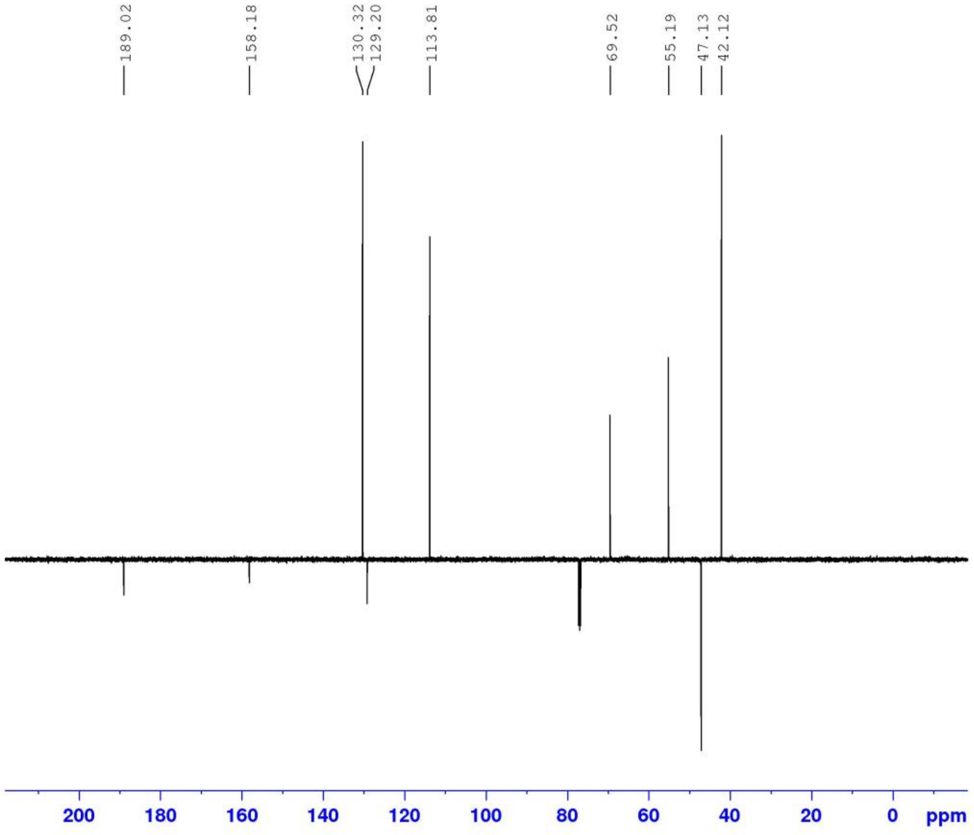

Current Data Parameters
NAME Compound S-13
EXPNO 2
PROCNO 1

F2 - Acquisition Parameters
Date_ 20161020
Time 15.21
INSTRUM spect
PROBHD 5 mm PABBO BB-
PULPROG jmod
TD 65536
SOLVENT CDCl3
NS 256
DS 4
SWH 29761.904 Hz
FIDRES 0.454131 Hz
AQ 1.1010048 sec
RG 198.17
DW 16.800 usec
DE 6.50 usec
TE 298.0 K
CNST2 145.0000000
CNST11 1.0000000
D1 2.00000000 sec
D20 0.00689655 sec
TD0 1

======== CHANNEL f1 ========
SFO1 125.7703643 MHz
NUC1 13C
P1 8.00 usec
P2 16.00 usec
PLW1 53.00000000 W

======== CHANNEL f2 ========
SFO2 500.1320005 MHz
NUC2 1H
CPDPRG[2 waltz16
PCPD2 80.00 usec
PLW2 28.00000000 W
PLW12 0.42017001 W

F2 - Processing parameters
SI 32768
SF 125.7577974 MHz
WDW EM
SSB 0
LB 1.00 Hz
GB 0
PC 1.40

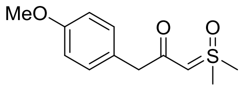

S-13

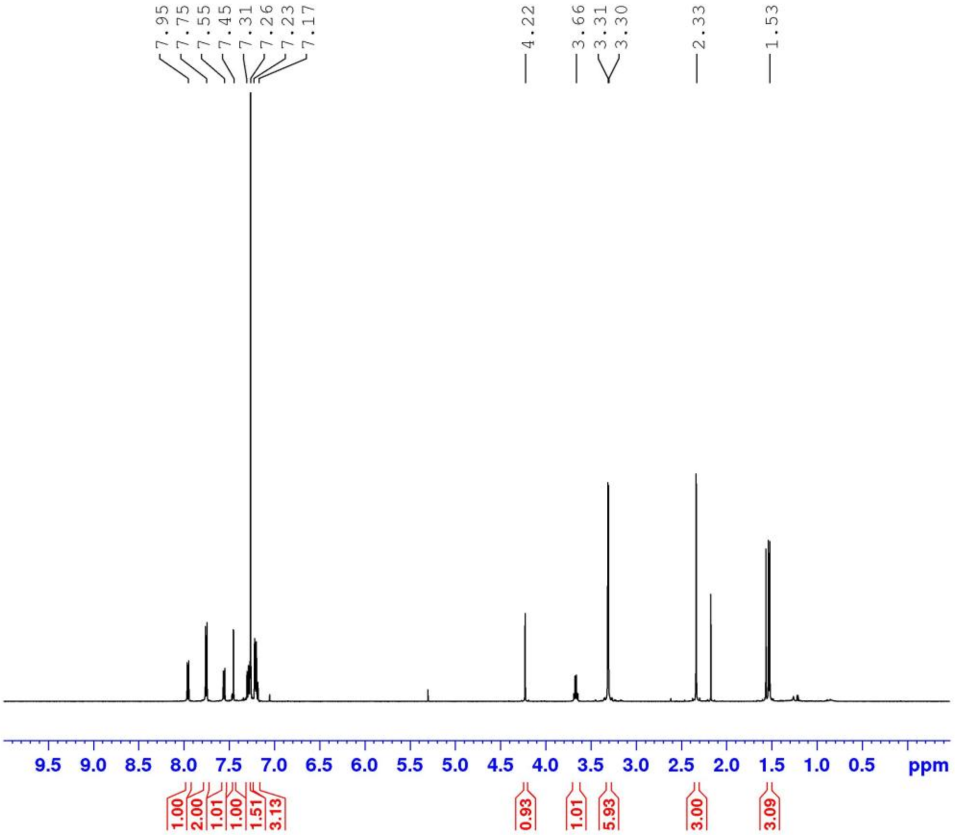

Current Data Parameters
NAME compound 9
EXPNO 1
PROCNO 1

F2 - Acquisition Parameters
Date_ 20190425
Time 9.09 h
INSTRUM spect
PROBHD Z800701_0108 (
PULPROG zg30
TD 65536
SOLVENT CDCl3
NS 16
DS 2
SWH 10000.000 Hz
FIDRES 0.305176 Hz
AQ 3.2767999 sec
RG 198.17
DW 50.000 usec
DE 6.50 usec
TE 298.0 K
D1 1.00000000 sec
TD0 1
SFO1 500.1330883 MHz
NUC1 1H
P1 10.00 usec
PLW1 26.00000000 W

F2 - Processing parameters
SI 65536
SF 500.1300122 MHz
WDW EM
SSB 0
LB 0.30 Hz
GB 0
PC 1.00

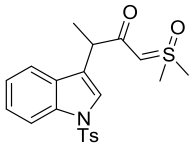

9

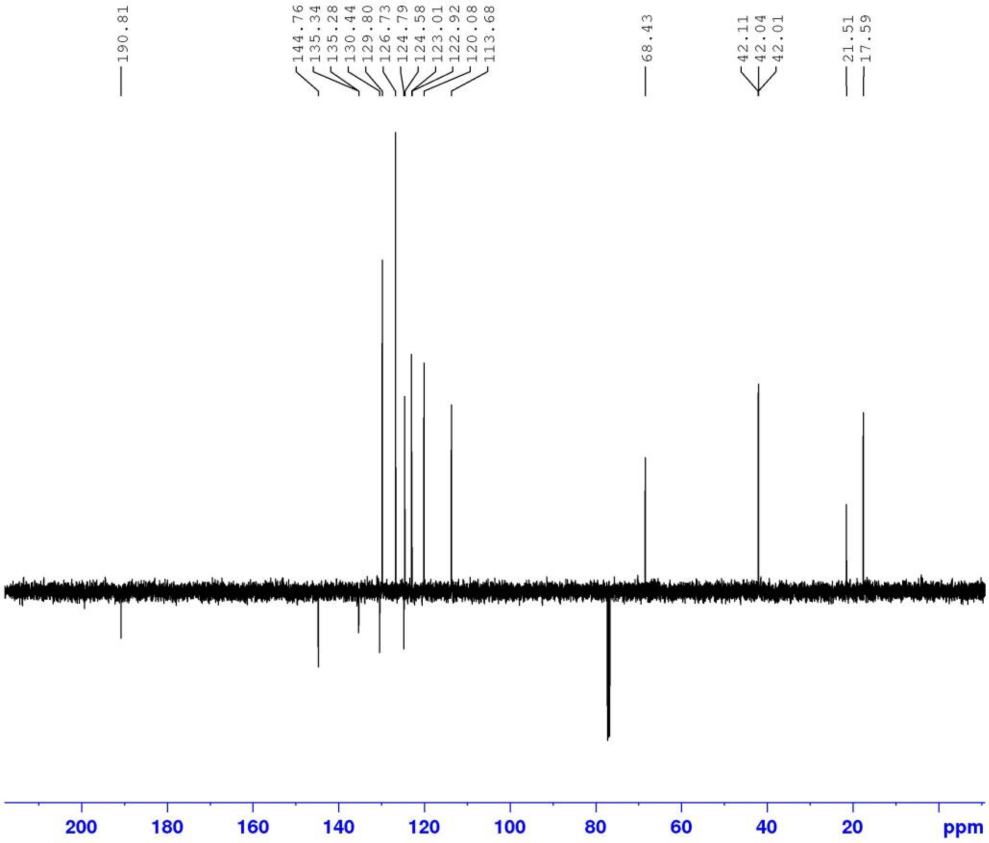

Current Data Parameters
NAME compound 9
EXPNO 3
PROCNO 1

F2 - Acquisition Parameters
Date_ 20190425
Time 9.24 h
INSTRUM spect
PROBHD Z800701_0108 (
PULPROG jmod
TD 65536
SOLVENT CDCl3
NS 81
DS 4
SWH 29761.904 Hz
FIDRES 0.908261 Hz
AQ 1.1010048 sec
RG 198.17
DW 16.800 usec
DE 6.50 usec
TE 298.0 K
CNST2 145.0000000
CNST11 1.0000000
D1 2.00000000 sec
D20 0.00689655 sec
TD0 1
SFO1 125.7703643 MHz
NUC1 13C
P1 8.00 usec
P2 16.00 usec
PLW1 53.00000000 W
SFO2 500.1320005 MHz
NUC2 1H
CPDPRG[2 waltz16
PCPD2 80.00 usec
PLW2 28.00000000 W
PLW12 0.42017001 W

F2 - Processing parameters
SI 32768
SF 125.7577944 MHz
WDW EM
SSB 0
LB 1.00 Hz
GB 0
PC 1.40

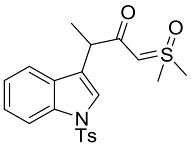

9

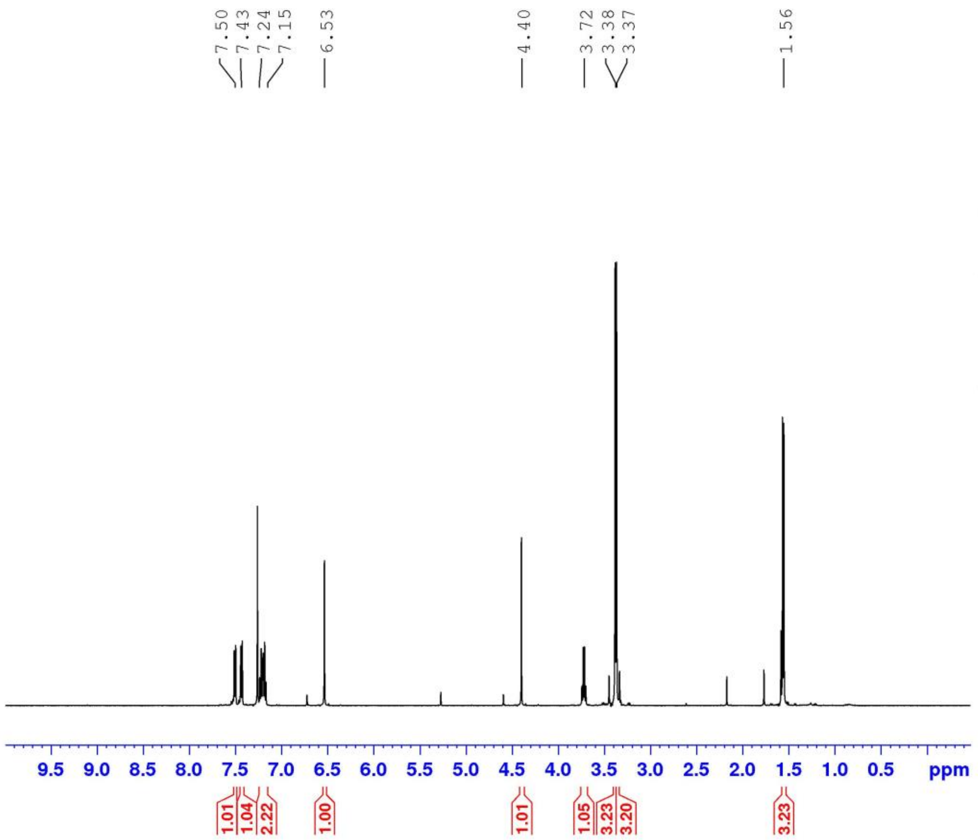


```
Current Data Parameters
NAME          compound 12
EXPNO                   1
PROCNO                  1

F2 - Acquisition Parameters
Date_            20190622
Time                11.44 h
INSTRUM             spect
PROBHD   Z800701_0108 (
PULPROG              zg30
TD                  65536
SOLVENT             CDCl3
NS                     16
DS                      2
SWH             10000.000 Hz
FIDRES           0.305176 Hz
AQ              3.2767999 sec
RG                 198.17
DW                 50.000 usec
DE                  13.89 usec
TE                  298.0 K
D1             1.00000000 sec
TD0                     1
SFO1          500.1330883 MHz
NUC1                   1H
P0                   3.33 usec
P1                  10.00 usec
PLW1          26.00000000 W

F2 - Processing parameters
SI                  65536
SF            500.1300121 MHz
WDW                    EM
SSB       0
LB                   0.30 Hz
GB        0
PC                   1.00
```

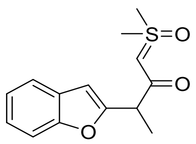

12

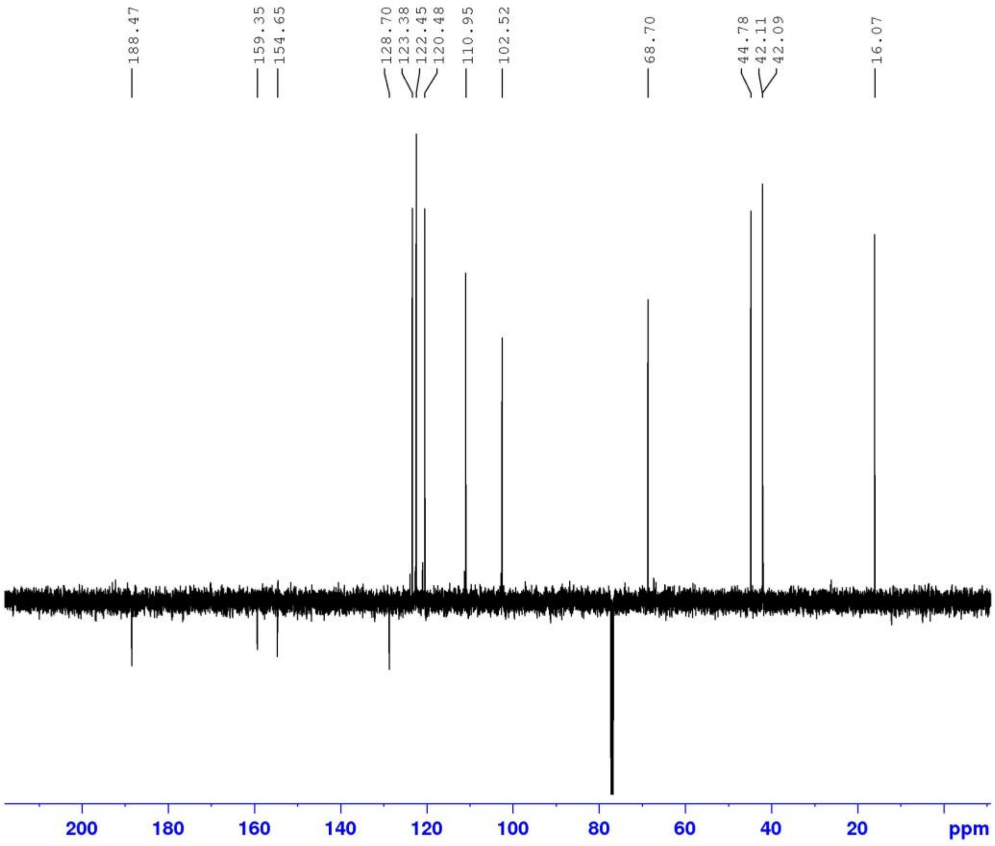


```
Current Data Parameters
NAME          compound 12
EXPNO                   2
PROCNO                  1

F2 - Acquisition Parameters
Date_            20190622
Time                12.00 h
INSTRUM             spect
PROBHD   Z800701_0108 (
PULPROG              jmod
TD                  65536
SOLVENT             CDCl3
NS                     59
DS                      4
SWH             29761.904 Hz
FIDRES           0.908261 Hz
AQ              1.1010048 sec
RG                 198.17
DW                 16.800 usec
DE                   6.50 usec
TE                  298.5 K
CNST2         145.0000000
CNST11          1.0000000
D1             2.00000000 sec
D20            0.00689655 sec
TD0                     1
SFO1          125.7703643 MHz
NUC1                  13C
P1                   8.00 usec
P2                  16.00 usec
PLW1          53.00000000 W
SFO2          500.1320005 MHz
NUC2                   1H
CPDPRG[2          waltz65
PCPD2               80.00 usec
PLW2          28.00000000 W
PLW12          0.42017001 W

F2 - Processing parameters
SI                  32768
SF            125.7577962 MHz
WDW                    EM
SSB       0
LB                   1.00 Hz
GB        0
PC                   1.40
```

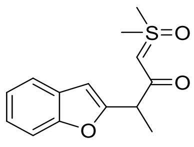

12

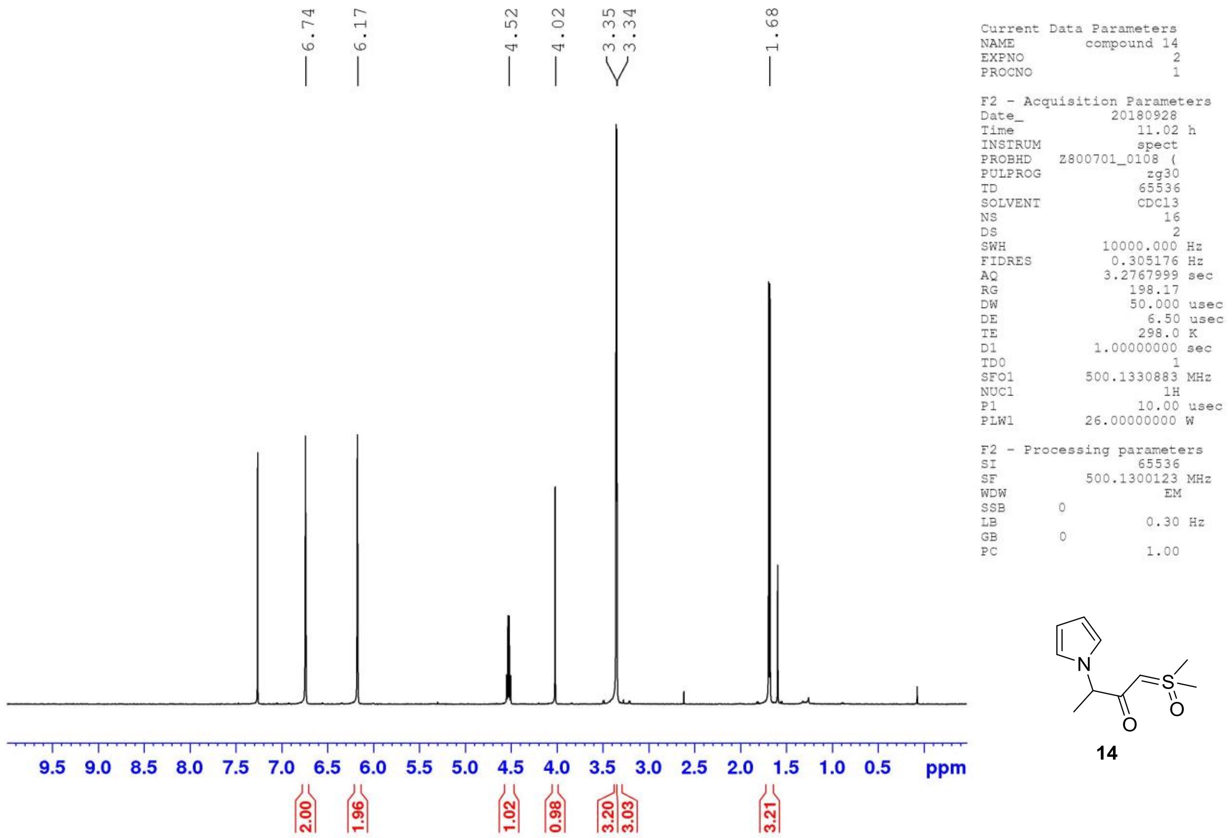

6.74
6.17
4.52
4.02
3.35
3.34
1.68
Current Data Parameters
NAME compound 14
EXPNO 2
PROCNO 1
F2 - Acquisition Parameters
Date_ 20180928
Time 11.02 h
INSTRUM spect
PROBHD Z800701_0108 (
PULPROG zg30
TD 65536
SOLVENT CDCl3
NS 16
DS 2
SWH 10000.000 Hz
FIDRES 0.305176 Hz
AQ 3.2767999 sec
RG 198.17
DW 50.000 usec
DE 6.50 usec
TE 298.0 K
D1 1.00000000 sec
TD0 1
SFO1 500.1330883 MHz
NUC1 1H
P1 10.00 usec
PLW1 26.00000000 W
F2 - Processing parameters
SI 65536
SF 500.1300123 MHz
WDW EM
SSB 0
LB 0.30 Hz
GB 0
PC 1.00
9.5 9.0 8.5 8.0 7.5 7.0 6.5 6.0 5.5 5.0 4.5 4.0 3.5 3.0 2.5 2.0 1.5 1.0 0.5 ppm
2.00
1.96
1.02
0.98
3.20
3.03
3.21
14

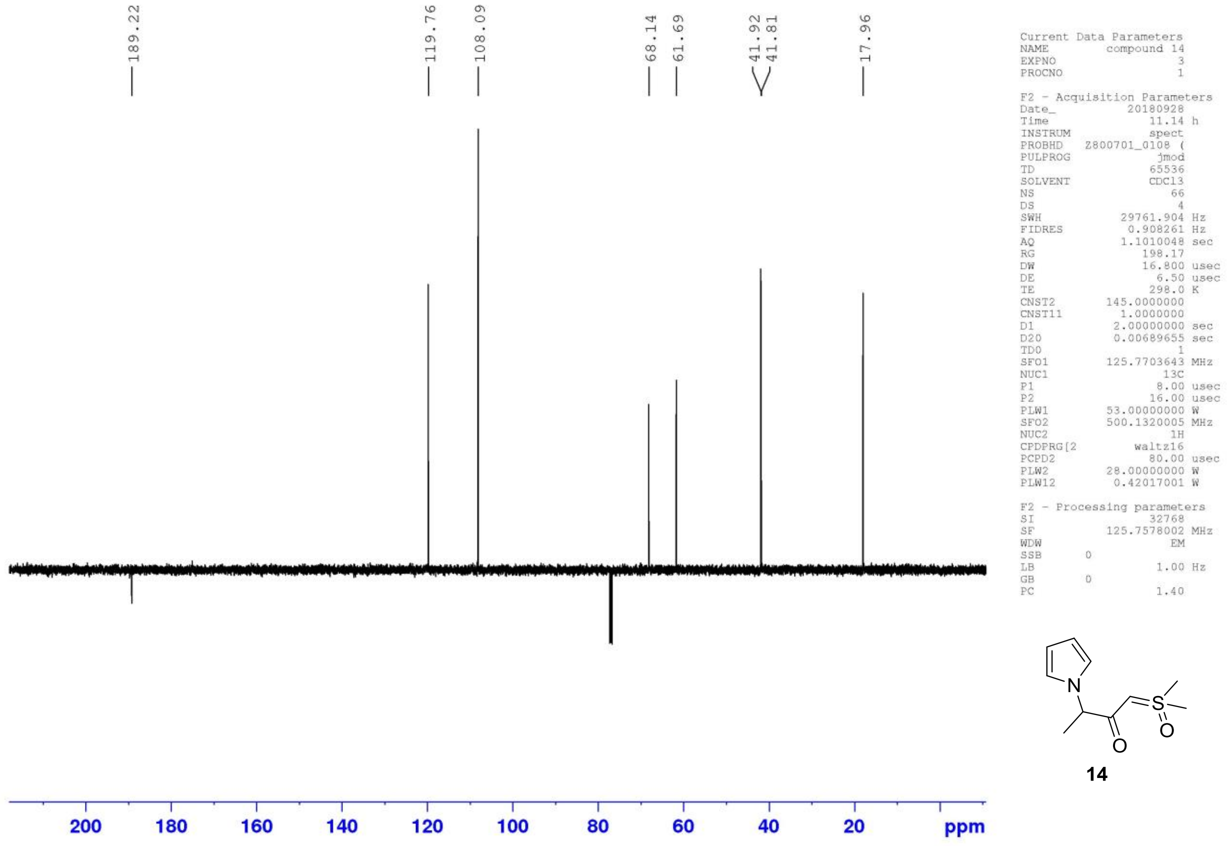

189.22
119.76
108.09
68.14
61.69
41.92
41.81
17.96
Current Data Parameters
NAME compound 14
EXPNO 3
PROCNO 1
F2 - Acquisition Parameters
Date_ 20180928
Time 11.14 h
INSTRUM spect
PROBHD Z800701_0108 (
PULPROG jmod
TD 65536
SOLVENT CDCl3
NS 66
DS 4
SWH 29761.904 Hz
FIDRES 0.908261 Hz
AQ 1.1010048 sec
RG 198.17
DW 16.800 usec
DE 6.50 usec
TE 298.0 K
CNST2 145.0000000
CNST11 1.0000000
D1 2.00000000 sec
D20 0.00689655 sec
TD0 1
SFO1 125.7703643 MHz
NUC1 13C
P1 8.00 usec
P2 16.00 usec
PLW1 53.00000000 W
SFO2 500.1320005 MHz
NUC2 1H
CPDPRG[2 waltz16
PCPD2 80.00 usec
PLW2 28.00000000 W
PLW12 0.42017001 W
F2 - Processing parameters
SI 32768
SF 125.7578002 MHz
WDW EM
SSB 0
LB 1.00 Hz
GB 0
PC 1.40
200 180 160 140 120 100 80 60 40 20 ppm
14

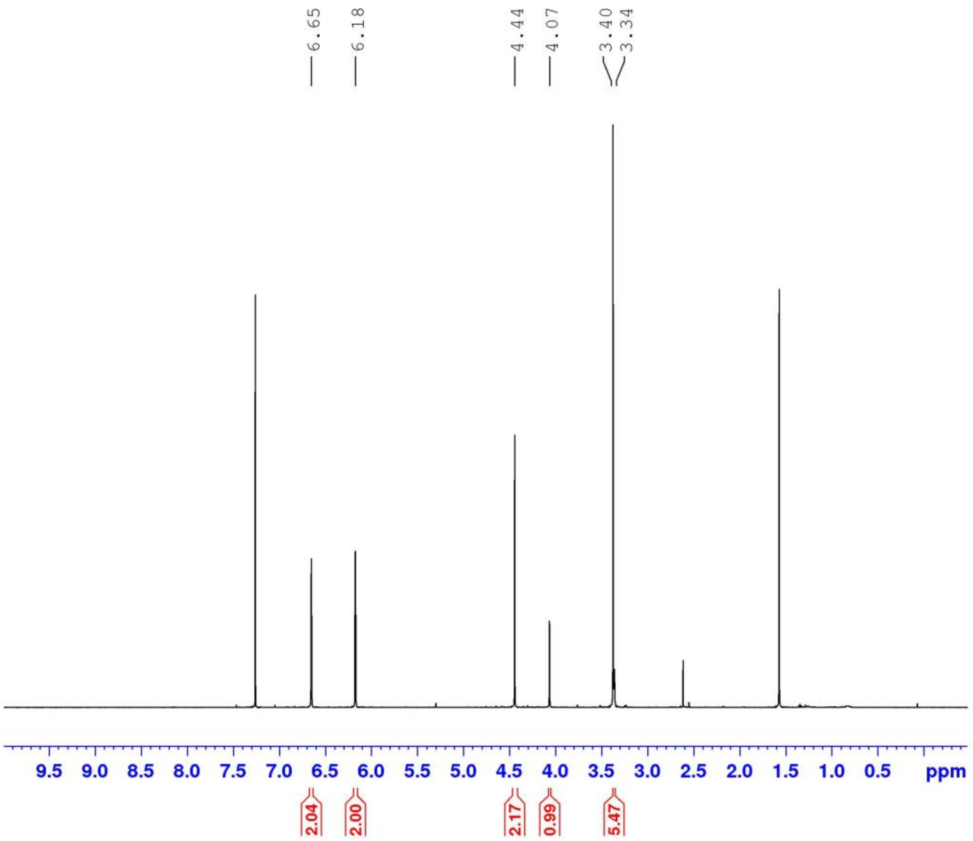
6.65
6.18
4.44
4.07
3.40
3.34
9.5 9.0 8.5 8.0 7.5 7.0 6.5 6.0 5.5 5.0 4.5 4.0 3.5 3.0 2.5 2.0 1.5 1.0 0.5 ppm
2.04
2.00
2.17
0.99
5.47
Current Data Parameters
NAME compound 17a
EXPNO 1
PROCNO 1
F2 - Acquisition Parameters
Date_ 20161129
Time 8.42
INSTRUM spect
PROBHD 5 mm PABBO BB-
PULPROG zg30
TD 65536
SOLVENT CDCl3
NS 16
DS 2
SWH 10000.000 Hz
FIDRES 0.152588 Hz
AQ 3.2767999 sec
RG 198.17
DW 50.000 usec
DE 6.50 usec
TE 298.0 K
D1 1.00000000 sec
TD0 1
======== CHANNEL f1 ========
SFO1 500.1330885 MHz
NUC1 1H
P1 10.00 usec
PLW1 26.00000000 W
F2 - Processing parameters
SI 65536
SF 500.1300120 MHz
WDW EM
SSB 0
LB 0.30 Hz
GB 0
PC 1.00

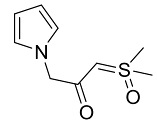

17a

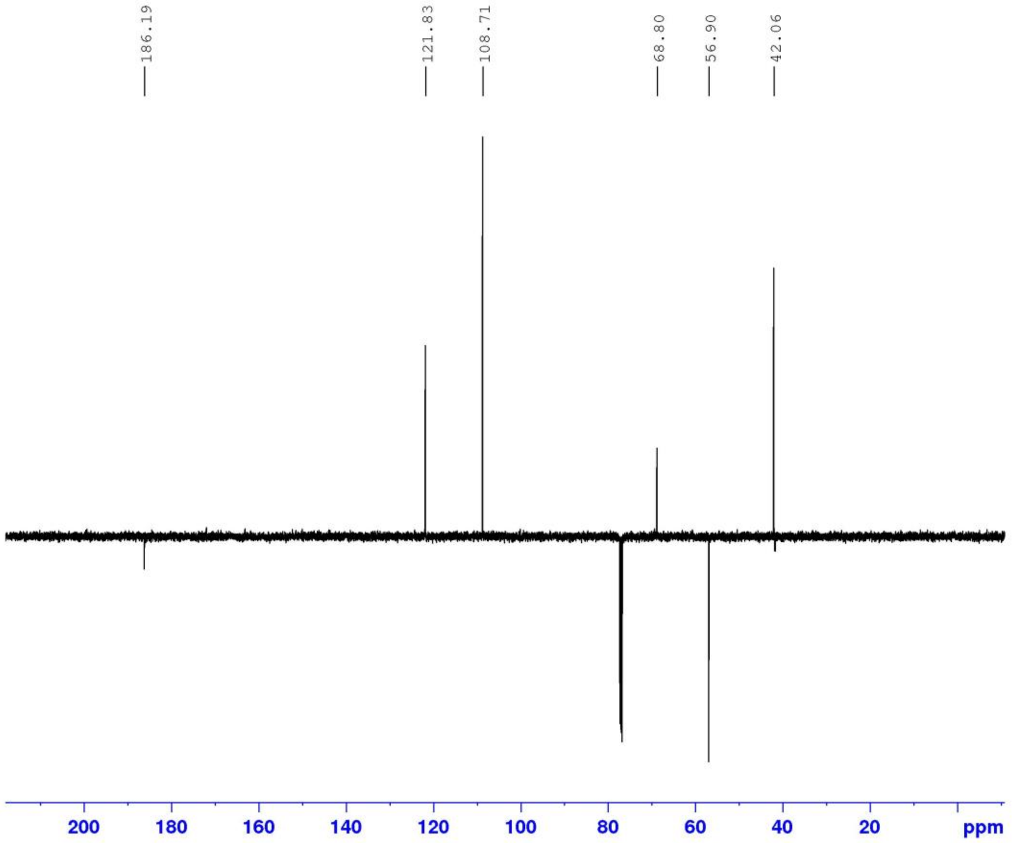
186.19
121.83
108.71
68.80
56.90
42.06
200 180 160 140 120 100 80 60 40 20 ppm
Current Data Parameters
NAME compound 17a
EXPNO 2
PROCNO 1
F2 - Acquisition Parameters
Date_ 20161220
Time 17.30
INSTRUM spect
PROBHD 5 mm PABBO BB-
PULPROG jmod
TD 65536
SOLVENT CDCl3
NS 512
DS 4
SWH 29761.904 Hz
FIDRES 0.454131 Hz
AQ 1.1010048 sec
RG 198.17
DW 16.800 usec
DE 6.50 usec
TE 298.0 K
CNST2 145.0000000
CNST11 1.0000000
D1 2.00000000 sec
D20 0.00689655 sec
TD0 1
======== CHANNEL f1 ========
SFO1 125.7703643 MHz
NUC1 13C
P1 8.00 usec
P2 16.00 usec
PLW1 53.00000000 W
======== CHANNEL f2 ========
SFO2 500.1320005 MHz
NUC2 1H
CPDPRG[2 waltz16
PCPD2 80.00 usec
PLW2 28.00000000 W
PLW12 0.42017001 W
F2 - Processing parameters
SI 32768
SF 125.7577930 MHz
WDW EM
SSB 0
LB 1.00 Hz
GB 0
PC 1.40

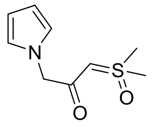

17a

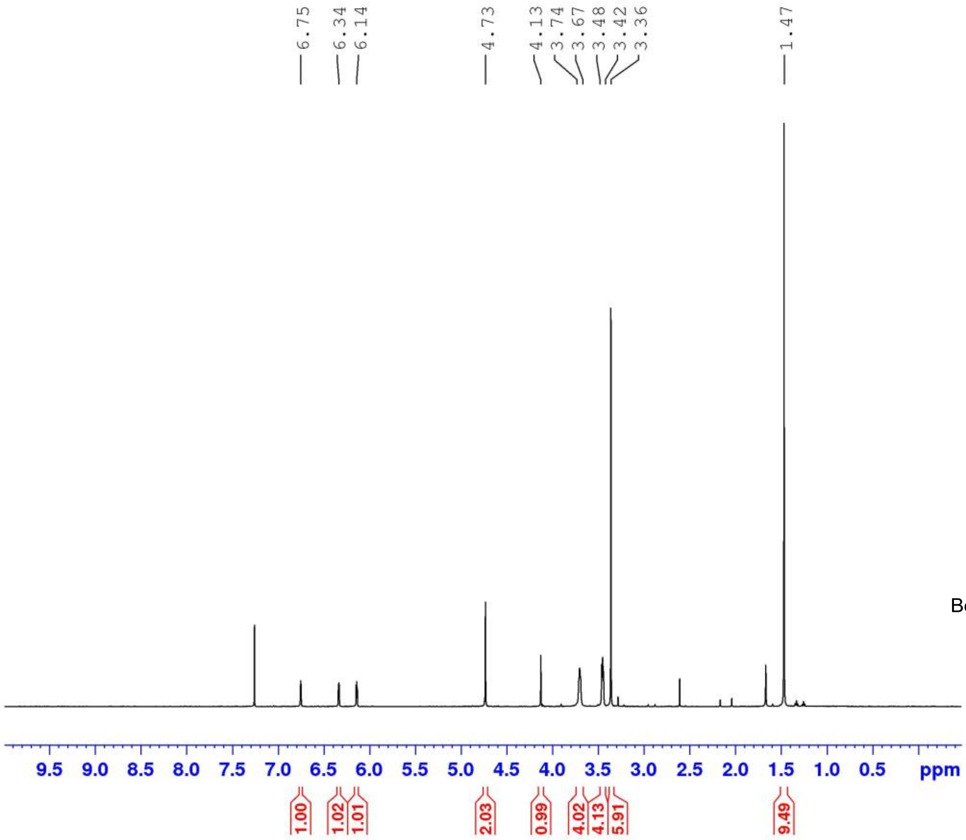

Current Data Parameters
NAME compound 17b
EXPNO 1
PROCNO 1

F2 - Acquisition Parameters
Date_ 20180727
Time 11.42 h
INSTRUM spect
PROBHD Z800701_0108 (
PULPROG zg30
TD 65536
SOLVENT CDCl3
NS 16
DS 2
SWH 10000.000 Hz
FIDRES 0.305176 Hz
AQ 3.2767999 sec
RG 198.17
DW 50.000 usec
DE 6.50 usec
TE 298.0 K
D1 1.00000000 sec
TD0 1
SFO1 500.1330883 MHz
NUC1 1H
P1 10.00 usec
PLW1 26.00000000 W

F2 - Processing parameters
SI 65536
SF 500.1300125 MHz
WDW EM
SSB 0
LB 0.30 Hz
GB 0
PC 1.00

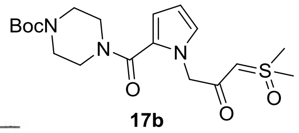

17b

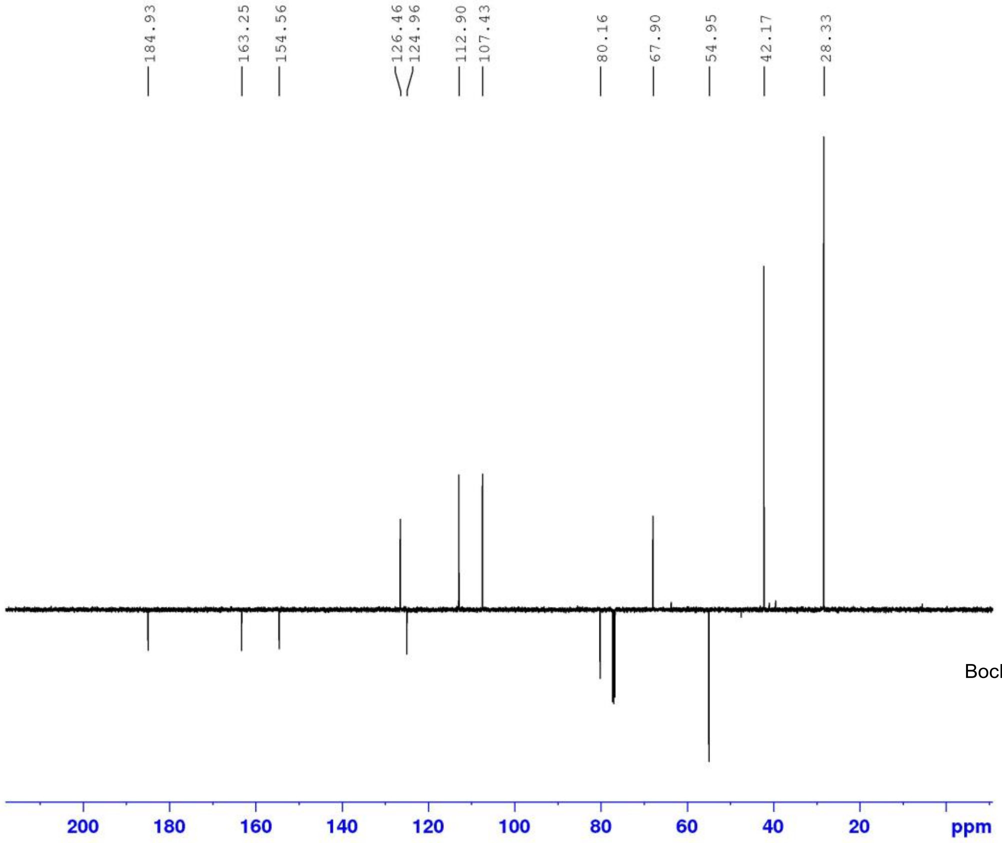

Current Data Parameters
NAME compound 17b
EXPNO 2
PROCNO 1

F2 - Acquisition Parameters
Date_ 20180727
Time 14.00 h
INSTRUM spect
PROBHD Z800701_0108 (
PULPROG jmod
TD 65536
SOLVENT CDCl3
NS 479
DS 4
SWH 29761.904 Hz
FIDRES 0.908261 Hz
AQ 1.1010048 sec
RG 198.17
DW 16.800 usec
DE 6.50 usec
TE 298.0 K
CNST2 145.0000000
CNST11 1.0000000
D1 2.00000000 sec
D20 0.00689655 sec
TD0 1
SFO1 125.7703643 MHz
NUC1 13C
P1 8.00 usec
P2 16.00 usec
PLW1 53.00000000 W
SFO2 500.1320005 MHz
NUC2 1H
CPDPRG[2 waltz16
PCPD2 80.00 usec
PLW2 28.00000000 W
PLW12 0.42017001 W

F2 - Processing parameters
SI 32768
SF 125.7577970 MHz
WDW EM
SSB 0
LB 1.00 Hz
GB 0
PC 1.40

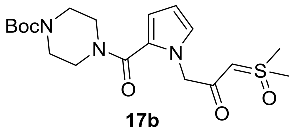

17b

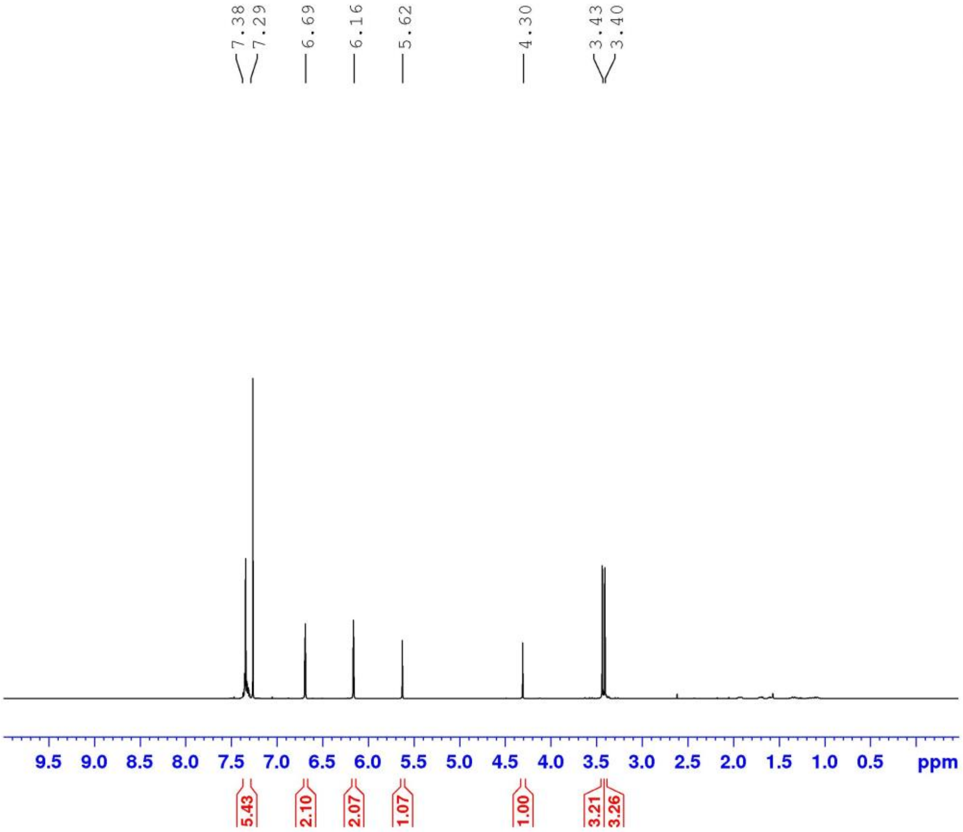


```
Current Data Parameters
NAME        compound 17c
EXPNO                  1
PROCNO                 1

F2 - Acquisition Parameters
Date_           20180628
Time                9.53 h
INSTRUM            spect
PROBHD   Z800701_0108 (
PULPROG             zg30
TD                 65536
SOLVENT            CDCl3
NS                    16
DS                     2
SWH            10000.000 Hz
FIDRES          0.305176 Hz
AQ             3.2767999 sec
RG                198.17
DW                50.000 usec
DE                  6.50 usec
TE                 298.0 K
D1            1.00000000 sec
TD0                    1
SFO1         500.1330883 MHz
NUC1                  1H
P1                 10.00 usec
PLW1         26.00000000 W

F2 - Processing parameters
SI                 65536
SF           500.1300125 MHz
WDW                   EM
SSB      0
LB                  0.30 Hz
GB       0
PC                  1.00
```

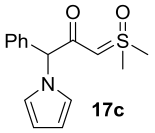

17c

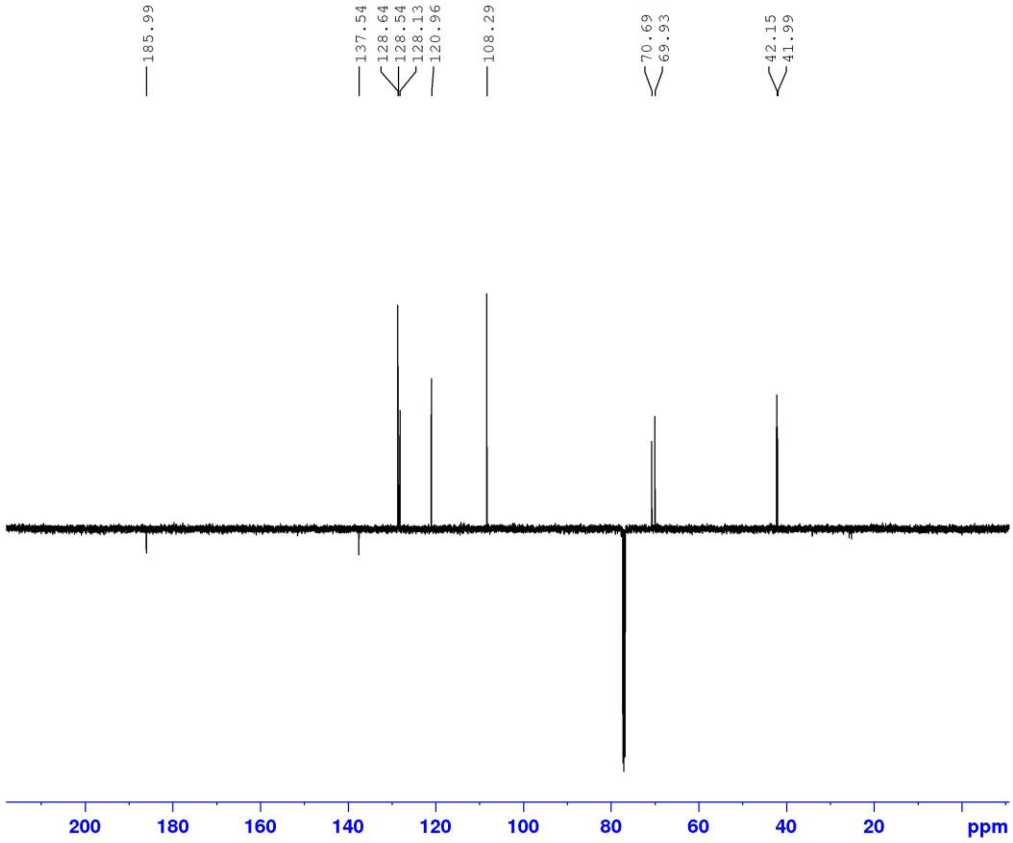


```
Current Data Parameters
NAME        compound 17c
EXPNO                  2
PROCNO                 1

F2 - Acquisition Parameters
Date_           20180627
Time               18.56 h
INSTRUM            spect
PROBHD   Z800701_0108 (
PULPROG             jmod
TD                 65536
SOLVENT            CDCl3
NS                   986
DS                     4
SWH            29761.904 Hz
FIDRES          0.908261 Hz
AQ             1.1010048 sec
RG                198.17
DW                16.800 usec
DE                  6.50 usec
TE                 298.0 K
CNST2        145.0000000
CNST11         1.0000000
D1            2.00000000 sec
D20           0.00689655 sec
TD0                    1
SFO1         125.7703643 MHz
NUC1                 13C
P1                  8.00 usec
P2                 16.00 usec
PLW1         53.00000000 W
SFO2         500.1320005 MHz
NUC2                  1H
CPDPRG[2         waltz16
PCPD2              80.00 usec
PLW2         28.00000000 W
PLW12         0.42017001 W

F2 - Processing parameters
SI                 32768
SF           125.7577927 MHz
WDW                   EM
SSB      0
LB                  1.00 Hz
GB       0
PC                  1.40
```

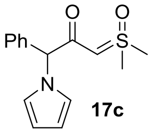

17c

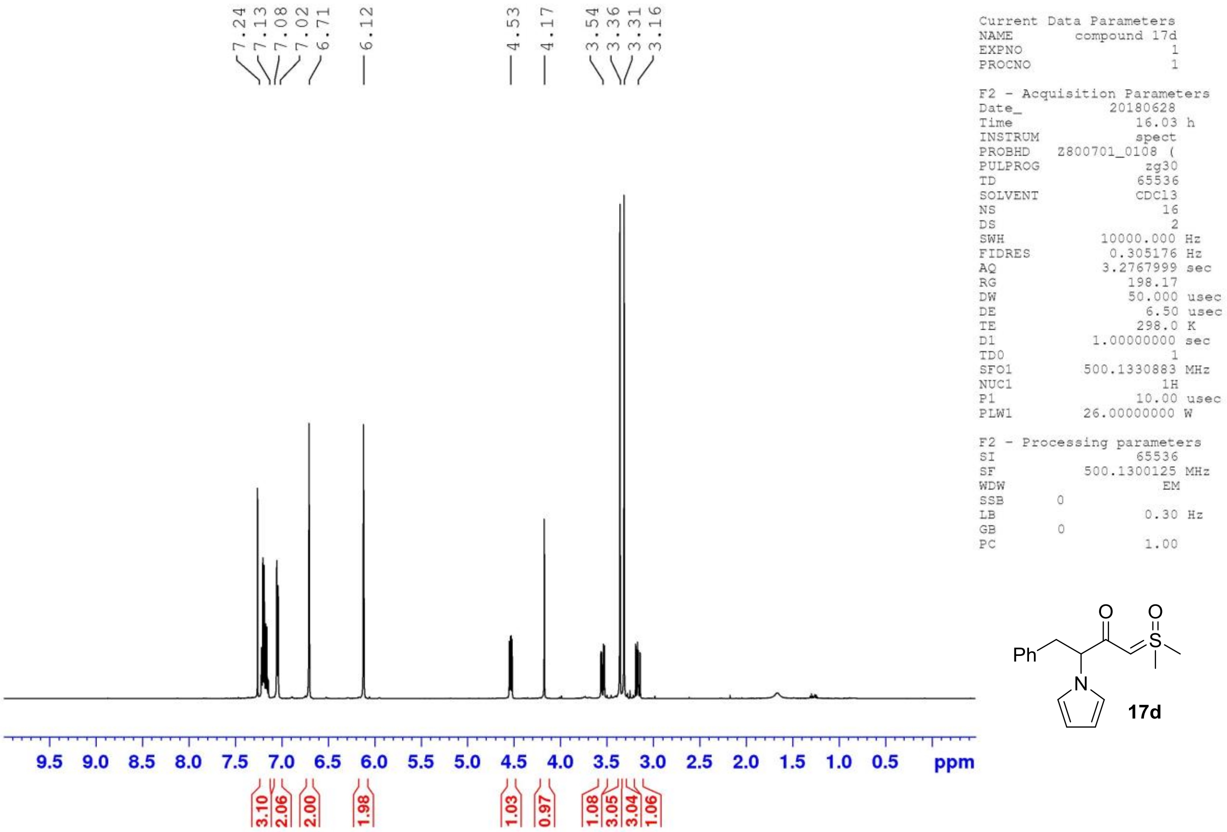
7.24
7.13
7.08
7.02
6.71
6.12
4.53
4.17
3.54
3.36
3.31
3.16
Current Data Parameters
NAME compound 17d
EXPNO 1
PROCNO 1
F2 - Acquisition Parameters
Date_ 20180628
Time 16.03 h
INSTRUM spect
PROBHD Z800701_0108 (
PULPROG zg30
TD 65536
SOLVENT CDCl3
NS 16
DS 2
SWH 10000.000 Hz
FIDRES 0.305176 Hz
AQ 3.2767999 sec
RG 198.17
DW 50.000 usec
DE 6.50 usec
TE 298.0 K
D1 1.00000000 sec
TD0 1
SFO1 500.1330883 MHz
NUC1 1H
P1 10.00 usec
PLW1 26.00000000 W
F2 - Processing parameters
SI 65536
SF 500.1300125 MHz
WDW EM
SSB 0
LB 0.30 Hz
GB 0
PC 1.00
Ph
N
17d
9.5 9.0 8.5 8.0 7.5 7.0 6.5 6.0 5.5 5.0 4.5 4.0 3.5 3.0 2.5 2.0 1.5 1.0 0.5 ppm
3.10
2.06
2.00
1.98
1.03
0.97
1.08
3.05
3.04
1.06

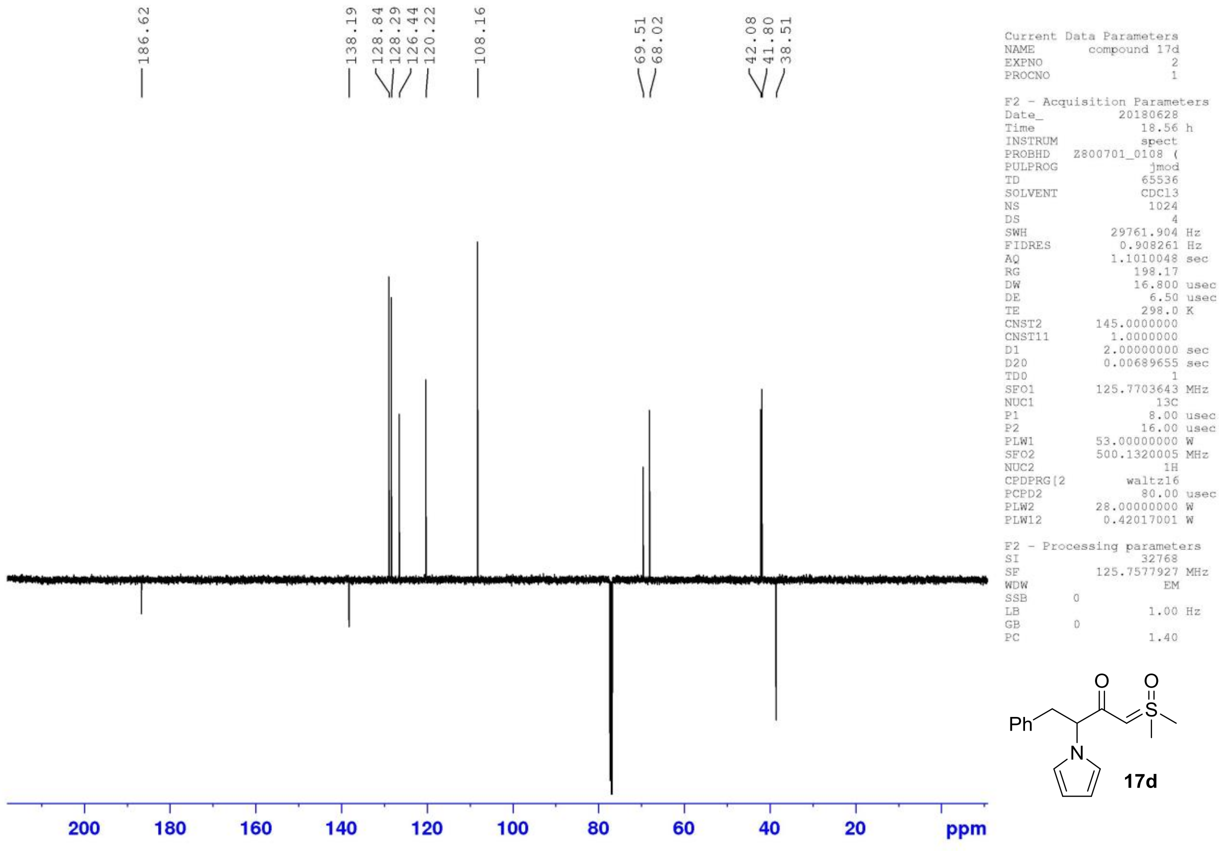
186.62
138.19
128.84
128.29
126.44
120.22
108.16
69.51
68.02
42.08
41.80
38.51
Current Data Parameters
NAME compound 17d
EXPNO 2
PROCNO 1
F2 - Acquisition Parameters
Date_ 20180628
Time 18.56 h
INSTRUM spect
PROBHD Z800701_0108 (
PULPROG jmod
TD 65536
SOLVENT CDCl3
NS 1024
DS 4
SWH 29761.904 Hz
FIDRES 0.908261 Hz
AQ 1.1010048 sec
RG 198.17
DW 16.800 usec
DE 6.50 usec
TE 298.0 K
CNST2 145.0000000
CNST11 1.0000000
D1 2.00000000 sec
D20 0.00689655 sec
TD0 1
SFO1 125.7703643 MHz
NUC1 13C
P1 8.00 usec
P2 16.00 usec
PLW1 53.00000000 W
SFO2 500.1320005 MHz
NUC2 1H
CPDPRG[2 waltz16
PCPD2 80.00 usec
PLW2 28.00000000 W
PLW12 0.42017001 W
F2 - Processing parameters
SI 32768
SF 125.7577927 MHz
WDW EM
SSB 0
LB 1.00 Hz
GB 0
PC 1.40
Ph
N
17d
200 180 160 140 120 100 80 60 40 20 ppm

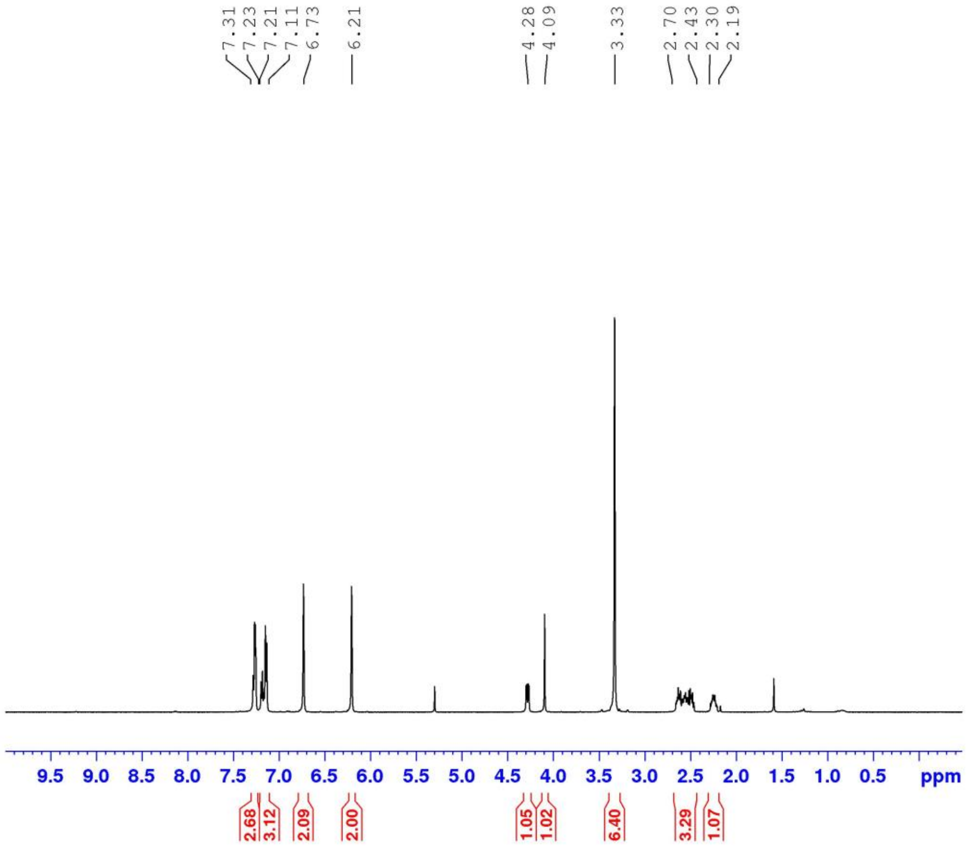


```
Current Data Parameters
NAME        compound 17e
EXPNO                  1
PROCNO                 1

F2 - Acquisition Parameters
Date_           20190715
Time               13.26 h
INSTRUM            spect
PROBHD   Z800701_0108 (
PULPROG             zg30
TD                 65536
SOLVENT            CDCl3
NS                    16
DS                     2
SWH            10000.000 Hz
FIDRES          0.305176 Hz
AQ             3.2767999 sec
RG                198.17
DW                50.000 usec
DE                 13.89 usec
TE                 298.0 K
D1            1.00000000 sec
TD0                    1
SFO1         500.1330883 MHz
NUC1                  1H
P0                  3.33 usec
P1                 10.00 usec
PLW1         26.00000000 W

F2 - Processing parameters
SI                 65536
SF           500.1300124 MHz
WDW                   EM
SSB      0
LB                  0.30 Hz
GB       0
PC                  1.00
```

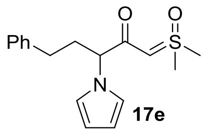

17e

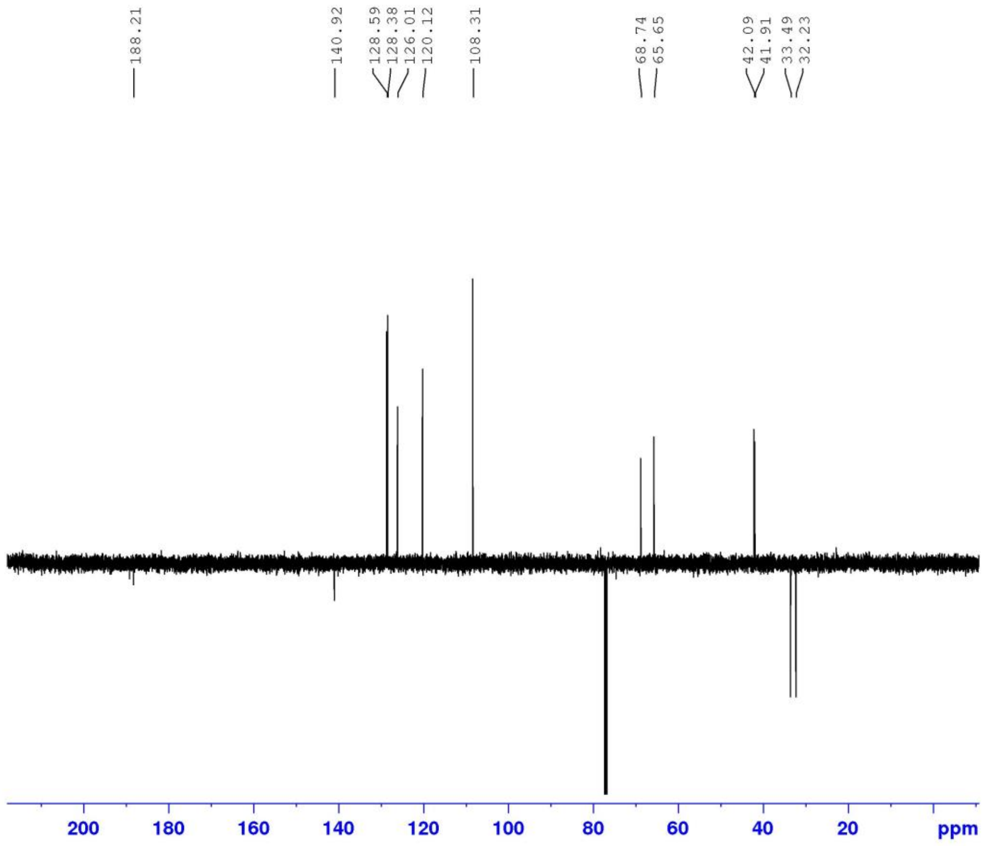


```
Current Data Parameters
NAME        compound 17e
EXPNO                  2
PROCNO                 1

F2 - Acquisition Parameters
Date_           20190715
Time               15.26 h
INSTRUM            spect
PROBHD   Z800701_0108 (
PULPROG             jmod
TD                 65536
SOLVENT            CDCl3
NS                   188
DS                     4
SWH            29761.904 Hz
FIDRES          0.908261 Hz
AQ             1.1010048 sec
RG                198.17
DW                16.800 usec
DE                  6.50 usec
TE                 298.0 K
CNST2        145.0000000
CNST11         1.0000000
D1            2.00000000 sec
D20           0.00689655 sec
TD0                    1
SFO1         125.7703643 MHz
NUC1                 13C
P1                  8.00 usec
P2                 16.00 usec
PLW1         53.00000000 W
SFO2         500.1320005 MHz
NUC2                  1H
CPDPRG[2         waltz65
PCPD2              80.00 usec
PLW2         28.00000000 W
PLW12         0.42017001 W

F2 - Processing parameters
SI                 32768
SF           125.7577919 MHz
WDW                   EM
SSB      0
LB                  1.00 Hz
GB       0
PC                  1.40
```

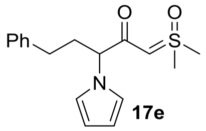

17e

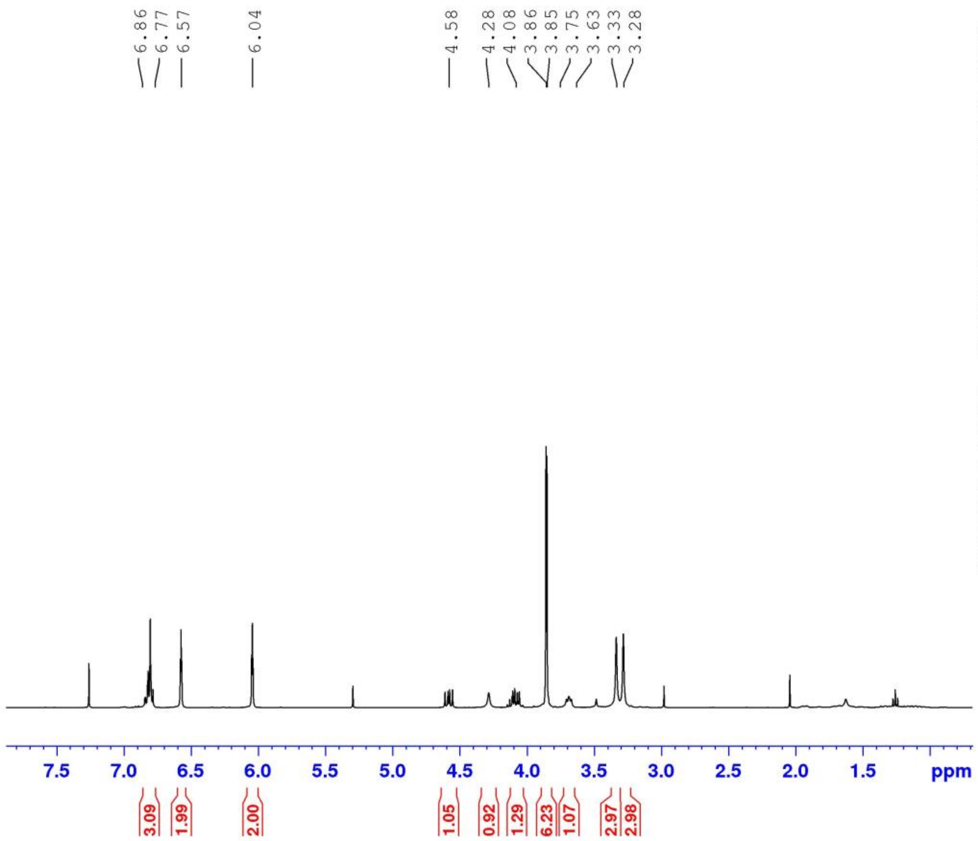


```
Current Data Parameters
NAME          compound 17f
EXPNO                    1
PROCNO                   1

F2 - Acquisition Parameters
Date_             20180313
Time                  9.23
INSTRUM              spect
PROBHD   5 mm QNP 1H/13
PULPROG               zg30
TD                   65536
SOLVENT              CDCl3
NS                      16
DS                       2
SWH               8278.146 Hz
FIDRES            0.126314 Hz
AQ               3.9583745 sec
RG                   228.1
DW                  60.400 usec
DE                    6.50 usec
TE                   298.0 K
D1              1.00000000 sec
TD0                      1

======== CHANNEL f1 ========
NUC1                    1H
P1                   10.00 usec
PL1                   1.00 dB
SFO1           400.1324710 MHz

F2 - Processing parameters
SI                   32768
SF             400.1300094 MHz
WDW                     EM
SSB      0
LB                    0.30 Hz
GB       0
PC                    1.00
```

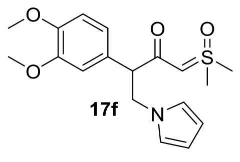

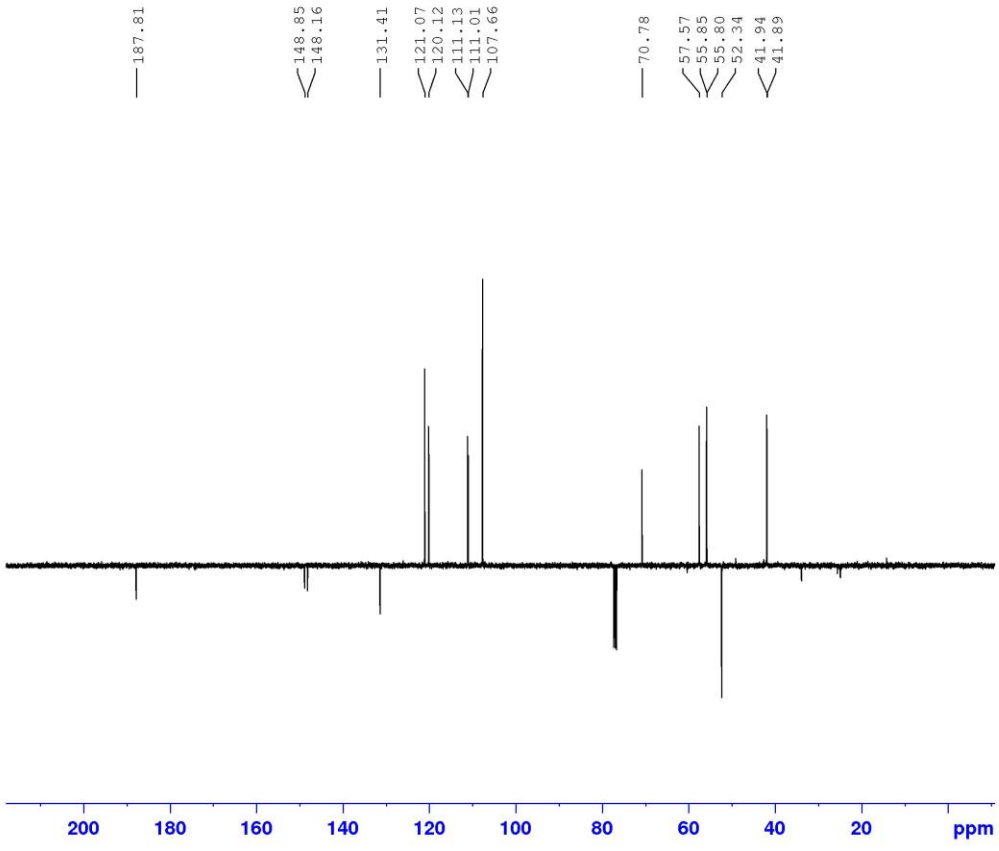


```
Current Data Parameters
NAME          compound 17f
EXPNO                    2
PROCNO                   1

F2 - Acquisition Parameters
Date_             20180313
Time                 10.12
INSTRUM              spect
PROBHD   5 mm QNP 1H/13
PULPROG               jmod
TD                   65536
SOLVENT              CDCl3
NS                     512
DS                       4
SWH              23980.814 Hz
FIDRES            0.365918 Hz
AQ               1.3664256 sec
RG                   16384
DW                  20.850 usec
DE                    6.50 usec
TE                   298.0 K
CNST2          145.0000000
CNST11           1.0000000
D1              2.00000000 sec
D20             0.00689655 sec
TD0                      1

======== CHANNEL f1 ========
NUC1                   13C
P1                    8.00 usec
P2                   16.00 usec
PL1      0 dB
SFO1           100.6228298 MHz

======== CHANNEL f2 ========
CPDPRG[2           waltz16
NUC2                    1H
PCPD2                80.00 usec
PL2                   1.00 dB
PL12                 19.06 dB
SFO2           400.1316005 MHz

F2 - Processing parameters
SI                   32768
SF             100.6127754 MHz
WDW                     EM
SSB      0
LB                    1.00 Hz
GB       0
PC                    1.40
```

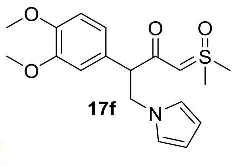

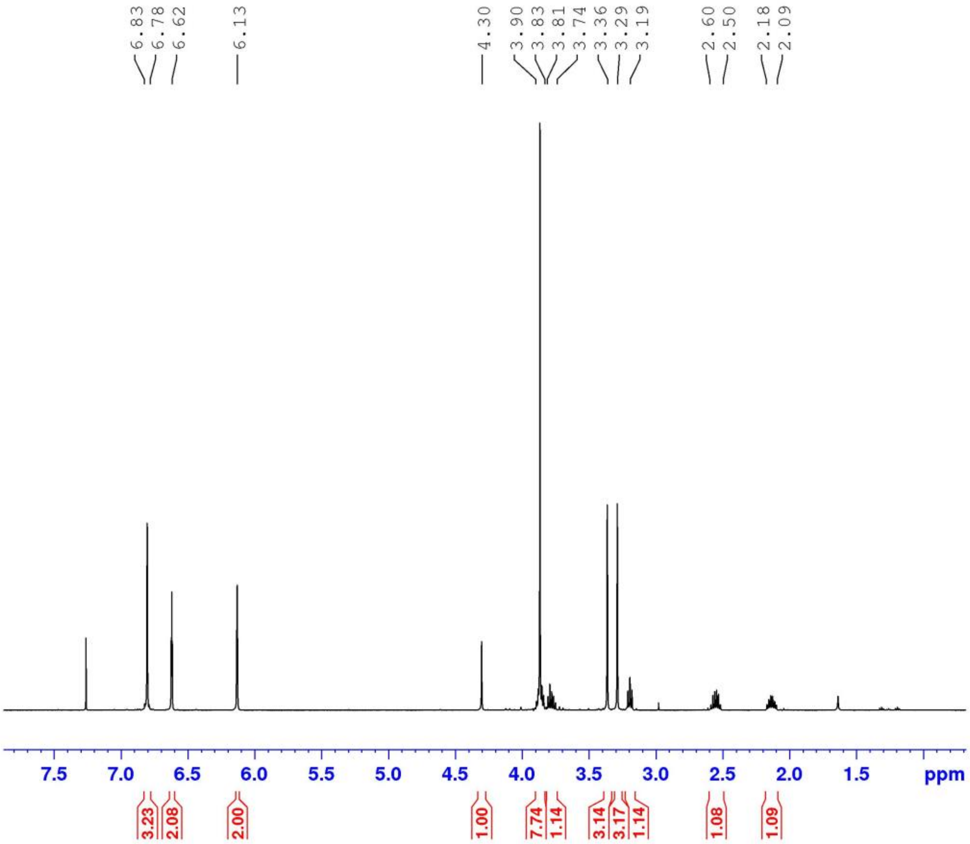
6.83
6.78
6.62
6.13
4.30
3.90
3.83
3.81
3.74
3.36
3.29
3.19
2.60
2.50
2.18
2.09
7.5 7.0 6.5 6.0 5.5 5.0 4.5 4.0 3.5 3.0 2.5 2.0 1.5 ppm
3.23
2.08
2.00
1.00
7.74
1.14
3.14
3.17
1.14
1.08
1.09
Current Data Parameters
NAME compound 17g
EXPNO 1
PROCNO 1
F2 - Acquisition Parameters
Date_ 20180725
Time 9.38 h
INSTRUM spect
PROBHD Z800701_0108 (
PULPROG zg30
TD 65536
SOLVENT CDCl3
NS 16
DS 2
SWH 10000.000 Hz
FIDRES 0.305176 Hz
AQ 3.2767999 sec
RG 198.17
DW 50.000 usec
DE 6.50 usec
TE 298.0 K
D1 1.00000000 sec
TD0 1
SFO1 500.1330883 MHz
NUC1 1H
P1 10.00 usec
PLW1 26.00000000 W
F2 - Processing parameters
SI 65536
SF 500.1300124 MHz
WDW EM
SSB 0
LB 0.30 Hz
GB 0
PC 1.00

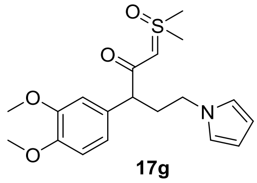
O
S
O
O
O
N
17g

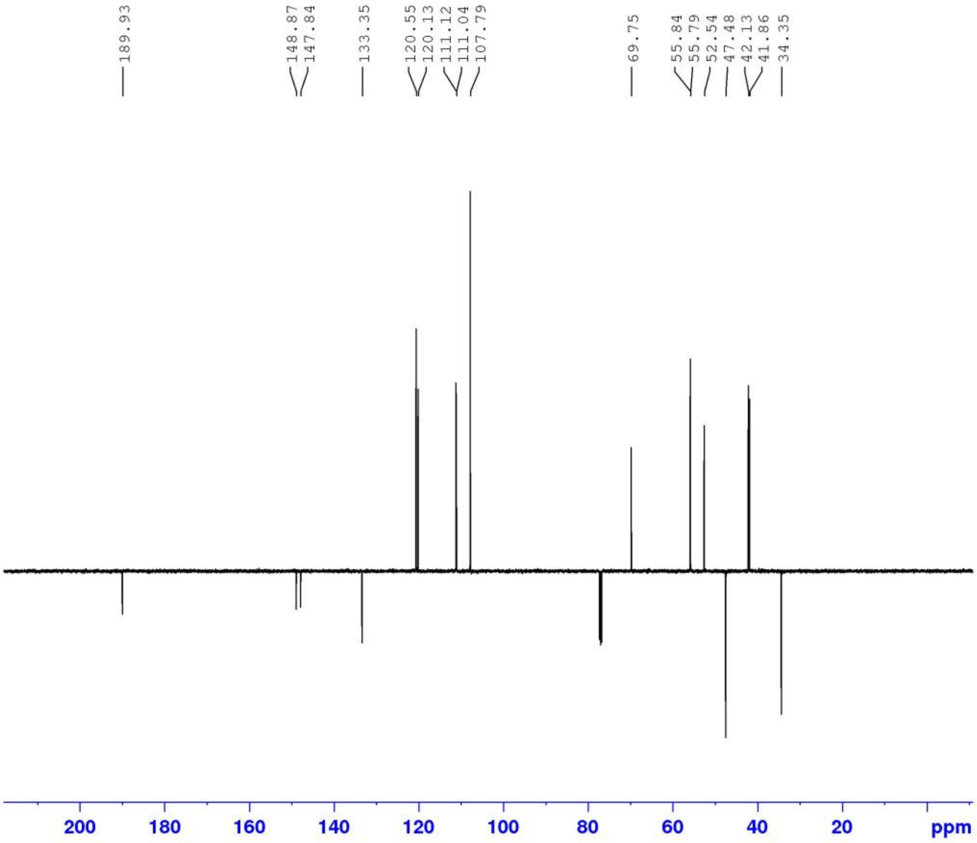
189.93
148.87
147.84
133.35
120.55
120.13
111.12
111.04
107.79
69.75
55.84
55.79
52.54
47.48
42.13
41.86
34.35
200 180 160 140 120 100 80 60 40 20 ppm
Current Data Parameters
NAME compound 17g
EXPNO 2
PROCNO 1
F2 - Acquisition Parameters
Date_ 20180725
Time 12.02 h
INSTRUM spect
PROBHD Z800701_0108 (
PULPROG jmod
TD 65536
SOLVENT CDCl3
NS 512
DS 4
SWH 29761.904 Hz
FIDRES 0.908261 Hz
AQ 1.1010048 sec
RG 198.17
DW 16.800 usec
DE 6.50 usec
TE 298.0 K
CNST2 145.0000000
CNST11 1.0000000
D1 2.00000000 sec
D20 0.00689655 sec
TD0 1
SFO1 125.7703643 MHz
NUC1 13C
P1 8.00 usec
P2 16.00 usec
PLW1 53.00000000 W
SFO2 500.1320005 MHz
NUC2 1H
CPDPRG[2 waltz16
PCPD2 80.00 usec
PLW2 28.00000000 W
PLW12 0.42017001 W
F2 - Processing parameters
SI 32768
SF 125.7577999 MHz
WDW EM
SSB 0
LB 1.00 Hz
GB 0
PC 1.40

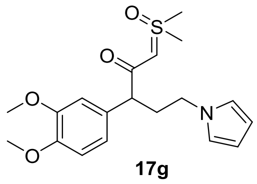
O
S
O
O
O
N
17g

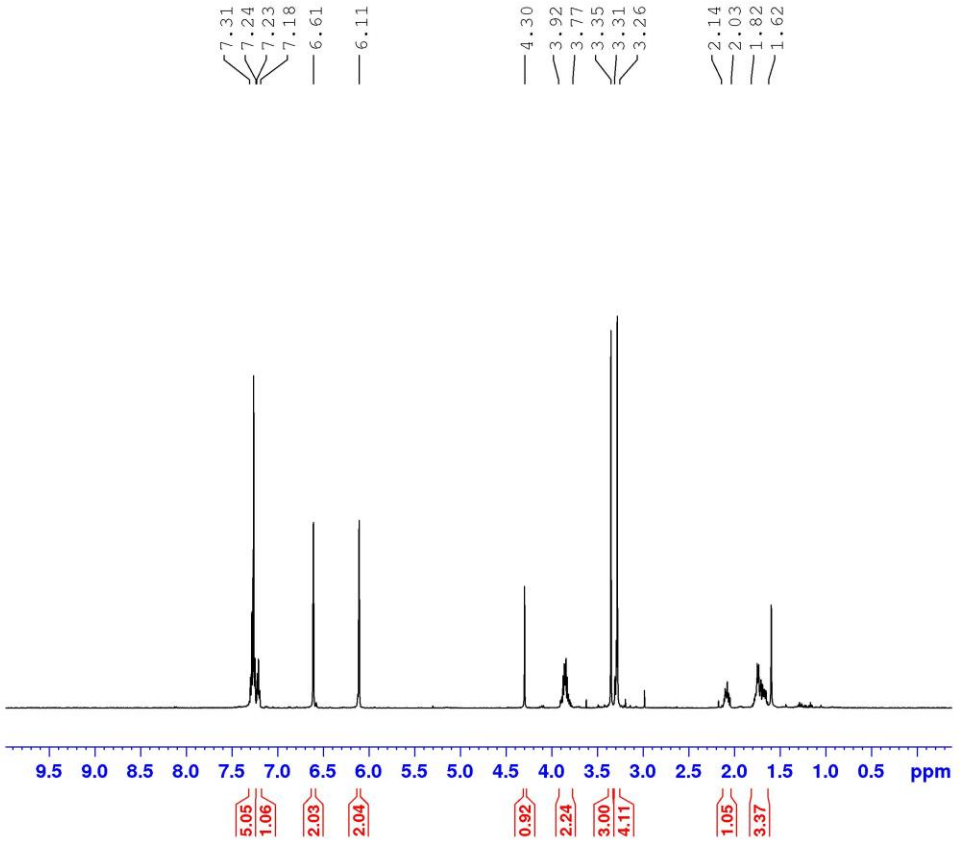


```
Current Data Parameters
NAME       compound 17 h
EXPNO                  4
PROCNO                 1

F2 - Acquisition Parameters
Date_           20190816
Time             17.01 h
INSTRUM            spect
PROBHD   Z800701_0108 (
PULPROG             zg30
TD                 65536
SOLVENT            CDCl3
NS                    16
DS                     2
SWH          10000.000 Hz
FIDRES        0.305176 Hz
AQ           3.2767999 sec
RG              198.17
DW              50.000 usec
DE               13.89 usec
TE               298.0 K
D1          1.00000000 sec
TD0                    1
SFO1       500.1330883 MHz
NUC1                  1H
P0                  3.33 usec
P1                 10.00 usec
PLW1       26.00000000 W

F2 - Processing parameters
SI                 65536
SF         500.1300121 MHz
WDW                   EM
SSB      0
LB                  0.30 Hz
GB       0
PC                  1.00
```

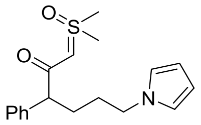

17h

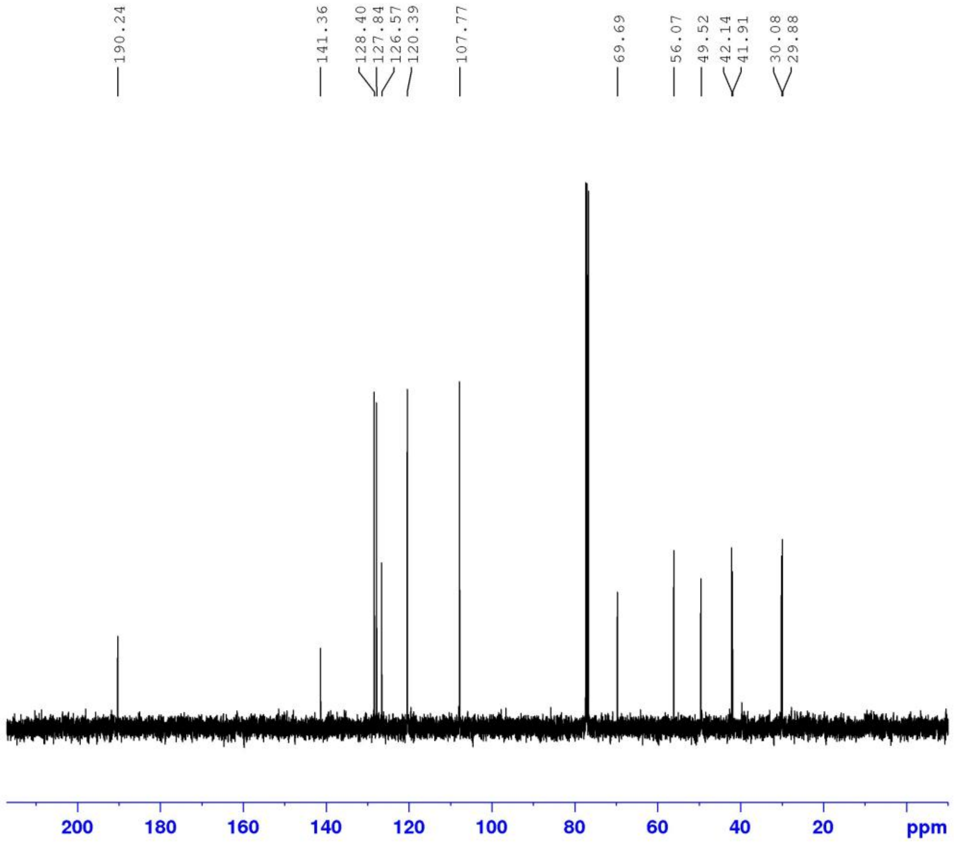


```
Current Data Parameters
NAME        Compound 17h
EXPNO                  2
PROCNO                 1

F2 - Acquisition Parameters
Date_           20190723
Time                9.30
INSTRUM            spect
PROBHD   5 mm QNP  1H/1
PULPROG           zgpg30
TD                 65536
SOLVENT            CDCl3
NS                    76
DS                     4
SWH            23980.814 Hz
FIDRES          0.365918 Hz
AQ             1.3664256 sec
RG                 228.1
DW                20.850 usec
DE                  6.50 usec
TE                 298.0 K
D1            2.00000000 sec
D11           0.03000000 sec
TD0                    1

======== CHANNEL f1 ========
NUC1                 13C
P1                  6.50 usec
PL1      0 dB
SFO1         100.6228298 MHz

======== CHANNEL f2 ========
CPDPRG[2         waltz16
NUC2                  1H
PCPD2              80.00 usec
PL2                 1.70 dB
PL12               19.76 dB
PL13               21.00 dB
SFO2         400.1316005 MHz

F2 - Processing parameters
SI                 32768
SF           100.6127781 MHz
WDW                   EM
SSB      0
LB                  1.00 Hz
GB       0
PC                  1.40
```

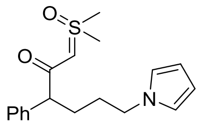

17h

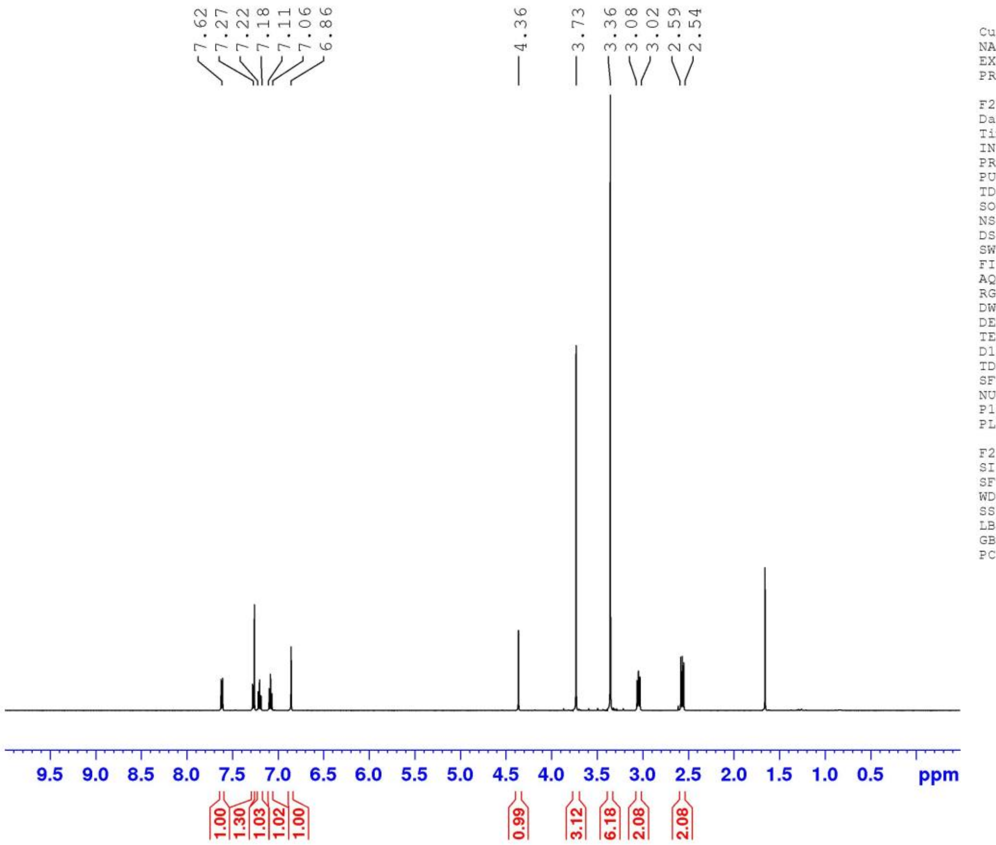

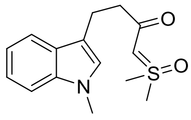

19

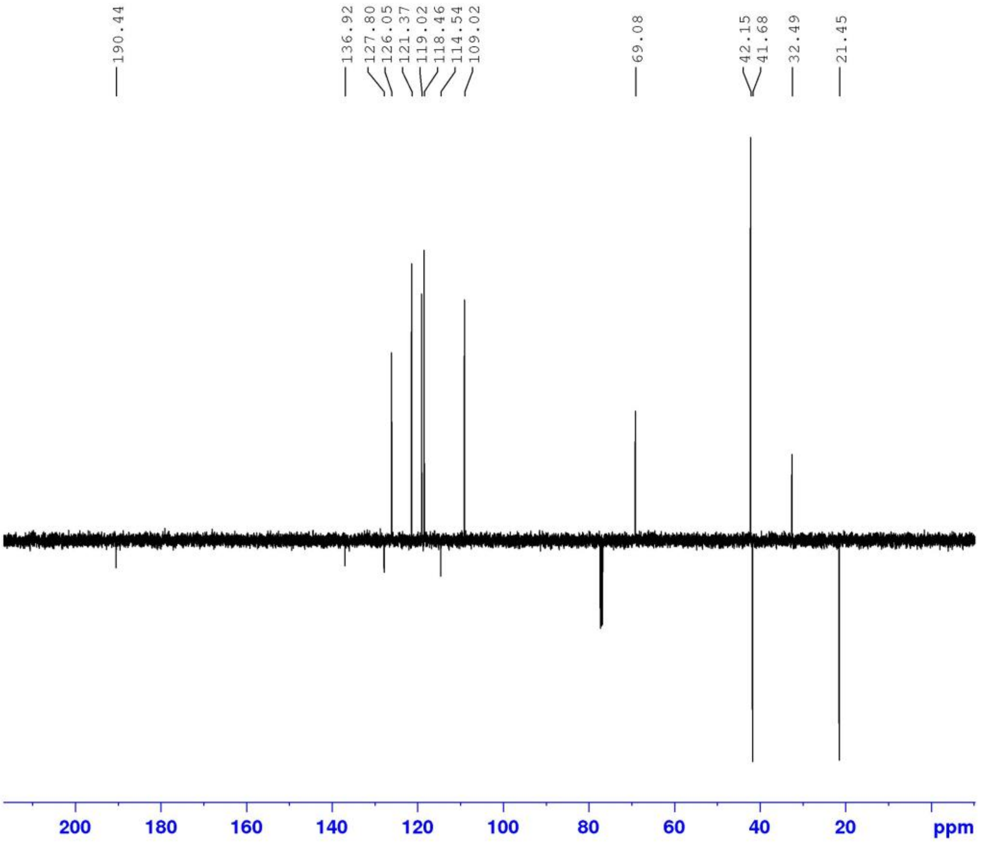

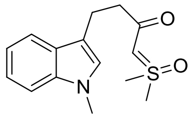

19

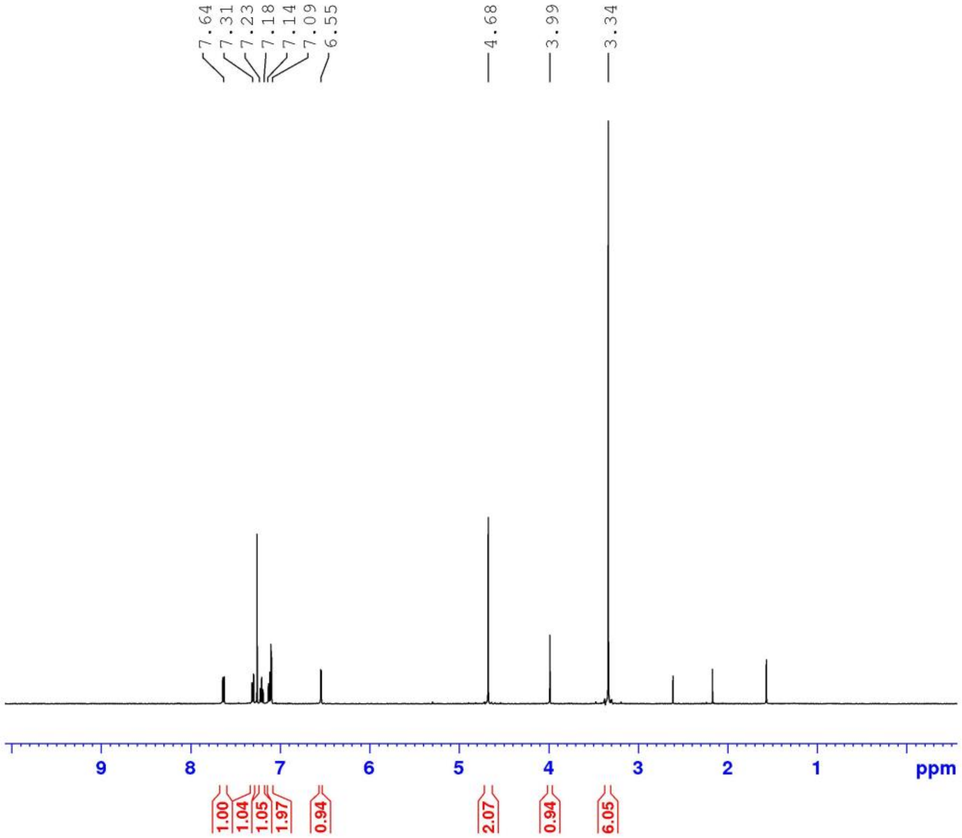


```
Current Data Parameters
NAME          Compound 21
EXPNO                   1
PROCNO                  1

F2 - Acquisition Parameters
Date_            20190502
Time                 9.01 h
INSTRUM             spect
PROBHD   Z800701_0108 (
PULPROG              zg30
TD                  65536
SOLVENT             CDCl3
NS                     16
DS                      2
SWH             10000.000 Hz
FIDRES           0.305176 Hz
AQ              3.2767999 sec
RG                 198.17
DW                 50.000 usec
DE                   6.50 usec
TE                  298.0 K
D1             1.00000000 sec
TD0                     1
SFO1          500.1330883 MHz
NUC1                   1H
P1                  10.00 usec
PLW1          26.00000000 W

F2 - Processing parameters
SI                  65536
SF            500.1300122 MHz
WDW                    EM
SSB      0
LB                   0.30 Hz
GB       0
PC                   1.00
```

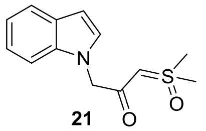

21

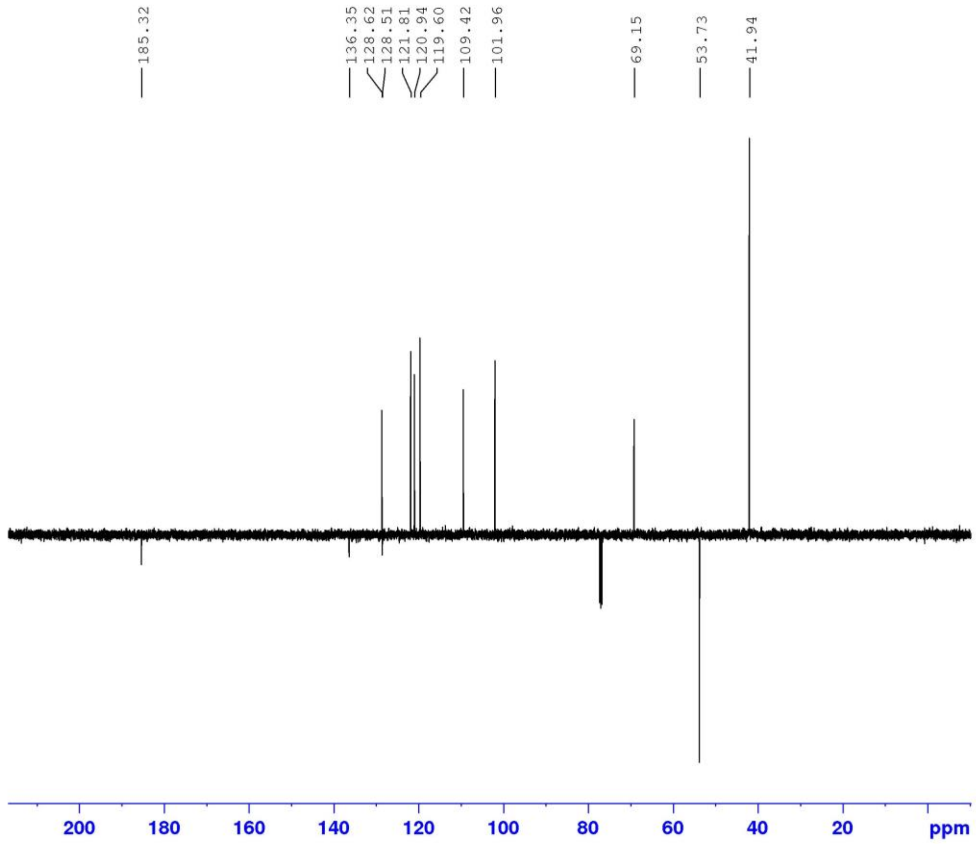


```
Current Data Parameters
NAME          Compound 21
EXPNO                   2
PROCNO                  1

F2 - Acquisition Parameters
Date_            20190502
Time                 9.15 h
INSTRUM             spect
PROBHD   Z800701_0108 (
PULPROG              jmod
TD                  65536
SOLVENT             CDCl3
NS                     63
DS                      4
SWH             29761.904 Hz
FIDRES           0.908261 Hz
AQ              1.1010048 sec
RG                 198.17
DW                 16.800 usec
DE                   6.50 usec
TE                  298.0 K
CNST2         145.0000000
CNST11          1.0000000
D1             2.00000000 sec
D20            0.00689655 sec
TD0                     1
SFO1          125.7703643 MHz
NUC1                  13C
P1                   8.00 usec
P2                  16.00 usec
PLW1          53.00000000 W
SFO2          500.1320005 MHz
NUC2                   1H
CPDPRG[2          waltz16
PCPD2               80.00 usec
PLW2          28.00000000 W
PLW12          0.42017001 W

F2 - Processing parameters
SI                  32768
SF            125.7577978 MHz
WDW                    EM
SSB      0
LB                   1.00 Hz
GB       0
PC                   1.40
```

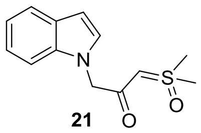

21

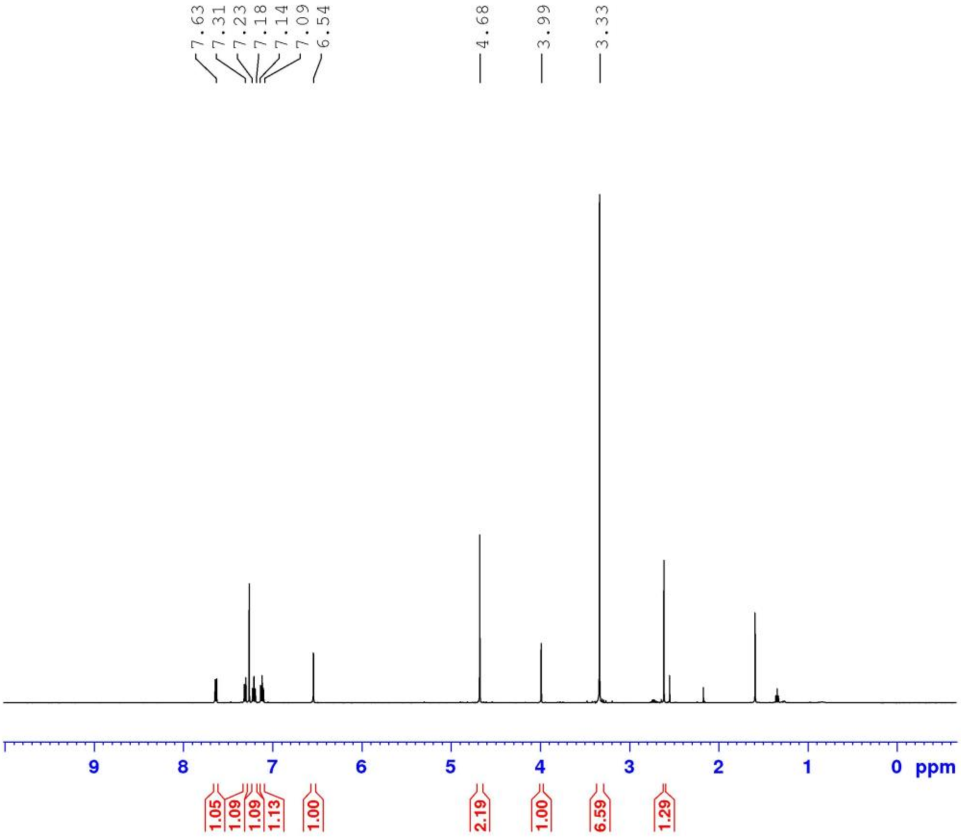


```
Current Data Parameters
NAME      compound D-21
EXPNO              1
PROCNO             1

F2 - Acquisition Parameters
Date_          20190531
Time              13.15 h
INSTRUM           spect
PROBHD   Z800701_0108 (
PULPROG            zg30
TD                65536
SOLVENT           CDCl3
NS                   16
DS                    2
SWH          10000.000 Hz
FIDRES        0.305176 Hz
AQ           3.2767999 sec
RG               198.17
DW               50.000 usec
DE                 6.50 usec
TE                298.0 K
D1          1.00000000 sec
TD0                   1
SFO1       500.1330883 MHz
NUC1                 1H
P1                10.00 usec
PLW1        26.00000000 W

F2 - Processing parameters
SI                65536
SF         500.1300120 MHz
WDW                  EM
SSB      0
LB                 0.30 Hz
GB       0
PC                 1.40
```

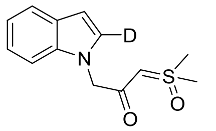

[D$_1$]-21

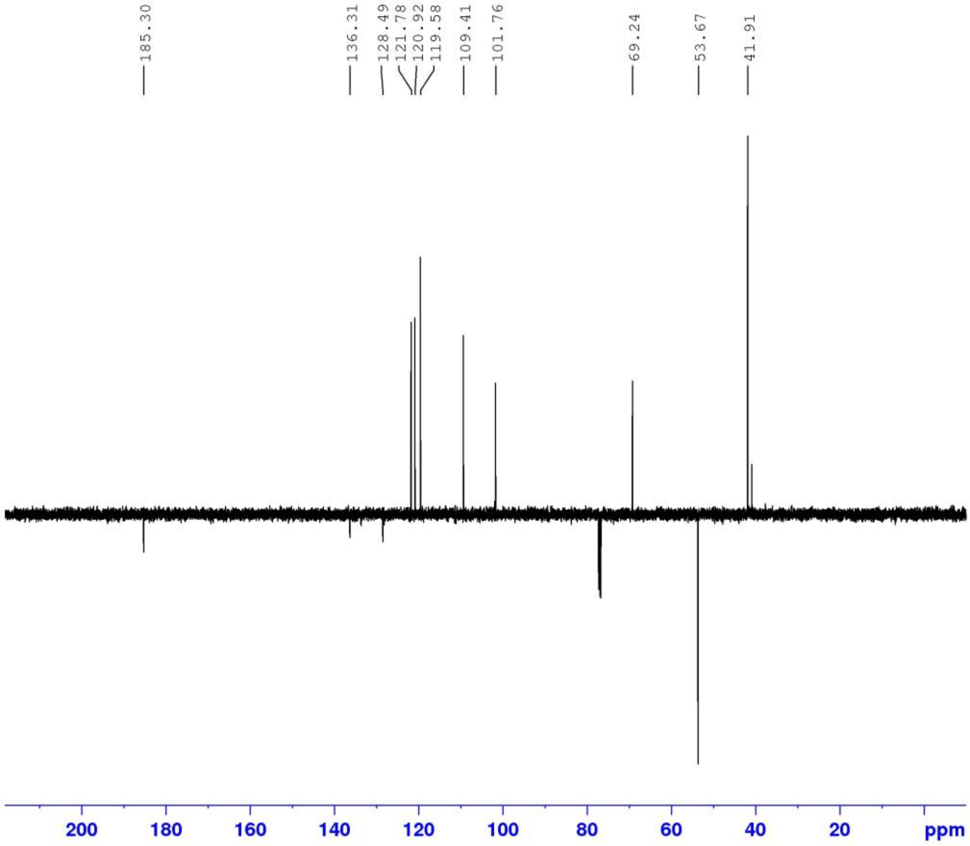


```
Current Data Parameters
NAME      compound D-21
EXPNO              2
PROCNO             1

F2 - Acquisition Parameters
Date_          20190531
Time              13.27 h
INSTRUM           spect
PROBHD   Z800701_0108 (
PULPROG            jmod
TD                65536
SOLVENT           CDCl3
NS                   60
DS                    4
SWH          29761.904 Hz
FIDRES        0.908261 Hz
AQ           1.1010048 sec
RG               198.17
DW               16.800 usec
DE                 6.50 usec
TE                298.4 K
CNST2       145.0000000
CNST11        1.0000000
D1          2.00000000 sec
D20         0.00689655 sec
TD0                   1
SFO1       125.7703643 MHz
NUC1                13C
P1                 8.00 usec
P2                16.00 usec
PLW1        53.00000000 W
SFO2       500.1320005 MHz
NUC2                 1H
CPDPRG[2        waltz16
PCPD2             80.00 usec
PLW2        28.00000000 W
PLW12        0.42017001 W

F2 - Processing parameters
SI                32768
SF         125.7577991 MHz
WDW                  EM
SSB      0
LB                 1.00 Hz
GB       0
PC                 1.40
```

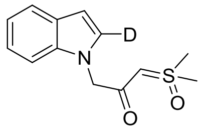

[D$_1$]-21

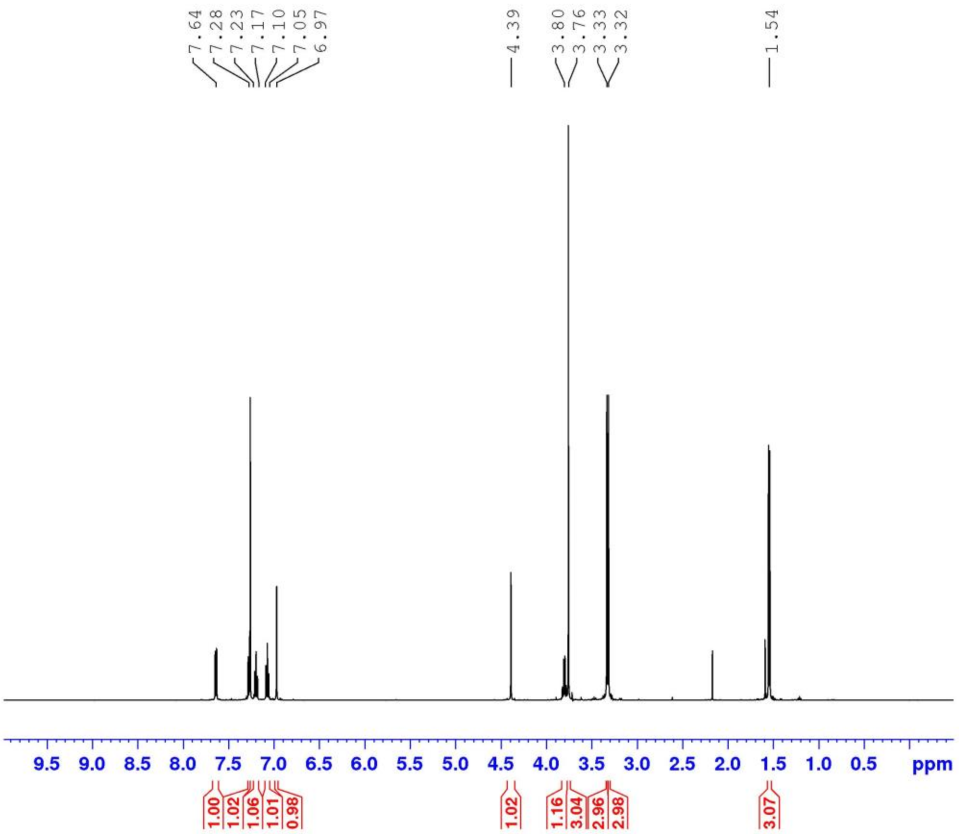


```
Current Data Parameters
NAME          Compound 23
EXPNO                   2
PROCNO                  1

F2 - Acquisition Parameters
Date_            20190325
Time               18.26 h
INSTRUM             spect
PROBHD   Z800701_0108 (
PULPROG              zg30
TD                  65536
SOLVENT             CDCl3
NS                     16
DS                      2
SWH             10000.000 Hz
FIDRES           0.305176 Hz
AQ              3.2767999 sec
RG                 198.17
DW                 50.000 usec
DE                   6.50 usec
TE                  298.0 K
D1             1.00000000 sec
TD0                     1
SFO1          500.1330883 MHz
NUC1                   1H
P1                  10.00 usec
PLW1          26.00000000 W

F2 - Processing parameters
SI                  65536
SF            500.1300121 MHz
WDW                    EM
SSB      0
LB                   0.30 Hz
GB       0
PC                   1.40
```

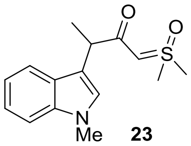

23

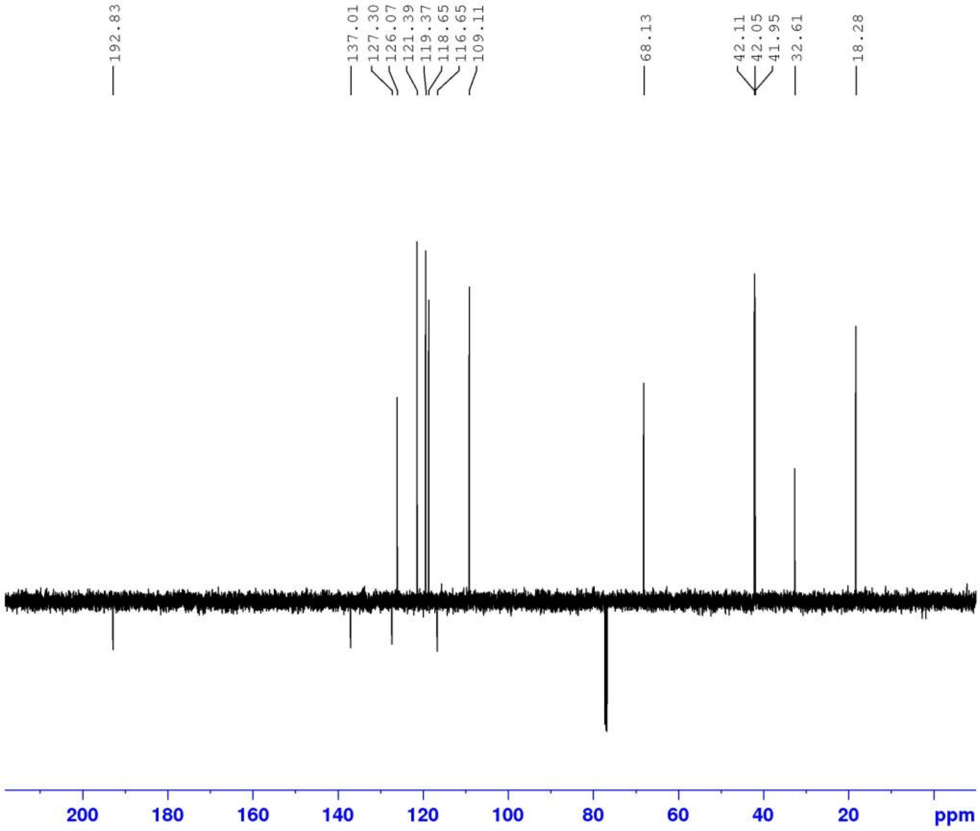


```
Current Data Parameters
NAME          Compound 23
EXPNO                   3
PROCNO                  1

F2 - Acquisition Parameters
Date_            20190325
Time               18.37 h
INSTRUM             spect
PROBHD   Z800701_0108 (
PULPROG              jmod
TD                  65536
SOLVENT             CDCl3
NS                     52
DS                      4
SWH             29761.904 Hz
FIDRES           0.908261 Hz
AQ              1.1010048 sec
RG                 198.17
DW                 16.800 usec
DE                   6.50 usec
TE                  298.0 K
CNST2         145.0000000
CNST11          1.0000000
D1             2.00000000 sec
D20            0.00689655 sec
TD0                     1
SFO1          125.7703643 MHz
NUC1                  13C
P1                   8.00 usec
P2                  16.00 usec
PLW1          53.00000000 W
SFO2          500.1320005 MHz
NUC2                   1H
CPDPRG[2          waltz16
PCPD2               80.00 usec
PLW2          28.00000000 W
PLW12          0.42017001 W

F2 - Processing parameters
SI                  32768
SF            125.7577983 MHz
WDW                    EM
SSB      0
LB                   1.00 Hz
GB       0
PC                   1.40
```

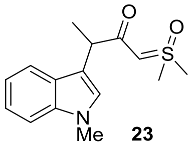

23

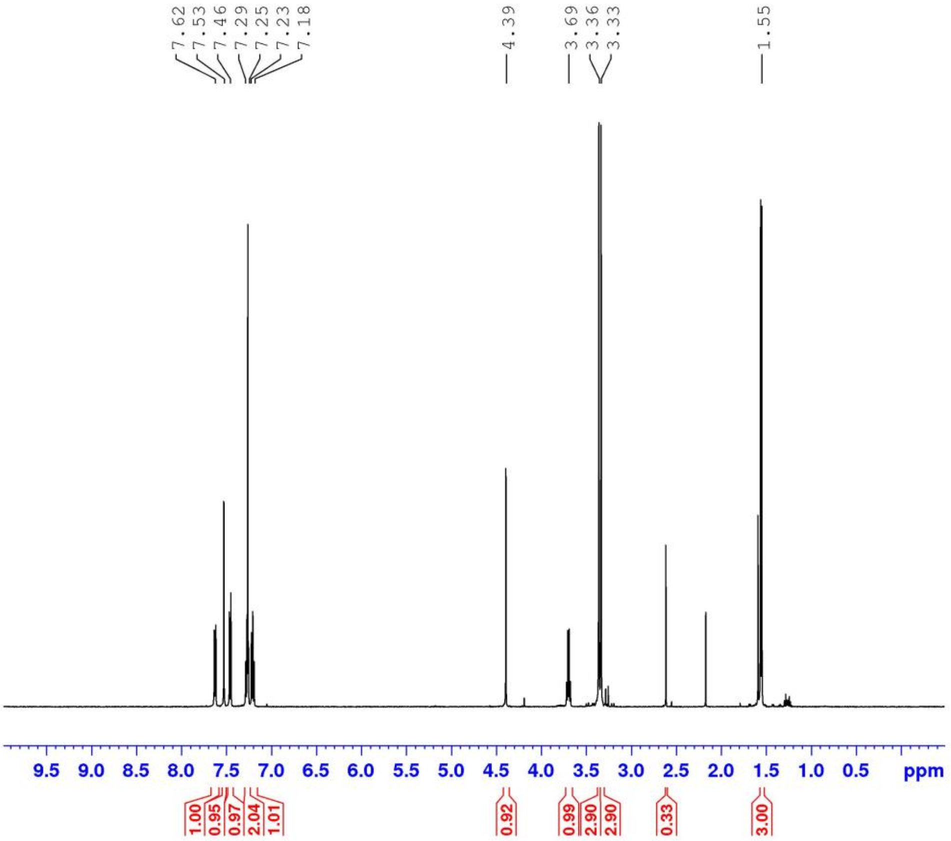


```
Current Data Parameters
NAME          Compound 25
EXPNO                   1
PROCNO                  1

F2 - Acquisition Parameters
Date_            20190510
Time                11.25 h
INSTRUM             spect
PROBHD   Z800701_0108 (
PULPROG              zg30
TD                  65536
SOLVENT             CDCl3
NS                     16
DS                      2
SWH             10000.000 Hz
FIDRES           0.305176 Hz
AQ              3.2767999 sec
RG                 198.17
DW                 50.000 usec
DE                   6.50 usec
TE                  298.0 K
D1             1.00000000 sec
TD0                     1
SFO1          500.1330883 MHz
NUC1                   1H
P1                  10.00 usec
PLW1          26.00000000 W

F2 - Processing parameters
SI                  65536
SF            500.1300119 MHz
WDW                    EM
SSB       0
LB                   0.30 Hz
GB        0
PC                   1.00
```

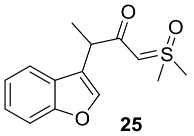

25

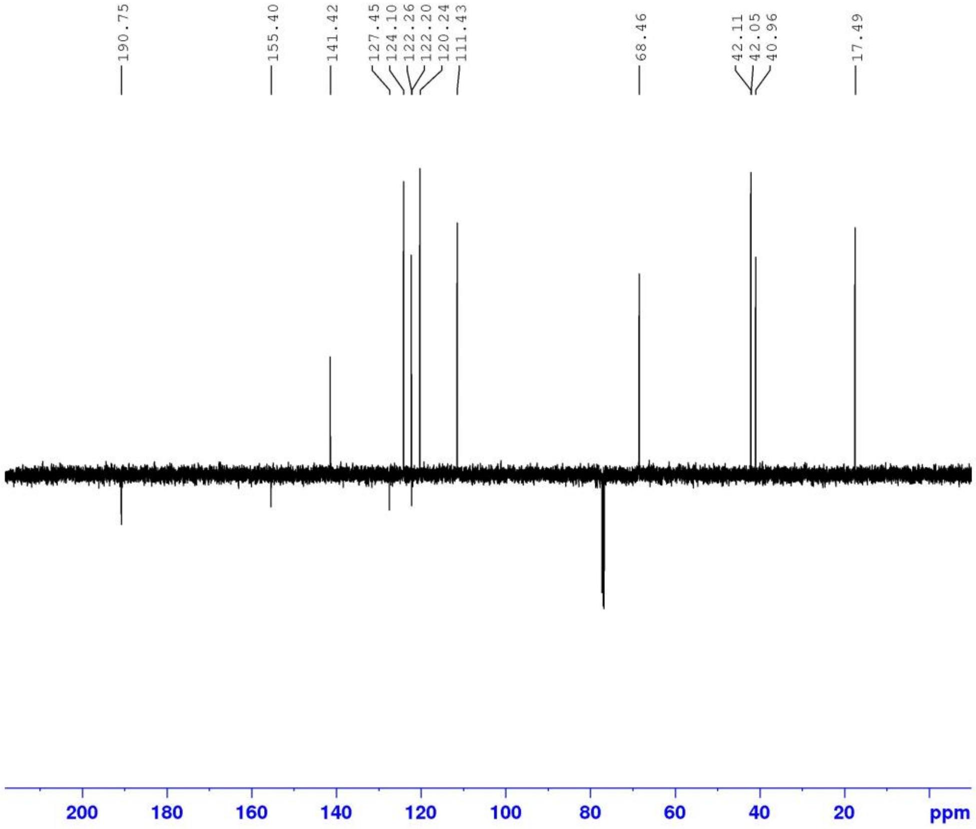


```
Current Data Parameters
NAME          Compound 25
EXPNO                   2
PROCNO                  1

F2 - Acquisition Parameters
Date_            20190510
Time                11.37 h
INSTRUM             spect
PROBHD   Z800701_0108 (
PULPROG              jmod
TD                  65536
SOLVENT             CDCl3
NS                     72
DS                      4
SWH             29761.904 Hz
FIDRES           0.908261 Hz
AQ              1.1010048 sec
RG                 198.17
DW                 16.800 usec
DE                   6.50 usec
TE                  298.0 K
CNST2         145.0000000
CNST11          1.0000000
D1             2.00000000 sec
D20            0.00689655 sec
TD0                     1
SFO1          125.7703643 MHz
NUC1                  13C
P1                   8.00 usec
P2                  16.00 usec
PLW1          53.00000000 W
SFO2          500.1320005 MHz
NUC2                   1H
CPDPRG[2          waltz16
PCPD2               80.00 usec
PLW2          28.00000000 W
PLW12          0.42017001 W

F2 - Processing parameters
SI                  32768
SF            125.7577956 MHz
WDW                    EM
SSB       0
LB                   1.00 Hz
GB        0
PC                   1.40
```

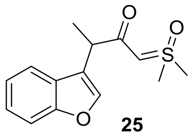

25

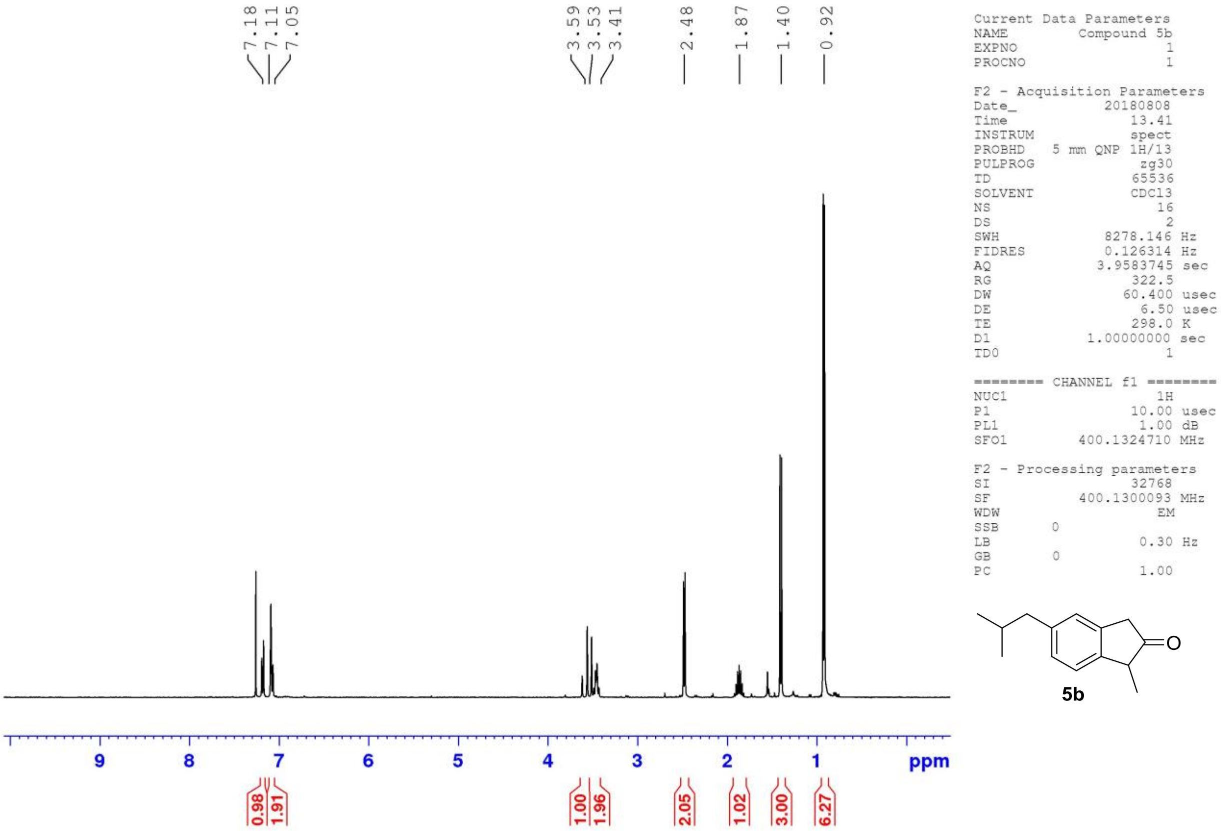
7.18
7.11
7.05
3.59
3.53
3.41
2.48
1.87
1.40
0.92
Current Data Parameters
NAME Compound 5b
EXPNO 1
PROCNO 1
F2 - Acquisition Parameters
Date_ 20180808
Time 13.41
INSTRUM spect
PROBHD 5 mm QNP 1H/13
PULPROG zg30
TD 65536
SOLVENT CDCl3
NS 16
DS 2
SWH 8278.146 Hz
FIDRES 0.126314 Hz
AQ 3.9583745 sec
RG 322.5
DW 60.400 usec
DE 6.50 usec
TE 298.0 K
D1 1.00000000 sec
TD0 1
======== CHANNEL f1 ========
NUC1 1H
P1 10.00 usec
PL1 1.00 dB
SFO1 400.1324710 MHz
F2 - Processing parameters
SI 32768
SF 400.1300093 MHz
WDW EM
SSB 0
LB 0.30 Hz
GB 0
PC 1.00
5b
9
8
7
6
5
4
3
2
1
ppm
0.98
1.91
1.00
1.96
2.05
1.02
3.00
6.27

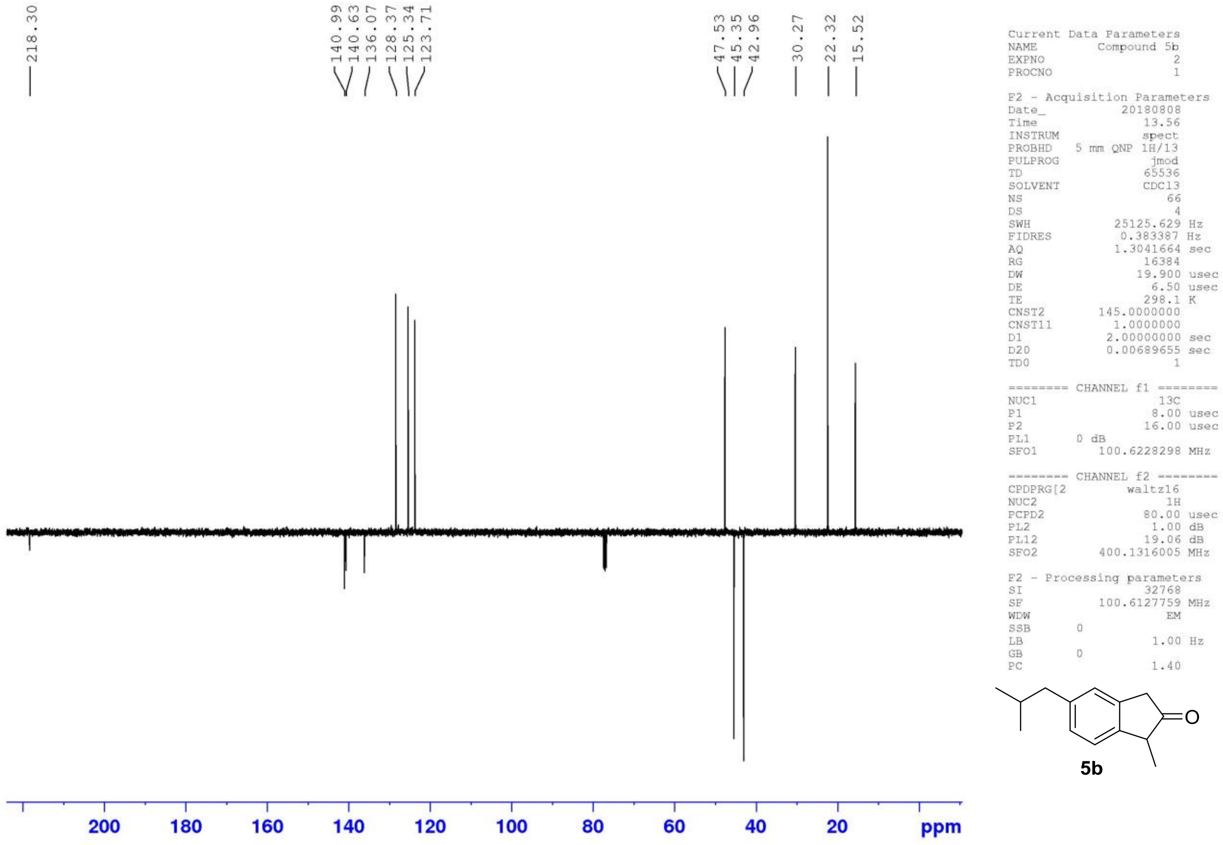
218.30
140.99
140.63
136.07
128.37
125.34
123.71
47.53
45.35
42.96
30.27
22.32
15.52
Current Data Parameters
NAME Compound 5b
EXPNO 2
PROCNO 1
F2 - Acquisition Parameters
Date_ 20180808
Time 13.56
INSTRUM spect
PROBHD 5 mm QNP 1H/13
PULPROG jmod
TD 65536
SOLVENT CDCl3
NS 66
DS 4
SWH 25125.629 Hz
FIDRES 0.383387 Hz
AQ 1.3041664 sec
RG 16384
DW 19.900 usec
DE 6.50 usec
TE 298.1 K
CNST2 145.0000000
CNST11 1.0000000
D1 2.00000000 sec
D20 0.00689655 sec
TD0 1
======== CHANNEL f1 ========
NUC1 13C
P1 8.00 usec
P2 16.00 usec
PL1 0 dB
SFO1 100.6228298 MHz
======== CHANNEL f2 ========
CPDPRG[2 waltz16
NUC2 1H
PCPD2 80.00 usec
PL2 1.00 dB
PL12 19.06 dB
SFO2 400.1316005 MHz
F2 - Processing parameters
SI 32768
SF 100.6127759 MHz
WDW EM
SSB 0
LB 1.00 Hz
GB 0
PC 1.40
5b
200
180
160
140
120
100
80
60
40
20
ppm

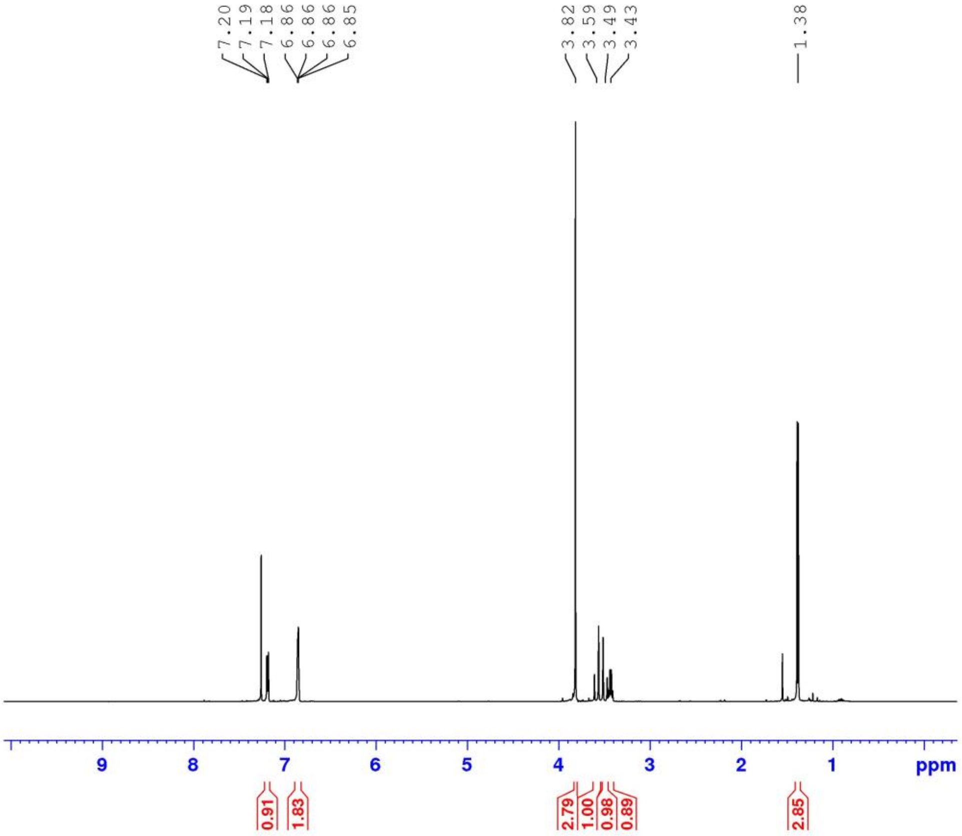

Current Data Parameters
NAME Compound 5c
EXPNO 1
PROCNO 1

F2 - Acquisition Parameters
Date_ 20180914
Time 11.46 h
INSTRUM spect
PROBHD Z800701_0108 (
PULPROG zg30
TD 65536
SOLVENT CDCl3
NS 16
DS 2
SWH 10000.000 Hz
FIDRES 0.305176 Hz
AQ 3.2767999 sec
RG 198.17
DW 50.000 usec
DE 6.50 usec
TE 298.0 K
D1 1.00000000 sec
TD0 1
SFO1 500.1330883 MHz
NUC1 1H
P1 10.00 usec
PLW1 26.00000000 W

F2 - Processing parameters
SI 65536
SF 500.1300124 MHz
WDW EM
SSB 0
LB 0.30 Hz
GB 0
PC 1.00

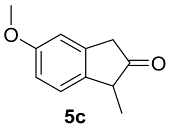

5c

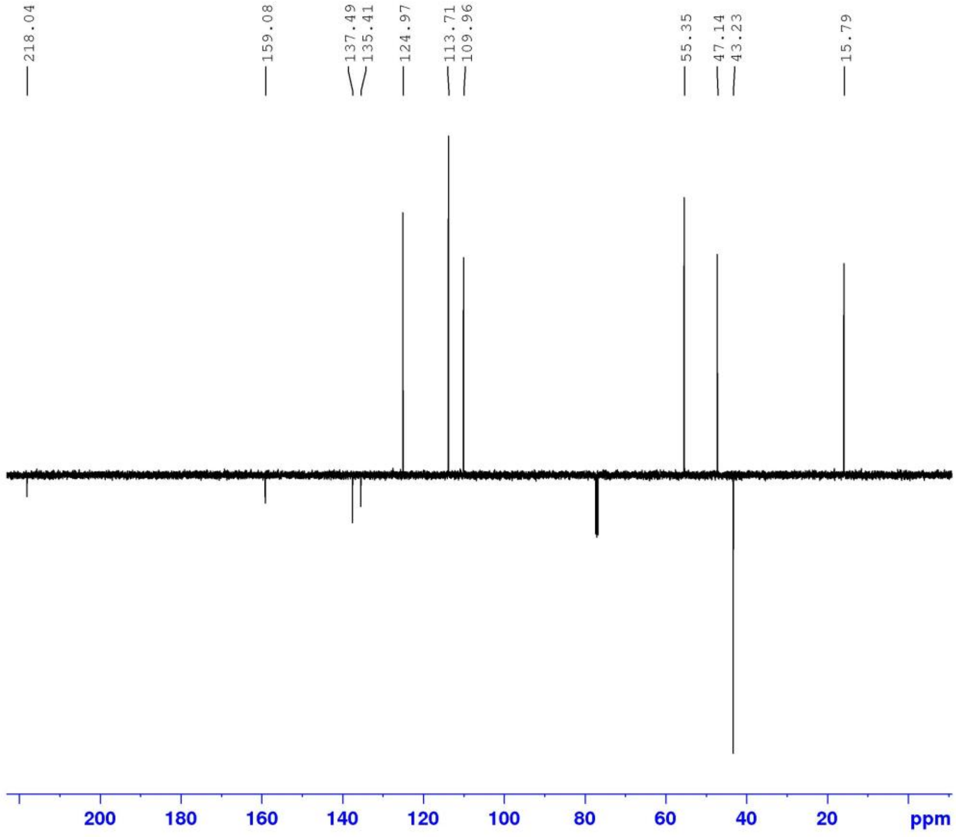

Current Data Parameters
NAME Compound 5c
EXPNO 2
PROCNO 1

F2 - Acquisition Parameters
Date_ 20180914
Time 11.59 h
INSTRUM spect
PROBHD Z800701_0108 (
PULPROG jmod
TD 65536
SOLVENT CDCl3
NS 63
DS 4
SWH 31250.000 Hz
FIDRES 0.953674 Hz
AQ 1.0485760 sec
RG 198.17
DW 16.000 usec
DE 6.50 usec
TE 298.0 K
CNST2 145.0000000
CNST11 1.0000000
D1 2.00000000 sec
D20 0.00689655 sec
TD0 1
SFO1 125.7703643 MHz
NUC1 13C
P1 8.00 usec
P2 16.00 usec
PLW1 53.00000000 W
SFO2 500.1320005 MHz
NUC2 1H
CPDPRG[2 waltz16
PCPD2 80.00 usec
PLW2 28.00000000 W
PLW12 0.42017001 W

F2 - Processing parameters
SI 32768
SF 125.7577971 MHz
WDW EM
SSB 0
LB 1.00 Hz
GB 0
PC 1.40

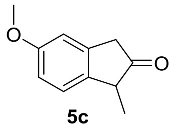

5c

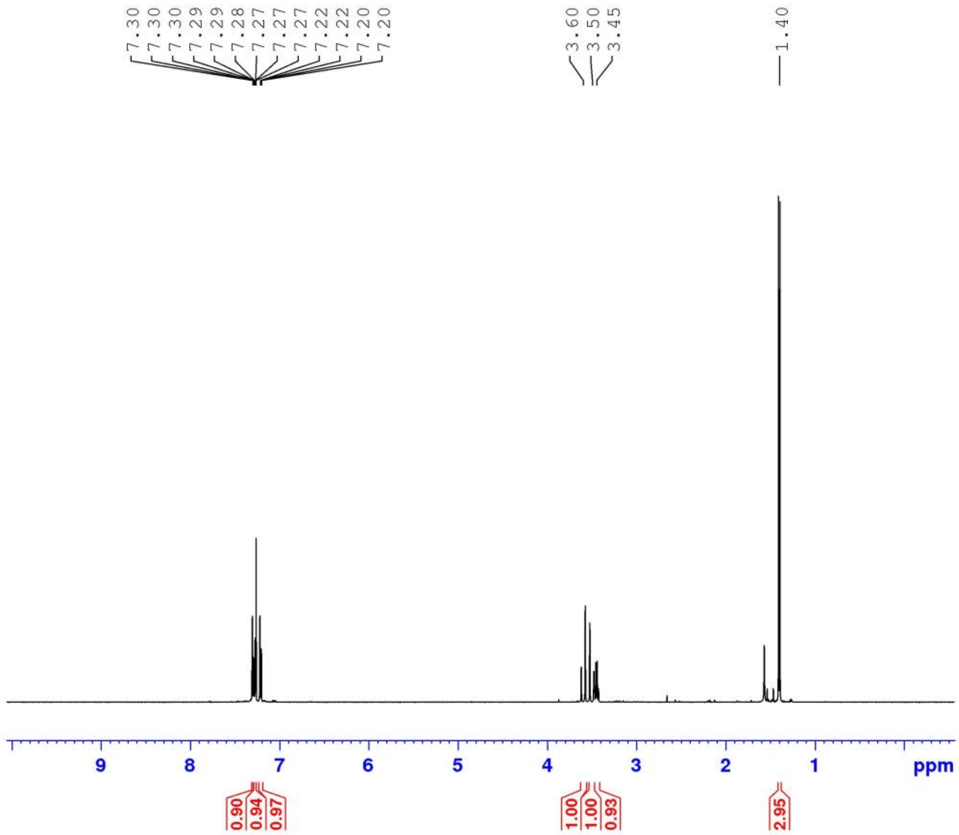

Current Data Parameters
NAME Compound 5d
EXPNO 1
PROCNO 1

F2 - Acquisition Parameters
Date_ 20170420
Time 16.59
INSTRUM spect
PROBHD 5 mm PABBO BB-
PULPROG zg30
TD 65536
SOLVENT CDCl3
NS 16
DS 2
SWH 10000.000 Hz
FIDRES 0.152588 Hz
AQ 3.2767999 sec
RG 198.17
DW 50.000 usec
DE 6.50 usec
TE 298.0 K
D1 1.00000000 sec
TD0 1

======== CHANNEL f1 ========
SFO1 500.1330885 MHz
NUC1 1H
P1 10.00 usec
PLW1 26.00000000 W

F2 - Processing parameters
SI 65536
SF 500.1300122 MHz
WDW EM
SSB 0
LB 0.30 Hz
GB 0
PC 1.00

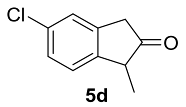

5d

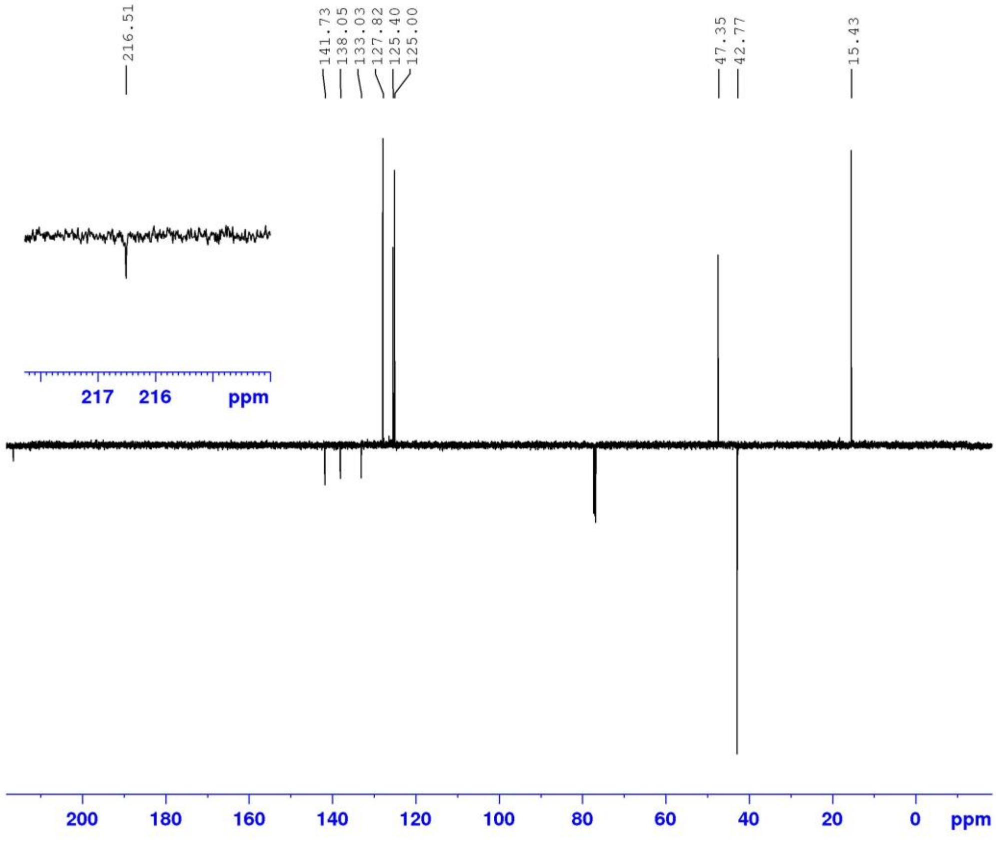

Current Data Parameters
NAME Compound 5 d
EXPNO 3
PROCNO 1

F2 - Acquisition Parameters
Date_ 20180801
Time 14.10 h
INSTRUM spect
PROBHD Z800701_0108 (
PULPROG jmod
TD 65536
SOLVENT CDCl3
NS 136
DS 4
SWH 29761.904 Hz
FIDRES 0.908261 Hz
AQ 1.1010048 sec
RG 198.17
DW 16.800 usec
DE 6.50 usec
TE 298.0 K
CNST2 145.0000000
CNST11 1.0000000
D1 2.00000000 sec
D20 0.00689655 sec
TD0 1
SFO1 125.7703643 MHz
NUC1 13C
P1 8.00 usec
P2 16.00 usec
PLW1 53.00000000 W
SFO2 500.1320005 MHz
NUC2 1H
CPDPRG[2 waltz16
PCPD2 80.00 usec
PLW2 28.00000000 W
PLW12 0.42017001 W

F2 - Processing parameters
SI 32768
SF 125.7577948 MHz
WDW EM
SSB 0
LB 1.00 Hz
GB 0
PC 1.40

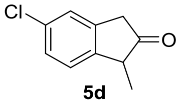

5d

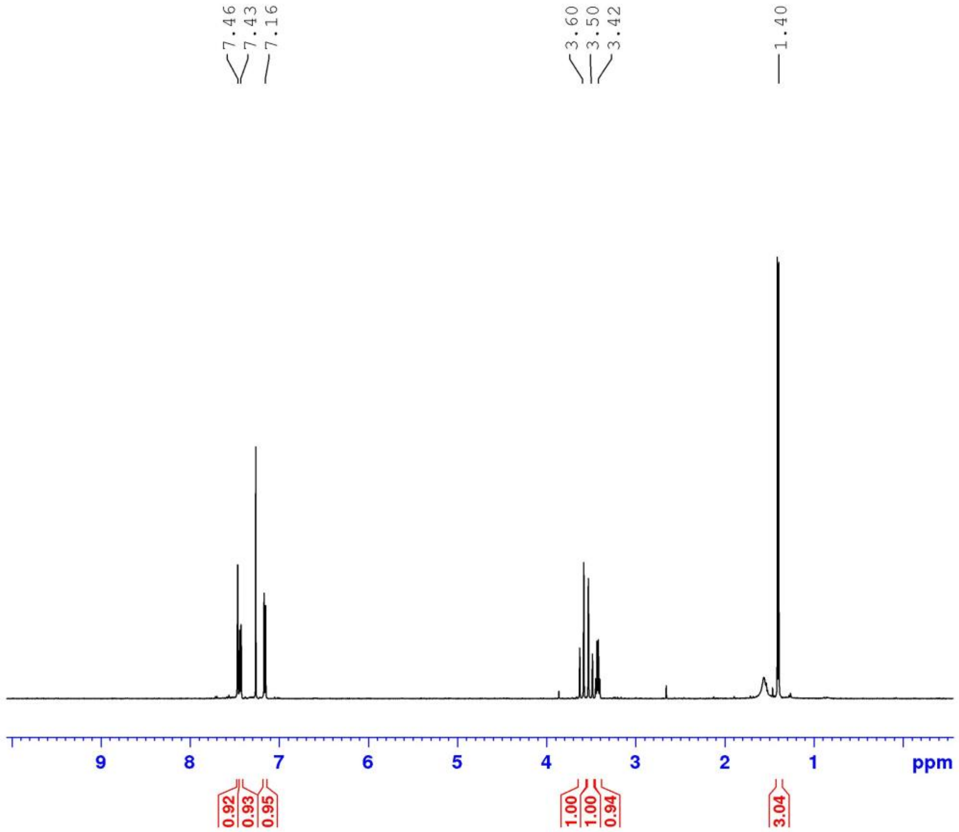


```
Current Data Parameters
NAME          Compound 5e
EXPNO                   1
PROCNO                  1

F2 - Acquisition Parameters
Date_            20180316
Time                16.55 h
INSTRUM             spect
PROBHD   Z800701_0108 (
PULPROG              zg30
TD                  65536
SOLVENT             CDCl3
NS                     16
DS                      2
SWH             10000.000 Hz
FIDRES           0.305176 Hz
AQ              3.2767999 sec
RG                 198.17
DW                 50.000 usec
DE                   6.50 usec
TE                  298.0 K
D1             1.00000000 sec
TD0                     1
SFO1          500.1330883 MHz
NUC1                   1H
P1                  10.00 usec
PLW1          26.00000000 W

F2 - Processing parameters
SI                  65536
SF            500.1300126 MHz
WDW                    EM
SSB      0
LB                   0.30 Hz
GB       0
PC                   1.00
```

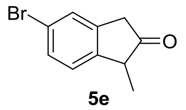

5e

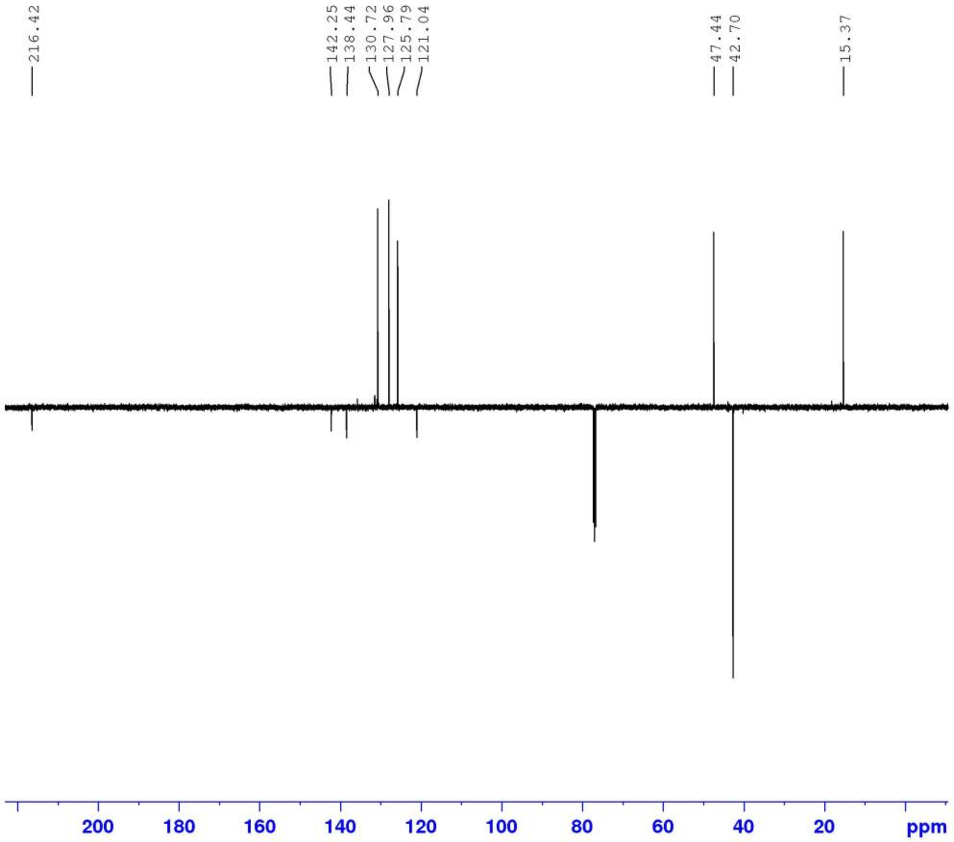


```
Current Data Parameters
NAME          Compound 5e
EXPNO                   2
PROCNO                  1

F2 - Acquisition Parameters
Date_            20180316
Time                17.42 h
INSTRUM             spect
PROBHD   Z800701_0108 (
PULPROG              jmod
TD                  65536
SOLVENT             CDCl3
NS                    507
DS                      4
SWH             31250.000 Hz
FIDRES           0.953674 Hz
AQ              1.0485760 sec
RG                 198.17
DW                 16.000 usec
DE                   6.50 usec
TE                  298.0 K
CNST2         145.0000000
CNST11          1.0000000
D1             2.00000000 sec
D20            0.00689655 sec
TD0                     1
SFO1          125.7703643 MHz
NUC1                  13C
P1                   8.00 usec
P2                  16.00 usec
PLW1          53.00000000 W
SFO2          500.1320005 MHz
NUC2                   1H
CPDPRG[2          waltz16
PCPD2               80.00 usec
PLW2          28.00000000 W
PLW12          0.42017001 W

F2 - Processing parameters
SI                  32768
SF            125.7577932 MHz
WDW                    EM
SSB      0
LB                   1.00 Hz
GB       0
PC                   1.40
```

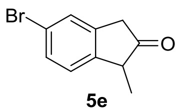

5e

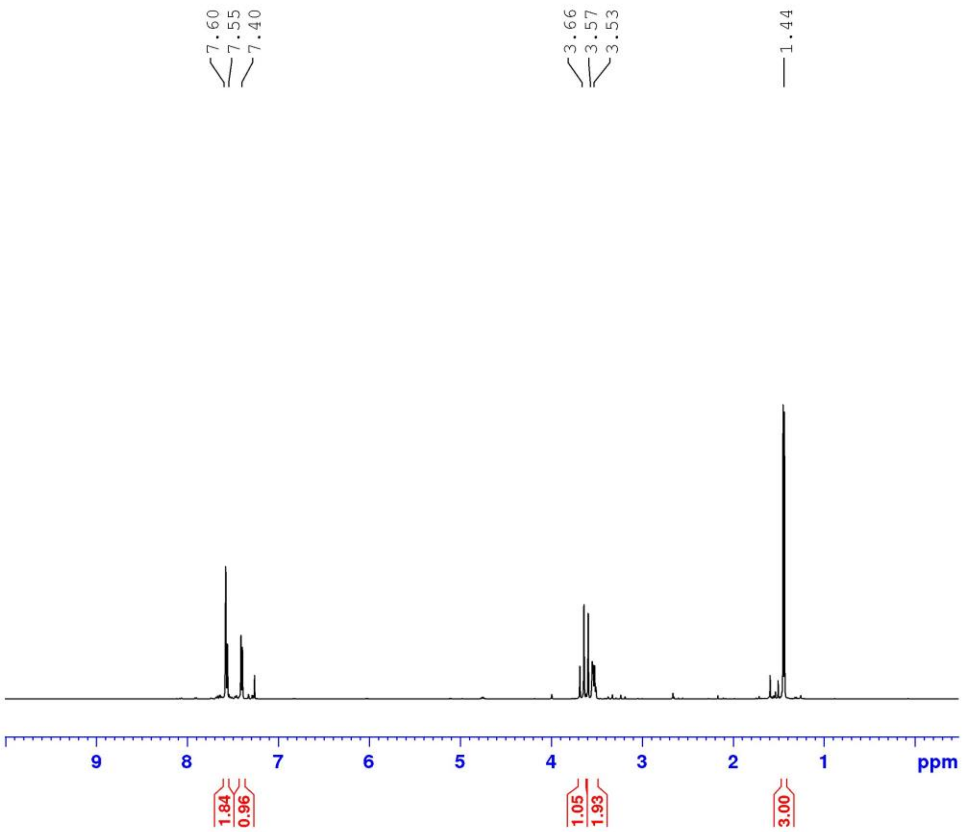


```
Current Data Parameters
NAME           compound 5f
EXPNO                    1
PROCNO                   1

F2 - Acquisition Parameters
Date_             20190802
Time                 14.09 h
INSTRUM              spect
PROBHD    Z800701_0108 (
PULPROG               zg30
TD                   65536
SOLVENT              CDCl3
NS                      16
DS                       2
SWH              10000.000 Hz
FIDRES            0.305176 Hz
AQ               3.2767999 sec
RG                  198.17
DW                  50.000 usec
DE                   13.89 usec
TE                   298.0 K
D1              1.00000000 sec
TD0                      1
SFO1           500.1330883 MHz
NUC1                    1H
P0                    3.33 usec
P1                   10.00 usec
PLW1           26.00000000 W

F2 - Processing parameters
SI                   65536
SF             500.1300119 MHz
WDW                     EM
SSB      0
LB                    0.30 Hz
GB       0
PC                    1.00
```

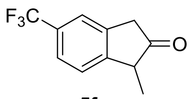

5f

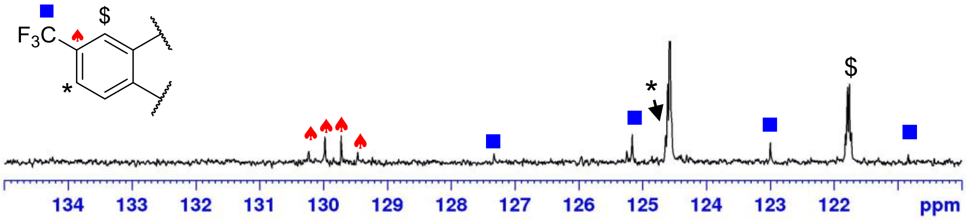

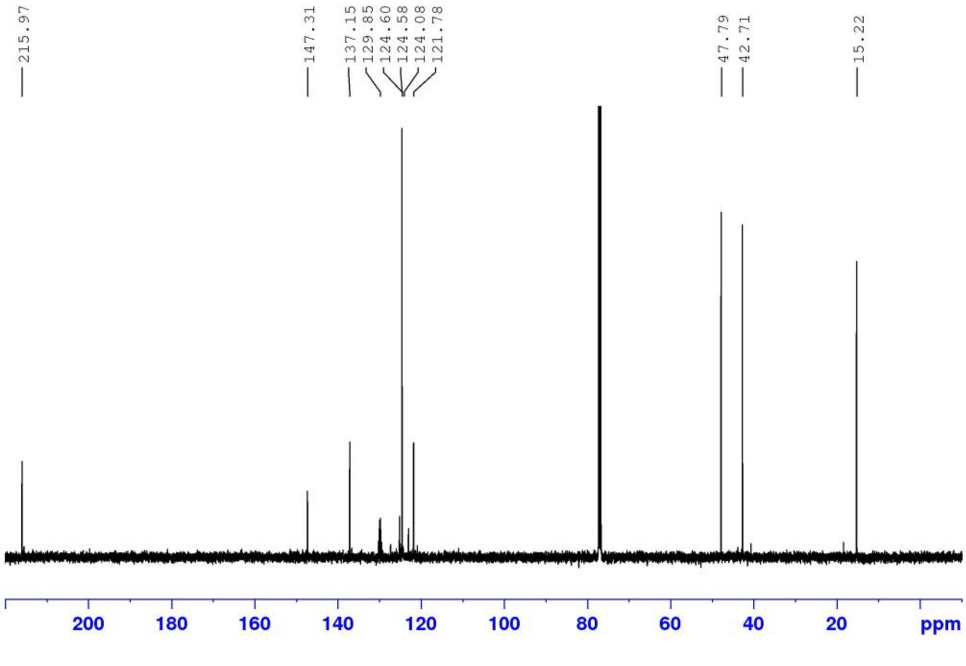


```
Current Data Parameters
NAME           compound 5f
EXPNO                    2
PROCNO                   1

F2 - Acquisition Parameters
Date_             20190802
Time                 15.19 h
INSTRUM              spect
PROBHD    Z800701_0108 (
PULPROG             zgpg30
TD                   65536
SOLVENT              CDCl3
NS                     255
DS                       4
SWH              31250.000 Hz
FIDRES            0.953674 Hz
AQ               1.0485760 sec
RG                  198.17
DW                  16.000 usec
DE                    6.50 usec
TE                   298.1 K
D1              2.00000000 sec
D11             0.03000000 sec
TD0                      1
SFO1           125.7703643 MHz
NUC1                   13C
P0                    2.67 usec
P1                    8.00 usec
PLW1           53.00000000 W
SFO2           500.1320005 MHz
NUC2                    1H
CPDPRG[2           waltz65
PCPD2                80.00 usec
PLW2           28.00000000 W
PLW12           0.42017001 W
PLW13           0.21134999 W

F2 - Processing parameters
SI                   32768
SF             125.7577913 MHz
WDW                     EM
SSB      0
LB                    1.00 Hz
GB       0
PC                    1.40
```

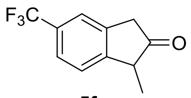

5f

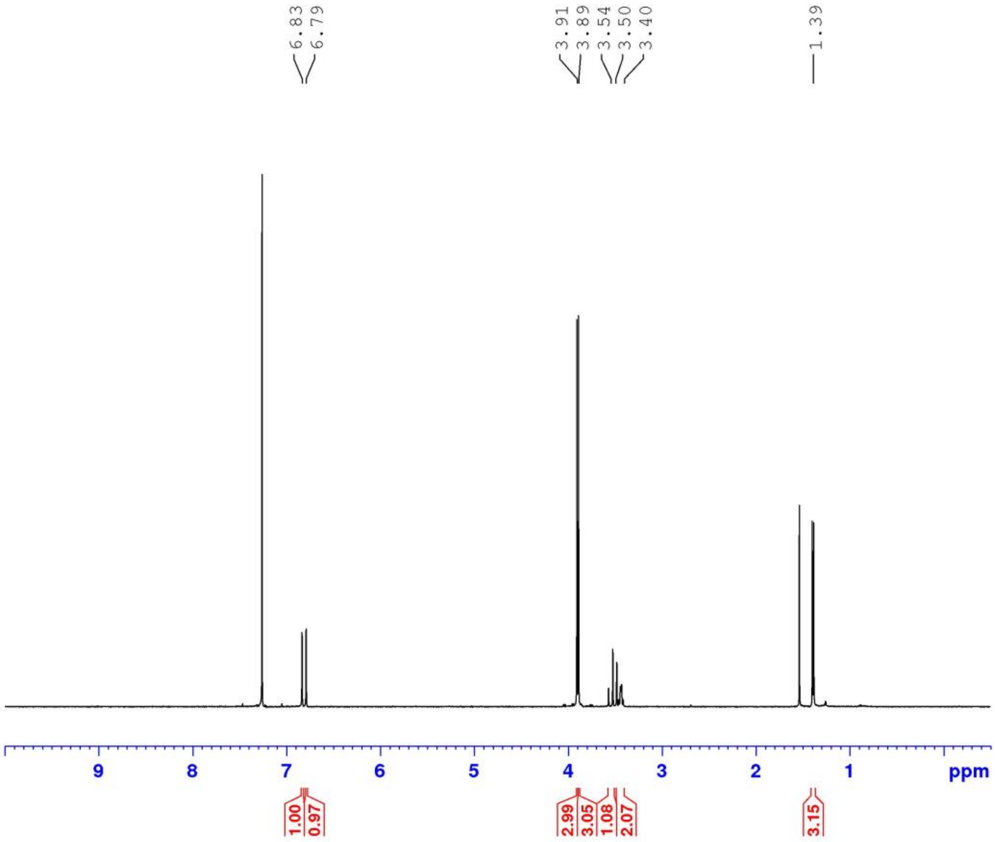
6.83
6.79
3.91
3.89
3.54
3.50
3.40
1.39
9
8
7
6
5
4
3
2
1
ppm
1.00
0.97
2.99
3.05
1.08
2.07
3.15
Current Data Parameters
NAME Compound 5h
EXPNO 2
PROCNO 1
F2 - Acquisition Parameters
Date_ 20180910
Time 17.00 h
INSTRUM spect
PROBHD Z800701_0108 (
PULPROG zg30
TD 65536
SOLVENT CDCl3
NS 16
DS 2
SWH 10000.000 Hz
FIDRES 0.305176 Hz
AQ 3.2767999 sec
RG 198.17
DW 50.000 usec
DE 6.50 usec
TE 298.0 K
D1 1.00000000 sec
TD0 1
SFO1 500.1330883 MHz
NUC1 1H
P1 10.00 usec
PLW1 26.00000000 W
F2 - Processing parameters
SI 65536
SF 500.1300125 MHz
WDW EM
SSB 0
LB 0.30 Hz
GB 0
PC 1.00

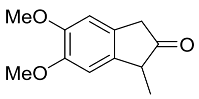
MeO
MeO
O

5h

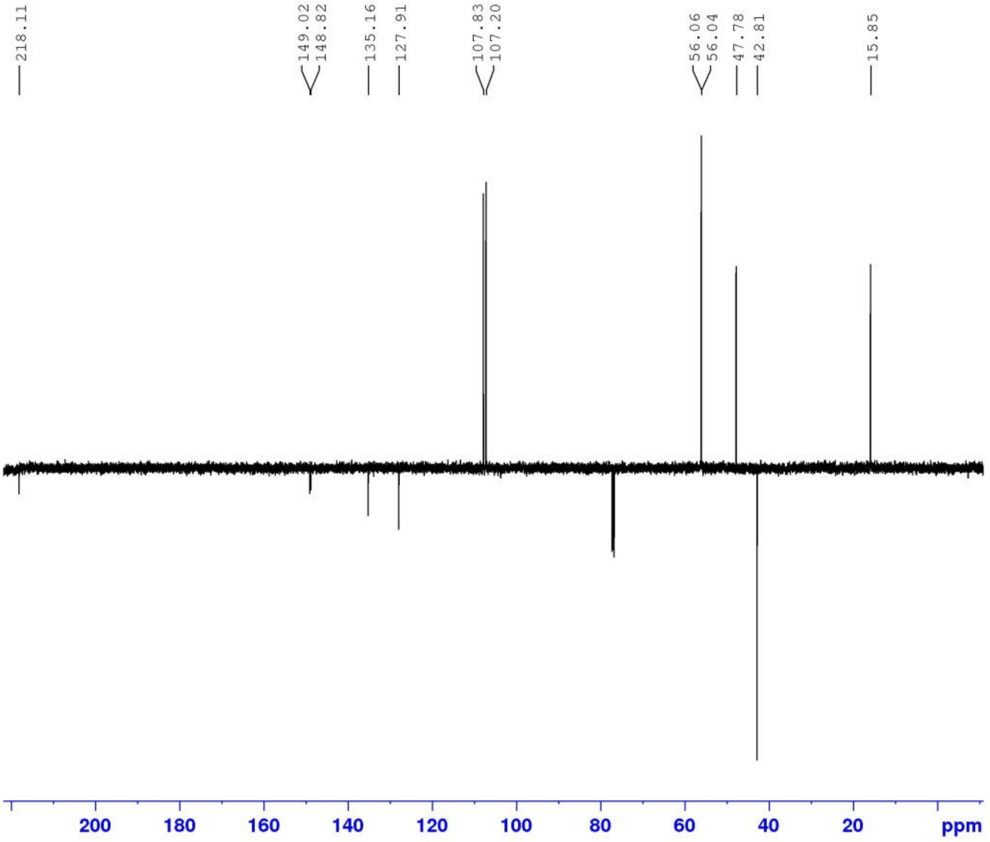
218.11
149.02
148.82
135.16
127.91
107.83
107.20
56.06
56.04
47.78
42.81
15.85
200
180
160
140
120
100
80
60
40
20
ppm
Current Data Parameters
NAME Compound 5h
EXPNO 4
PROCNO 1
F2 - Acquisition Parameters
Date_ 20180912
Time 16.55 h
INSTRUM spect
PROBHD Z800701_0108 (
PULPROG jmod
TD 65536
SOLVENT CDCl3
NS 75
DS 4
SWH 31250.000 Hz
FIDRES 0.953674 Hz
AQ 1.0485760 sec
RG 198.17
DW 16.000 usec
DE 6.50 usec
TE 298.0 K
CNST2 145.0000000
CNST11 1.0000000
D1 2.00000000 sec
D20 0.00689655 sec
TD0 1
SFO1 125.7703643 MHz
NUC1 13C
P1 8.00 usec
P2 16.00 usec
PLW1 53.00000000 W
SFO2 500.1320005 MHz
NUC2 1H
CPDPRG[2 waltz16
PCPD2 80.00 usec
PLW2 28.00000000 W
PLW12 0.42017001 W
F2 - Processing parameters
SI 32768
SF 125.7577957 MHz
WDW EM
SSB 0
LB 1.00 Hz
GB 0
PC 1.40

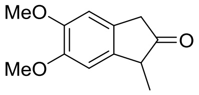
MeO
MeO
O

5h

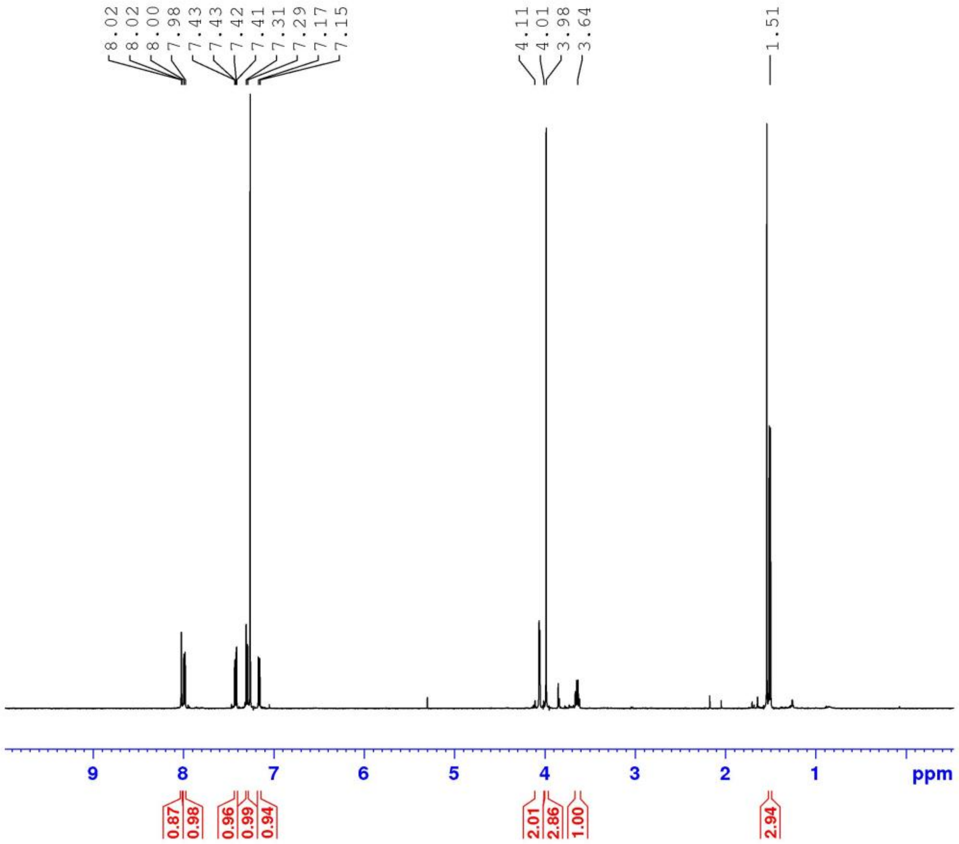


```
Current Data Parameters
NAME        Compound  5i
EXPNO                  1
PROCNO                 1

F2 - Acquisition Parameters
Date_          20170426
Time              17.12
INSTRUM           spect
PROBHD   5 mm PABBO BB-
PULPROG            zg30
TD                65536
SOLVENT           CDCl3
NS                   16
DS                    2
SWH          10000.000 Hz
FIDRES        0.152588 Hz
AQ           3.2767999 sec
RG              198.17
DW              50.000 usec
DE                6.50 usec
TE               298.0 K
D1          1.00000000 sec
TD0                   1

======== CHANNEL f1 ========
SFO1       500.1330885 MHz
NUC1                1H
P1               10.00 usec
PLW1       26.00000000 W

F2 - Processing parameters
SI                65536
SF         500.1300123 MHz
WDW                  EM
SSB      0
LB                0.30 Hz
GB       0
PC                1.00
```

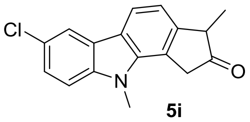

5i

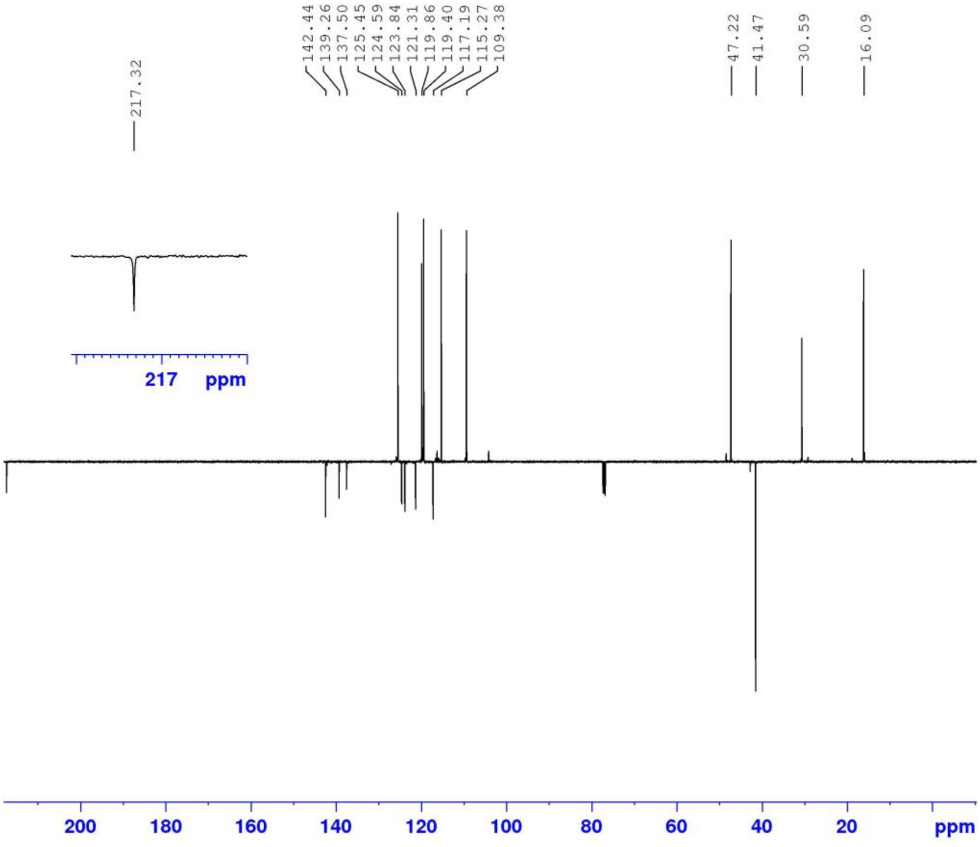


```
Current Data Parameters
NAME        Compound  5i
EXPNO                  2
PROCNO                 1

F2 - Acquisition Parameters
Date_          20170427
Time              11.30
INSTRUM           spect
PROBHD   5 mm PABBO BB-
PULPROG            jmod
TD                65536
SOLVENT           CDCl3
NS                  512
DS                    4
SWH          29761.904 Hz
FIDRES        0.454131 Hz
AQ           1.1010048 sec
RG              198.17
DW              16.800 usec
DE                6.50 usec
TE               298.0 K
CNST2      145.0000000
CNST11       1.0000000
D1          2.00000000 sec
D20         0.00689655 sec
TD0                   1

======== CHANNEL f1 ========
SFO1       125.7703643 MHz
NUC1                13C
P1                8.00 usec
P2               16.00 usec
PLW1       53.00000000 W

======== CHANNEL f2 ========
SFO2       500.1320005 MHz
NUC2                 1H
CPDPRG[2         waltz16
PCPD2            80.00 usec
PLW2       28.00000000 W
PLW12       0.42017001 W

F2 - Processing parameters
SI                32768
SF         125.7578021 MHz
WDW                  EM
SSB      0
LB                1.00 Hz
GB       0
PC                1.40
```

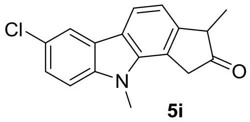

5i

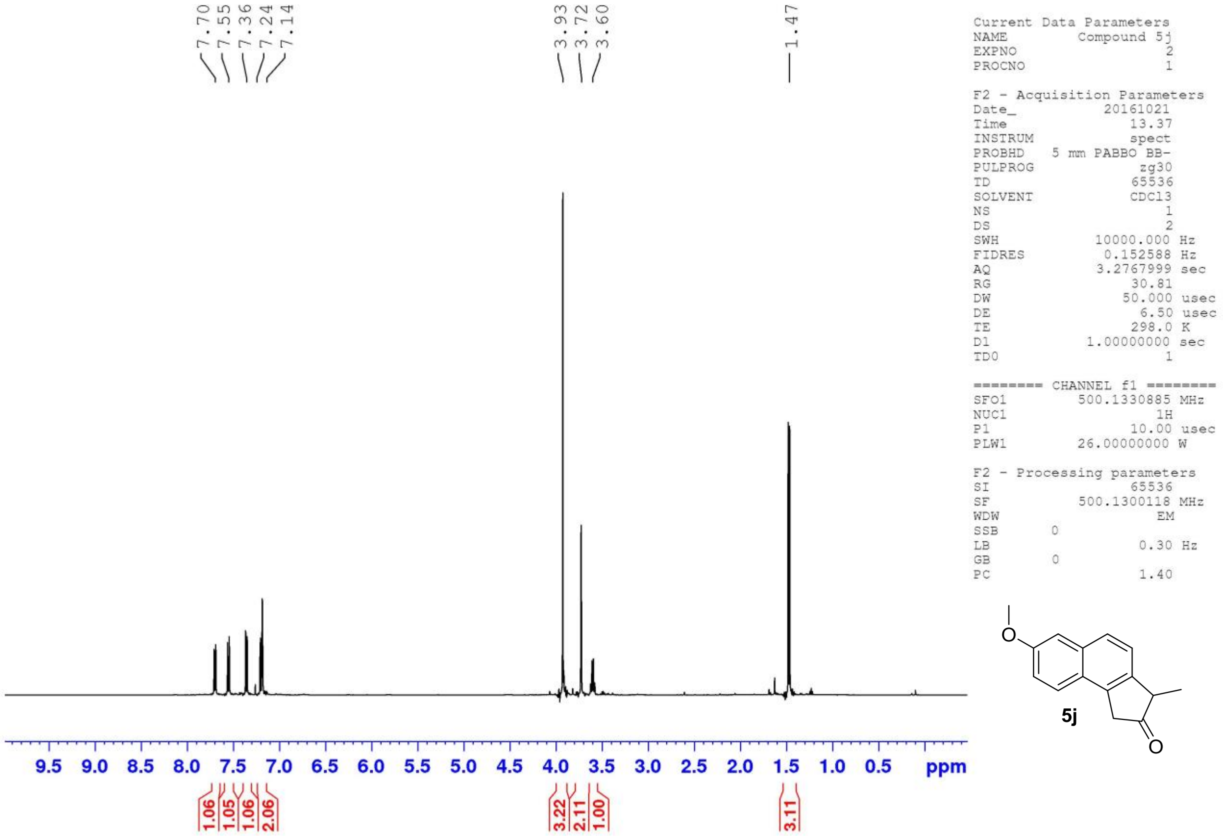

7.70
7.55
7.36
7.24
7.14
3.93
3.72
3.60
1.47
Current Data Parameters
NAME Compound 5j
EXPNO 2
PROCNO 1
F2 - Acquisition Parameters
Date_ 20161021
Time 13.37
INSTRUM spect
PROBHD 5 mm PABBO BB-
PULPROG zg30
TD 65536
SOLVENT CDCl3
NS 1
DS 2
SWH 10000.000 Hz
FIDRES 0.152588 Hz
AQ 3.2767999 sec
RG 30.81
DW 50.000 usec
DE 6.50 usec
TE 298.0 K
D1 1.00000000 sec
TD0 1
======== CHANNEL f1 ========
SFO1 500.1330885 MHz
NUC1 1H
P1 10.00 usec
PLW1 26.00000000 W
F2 - Processing parameters
SI 65536
SF 500.1300118 MHz
WDW EM
SSB 0
LB 0.30 Hz
GB 0
PC 1.40
5j
9.5 9.0 8.5 8.0 7.5 7.0 6.5 6.0 5.5 5.0 4.5 4.0 3.5 3.0 2.5 2.0 1.5 1.0 0.5 ppm
1.06
1.05
1.06
2.06
3.22
2.11
1.00
3.11

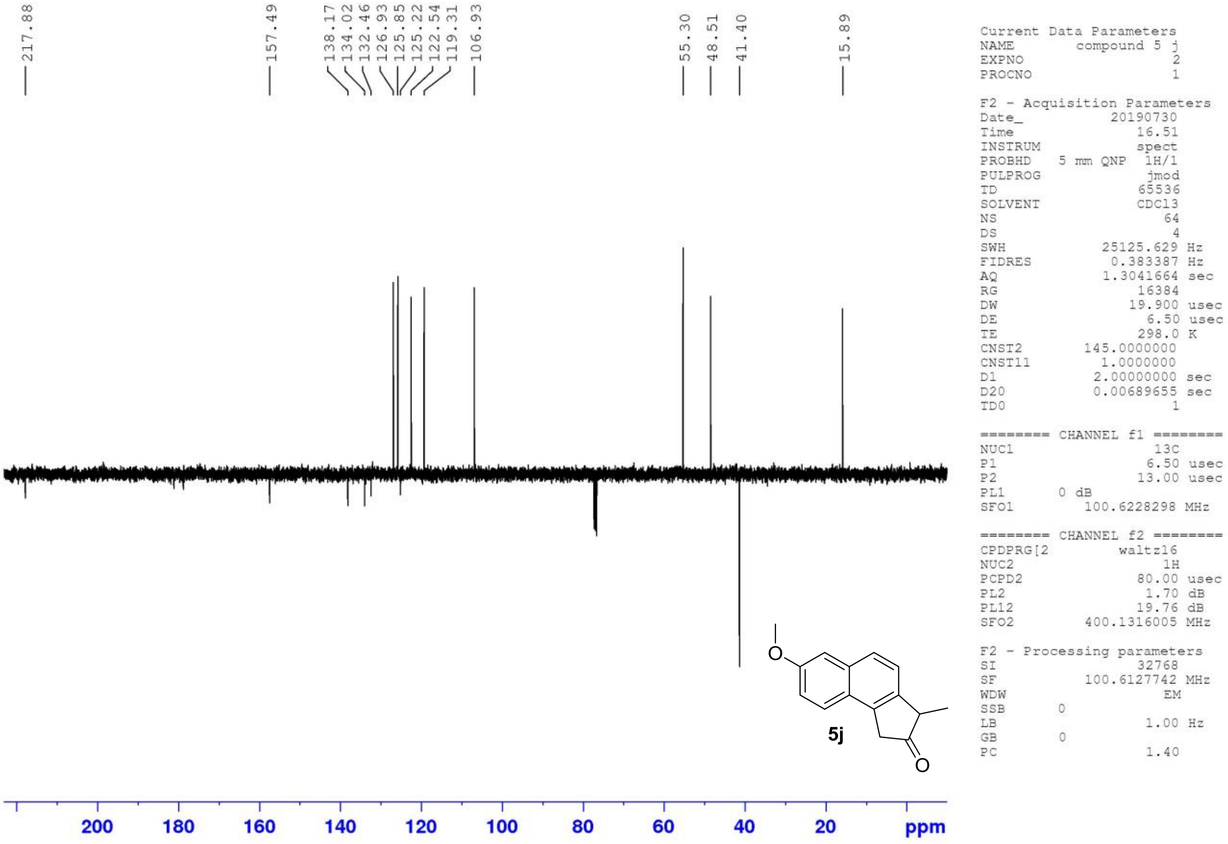

217.88
157.49
138.17
134.02
132.46
126.93
125.85
125.22
122.54
119.31
106.93
55.30
48.51
41.40
15.89
Current Data Parameters
NAME compound 5 j
EXPNO 2
PROCNO 1
F2 - Acquisition Parameters
Date_ 20190730
Time 16.51
INSTRUM spect
PROBHD 5 mm QNP 1H/1
PULPROG jmod
TD 65536
SOLVENT CDCl3
NS 64
DS 4
SWH 25125.629 Hz
FIDRES 0.383387 Hz
AQ 1.3041664 sec
RG 16384
DW 19.900 usec
DE 6.50 usec
TE 298.0 K
CNST2 145.0000000
CNST11 1.0000000
D1 2.00000000 sec
D20 0.00689655 sec
TD0 1
======== CHANNEL f1 ========
NUC1 13C
P1 6.50 usec
P2 13.00 usec
PL1 0 dB
SFO1 100.6228298 MHz
======== CHANNEL f2 ========
CPDPRG[2 waltz16
NUC2 1H
PCPD2 80.00 usec
PL2 1.70 dB
PL12 19.76 dB
SFO2 400.1316005 MHz
F2 - Processing parameters
SI 32768
SF 100.6127742 MHz
WDW EM
SSB 0
LB 1.00 Hz
GB 0
PC 1.40
5j
200 180 160 140 120 100 80 60 40 20 ppm

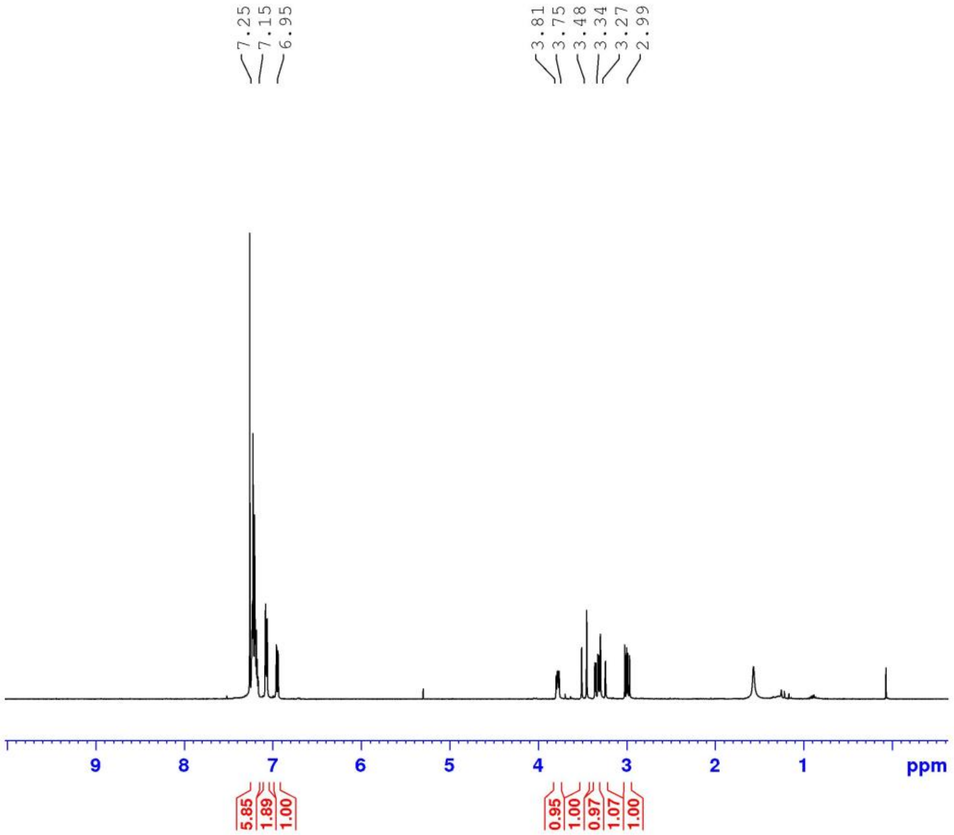

Current Data Parameters
NAME Compound 5k
EXPNO 1
PROCNO 1

F2 - Acquisition Parameters
Date_ 20170912
Time 13.22
INSTRUM spect
PROBHD 5 mm QNP 1H/13
PULPROG zg30
TD 65536
SOLVENT CDCl3
NS 16
DS 2
SWH 8278.146 Hz
FIDRES 0.126314 Hz
AQ 3.9583745 sec
RG 456.1
DW 60.400 usec
DE 6.50 usec
TE 300.0 K
D1 1.00000000 sec
TD0 1

======== CHANNEL f1 ========
NUC1 1H
P1 10.00 usec
PL1 1.00 dB
SFO1 400.1324710 MHz

F2 - Processing parameters
SI 32768
SF 400.1300098 MHz
WDW EM
SSB 0
LB 0.30 Hz
GB 0
PC 1.00

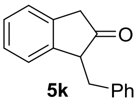

5k

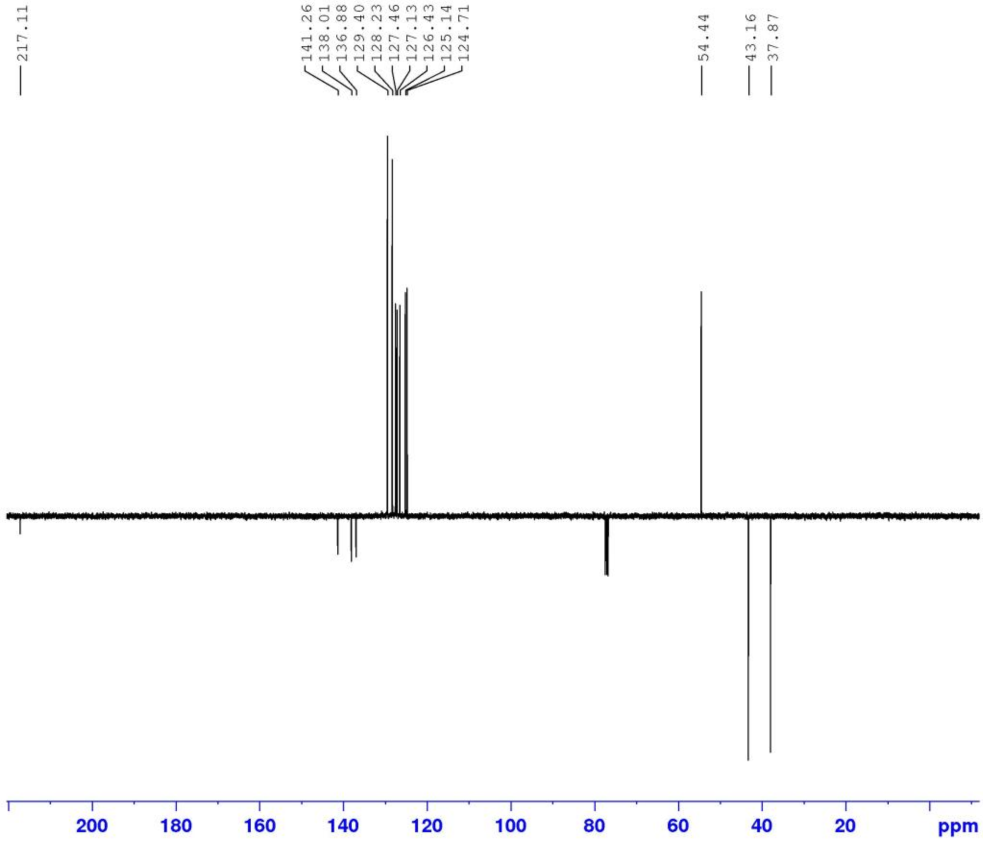

Current Data Parameters
NAME Compound 5k
EXPNO 2
PROCNO 1

F2 - Acquisition Parameters
Date_ 20170912
Time 14.42
INSTRUM spect
PROBHD 5 mm QNP 1H/13
PULPROG jmod
TD 65536
SOLVENT CDCl3
NS 256
DS 4
SWH 25125.629 Hz
FIDRES 0.383387 Hz
AQ 1.3041664 sec
RG 16384
DW 19.900 usec
DE 6.50 usec
TE 300.0 K
CNST2 145.0000000
CNST11 1.0000000
D1 2.00000000 sec
D20 0.00689655 sec
TD0 1

======== CHANNEL f1 ========
NUC1 13C
P1 8.00 usec
P2 16.00 usec
PL1 0 dB
SFO1 100.6228298 MHz

======== CHANNEL f2 ========
CPDPRG[2 waltz16
NUC2 1H
PCPD2 80.00 usec
PL2 1.00 dB
PL12 19.06 dB
SFO2 400.1316005 MHz

F2 - Processing parameters
SI 32768
SF 100.6127770 MHz
WDW EM
SSB 0
LB 1.00 Hz
GB 0
PC 1.40

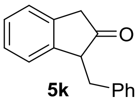

5k

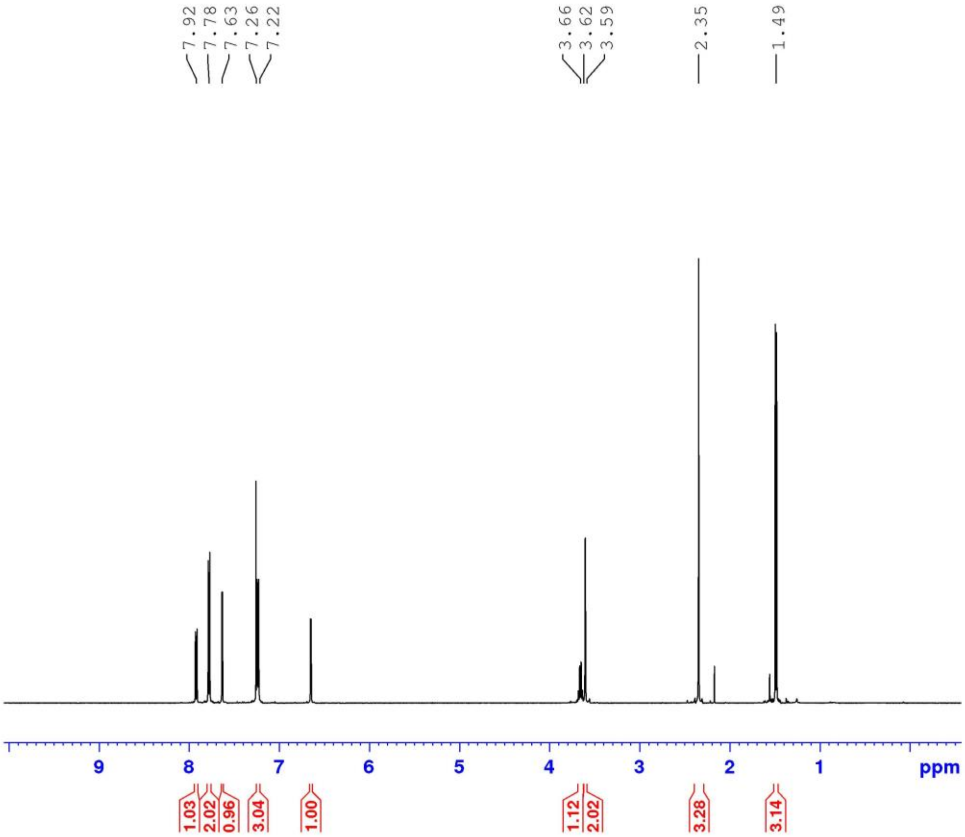


```
Current Data Parameters
NAME          Compound 5l
EXPNO                   2
PROCNO                  1

F2 - Acquisition Parameters
Date_            20190313
Time                17.40 h
INSTRUM             spect
PROBHD   Z800701_0108 (
PULPROG              zg30
TD                  65536
SOLVENT             CDCl3
NS                     16
DS                      2
SWH             10000.000 Hz
FIDRES           0.305176 Hz
AQ              3.2767999 sec
RG                 198.17
DW                 50.000 usec
DE                   6.50 usec
TE                  298.0 K
D1             1.00000000 sec
TD0                     1
SFO1          500.1330883 MHz
NUC1                   1H
P1                  10.00 usec
PLW1          26.00000000 W

F2 - Processing parameters
SI                  65536
SF            500.1300122 MHz
WDW                    EM
SSB      0
LB                   0.30 Hz
GB       0
PC                   1.00
```

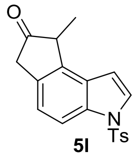
5l

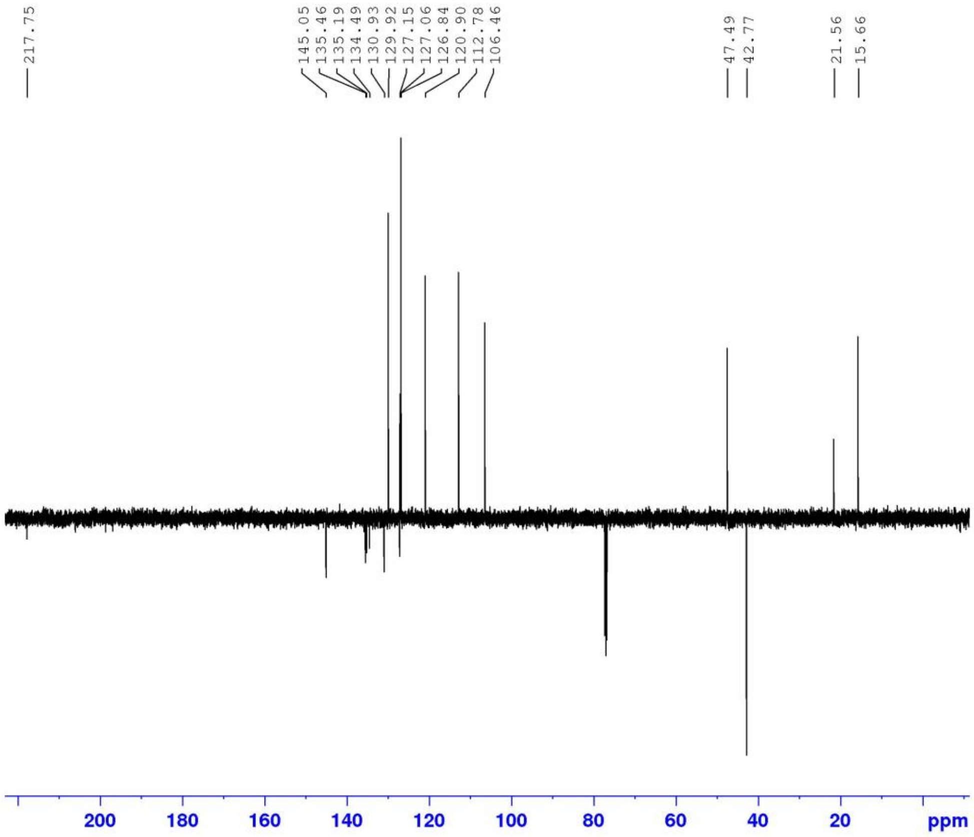


```
Current Data Parameters
NAME          Compound 5l
EXPNO                   3
PROCNO                  1

F2 - Acquisition Parameters
Date_            20190313
Time                13.32 h
INSTRUM             spect
PROBHD   Z800701_0108 (
PULPROG              jmod
TD                  65536
SOLVENT             CDCl3
NS                     78
DS                      4
SWH             31250.000 Hz
FIDRES           0.953674 Hz
AQ              1.0485760 sec
RG                 198.17
DW                 16.000 usec
DE                   6.50 usec
TE                  298.0 K
CNST2         145.0000000
CNST11          1.0000000
D1             2.00000000 sec
D20            0.00689655 sec
TD0                     1
SFO1          125.7703643 MHz
NUC1                  13C
P1                   8.00 usec
P2                  16.00 usec
PLW1          53.00000000 W
SFO2          500.1320005 MHz
NUC2                   1H
CPDPRG[2          waltz16
PCPD2               80.00 usec
PLW2          28.00000000 W
PLW12          0.42017001 W

F2 - Processing parameters
SI                  32768
SF            125.7577932 MHz
WDW                    EM
SSB      0
LB                   1.00 Hz
GB       0
PC                   1.40
```

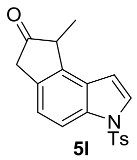
5l

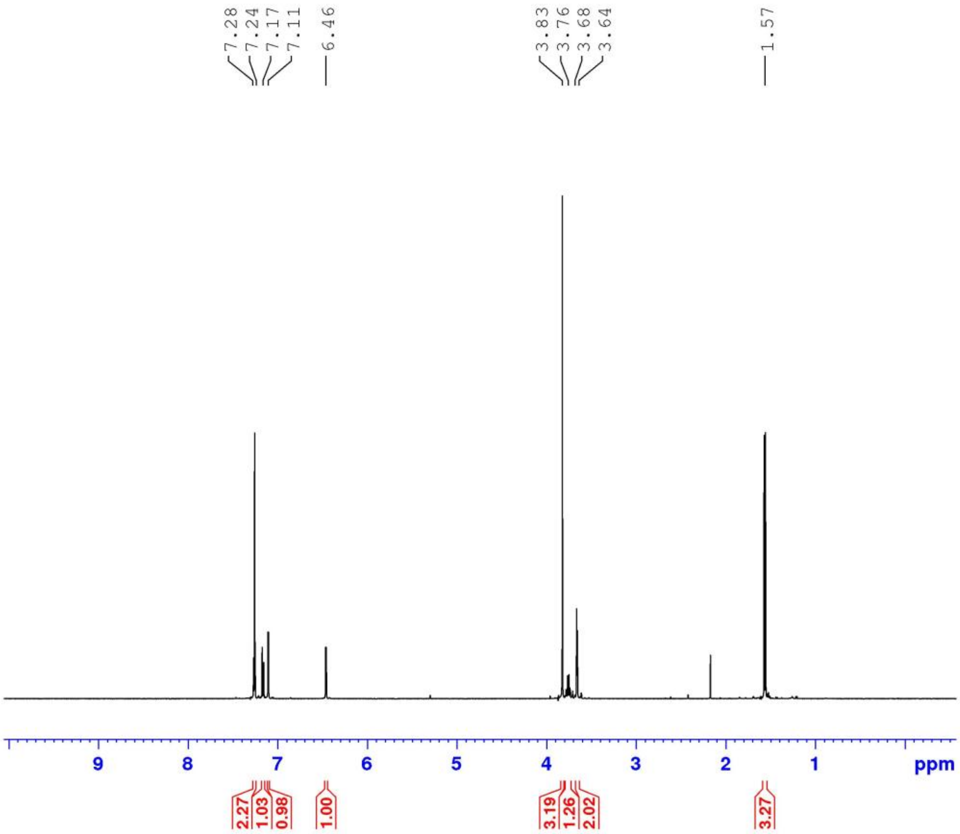


```
Current Data Parameters
NAME          Compound 5m
EXPNO                   1
PROCNO                  1

F2 - Acquisition Parameters
Date_            20190405
Time                13.04 h
INSTRUM             spect
PROBHD    Z800701_0108 (
PULPROG              zg30
TD                  65536
SOLVENT             CDCl3
NS                     16
DS                      2
SWH             10000.000 Hz
FIDRES           0.305176 Hz
AQ              3.2767999 sec
RG                 198.17
DW                 50.000 usec
DE                   6.50 usec
TE                  298.0 K
D1             1.00000000 sec
TD0                     1
SFO1          500.1330883 MHz
NUC1                   1H
P1                  10.00 usec
PLW1          26.00000000 W

F2 - Processing parameters
SI                  65536
SF            500.1300121 MHz
WDW                    EM
SSB      0
LB                   0.30 Hz
GB       0
PC                   1.00
```

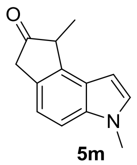

5m

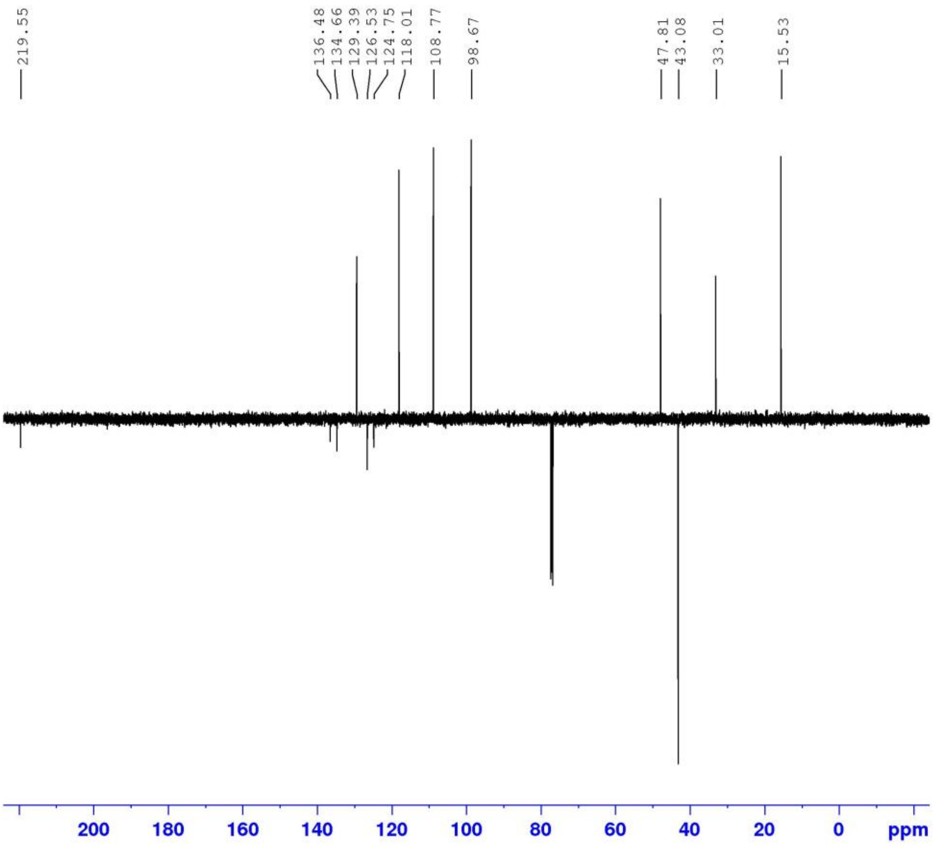


```
Current Data Parameters
NAME          Compound 5m
EXPNO                   2
PROCNO                  1

F2 - Acquisition Parameters
Date_            20190405
Time                13.26 h
INSTRUM             spect
PROBHD    Z800701_0108 (
PULPROG              jmod
TD                  65536
SOLVENT             CDCl3
NS                    256
DS                      4
SWH             31250.000 Hz
FIDRES           0.953674 Hz
AQ              1.0485760 sec
RG                 198.17
DW                 16.000 usec
DE                   6.50 usec
TE                  298.0 K
CNST2         145.0000000
CNST11          1.0000000
D1             2.00000000 sec
D20            0.00689655 sec
TD0                     1
SFO1          125.7703643 MHz
NUC1                  13C
P1                   8.00 usec
P2                  16.00 usec
PLW1          53.00000000 W
SFO2          500.1320005 MHz
NUC2                   1H
CPDPRG[2          waltz16
PCPD2               80.00 usec
PLW2          28.00000000 W
PLW12          0.42017001 W

F2 - Processing parameters
SI                  32768
SF            125.7577939 MHz
WDW                    EM
SSB      0
LB                   1.00 Hz
GB       0
PC                   1.40
```

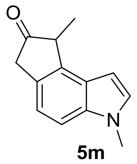

5m

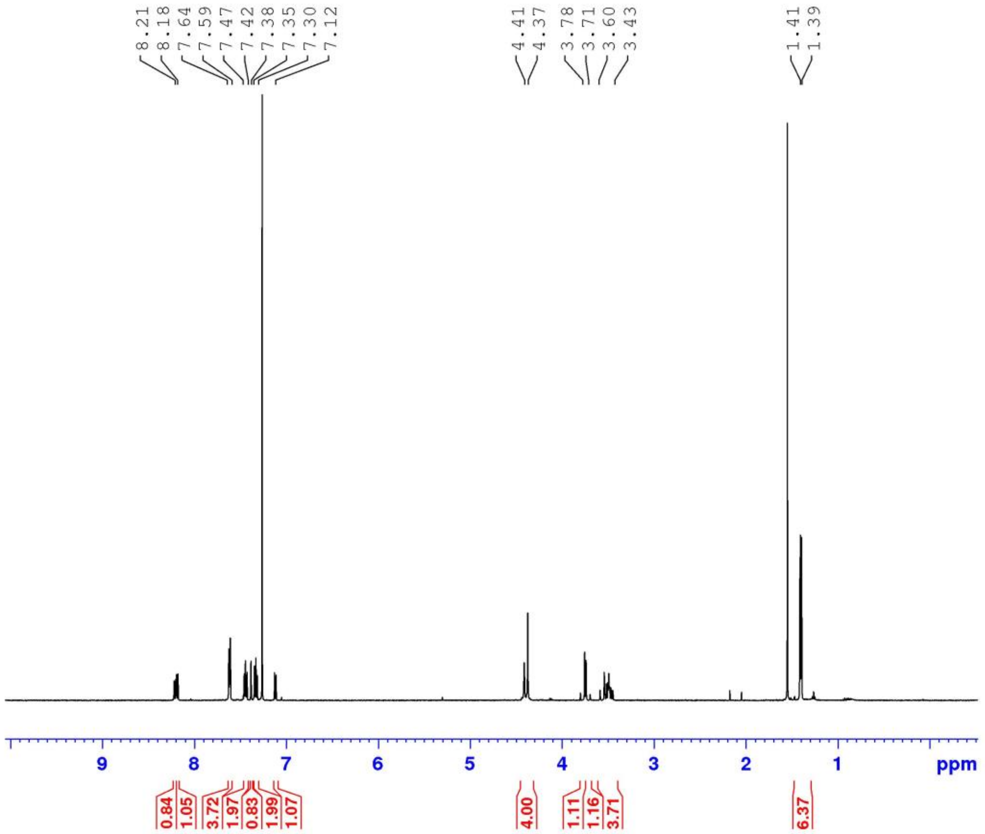

Current Data Parameters
NAME Compound 5o
EXPNO 1
PROCNO 1

F2 - Acquisition Parameters
Date_ 20170426
Time 8.47
INSTRUM spect
PROBHD 5 mm PABBO BB-
PULPROG zg30
TD 65536
SOLVENT CDCl3
NS 16
DS 2
SWH 10000.000 Hz
FIDRES 0.152588 Hz
AQ 3.2767999 sec
RG 198.17
DW 50.000 usec
DE 6.50 usec
TE 298.0 K
D1 1.00000000 sec
TD0 1

======== CHANNEL f1 ========
SFO1 500.1330885 MHz
NUC1 1H
P1 10.00 usec
PLW1 26.00000000 W

F2 - Processing parameters
SI 65536
SF 500.1300123 MHz
WDW EM
SSB 0
LB 0.30 Hz
GB 0
PC 1.00

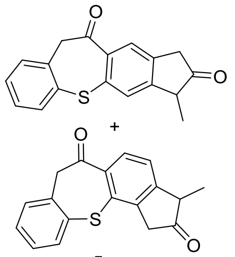

5o

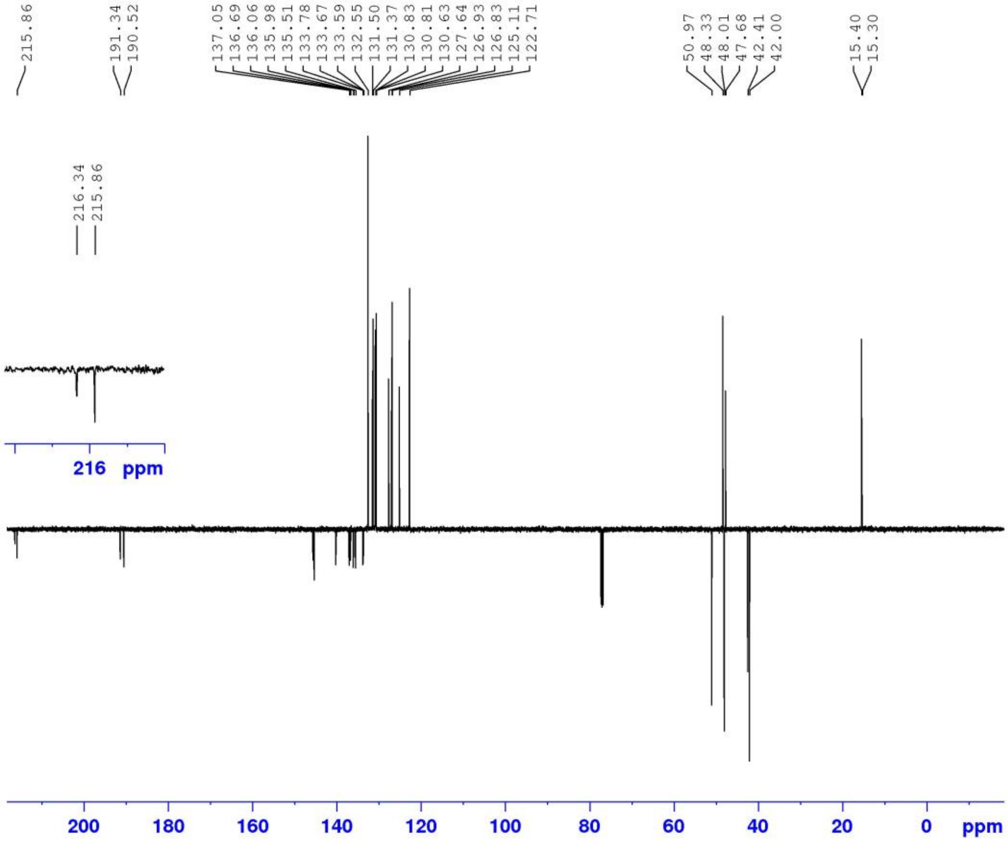

Current Data Parameters
NAME Compound 5o
EXPNO 2
PROCNO 1

F2 - Acquisition Parameters
Date_ 20170426
Time 9.25
INSTRUM spect
PROBHD 5 mm PABBO BB-
PULPROG jmod
TD 65536
SOLVENT CDCl3
NS 512
DS 4
SWH 29761.904 Hz
FIDRES 0.454131 Hz
AQ 1.1010048 sec
RG 198.17
DW 16.800 usec
DE 6.50 usec
TE 298.0 K
CNST2 145.0000000
CNST11 1.0000000
D1 2.00000000 sec
D20 0.00689655 sec
TD0 1

======== CHANNEL f1 ========
SFO1 125.7703643 MHz
NUC1 13C
P1 8.00 usec
P2 16.00 usec
PLW1 53.00000000 W

======== CHANNEL f2 ========
SFO2 500.1320005 MHz
NUC2 1H
CPDPRG[2 waltz16
PCPD2 80.00 usec
PLW2 28.00000000 W
PLW12 0.42017001 W

F2 - Processing parameters
SI 32768
SF 125.7577958 MHz
WDW EM
SSB 0
LB 1.00 Hz
GB 0
PC 1.40

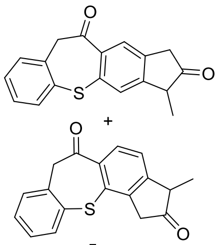

5o

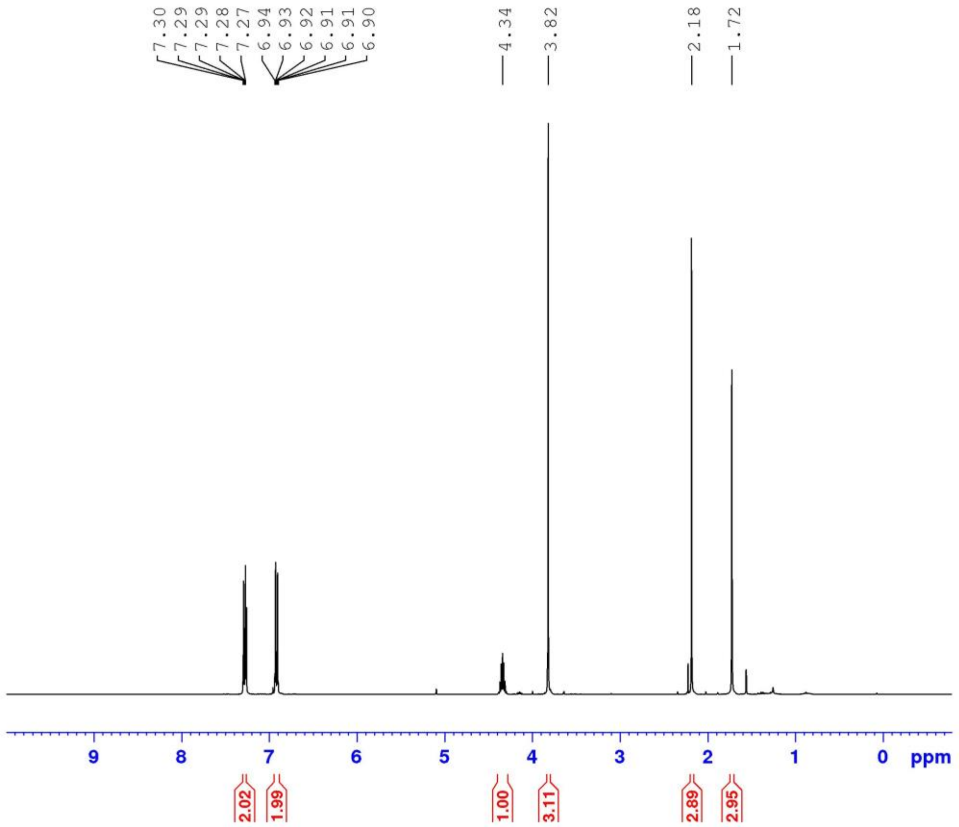


```
Current Data Parameters
NAME            Compound 6_
EXPNO                     1
PROCNO                    1

F2 - Acquisition Parameters
Date_              20180914
Time                  12.34
INSTRUM               spect
PROBHD   5 mm QNP 1H/13
PULPROG                zg30
TD                    65536
SOLVENT               CDCl3
NS                       16
DS                        2
SWH               8278.146 Hz
FIDRES            0.126314 Hz
AQ               3.9583745 sec
RG                    228.1
DW                   60.400 usec
DE                     6.50 usec
TE                    673.2 K
D1               1.00000000 sec
TD0                       1

======== CHANNEL f1 ========
NUC1                     1H
P1                    10.00 usec
PL1                    1.00 dB
SFO1            400.1324710 MHz

F2 - Processing parameters
SI                    32768
SF              400.1300097 MHz
WDW                      EM
SSB      0
LB                     0.30 Hz
GB       0
PC                     1.00
```

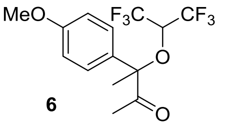

6

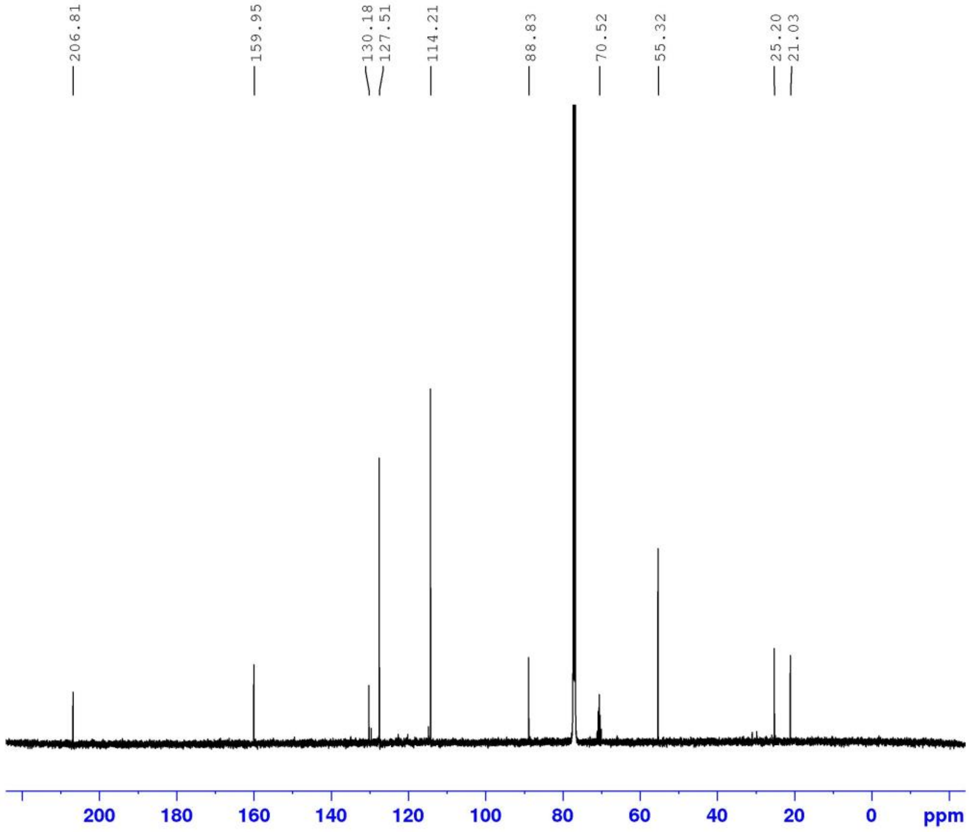


```
Current Data Parameters
NAME            compound 6
EXPNO                    1
PROCNO                   1

F2 - Acquisition Parameters
Date_             20190730
Time                  8.17 h
INSTRUM              spect
PROBHD   Z800701_0108 (
PULPROG            zgpg30
TD                   65536
SOLVENT              CDCl3
NS                   16384
DS                       4
SWH              31250.000 Hz
FIDRES            0.953674 Hz
AQ               1.0485760 sec
RG                  198.17
DW                  16.000 usec
DE                    6.50 usec
TE                   298.0 K
D1              2.00000000 sec
D11             0.03000000 sec
TD0                      1
SFO1           125.7703643 MHz
NUC1                   13C
P0                    2.67 usec
P1                    8.00 usec
PLW1           53.00000000 W
SFO2           500.1320005 MHz
NUC2                    1H
CPDPRG[2           waltz65
PCPD2                80.00 usec
PLW2           28.00000000 W
PLW12           0.42017001 W
PLW13           0.21134999 W

F2 - Processing parameters
SI                   32768
SF             125.7577885 MHz
WDW                     EM
SSB      0
LB                    1.00 Hz
GB       0
PC                    1.40
```

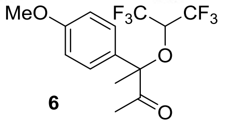

6

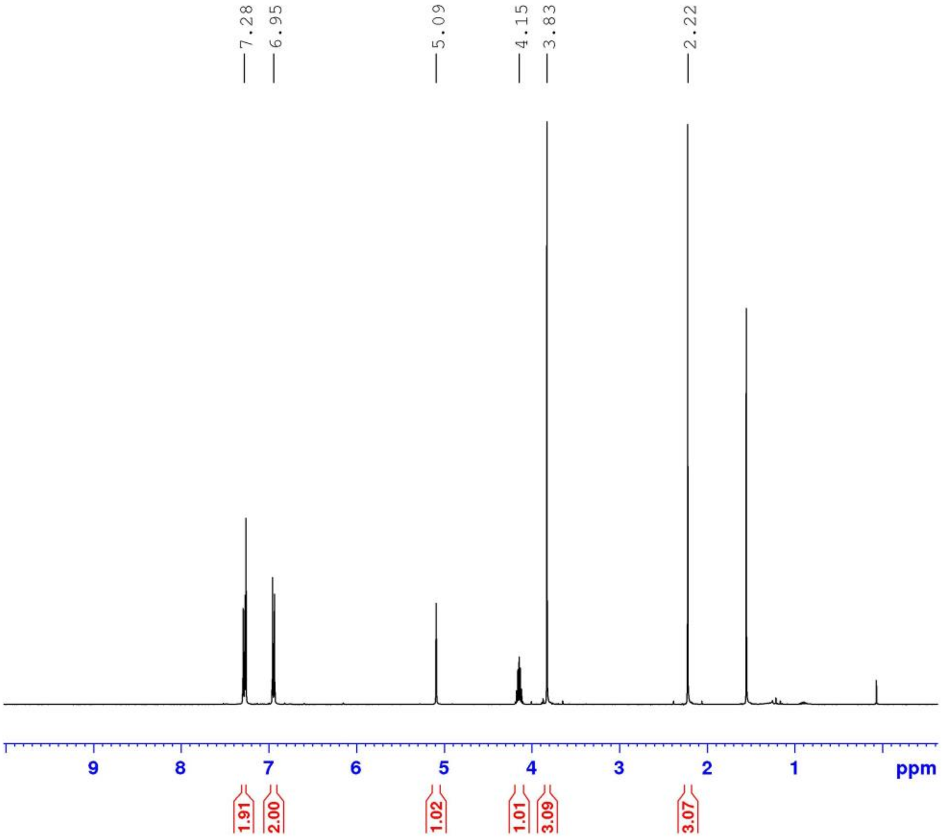


```
Current Data Parameters
NAME          Compound 7
EXPNO                  1
PROCNO                 1

F2 - Acquisition Parameters
Date_           20180919
Time               10.16
INSTRUM            spect
PROBHD   5 mm QNP 1H/13
PULPROG             zg30
TD                 65536
SOLVENT            CDCl3
NS                    16
DS                     2
SWH             8278.146 Hz
FIDRES          0.126314 Hz
AQ             3.9583745 sec
RG                   362
DW                60.400 usec
DE                  6.50 usec
TE                 298.0 K
D1            1.00000000 sec
TD0                    1

======== CHANNEL f1 ========
NUC1                  1H
P1                 10.00 usec
PL1                 1.00 dB
SFO1         400.1324710 MHz

F2 - Processing parameters
SI                 32768
SF           400.1300095 MHz
WDW                   EM
SSB      0
LB                  0.30 Hz
GB       0
PC                  1.00
```

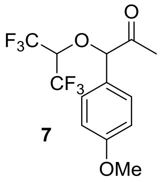

7

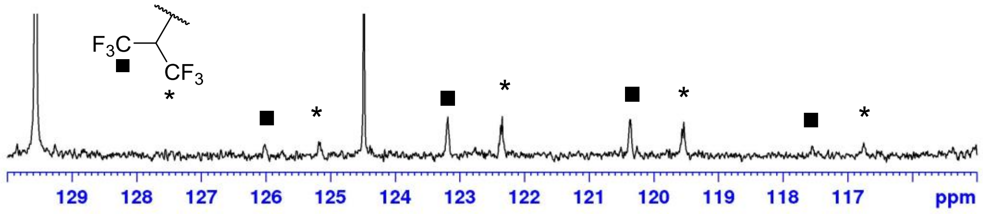

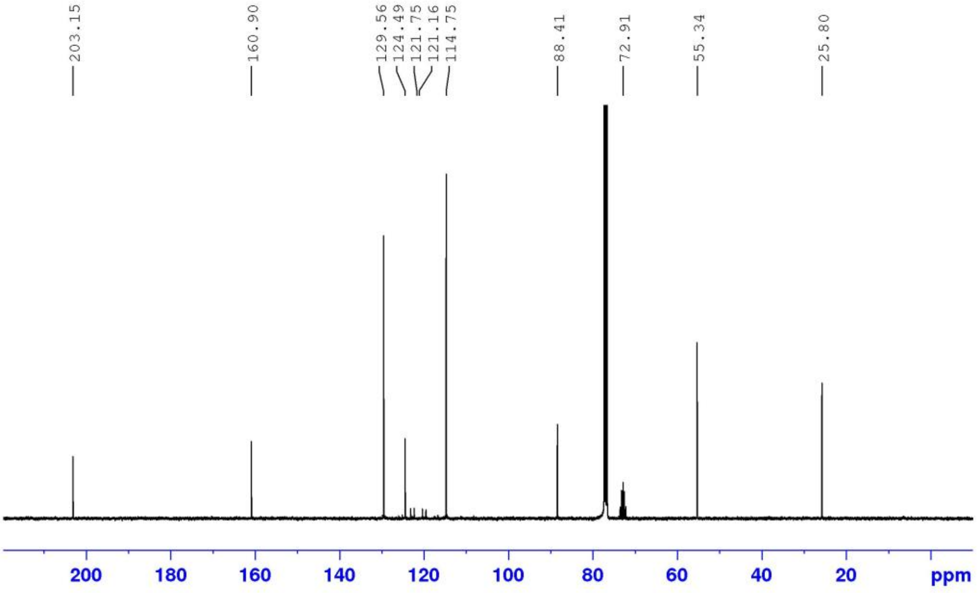


```
Current Data Parameters
NAME          Compound 7
EXPNO                  4
PROCNO                 1

F2 - Acquisition Parameters
Date_           20190725
Time                8.09
INSTRUM            spect
PROBHD   5 mm QNP 1H/1
PULPROG          zgpg30
TD                 65536
SOLVENT            CDCl3
NS                 15000
DS                     4
SWH            25125.629 Hz
FIDRES          0.383387 Hz
AQ             1.3041664 sec
RG                 574.7
DW                19.900 usec
DE                  6.50 usec
TE                 298.0 K
D1            2.00000000 sec
D11           0.03000000 sec
TD0                    1

======== CHANNEL f1 ========
NUC1                 13C
P1                  6.50 usec
PL1       0 dB
SFO1         100.6228298 MHz

======== CHANNEL f2 ========
CPDPRG[2         waltz16
NUC2                  1H
PCPD2              80.00 usec
PL2                 1.70 dB
PL12               19.76 dB
PL13               21.00 dB
SFO2         400.1316005 MHz

F2 - Processing parameters
SI                 32768
SF           100.6127702 MHz
WDW                   EM
SSB      0
LB                  1.00 Hz
GB       0
PC                  1.40
```

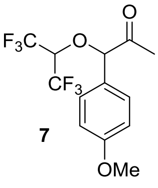

7

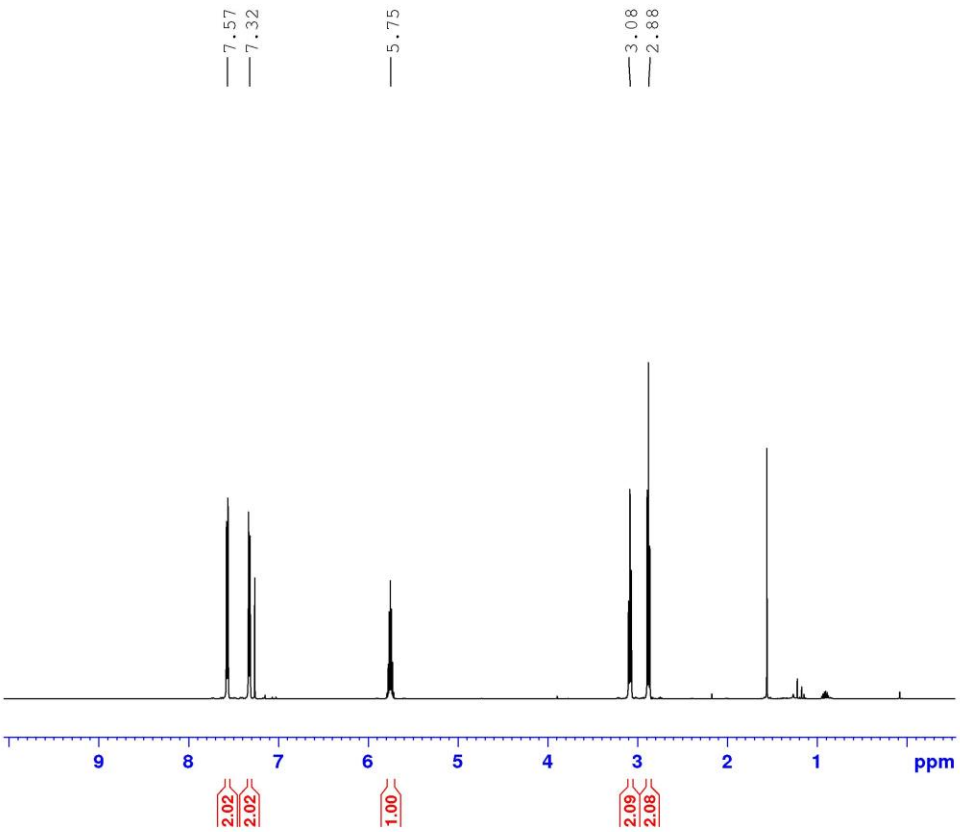


```
Current Data Parameters
NAME           Compound 8
EXPNO                   1
PROCNO                  1

F2 - Acquisition Parameters
Date_            20160928
Time                 9.47
INSTRUM             spect
PROBHD    5 mm PABBO BB-
PULPROG              zg30
TD                  65536
SOLVENT             CDCl3
NS                     16
DS                      2
SWH            10000.000 Hz
FIDRES          0.152588 Hz
AQ             3.2767999 sec
RG                 198.17
DW                 50.000 usec
DE                   6.50 usec
TE                  298.0 K
D1            1.00000000 sec
TD0                     1

======== CHANNEL f1 ========
SFO1         500.1330885 MHz
NUC1                   1H
P1                  10.00 usec
PLW1         26.00000000 W

F2 - Processing parameters
SI                  65536
SF           500.1300121 MHz
WDW                    EM
SSB         0
LB                   0.30 Hz
GB          0
PC                   1.00
```

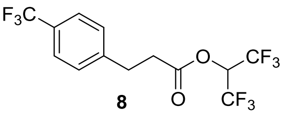

8

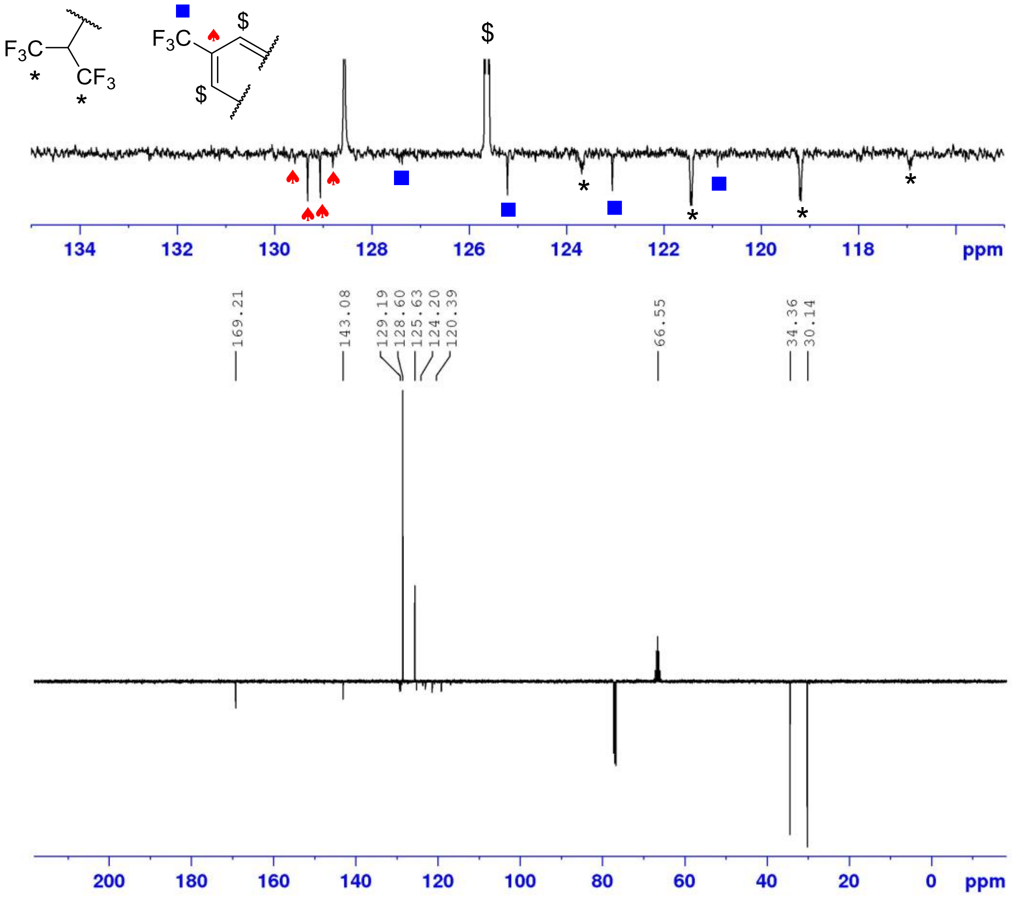


```
Current Data Parameters
NAME           Compound 8
EXPNO                   3
PROCNO                  1

F2 - Acquisition Parameters
Date_            20160928
Time                17.57
INSTRUM             spect
PROBHD    5 mm PABBO BB-
PULPROG              jmod
TD                  65536
SOLVENT             CDCl3
NS                    986
DS                      4
SWH            29761.904 Hz
FIDRES          0.454131 Hz
AQ             1.1010048 sec
RG                 198.17
DW                 16.800 usec
DE                   6.50 usec
TE                  298.0 K
CNST2         145.0000000
CNST11          1.0000000
D1            2.00000000 sec
D20           0.00689655 sec
TD0                     1

======== CHANNEL f1 ========
SFO1         125.7703643 MHz
NUC1                  13C
P1                   8.00 usec
P2                  16.00 usec
PLW1         53.00000000 W

======== CHANNEL f2 ========
SFO2         500.1320005 MHz
NUC2                   1H
CPDPRG[2          waltz16
PCPD2               80.00 usec
PLW2         28.00000000 W
PLW12         0.42017001 W

F2 - Processing parameters
SI                  32768
SF           125.7577890 MHz
WDW                    EM
SSB         0
LB                   1.00 Hz
GB          0
PC                   1.40
```

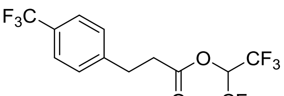

8

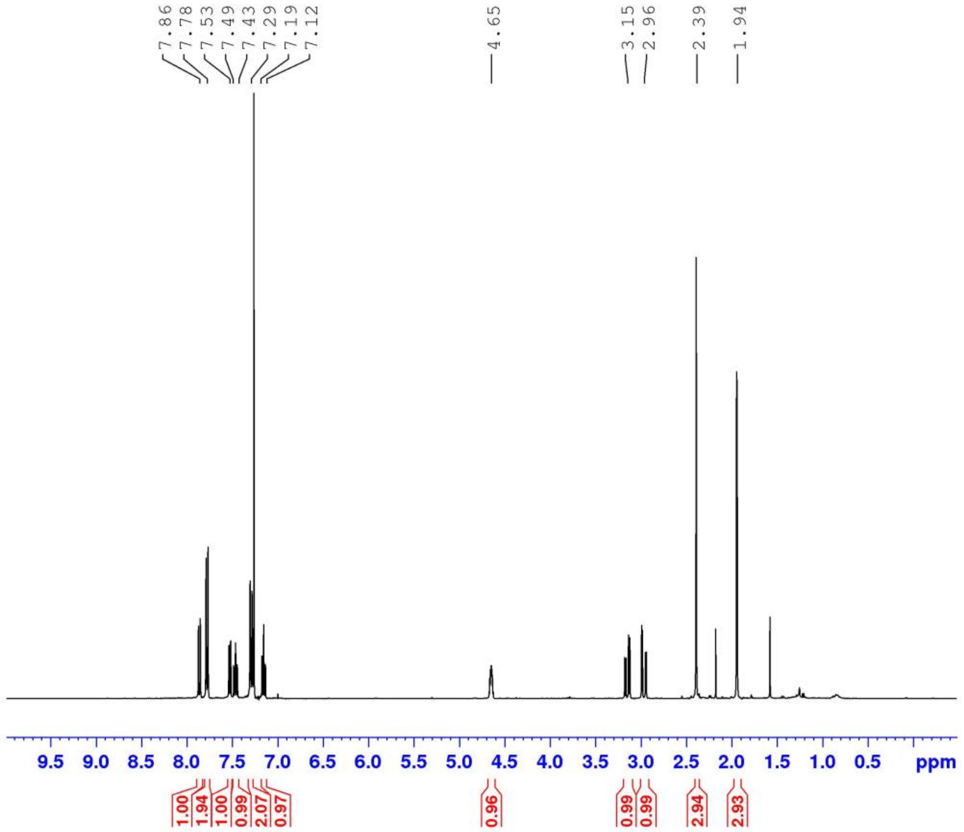

Current Data Parameters
NAME compound 10
EXPNO 7
PROCNO 1

F2 - Acquisition Parameters
Date_ 20190426
Time 10.03
INSTRUM spect
PROBHD 5 mm QNP 1H/1
PULPROG zg30
TD 65536
SOLVENT CDCl3
NS 16
DS 2
SWH 8278.146 Hz
FIDRES 0.126314 Hz
AQ 3.9583745 sec
RG 322.5
DW 60.400 usec
DE 6.50 usec
TE 292.8 K
D1 1.00000000 sec
TD0 1

======== CHANNEL f1 ========
NUC1 1H
P1 10.00 usec
PL1 0.50 dB
SFO1 400.1324710 MHz

F2 - Processing parameters
SI 32768
SF 400.1300100 MHz
WDW EM
SSB 0
LB 0.30 Hz
GB 0
PC 1.00

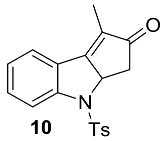

10

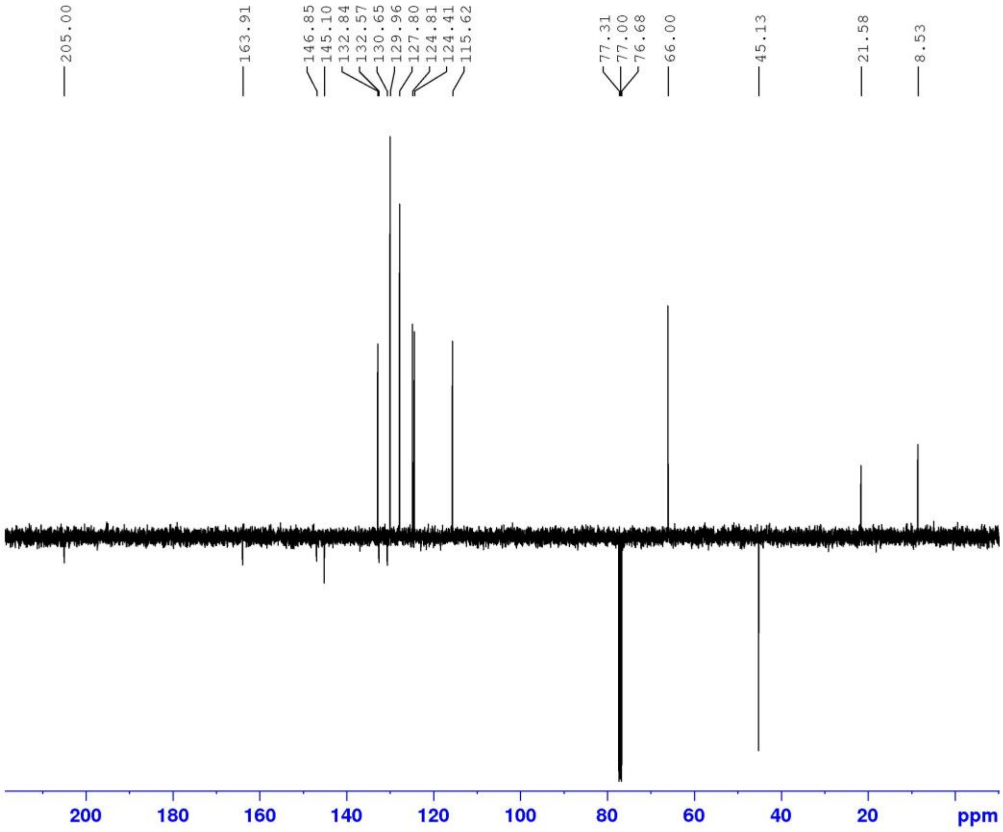

Current Data Parameters
NAME compound 10
EXPNO 3
PROCNO 1

F2 - Acquisition Parameters
Date_ 20190425
Time 16.19
INSTRUM spect
PROBHD 5 mm QNP 1H/1
PULPROG jmod
TD 65536
SOLVENT CDCl3
NS 512
DS 4
SWH 25125.629 Hz
FIDRES 0.383387 Hz
AQ 1.3041664 sec
RG 16384
DW 19.900 usec
DE 6.50 usec
TE 294.3 K
CNST2 145.0000000
CNST11 1.0000000
D1 2.00000000 sec
D20 0.00689655 sec
TD0 1

======== CHANNEL f1 ========
NUC1 13C
P1 6.50 usec
P2 13.00 usec
PL1 0 dB
SFO1 100.6228298 MHz

======== CHANNEL f2 ========
CPDPRG[2 waltz16
NUC2 1H
PCPD2 80.00 usec
PL2 1.70 dB
PL12 19.76 dB
SFO2 400.1316005 MHz

F2 - Processing parameters
SI 32768
SF 100.6127725 MHz
WDW EM
SSB 0
LB 1.00 Hz
GB 0
PC 1.40

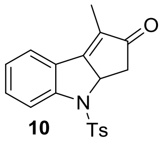

10

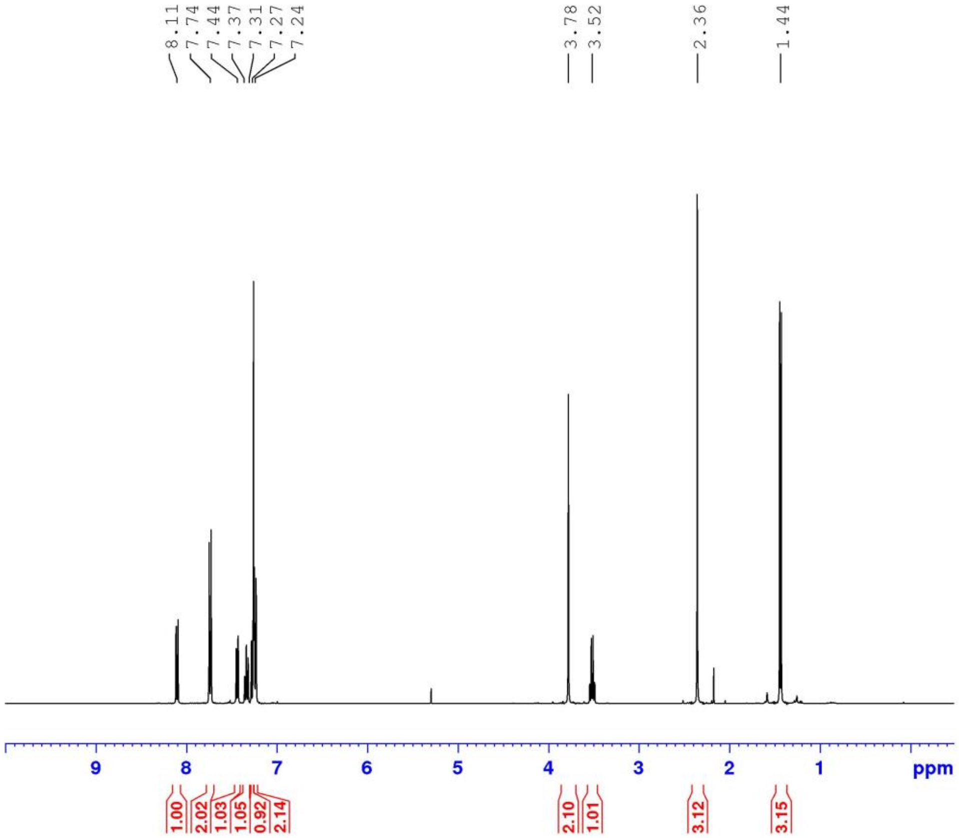


```
Current Data Parameters
NAME          compound 11
EXPNO                   1
PROCNO                  1

F2 - Acquisition Parameters
Date_            20190425
Time                16.04
INSTRUM             spect
PROBHD   5 mm QNP  1H/1
PULPROG              zg30
TD                  65536
SOLVENT             CDCl3
NS                     16
DS                      2
SWH              8278.146 Hz
FIDRES           0.126314 Hz
AQ              3.9583745 sec
RG                    181
DW                 60.400 usec
DE                   6.50 usec
TE                  293.9 K
D1             1.00000000 sec
TD0                     1

======== CHANNEL f1 ========
NUC1                   1H
P1                  10.00 usec
PL1                  0.50 dB
SFO1          400.1324710 MHz

F2 - Processing parameters
SI                  32768
SF            400.1300098 MHz
WDW                    EM
SSB      0
LB                   0.30 Hz
GB       0
PC                   1.00
```

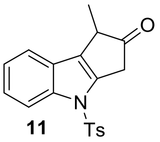
11

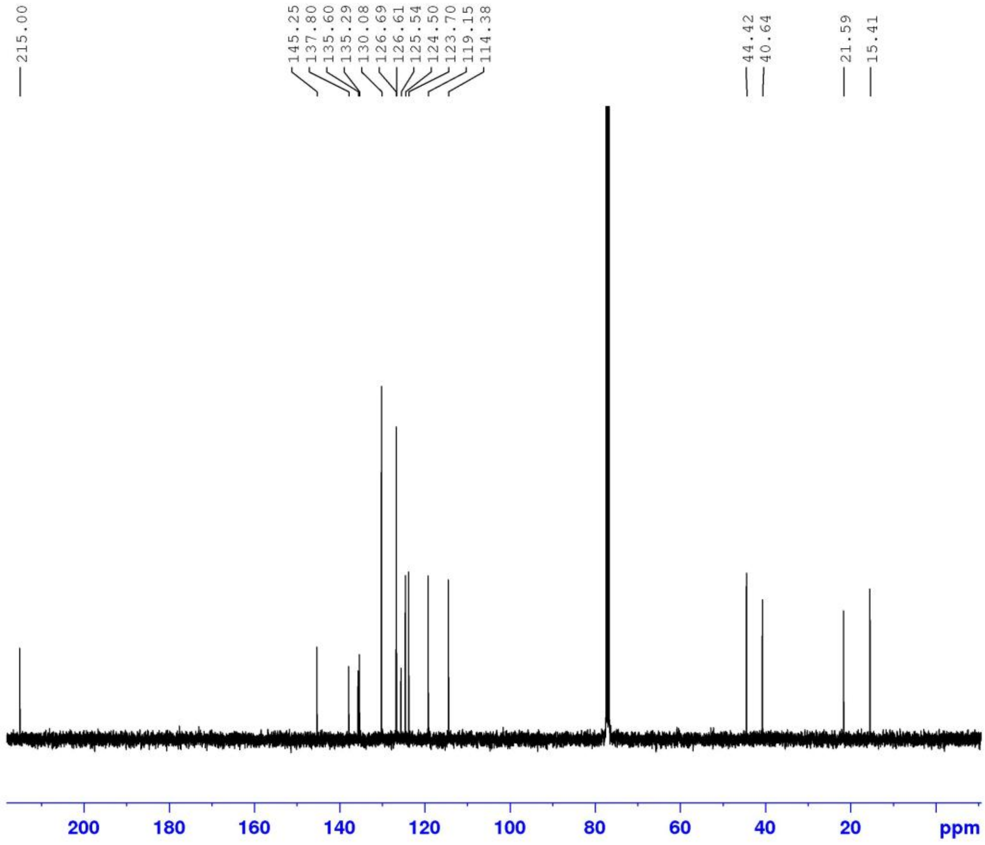


```
Current Data Parameters
NAME          compound 11
EXPNO                   2
PROCNO                  1

F2 - Acquisition Parameters
Date_            20190427
Time                11.32
INSTRUM             spect
PROBHD   5 mm QNP  1H/1
PULPROG            zgpg30
TD                  65536
SOLVENT             CDCl3
NS                    613
DS                      4
SWH             23980.814 Hz
FIDRES           0.365918 Hz
AQ              1.3664256 sec
RG                    128
DW                 20.850 usec
DE                   6.50 usec
TE                  291.2 K
D1             2.00000000 sec
D11            0.03000000 sec
TD0                     1

======== CHANNEL f1 ========
NUC1                  13C
P1                   6.50 usec
PL1        0 dB
SFO1          100.6228298 MHz

======== CHANNEL f2 ========
CPDPRG[2          waltz16
NUC2                   1H
PCPD2               80.00 usec
PL2                  1.70 dB
PL12                19.76 dB
PL13                21.00 dB
SFO2          400.1316005 MHz

F2 - Processing parameters
SI                  32768
SF            100.6127739 MHz
WDW                    EM
SSB      0
LB                   1.00 Hz
GB       0
PC                   1.40
```

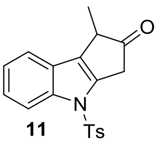
11

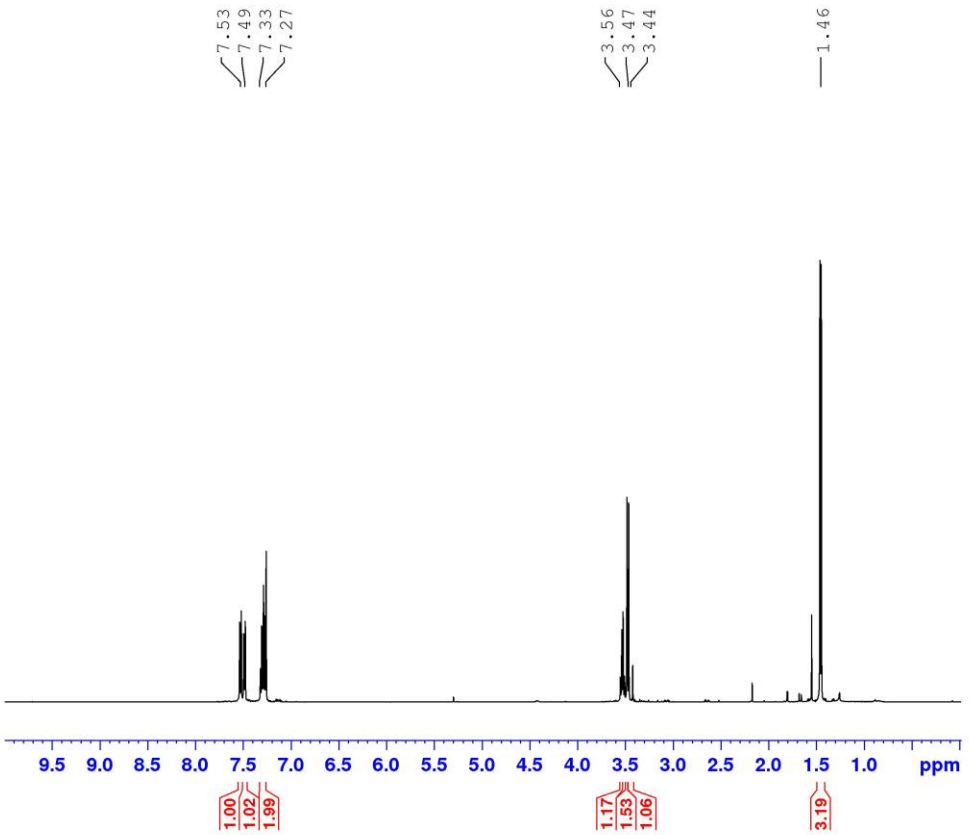


```
Current Data Parameters
NAME          compound 13
EXPNO                   1
PROCNO                  1

F2 - Acquisition Parameters
Date_            20190624
Time                15.08 h
INSTRUM             spect
PROBHD   Z800701_0108 (
PULPROG              zg30
TD                  65536
SOLVENT             CDCl3
NS                     16
DS                      2
SWH             10000.000 Hz
FIDRES           0.305176 Hz
AQ              3.2767999 sec
RG                 198.17
DW                 50.000 usec
DE                  13.89 usec
TE                  298.0 K
D1            1.00000000 sec
TD0                     1
SFO1          500.1330883 MHz
NUC1                   1H
P0                   3.33 usec
P1                  10.00 usec
PLW1          26.00000000 W

F2 - Processing parameters
SI                  65536
SF            500.1300121 MHz
WDW                    EM
SSB      0
LB                   0.30 Hz
GB       0
PC                   1.00
```

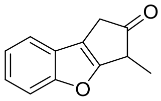

13

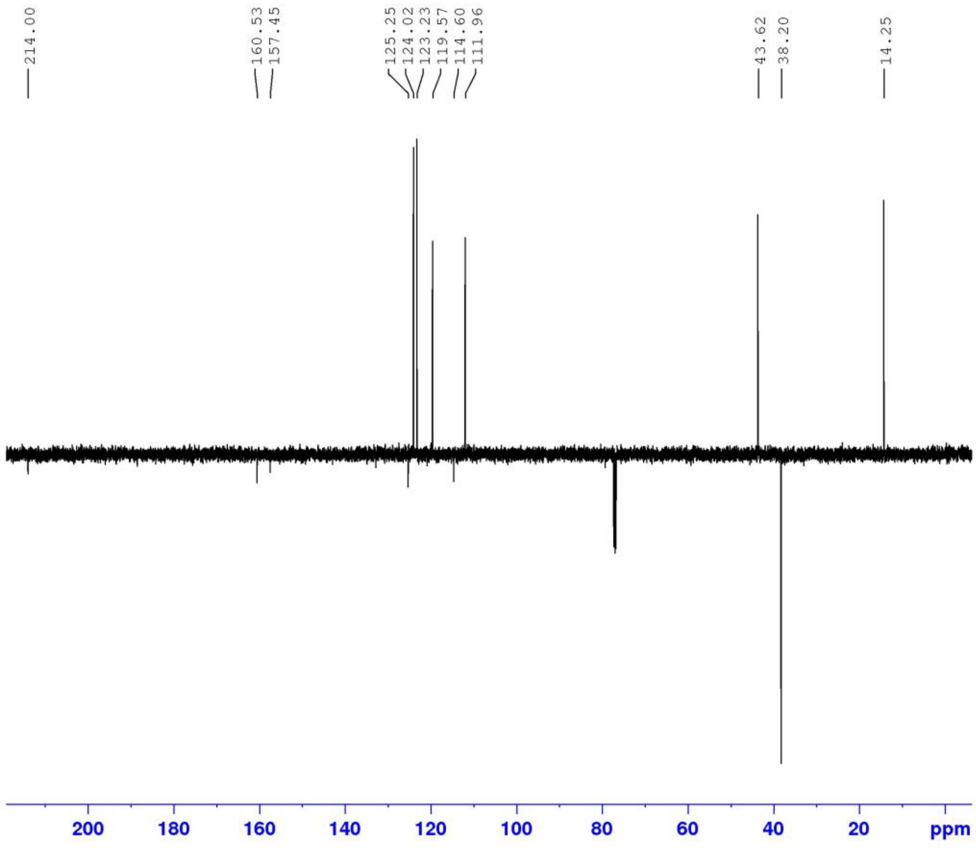


```
Current Data Parameters
NAME          compound 13
EXPNO                   2
PROCNO                  1

F2 - Acquisition Parameters
Date_            20190624
Time                15.21 h
INSTRUM             spect
PROBHD   Z800701_0108 (
PULPROG              jmod
TD                  65536
SOLVENT             CDCl3
NS                     74
DS                      4
SWH             31250.000 Hz
FIDRES           0.953674 Hz
AQ              1.0485760 sec
RG                 198.17
DW                 16.000 usec
DE                   6.50 usec
TE                  298.4 K
CNST2         145.0000000
CNST11          1.0000000
D1            2.00000000 sec
D20           0.00689655 sec
TD0                     1
SFO1          125.7703643 MHz
NUC1                  13C
P1                   8.00 usec
P2                  16.00 usec
PLW1          53.00000000 W
SFO2          500.1320005 MHz
NUC2                   1H
CPDPRG[2          waltz65
PCPD2               80.00 usec
PLW2          28.00000000 W
PLW12          0.42017001 W

F2 - Processing parameters
SI                  32768
SF            125.7577942 MHz
WDW                    EM
SSB      0
LB                   1.00 Hz
GB       0
PC                   1.40
```

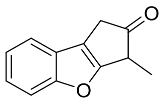

13

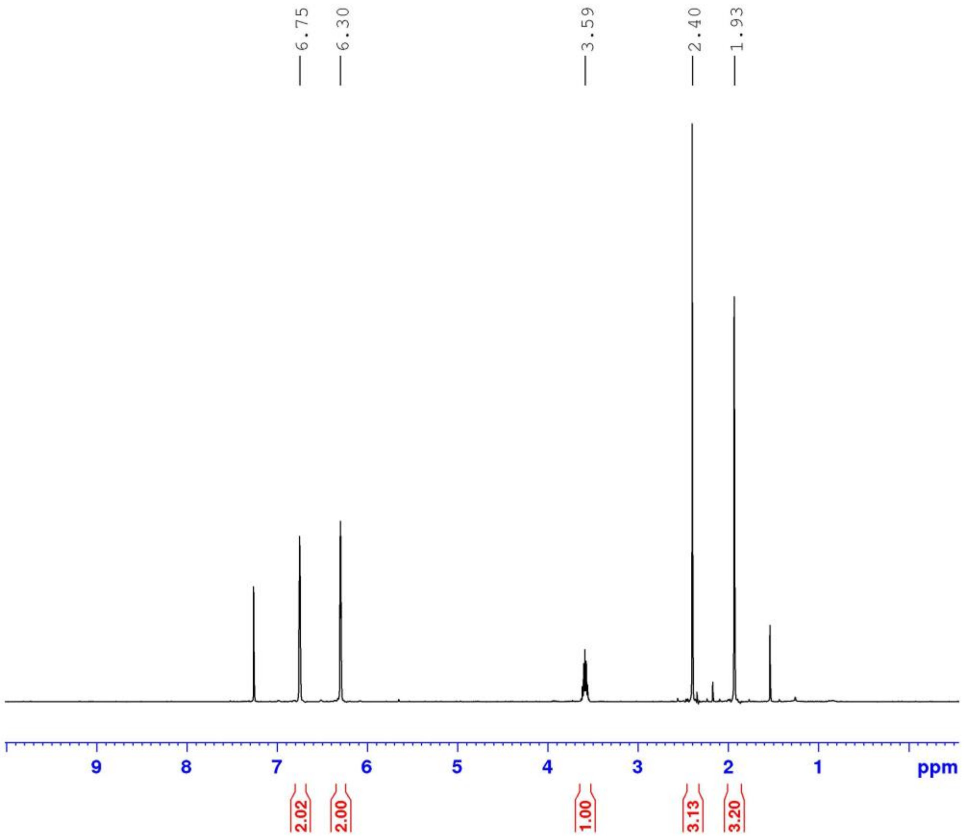

Current Data Parameters
NAME compound 15
EXPNO 1
PROCNO 1

F2 - Acquisition Parameters
Date_ 20190711
Time 13.43
INSTRUM spect
PROBHD 5 mm QNP 1H/1
PULPROG zg30
TD 65536
SOLVENT CDCl3
NS 16
DS 2
SWH 8278.146 Hz
FIDRES 0.126314 Hz
AQ 3.9583745 sec
RG 456.1
DW 60.400 usec
DE 6.50 usec
TE 298.0 K
D1 1.00000000 sec
TD0 1

======== CHANNEL f1 ========
NUC1 1H
P1 10.00 usec
PL1 0.50 dB
SFO1 400.1324710 MHz

F2 - Processing parameters
SI 32768
SF 400.1300112 MHz
WDW EM
SSB 0
LB 0.30 Hz
GB 0
PC 1.00

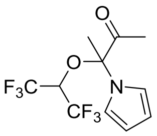

15

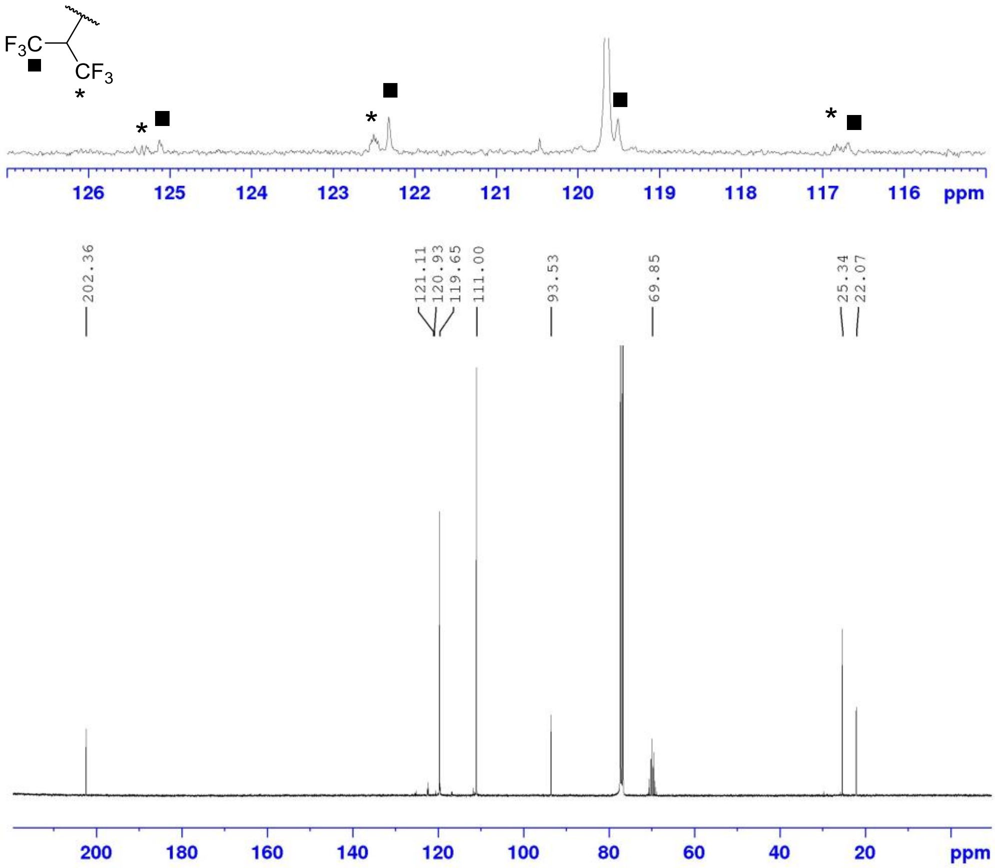

Current Data Parameters
NAME compound 15
EXPNO 4
PROCNO 1

F2 - Acquisition Parameters
Date_ 20190726
Time 8.08
INSTRUM spect
PROBHD 5 mm QNP 1H/1
PULPROG zgpg30
TD 65536
SOLVENT CDCl3
NS 15000
DS 4
SWH 25125.629 Hz
FIDRES 0.383387 Hz
AQ 1.3041664 sec
RG 456.1
DW 19.900 usec
DE 6.50 usec
TE 298.0 K
D1 2.00000000 sec
D11 0.03000000 sec
TD0 1

======== CHANNEL f1 ========
NUC1 13C
P1 6.50 usec
PL1 0 dB
SFO1 100.6228298 MHz

======== CHANNEL f2 ========
CPDPRG[2 waltz16
NUC2 1H
PCPD2 80.00 usec
PL2 1.70 dB
PL12 19.76 dB
PL13 21.00 dB
SFO2 400.1316005 MHz

F2 - Processing parameters
SI 32768
SF 100.6127703 MHz
WDW EM
SSB 0
LB 1.00 Hz
GB 0
PC 1.40

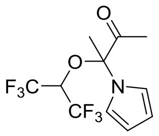

15

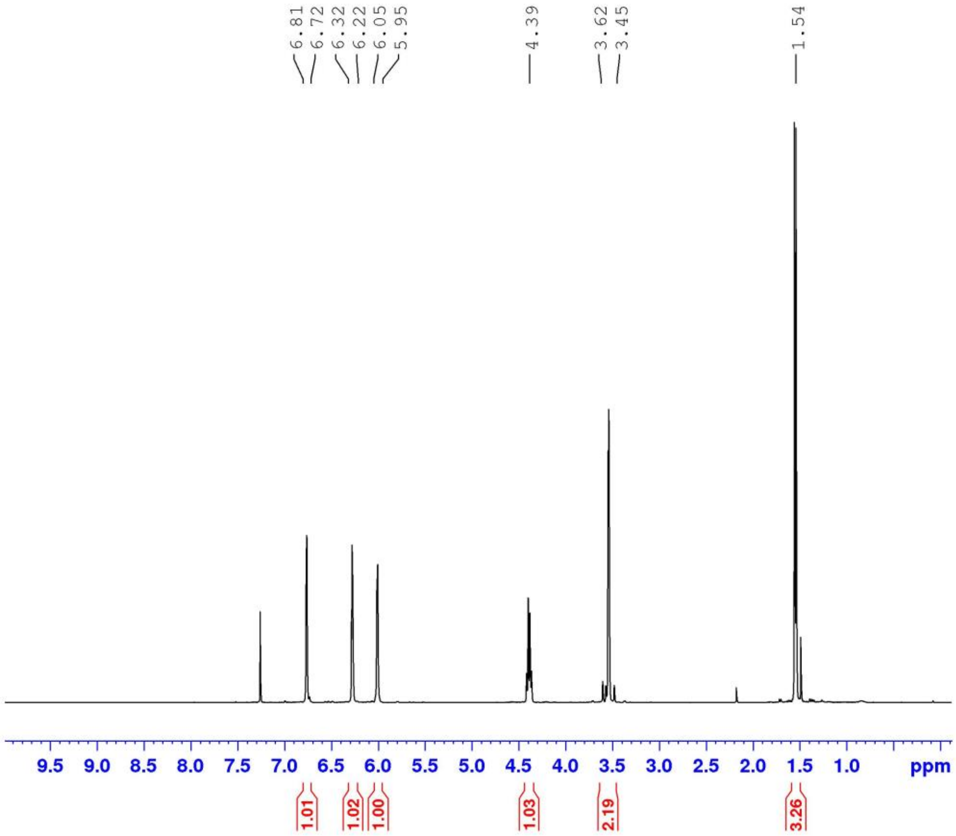


```
Current Data Parameters
NAME          Compound 16
EXPNO                   1
PROCNO                  1

F2 - Acquisition Parameters
Date_            20190708
Time                18.00
INSTRUM             spect
PROBHD    5 mm QNP  1H/1
PULPROG              zg30
TD                  65536
SOLVENT             CDCl3
NS                     16
DS                      2
SWH              8278.146 Hz
FIDRES           0.126314 Hz
AQ              3.9583745 sec
RG                    256
DW                 60.400 usec
DE                   6.50 usec
TE                  298.0 K
D1             1.00000000 sec
TD0                     1

======== CHANNEL f1 ========
NUC1                   1H
P1                  10.00 usec
PL1                  0.50 dB
SFO1          400.1324710 MHz

F2 - Processing parameters
SI                  32768
SF            400.1300112 MHz
WDW                    EM
SSB       0
LB                   0.30 Hz
GB        0
PC                   1.00
```

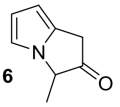
16

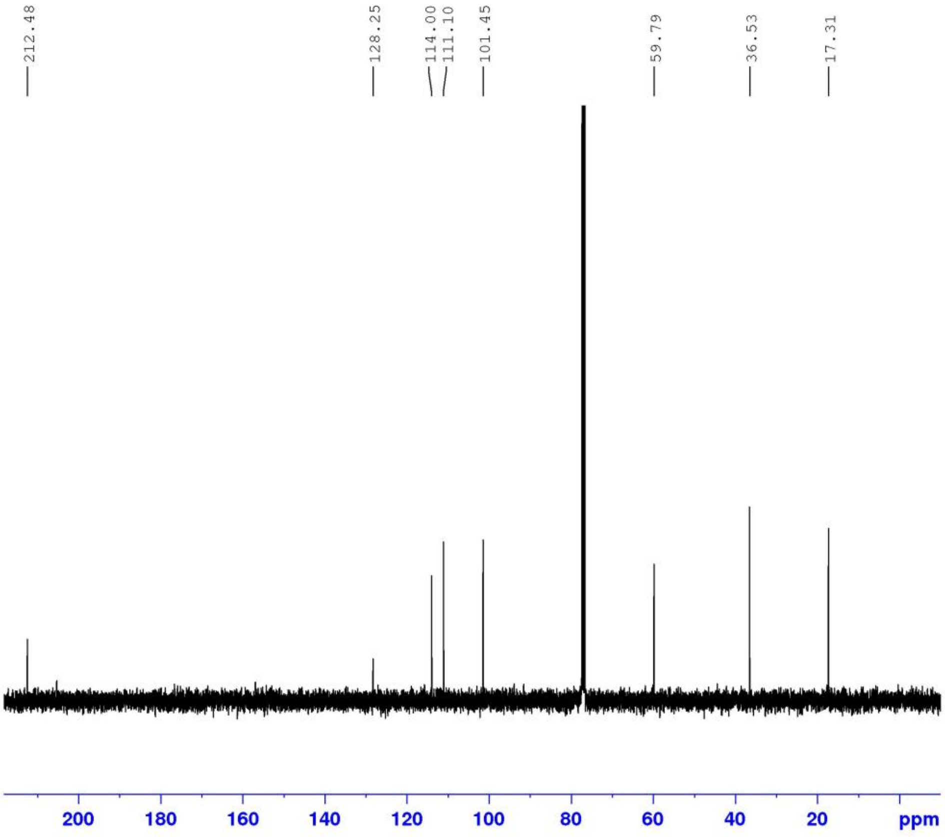


```
Current Data Parameters
NAME          Compound 16
EXPNO                   2
PROCNO                  1

F2 - Acquisition Parameters
Date_            20190708
Time                18.12
INSTRUM             spect
PROBHD    5 mm QNP  1H/1
PULPROG            zgpg30
TD                  65536
SOLVENT             CDCl3
NS                    174
DS                      4
SWH             23980.814 Hz
FIDRES           0.365918 Hz
AQ              1.3664256 sec
RG                  228.1
DW                 20.850 usec
DE                   6.50 usec
TE                  298.0 K
D1             2.00000000 sec
D11            0.03000000 sec
TD0                     1

======== CHANNEL f1 ========
NUC1                  13C
P1                   6.50 usec
PL1       0 dB
SFO1          100.6228298 MHz

======== CHANNEL f2 ========
CPDPRG[2          waltz16
NUC2                   1H
PCPD2               80.00 usec
PL2                  1.70 dB
PL12                19.76 dB
PL13                21.00 dB
SFO2          400.1316005 MHz

F2 - Processing parameters
SI                  32768
SF            100.6127716 MHz
WDW                    EM
SSB       0
LB                   1.00 Hz
GB        0
PC                   1.40
```

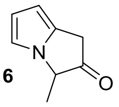
16

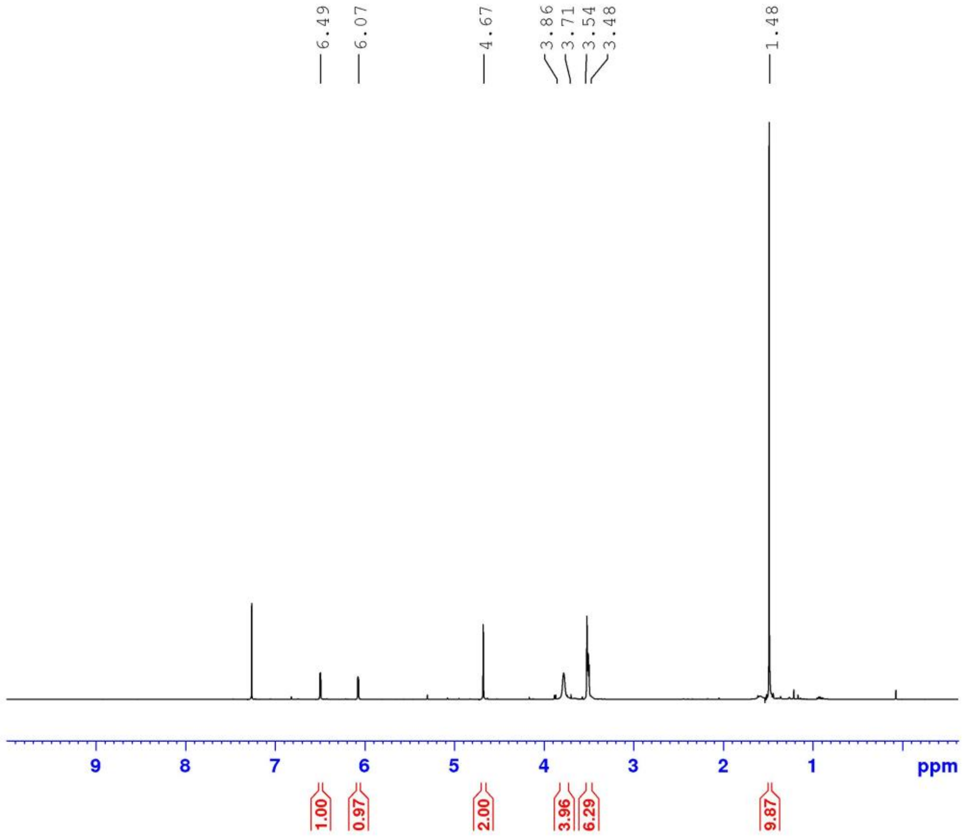


```
Current Data Parameters
NAME        compound 18b
EXPNO                  1
PROCNO                 1

F2 - Acquisition Parameters
Date_           20180730
Time             10.36 h
INSTRUM            spect
PROBHD   Z800701_0108 (
PULPROG             zg30
TD                 65536
SOLVENT            CDCl3
NS                    16
DS                     2
SWH          10000.000 Hz
FIDRES        0.305176 Hz
AQ           3.2767999 sec
RG              198.17
DW              50.000 usec
DE                6.50 usec
TE               298.0 K
D1          1.00000000 sec
TD0                    1
SFO1       500.1330883 MHz
NUC1                  1H
P1               10.00 usec
PLW1       26.00000000 W

F2 - Processing parameters
SI                 65536
SF         500.1300125 MHz
WDW                   EM
SSB      0
LB                0.30 Hz
GB       0
PC                1.00
```

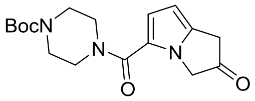

18b

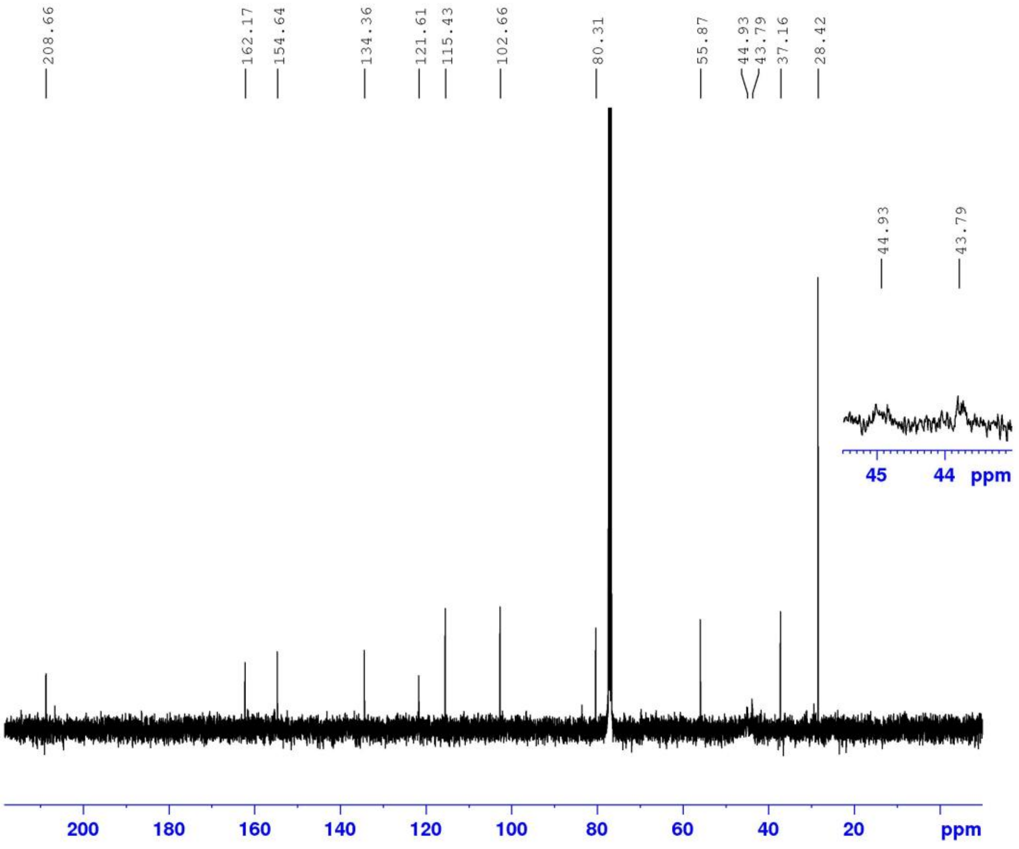


```
Current Data Parameters
NAME        compound 18b
EXPNO                  2
PROCNO                 1

F2 - Acquisition Parameters
Date_           20180831
Time               15.14
INSTRUM            spect
PROBHD   5 mm QNP 1H/13
PULPROG           zgpg30
TD                 65536
SOLVENT            CDCl3
NS                   348
DS                     4
SWH          23980.814 Hz
FIDRES        0.365918 Hz
AQ           1.3664256 sec
RG               645.1
DW              20.850 usec
DE                6.50 usec
TE               312.0 K
D1          4.00000000 sec
D11         0.03000000 sec
TD0                    1

======== CHANNEL f1 ========
NUC1                 13C
P1                8.00 usec
PL1      0 dB
SFO1       100.6228298 MHz

======== CHANNEL f2 ========
CPDPRG[2        waltz16
NUC2                  1H
PCPD2            80.00 usec
PL2               1.00 dB
PL12             19.06 dB
PL13             20.00 dB
SFO2       400.1316005 MHz

F2 - Processing parameters
SI                 32768
SF         100.6127656 MHz
WDW                   EM
SSB      0
LB                1.00 Hz
GB       0
PC                1.40
```

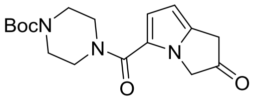

18b

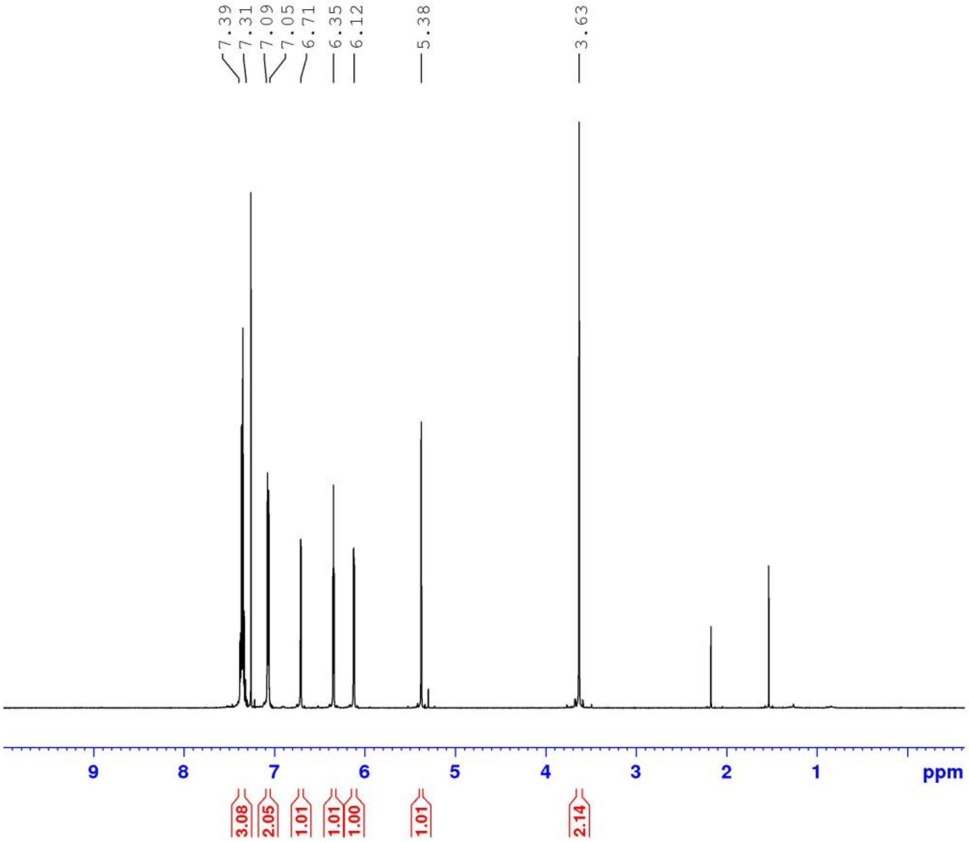


```
Current Data Parameters
NAME         compound 18c
EXPNO                   1
PROCNO                  1

F2 - Acquisition Parameters
Date_            20190226
Time                14.53 h
INSTRUM             spect
PROBHD   Z800701_0108 (
PULPROG              zg30
TD                  65536
SOLVENT             CDCl3
NS                     16
DS                      2
SWH             10000.000 Hz
FIDRES           0.305176 Hz
AQ              3.2767999 sec
RG                 198.17
DW                 50.000 usec
DE                   6.50 usec
TE                  298.0 K
D1             1.00000000 sec
TD0                     1
SFO1          500.1330883 MHz
NUC1                   1H
P1                  10.00 usec
PLW1          26.00000000 W

F2 - Processing parameters
SI                  65536
SF            500.1300120 MHz
WDW                    EM
SSB      0
LB                   0.30 Hz
GB       0
PC                   1.00
```

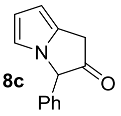

18c

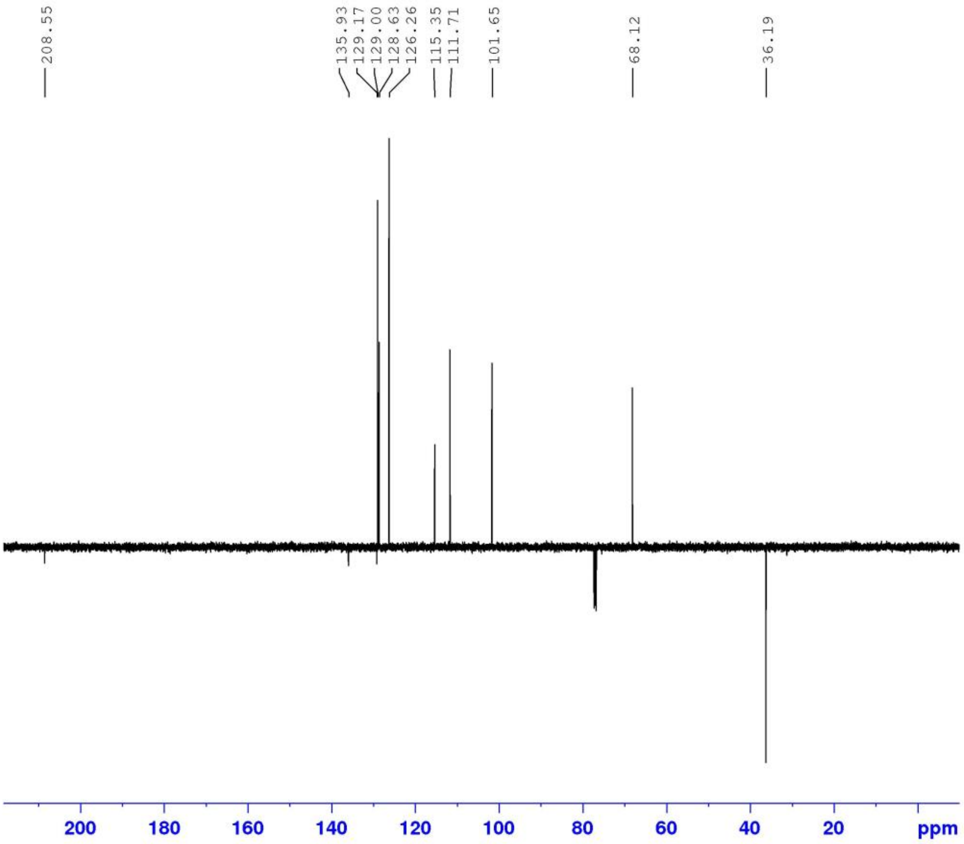


```
Current Data Parameters
NAME         compound 18c
EXPNO                   2
PROCNO                  1

F2 - Acquisition Parameters
Date_            20190226
Time                15.06 h
INSTRUM             spect
PROBHD   Z800701_0108 (
PULPROG              jmod
TD                  65536
SOLVENT             CDCl3
NS                     72
DS                      4
SWH             31250.000 Hz
FIDRES           0.953674 Hz
AQ              1.0485760 sec
RG                 198.17
DW                 16.000 usec
DE                   6.50 usec
TE                  298.0 K
CNST2         145.0000000
CNST11          1.0000000
D1             2.00000000 sec
D20            0.00689655 sec
TD0                     1
SFO1          125.7703643 MHz
NUC1                  13C
P1                   8.00 usec
P2                  16.00 usec
PLW1          53.00000000 W
SFO2          500.1320005 MHz
NUC2                   1H
CPDPRG[2          waltz16
PCPD2               80.00 usec
PLW2          28.00000000 W
PLW12          0.42017001 W

F2 - Processing parameters
SI                  32768
SF            125.7577959 MHz
WDW                    EM
SSB      0
LB                   1.00 Hz
GB       0
PC                   1.40
```

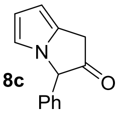

18c

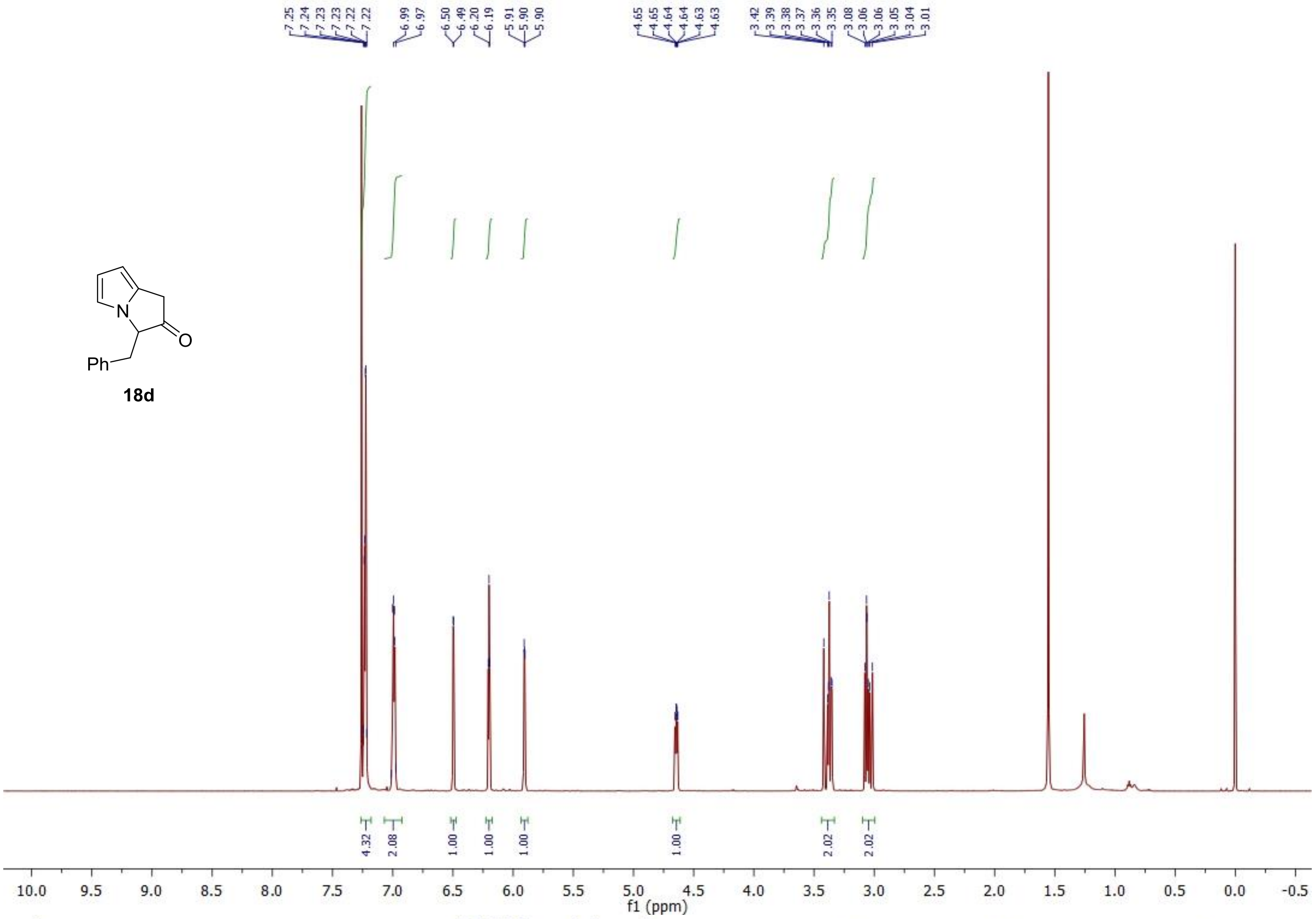
7.25
7.24
7.23
7.23
7.22
7.22
6.99
6.97
6.50
6.49
6.20
6.19
5.91
5.90
5.90
4.65
4.65
4.64
4.64
4.63
4.63
3.42
3.39
3.38
3.37
3.36
3.35
3.08
3.06
3.06
3.05
3.04
3.01
N
O
Ph
18d
4.32
2.08
1.00
1.00
1.00
1.00
2.02
2.02
f1 (ppm)

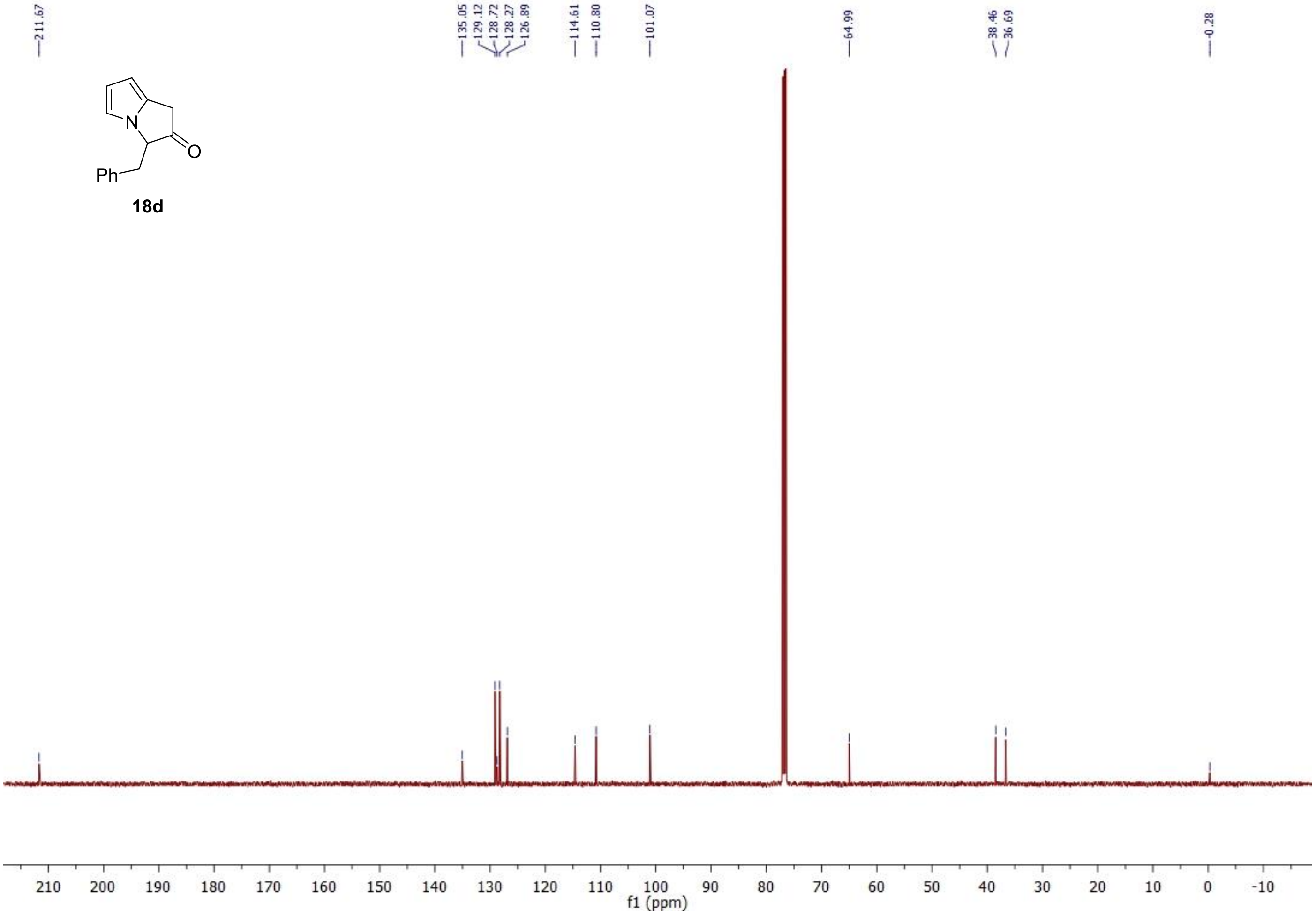
211.67
135.05
129.12
128.72
128.27
126.89
114.61
110.80
101.07
64.99
38.46
36.69
-0.28
N
O
Ph
18d
f1 (ppm)

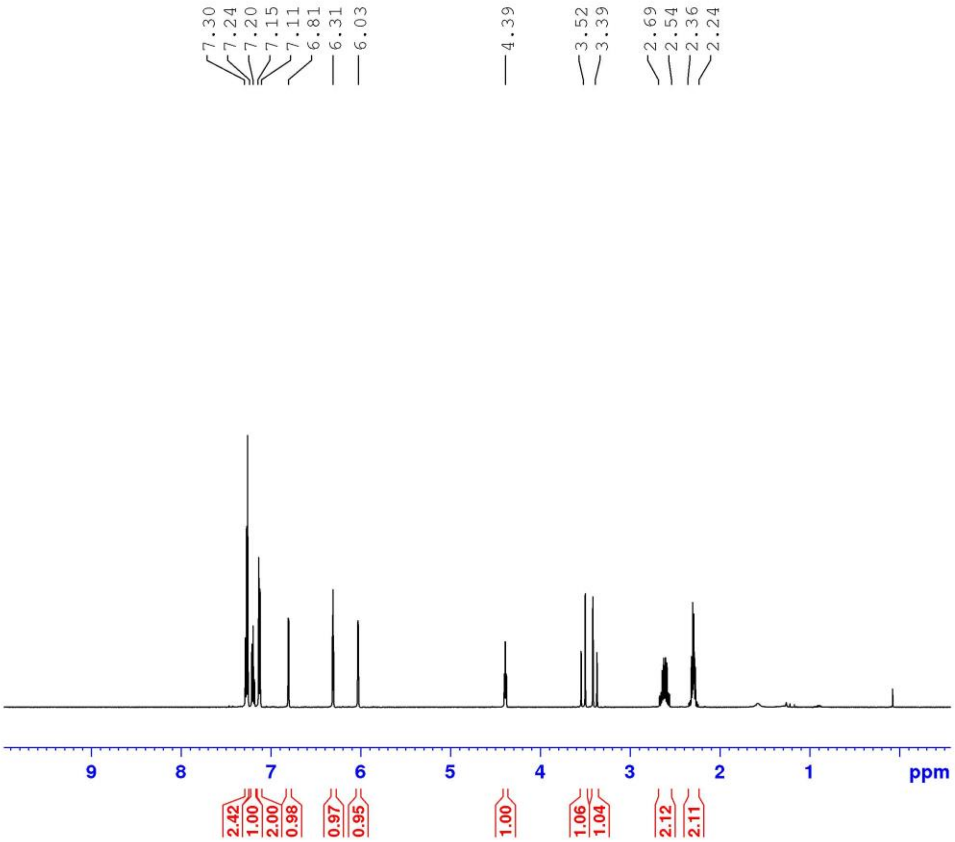

Current Data Parameters
NAME compound 18 e
EXPNO 1
PROCNO 1

F2 - Acquisition Parameters
Date_ 20180110
Time 11.08 h
INSTRUM spect
PROBHD Z800701_0108 (
PULPROG zg30
TD 65536
SOLVENT CDCl3
NS 16
DS 2
SWH 10000.000 Hz
FIDRES 0.305176 Hz
AQ 3.2767999 sec
RG 198.17
DW 50.000 usec
DE 6.50 usec
TE 298.0 K
D1 1.00000000 sec
TD0 1
SFO1 500.1330883 MHz
NUC1 1H
P1 10.00 usec
PLW1 26.00000000 W

F2 - Processing parameters
SI 65536
SF 500.1300122 MHz
WDW EM
SSB 0
LB 0.30 Hz
GB 0
PC 1.00

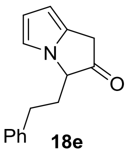

18e

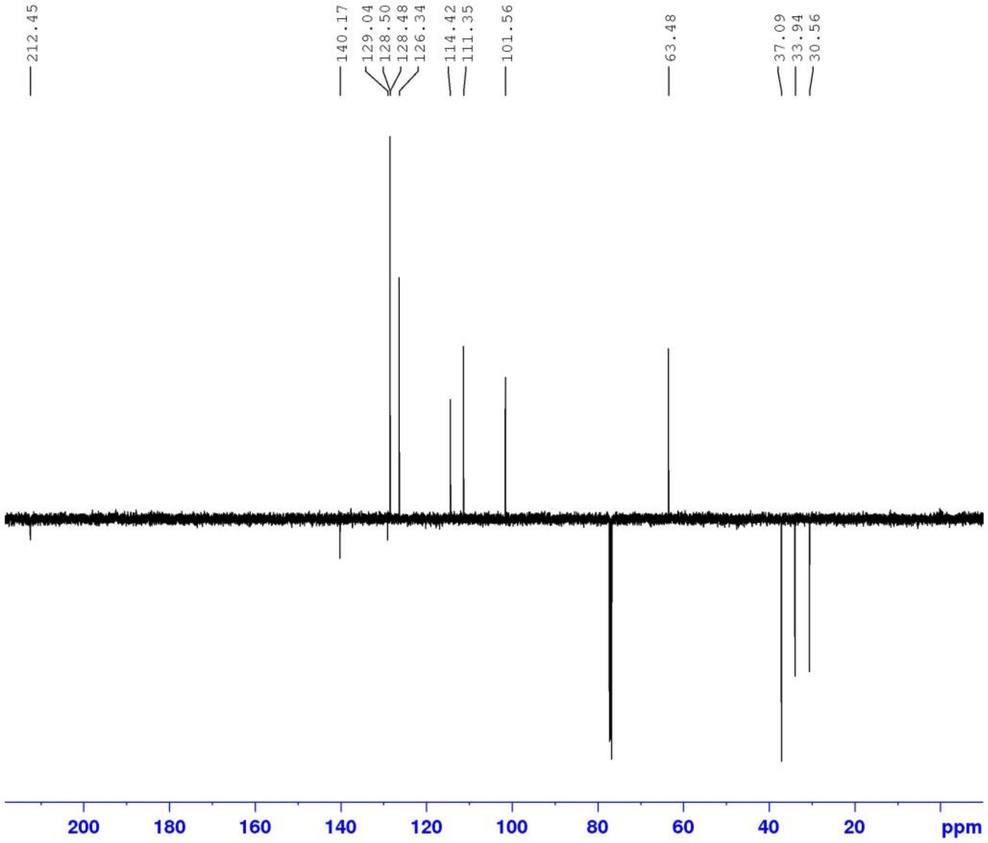

Current Data Parameters
NAME compound 18 e
EXPNO 3
PROCNO 1

F2 - Acquisition Parameters
Date_ 20180110
Time 11.42 h
INSTRUM spect
PROBHD Z800701_0108 (
PULPROG jmod
TD 65536
SOLVENT CDCl3
NS 512
DS 4
SWH 31250.000 Hz
FIDRES 0.953674 Hz
AQ 1.0485760 sec
RG 198.17
DW 16.000 usec
DE 6.50 usec
TE 298.0 K
CNST2 145.0000000
CNST11 1.0000000
D1 2.00000000 sec
D20 0.00689655 sec
TD0 1
SFO1 125.7703643 MHz
NUC1 13C
P1 8.00 usec
P2 16.00 usec
PLW1 53.00000000 W
SFO2 500.1320005 MHz
NUC2 1H
CPDPRG[2 waltz16
PCPD2 80.00 usec
PLW2 28.00000000 W
PLW12 0.42017001 W

F2 - Processing parameters
SI 32768
SF 125.7577921 MHz
WDW EM
SSB 0
LB 1.00 Hz
GB 0
PC 1.40

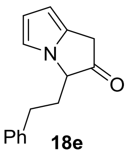

18e

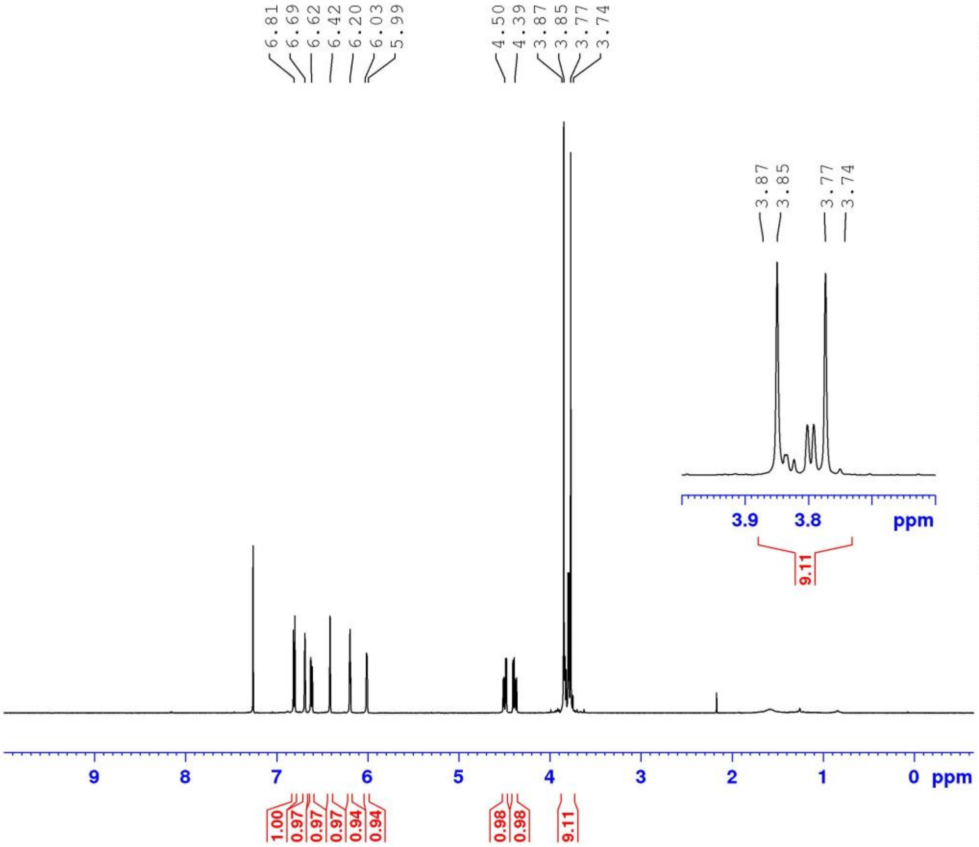


```
Current Data Parameters
NAME        Compound 18f
EXPNO                  2
PROCNO                 1

F2 - Acquisition Parameters
Date_           20190731
Time                8.50 h
INSTRUM            spect
PROBHD    Z800701_0108 (
PULPROG             zg30
TD                 65536
SOLVENT            CDCl3
NS                    16
DS                     2
SWH            10000.000 Hz
FIDRES          0.305176 Hz
AQ             3.2767999 sec
RG                198.17
DW                50.000 usec
DE                 13.89 usec
TE                 298.0 K
D1            1.00000000 sec
TD0                    1
SFO1         500.1330883 MHz
NUC1                  1H
P0                  3.33 usec
P1                 10.00 usec
PLW1         26.00000000 W

F2 - Processing parameters
SI                 65536
SF           500.1300121 MHz
WDW                   EM
SSB       0
LB                  0.30 Hz
GB        0
PC                  1.00
```

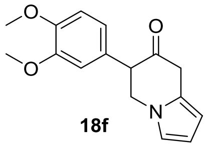

18f

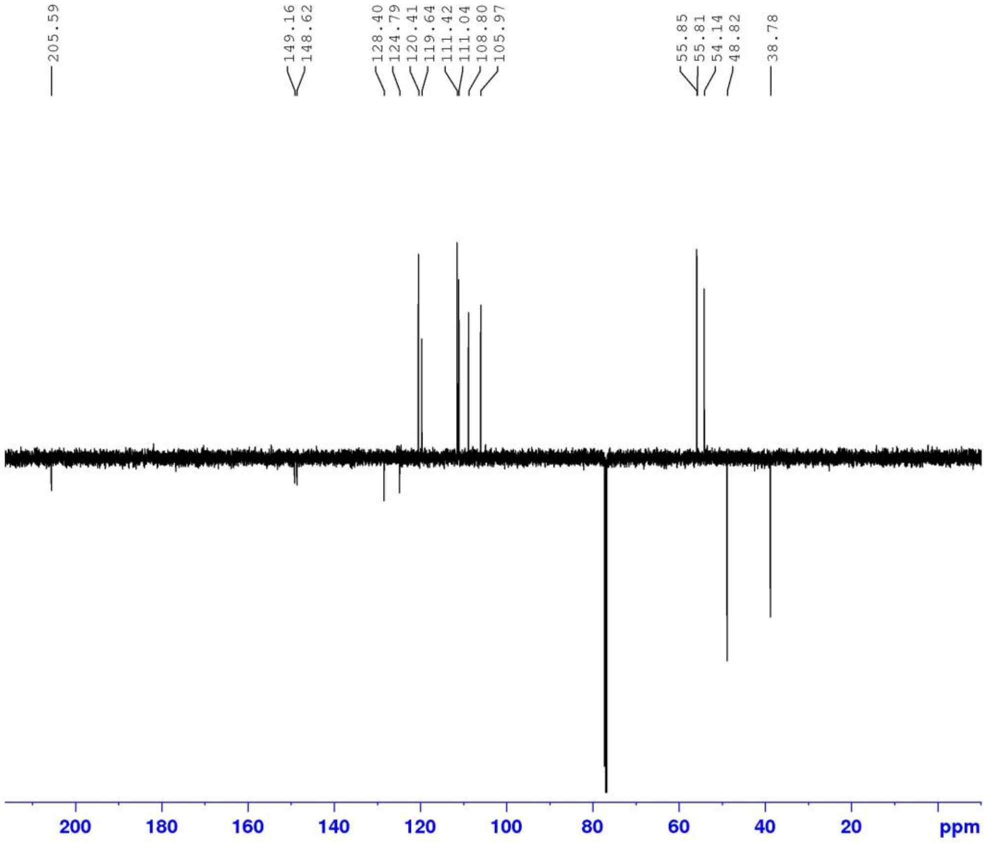


```
Current Data Parameters
NAME         compound 18 f
EXPNO                  3
PROCNO                 1

F2 - Acquisition Parameters
Date_           20180314
Time               13.37 h
INSTRUM            spect
PROBHD    Z800701_0108 (
PULPROG             jmod
TD                 65536
SOLVENT            CDCl3
NS                   512
DS                     4
SWH            29761.904 Hz
FIDRES          0.908261 Hz
AQ             1.1010048 sec
RG                198.17
DW                16.800 usec
DE                  6.50 usec
TE                 298.0 K
CNST2        145.0000000
CNST11         1.0000000
D1            2.00000000 sec
D20           0.00689655 sec
TD0                    1
SFO1         125.7703643 MHz
NUC1                 13C
P1                  8.00 usec
P2                 16.00 usec
PLW1         53.00000000 W
SFO2         500.1320005 MHz
NUC2                  1H
CPDPRG[2         waltz16
PCPD2              80.00 usec
PLW2         28.00000000 W
PLW12         0.42017001 W

F2 - Processing parameters
SI                 32768
SF           125.7577923 MHz
WDW                   EM
SSB       0
LB                  1.00 Hz
GB        0
PC                  1.40
```

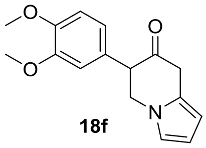

18f

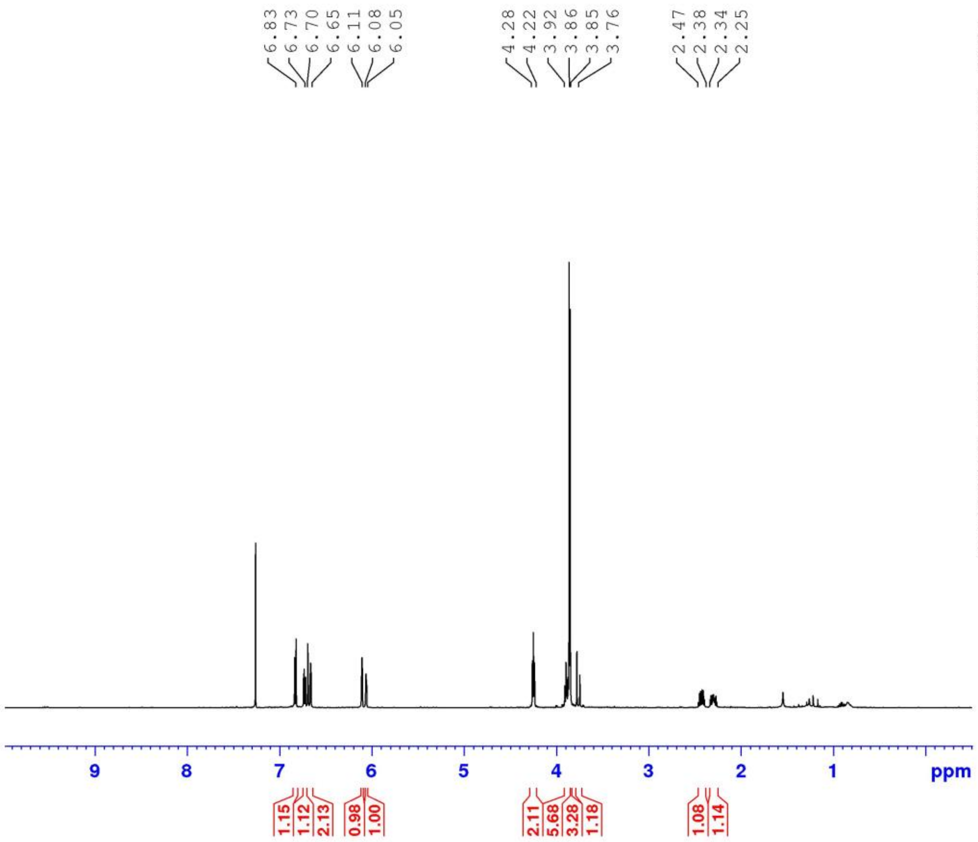

Current Data Parameters
NAME compound 18 g
EXPNO 4
PROCNO 1

F2 - Acquisition Parameters
Date_ 20180810
Time 16.54 h
INSTRUM spect
PROBHD Z800701_0108 (
PULPROG zg30
TD 65536
SOLVENT CDCl3
NS 16
DS 2
SWH 10000.000 Hz
FIDRES 0.305176 Hz
AQ 3.2767999 sec
RG 198.17
DW 50.000 usec
DE 6.50 usec
TE 298.0 K
D1 1.00000000 sec
TD0 1
SFO1 500.1330883 MHz
NUC1 1H
P1 10.00 usec
PLW1 26.00000000 W

F2 - Processing parameters
SI 65536
SF 500.1300125 MHz
WDW EM
SSB 0
LB 0.30 Hz
GB 0
PC 1.00

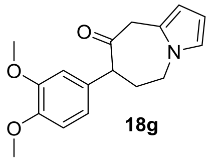

18g

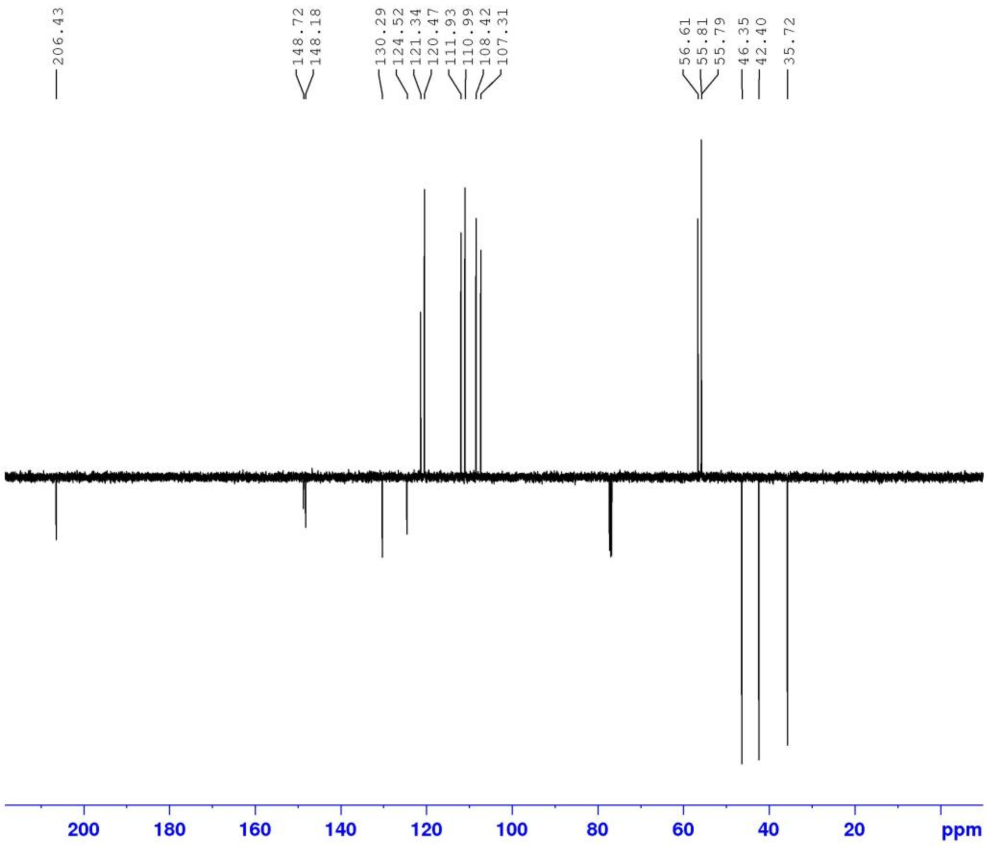

Current Data Parameters
NAME compound 18 g
EXPNO 2
PROCNO 1

F2 - Acquisition Parameters
Date_ 20180807
Time 15.57 h
INSTRUM spect
PROBHD Z800701_0108 (
PULPROG jmod
TD 65536
SOLVENT CDCl3
NS 79
DS 4
SWH 31250.000 Hz
FIDRES 0.953674 Hz
AQ 1.0485760 sec
RG 198.17
DW 16.000 usec
DE 6.50 usec
TE 298.0 K
CNST2 145.0000000
CNST11 1.0000000
D1 2.00000000 sec
D20 0.00689655 sec
TD0 1
SFO1 125.7703643 MHz
NUC1 13C
P1 8.00 usec
P2 16.00 usec
PLW1 53.00000000 W
SFO2 500.1320005 MHz
NUC2 1H
CPDPRG[2 waltz16
PCPD2 80.00 usec
PLW2 28.00000000 W
PLW12 0.42017001 W

F2 - Processing parameters
SI 32768
SF 125.7578005 MHz
WDW EM
SSB 0
LB 1.00 Hz
GB 0
PC 1.40

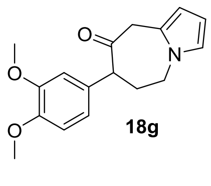

18g

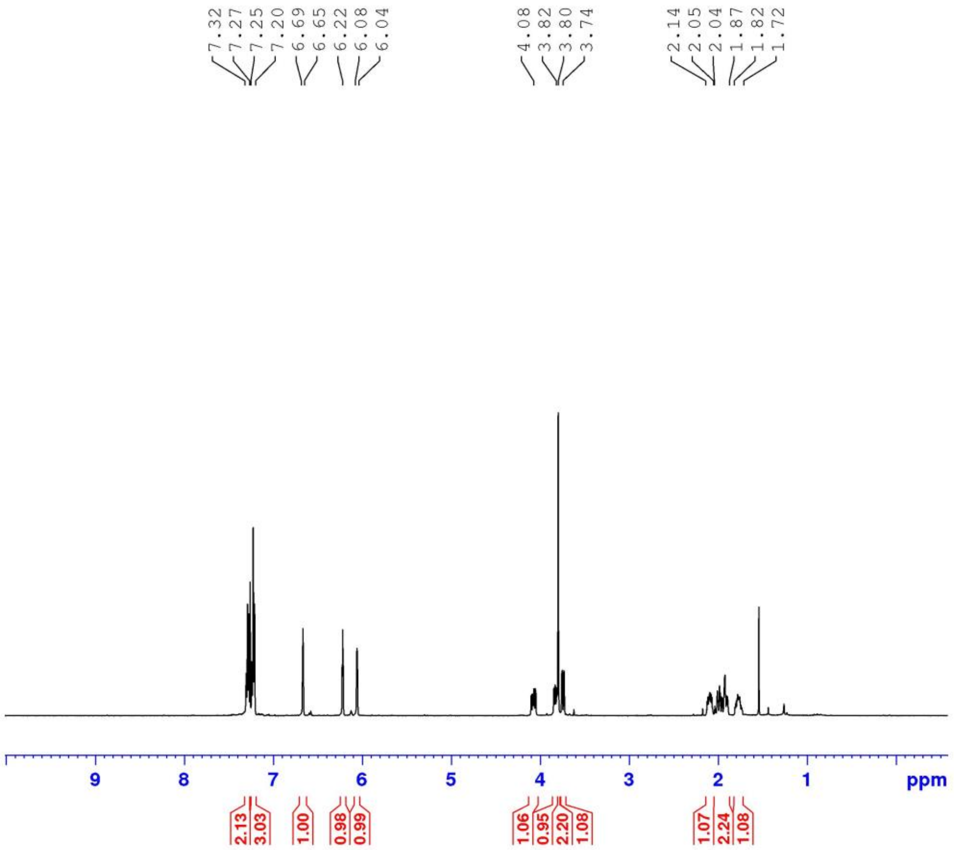

Current Data Parameters
NAME compound_18h
EXPNO 3
PROCNO 1

F2 - Acquisition Parameters
Date_ 20190819
Time 18.38 h
INSTRUM spect
PROBHD Z800701_0108 (
PULPROG zg30
TD 65536
SOLVENT CDCl3
NS 16
DS 2
SWH 10000.000 Hz
FIDRES 0.305176 Hz
AQ 3.2767999 sec
RG 198.17
DW 50.000 usec
DE 13.89 usec
TE 298.0 K
D1 1.00000000 sec
TD0 1
SFO1 500.1330883 MHz
NUC1 1H
P0 3.33 usec
P1 10.00 usec
PLW1 26.00000000 W

F2 - Processing parameters
SI 65536
SF 500.1300119 MHz
WDW EM
SSB 0
LB 0.30 Hz
GB 0
PC 1.00

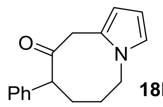

18h

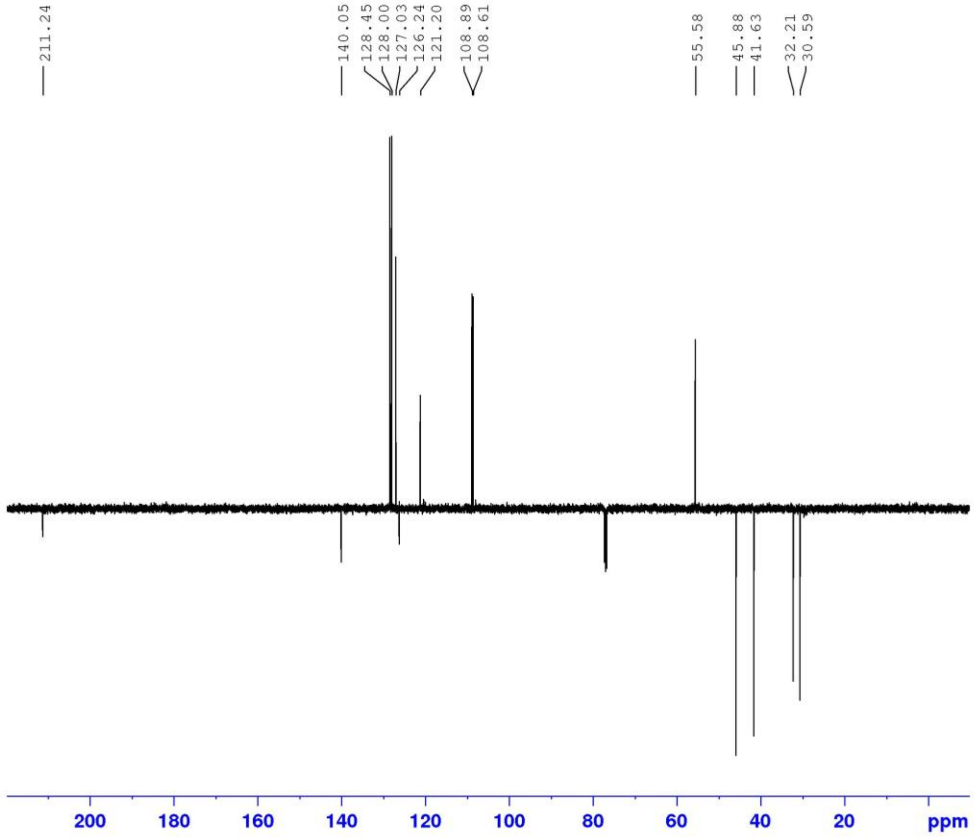

Current Data Parameters
NAME compound_18h
EXPNO 2
PROCNO 1

F2 - Acquisition Parameters
Date_ 20190819
Time 17.02 h
INSTRUM spect
PROBHD Z800701_0108 (
PULPROG jmod
TD 65536
SOLVENT CDCl3
NS 64
DS 4
SWH 31250.000 Hz
FIDRES 0.953674 Hz
AQ 1.0485760 sec
RG 198.17
DW 16.000 usec
DE 6.50 usec
TE 298.3 K
CNST2 145.0000000
CNST11 1.0000000
D1 2.00000000 sec
D20 0.00689655 sec
TD0 1
SFO1 125.7703643 MHz
NUC1 13C
P1 8.00 usec
P2 16.00 usec
PLW1 53.00000000 W
SFO2 500.1320005 MHz
NUC2 1H
CPDPRG[2 waltz65
PCPD2 80.00 usec
PLW2 28.00000000 W
PLW12 0.42017001 W

F2 - Processing parameters
SI 32768
SF 125.7577978 MHz
WDW EM
SSB 0
LB 1.00 Hz
GB 0
PC 1.40

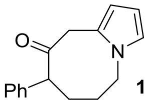

18h

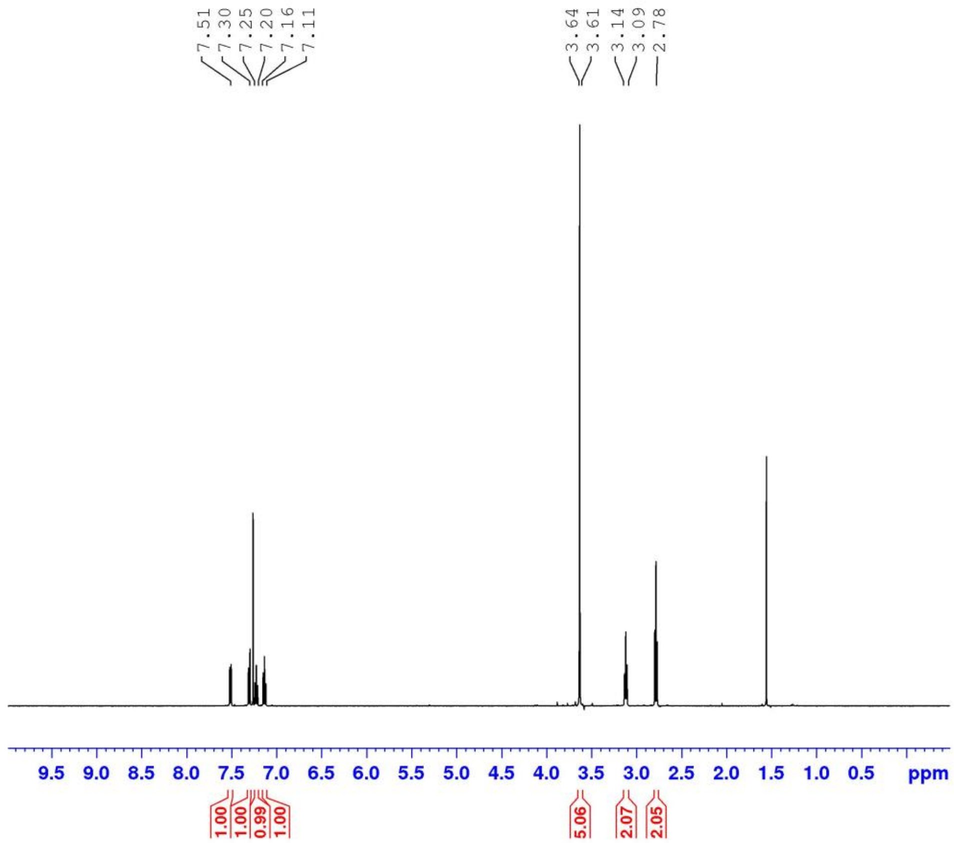


```
Current Data Parameters
NAME          compound 20
EXPNO                   1
PROCNO                  1

F2 - Acquisition Parameters
Date_            20170510
Time                17.12
INSTRUM             spect
PROBHD   5 mm PABBO BB-
PULPROG              zg30
TD                  65536
SOLVENT             CDCl3
NS                     16
DS                      2
SWH             10000.000 Hz
FIDRES           0.152588 Hz
AQ              3.2767999 sec
RG                 198.17
DW                 50.000 usec
DE                   6.50 usec
TE                  298.0 K
D1             1.00000000 sec
TD0                     1

======== CHANNEL f1 ========
SFO1         500.1330885 MHz
NUC1                   1H
P1                  10.00 usec
PLW1          26.00000000 W

F2 - Processing parameters
SI                  65536
SF            500.1300123 MHz
WDW                    EM
SSB      0
LB                   0.30 Hz
GB       0
PC                   1.00
```

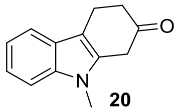
20

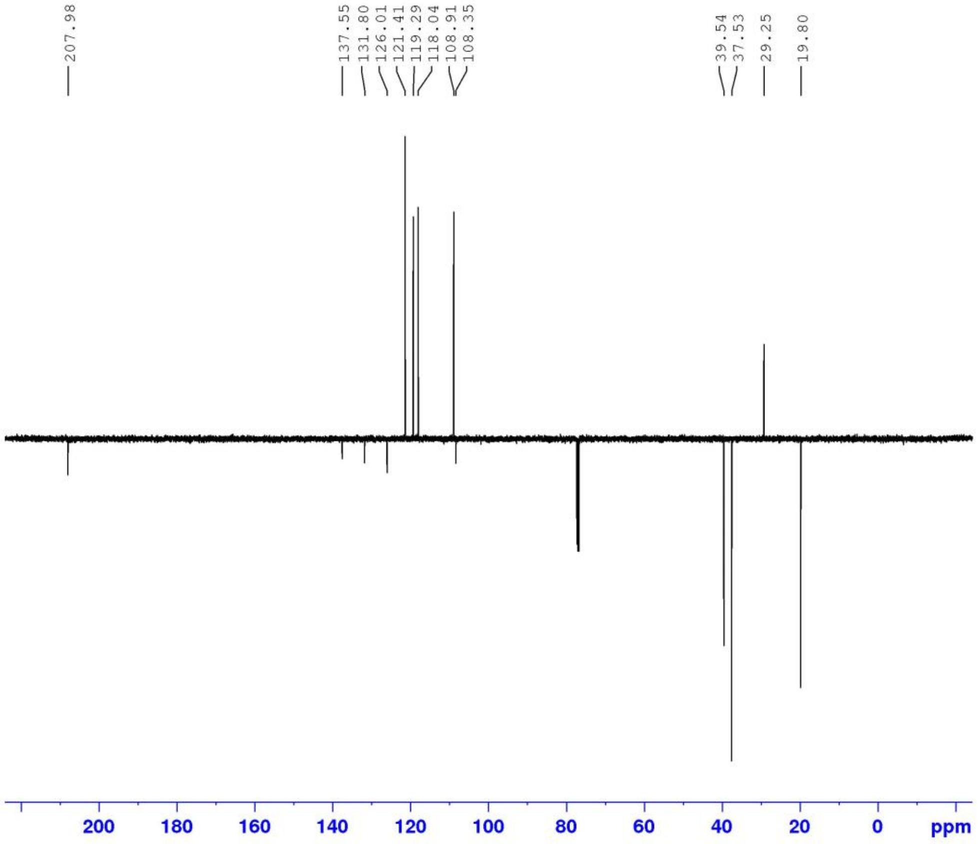


```
Current Data Parameters
NAME          compound 20
EXPNO                   2
PROCNO                  1

F2 - Acquisition Parameters
Date_            20170511
Time                 9.30
INSTRUM             spect
PROBHD   5 mm PABBO BB-
PULPROG              jmod
TD                  65536
SOLVENT             CDCl3
NS                    512
DS                      4
SWH             31250.000 Hz
FIDRES           0.476837 Hz
AQ              1.0485760 sec
RG                 198.17
DW                 16.000 usec
DE                   6.50 usec
TE                  298.0 K
CNST2         145.0000000
CNST11          1.0000000
D1             2.00000000 sec
D20            0.00689655 sec
TD0                     1

======== CHANNEL f1 ========
SFO1         125.7703643 MHz
NUC1                  13C
P1                   8.00 usec
P2                  16.00 usec
PLW1          53.00000000 W

======== CHANNEL f2 ========
SFO2         500.1320005 MHz
NUC2                   1H
CPDPRG[2          waltz16
PCPD2               80.00 usec
PLW2          28.00000000 W
PLW12          0.42017001 W

F2 - Processing parameters
SI                  32768
SF            125.7577954 MHz
WDW                    EM
SSB      0
LB                   1.00 Hz
GB       0
PC                   1.40
```

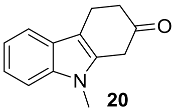
20

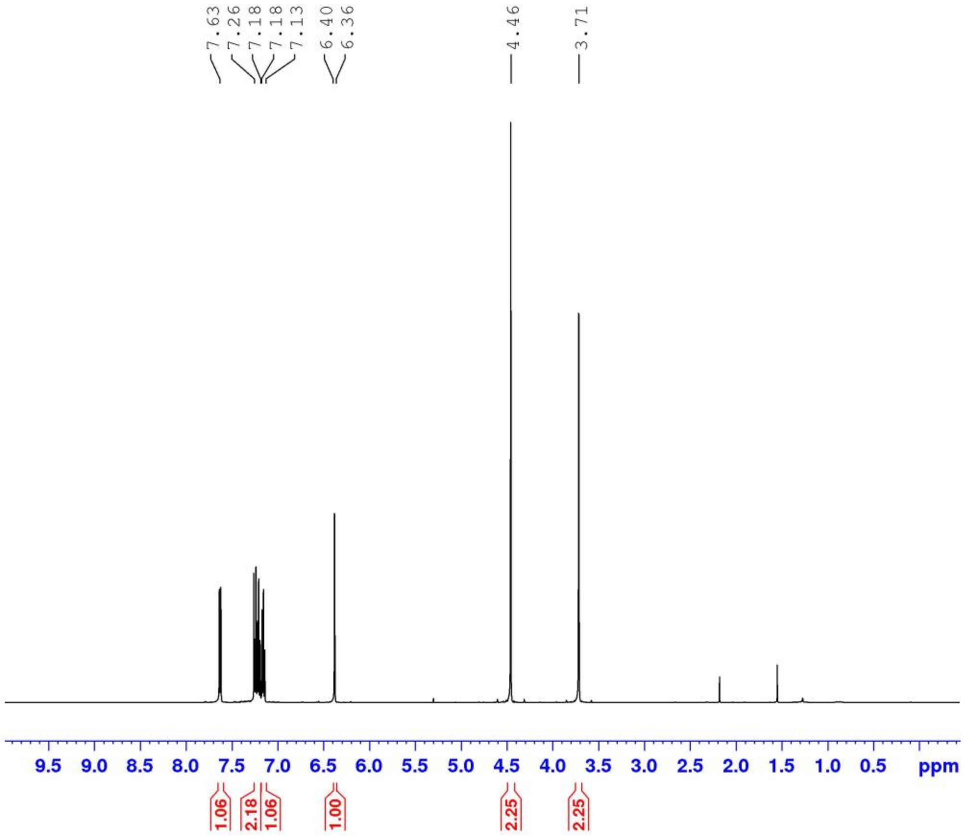


```
Current Data Parameters
NAME          Compound 22
EXPNO                   1
PROCNO                  1

F2 - Acquisition Parameters
Date_            20190508
Time                 8.58 h
INSTRUM             spect
PROBHD   Z800701_0108 (
PULPROG              zg30
TD                  65536
SOLVENT             CDCl3
NS                     16
DS                      2
SWH             10000.000 Hz
FIDRES           0.305176 Hz
AQ              3.2767999 sec
RG                 198.17
DW                 50.000 usec
DE                   6.50 usec
TE                  298.0 K
D1             1.00000000 sec
TD0                     1
SFO1          500.1330883 MHz
NUC1                   1H
P1                  10.00 usec
PLW1          26.00000000 W

F2 - Processing parameters
SI                  65536
SF            500.1300121 MHz
WDW                    EM
SSB       0
LB                   0.30 Hz
GB        0
PC                   1.00
```

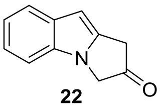

22

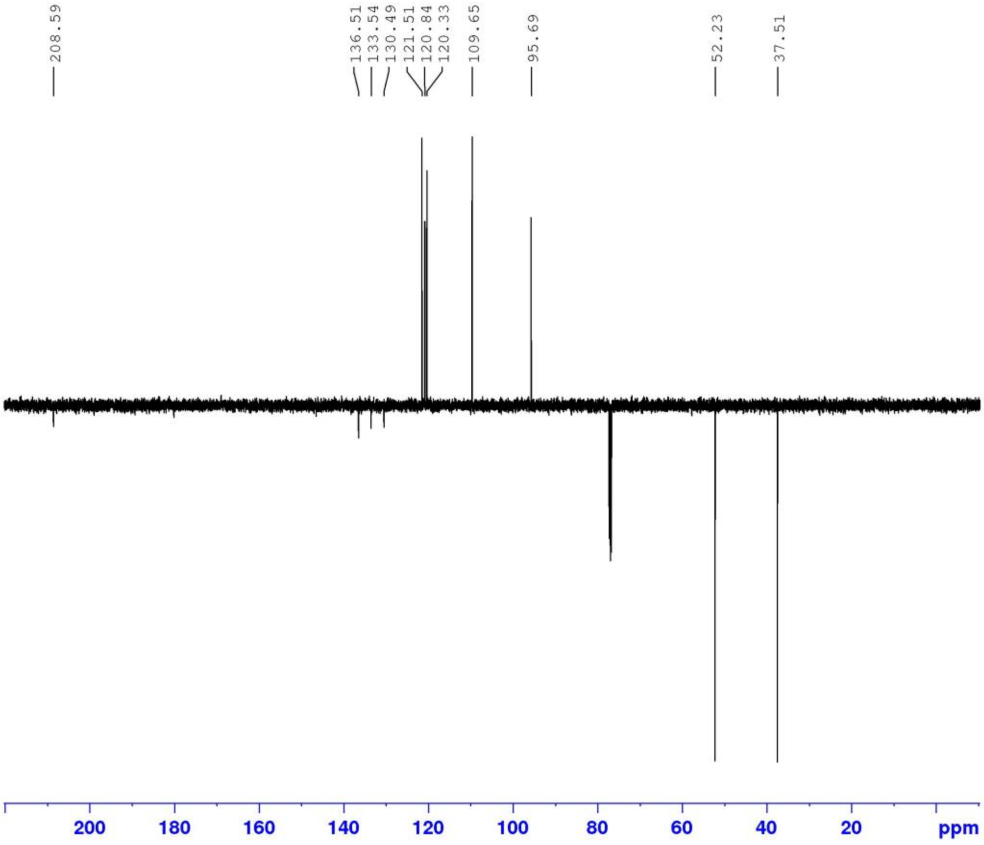


```
Current Data Parameters
NAME          Compound 22
EXPNO                   2
PROCNO                  1

F2 - Acquisition Parameters
Date_            20190508
Time                 9.10 h
INSTRUM             spect
PROBHD   Z800701_0108 (
PULPROG              jmod
TD                  65536
SOLVENT             CDCl3
NS                    176
DS                      4
SWH             31250.000 Hz
FIDRES           0.953674 Hz
AQ              1.0485760 sec
RG                 198.17
DW                 16.000 usec
DE                   6.50 usec
TE                  298.0 K
CNST2         145.0000000
CNST11          1.0000000
D1             2.00000000 sec
D20            0.00689655 sec
TD0                     1
SFO1          125.7703643 MHz
NUC1                  13C
P1                   8.00 usec
P2                  16.00 usec
PLW1          53.00000000 W
SFO2          500.1320005 MHz
NUC2                   1H
CPDPRG[2          waltz16
PCPD2               80.00 usec
PLW2          28.00000000 W
PLW12          0.42017001 W

F2 - Processing parameters
SI                  32768
SF            125.7577940 MHz
WDW                    EM
SSB       0
LB                   1.00 Hz
GB        0
PC                   1.40
```

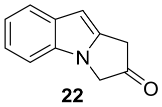

22

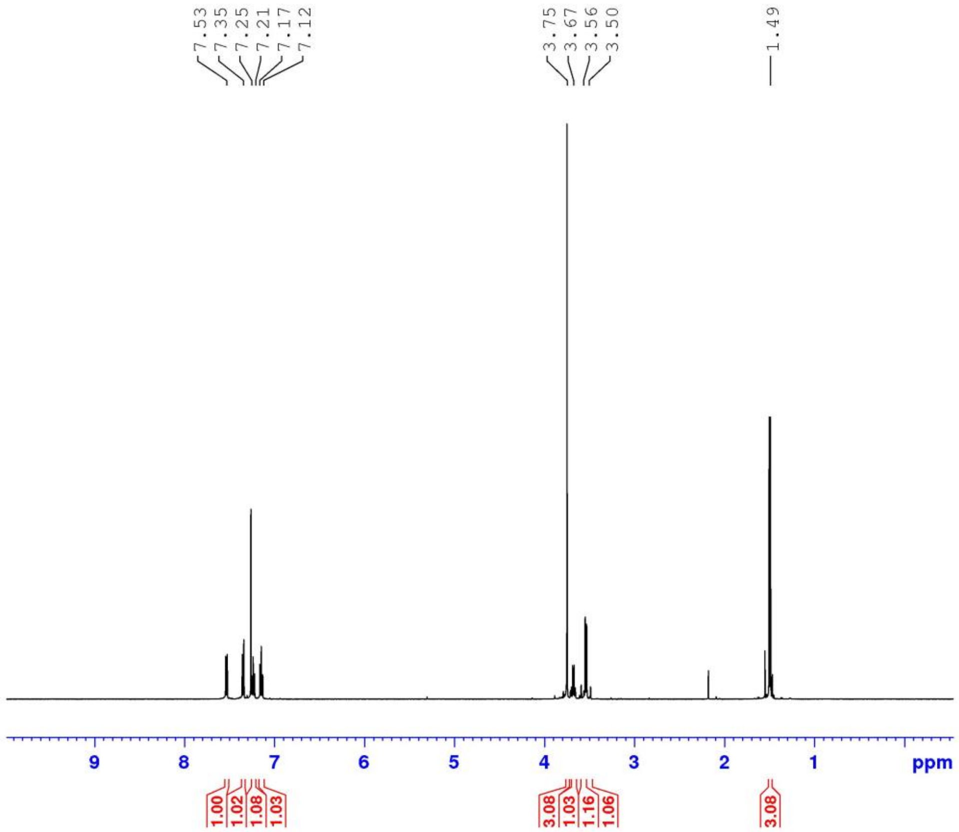

Current Data Parameters
NAME Compound 24
EXPNO 1
PROCNO 1

F2 - Acquisition Parameters
Date_ 20190326
Time 8.47 h
INSTRUM spect
PROBHD Z800701_0108 (
PULPROG zg30
TD 65536
SOLVENT CDCl3
NS 16
DS 2
SWH 10000.000 Hz
FIDRES 0.305176 Hz
AQ 3.2767999 sec
RG 198.17
DW 50.000 usec
DE 6.50 usec
TE 298.0 K
D1 1.00000000 sec
TD0 1
SFO1 500.1330883 MHz
NUC1 1H
P1 10.00 usec
PLW1 26.00000000 W

F2 - Processing parameters
SI 65536
SF 500.1300120 MHz
WDW EM
SSB 0
LB 0.30 Hz
GB 0
PC 1.00

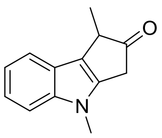

24

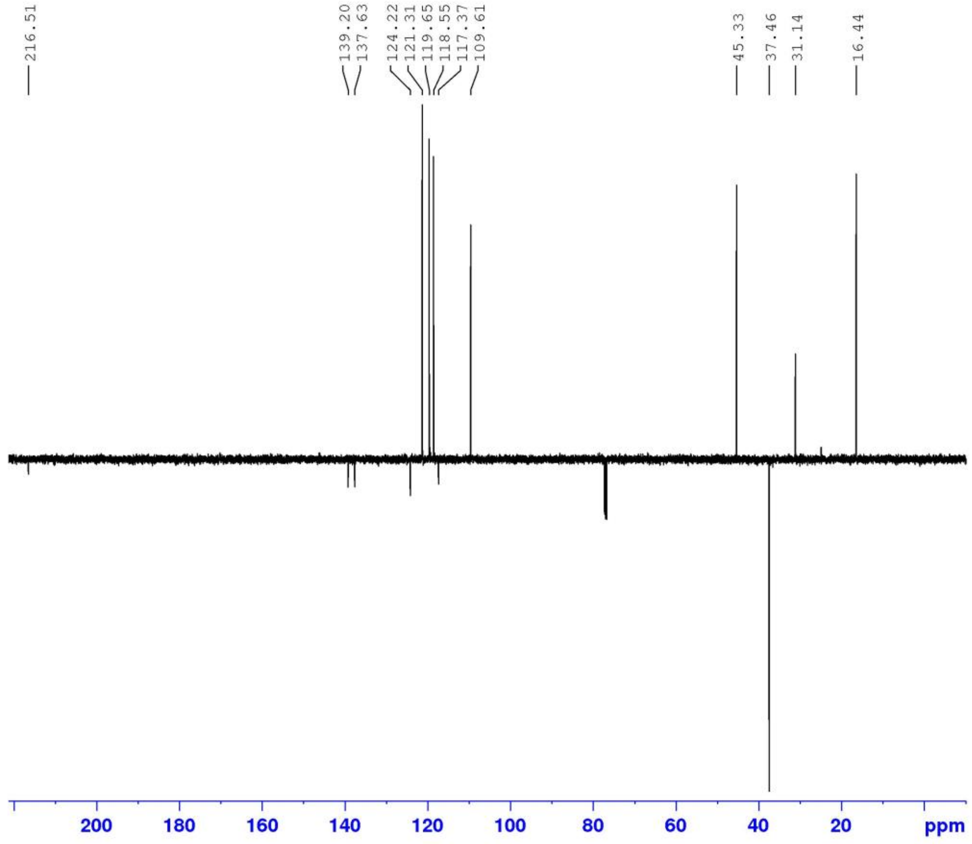

Current Data Parameters
NAME Compound 24
EXPNO 2
PROCNO 1

F2 - Acquisition Parameters
Date_ 20190326
Time 8.59 h
INSTRUM spect
PROBHD Z800701_0108 (
PULPROG jmod
TD 65536
SOLVENT CDCl3
NS 63
DS 4
SWH 31250.000 Hz
FIDRES 0.953674 Hz
AQ 1.0485760 sec
RG 198.17
DW 16.000 usec
DE 6.50 usec
TE 298.0 K
CNST2 145.0000000
CNST11 1.0000000
D1 2.00000000 sec
D20 0.00689655 sec
TD0 1
SFO1 125.7703643 MHz
NUC1 13C
P1 8.00 usec
P2 16.00 usec
PLW1 53.00000000 W
SFO2 500.1320005 MHz
NUC2 1H
CPDPRG[2 waltz16
PCPD2 80.00 usec
PLW2 28.00000000 W
PLW12 0.42017001 W

F2 - Processing parameters
SI 32768
SF 125.7578008 MHz
WDW EM
SSB 0
LB 1.00 Hz
GB 0
PC 1.40

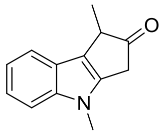

24

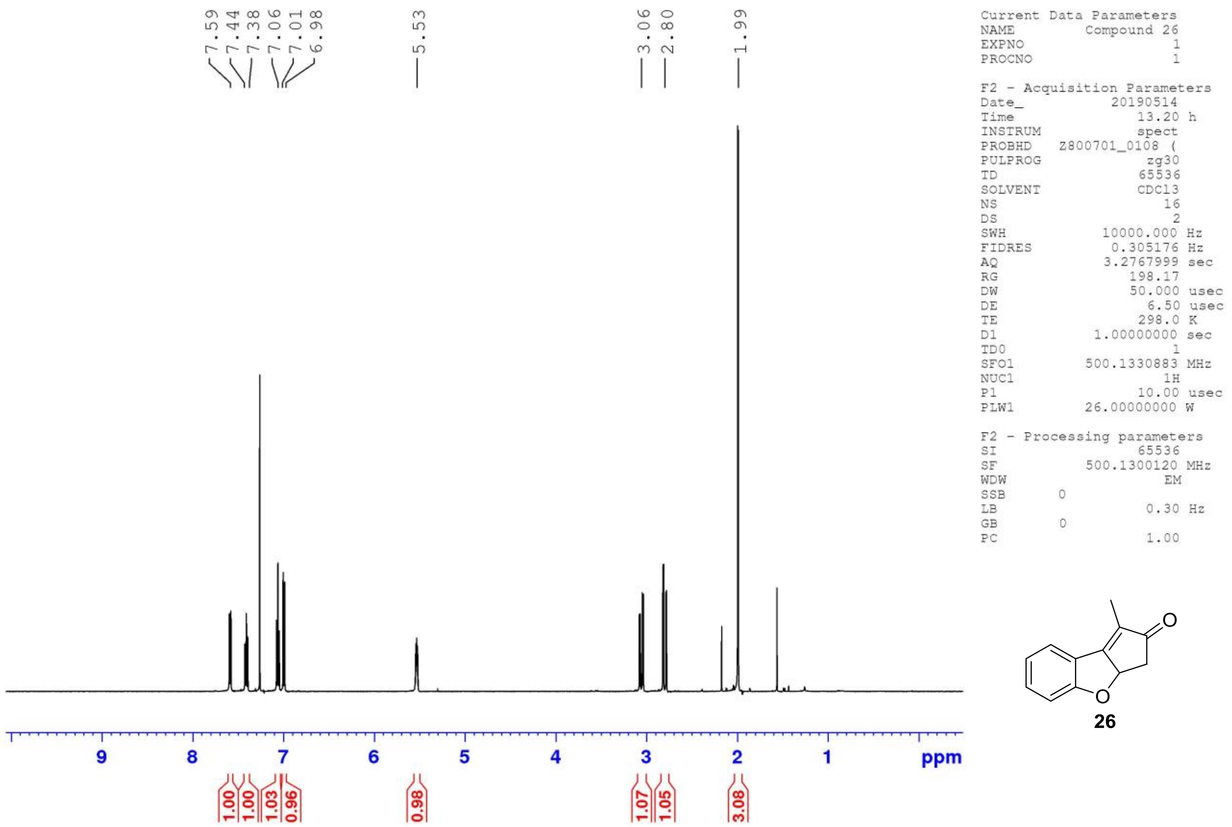
7.59
7.44
7.38
7.06
7.01
6.98
5.53
3.06
2.80
1.99
9 8 7 6 5 4 3 2 1 ppm
1.00 1.00 1.03 0.96 0.98 1.07 1.05 3.08
Current Data Parameters
NAME Compound 26
EXPNO 1
PROCNO 1
F2 - Acquisition Parameters
Date_ 20190514
Time 13.20 h
INSTRUM spect
PROBHD Z800701_0108 (
PULPROG zg30
TD 65536
SOLVENT CDCl3
NS 16
DS 2
SWH 10000.000 Hz
FIDRES 0.305176 Hz
AQ 3.2767999 sec
RG 198.17
DW 50.000 usec
DE 6.50 usec
TE 298.0 K
D1 1.00000000 sec
TD0 1
SFO1 500.1330883 MHz
NUC1 1H
P1 10.00 usec
PLW1 26.00000000 W
F2 - Processing parameters
SI 65536
SF 500.1300120 MHz
WDW EM
SSB 0
LB 0.30 Hz
GB 0
PC 1.00
O
O
26

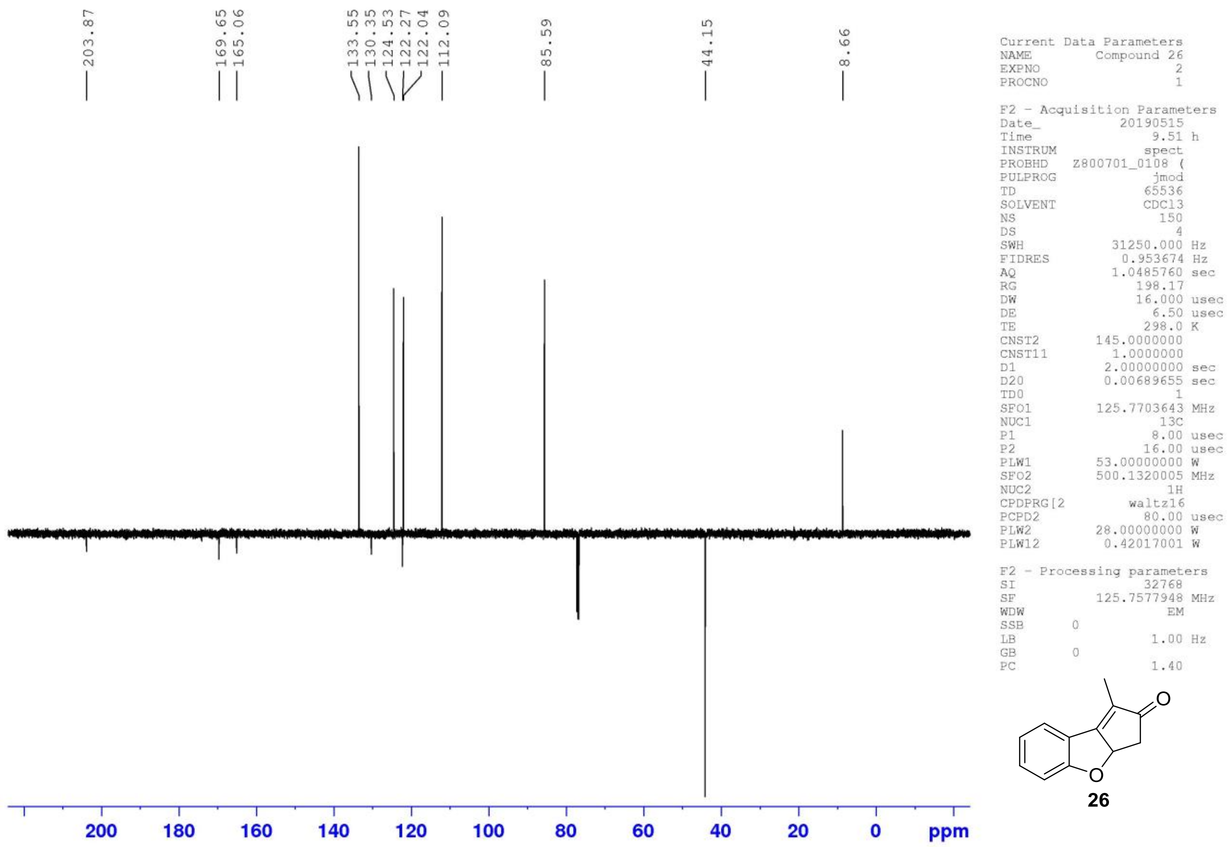
203.87
169.65
165.06
133.55
130.35
124.53
122.27
122.04
112.09
85.59
44.15
8.66
200 180 160 140 120 100 80 60 40 20 0 ppm
Current Data Parameters
NAME Compound 26
EXPNO 2
PROCNO 1
F2 - Acquisition Parameters
Date_ 20190515
Time 9.51 h
INSTRUM spect
PROBHD Z800701_0108 (
PULPROG jmod
TD 65536
SOLVENT CDCl3
NS 150
DS 4
SWH 31250.000 Hz
FIDRES 0.953674 Hz
AQ 1.0485760 sec
RG 198.17
DW 16.000 usec
DE 6.50 usec
TE 298.0 K
CNST2 145.0000000
CNST11 1.0000000
D1 2.00000000 sec
D20 0.00689655 sec
TD0 1
SFO1 125.7703643 MHz
NUC1 13C
P1 8.00 usec
P2 16.00 usec
PLW1 53.00000000 W
SFO2 500.1320005 MHz
NUC2 1H
CPDPRG[2 waltz16
PCPD2 80.00 usec
PLW2 28.00000000 W
PLW12 0.42017001 W
F2 - Processing parameters
SI 32768
SF 125.7577948 MHz
WDW EM
SSB 0
LB 1.00 Hz
GB 0
PC 1.40
O
O
26

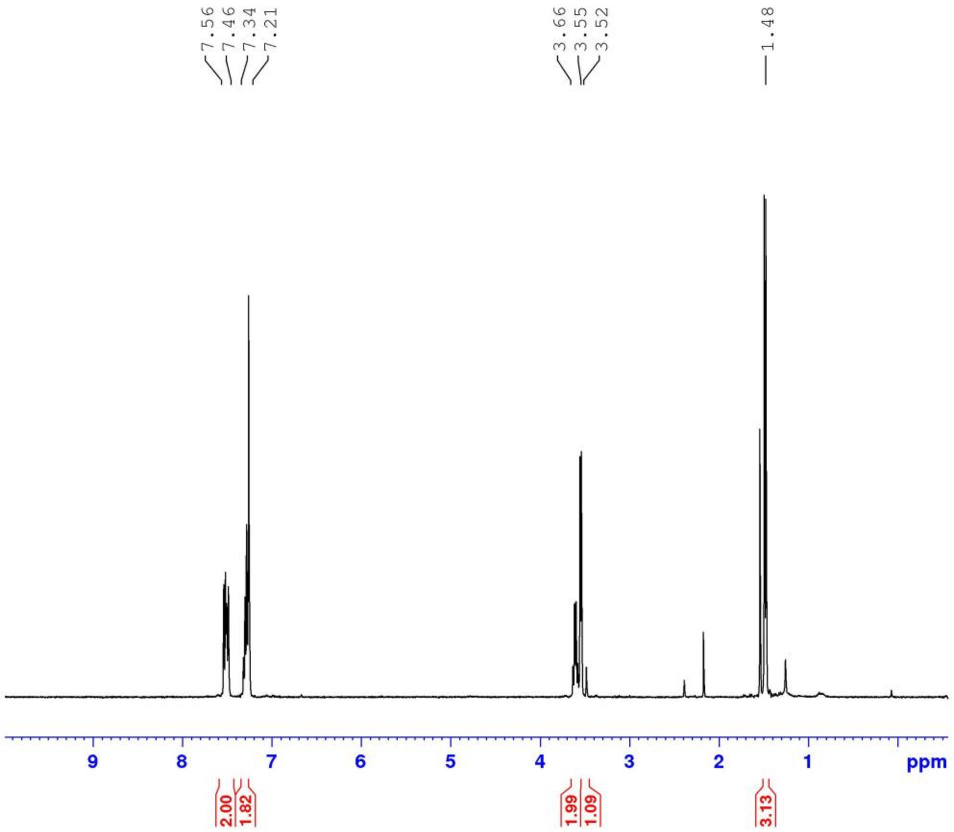


```
Current Data Parameters
NAME        Compound  27
EXPNO                  1
PROCNO                 1

F2 - Acquisition Parameters
Date_           20190809
Time               14.03
INSTRUM            spect
PROBHD   5 mm QNP   1H/1
PULPROG             zg30
TD                 65536
SOLVENT            CDCl3
NS                    16
DS                     2
SWH             8278.146 Hz
FIDRES          0.126314 Hz
AQ             3.9583745 sec
RG                   512
DW                60.400 usec
DE                  6.50 usec
TE                 298.0 K
D1            1.00000000 sec
TD0                    1

======== CHANNEL f1 ========
NUC1                  1H
P1                 10.00 usec
PL1                 0.50 dB
SFO1         400.1324710 MHz

F2 - Processing parameters
SI                 32768
SF           400.1300116 MHz
WDW                   EM
SSB      0
LB                  0.30 Hz
GB       0
PC                  1.00
```

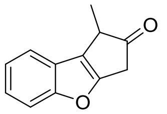

27

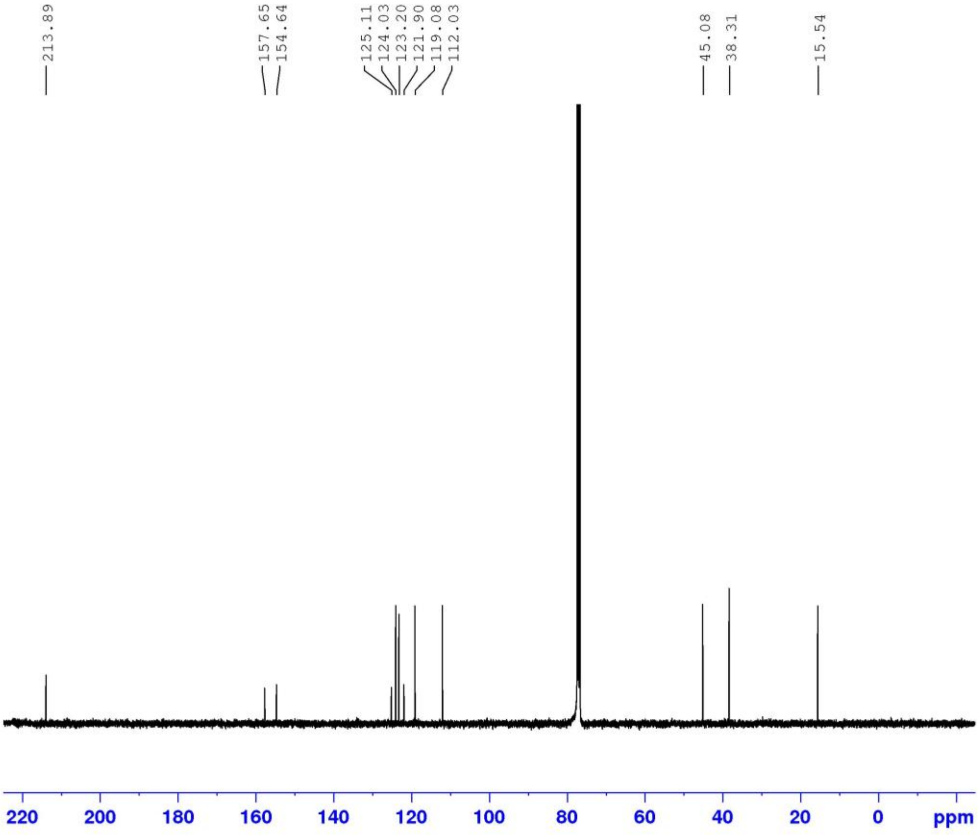


```
Current Data Parameters
NAME        Compound 27
EXPNO                 2
PROCNO                1

F2 - Acquisition Parameters
Date_          20190810
Time               8.26
INSTRUM           spect
PROBHD   5 mm QNP  1H/1
PULPROG          zgpg30
TD                65536
SOLVENT           CDCl3
NS                16384
DS                    4
SWH           25125.629 Hz
FIDRES         0.383387 Hz
AQ            1.3041664 sec
RG                  512
DW               19.900 usec
DE                 6.50 usec
TE                298.0 K
D1           2.00000000 sec
D11          0.03000000 sec
TD0                   1

======== CHANNEL f1 ========
NUC1                13C
P1                 6.50 usec
PL1        0 dB
SFO1        100.6228298 MHz

======== CHANNEL f2 ========
CPDPRG[2          waltz16
NUC2                   1H
PCPD2               80.00 usec
PL2                  1.70 dB
PL12                19.76 dB
PL13                21.00 dB
SFO2          400.1316005 MHz

F2 - Processing parameters
SI                 32768
SF           100.6127703 MHz
WDW                   EM
SSB      0
LB                  1.00 Hz
GB       0
PC                  1.40
```

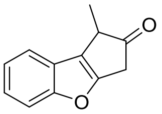

27
